# Conservation and diversity of the pollen microbiome of Pan-American maize using PacBio and MiSeq

**DOI:** 10.3389/fmicb.2023.1276241

**Published:** 2023-12-21

**Authors:** Eman M. Khalaf, Anuja Shrestha, Michelle Reid, Benjamin J. McFadyen, Manish N. Raizada

**Affiliations:** ^1^Department of Plant Agriculture, University of Guelph, Guelph, ON, Canada; ^2^Department of Microbiology and Immunology, Faculty of Pharmacy, Damanhour University, Damanhour, Egypt

**Keywords:** pollen, microbiome, maize, landraces, conservation, PacBio, MiSeq, metagenomics

## Abstract

Pollen is a vector for diversification, fitness-selection, and transmission of plant genetic material. The extent to which the pollen microbiome may contribute to host diversification is largely unknown, because pollen microbiome diversity within a plant species has not been reported, and studies have been limited to conventional short-read 16S rRNA gene sequencing (e.g., V4-MiSeq) which suffers from poor taxonomic resolution. Here we report the pollen microbiomes of 16 primitive and traditional accessions of maize (corn) selected by indigenous peoples across the Americas, along with the modern U.S. inbred B73. The maize pollen microbiome has not previously been reported. The pollen microbiomes were identified using full-length (FL) 16S rRNA gene PacBio SMRT sequencing compared to V4-MiSeq. The Pan-American maize pollen microbiome encompasses 765 taxa spanning 39 genera and 46 species, including known plant growth promoters, insect-obligates, plant pathogens, nitrogen-fixers and biocontrol agents. Eleven genera and 13 species composed the core microbiome. Of 765 taxa, 63% belonged to only four genera: 28% were *Pantoea*, 15% were *Lactococcus*, 11% were *Pseudomonas,* and 10% were *Erwinia*. Interestingly, of the 215 *Pantoea* taxa, 180 belonged to a single species, *P. ananatis*. Surprisingly, the diversity within *P. ananatis* ranged nearly 10-fold amongst the maize accessions analyzed (those with ≥3 replicates), despite being grown in a common field. The highest diversity within *P. ananatis* occurred in accessions that originated near the center of diversity of domesticated maize, with reduced diversity associated with the north–south migration of maize. This sub-species diversity was revealed by FL-PacBio but missed by V4-MiSeq. V4-MiSeq also mis-identified some dominant genera captured by FL-PacBio. The study, though limited to a single season and common field, provides initial evidence that pollen microbiomes reflect evolutionary and migratory relationships of their host plants.

## Introduction

In flowering plants, pollen transmits genetic material to future generations (Hafidh and Honys, 2021). The pollen is the male gametophyte that, upon landing on the female, extends a tube (pollen tube) inside the maternal style channel to deliver sperm nuclei to the ovary to facilitate double fertilization, culminating in seed production (Dresselhaus et al., 2016). Pollen is also a vector for genetic diversification of progeny by transmitting gametes derived from meiotic recombination, independent assortment, and transposition (Raizada et al., 2001; Barrett, 2002, 2010; Warman et al., 2020). Furthermore, in many plant species, pollen acts to disperse male gametes large distances via wind or pollinators (Wessinger, 2021; Rodrigues et al., 2023).

Pollen is an independent multicellular organism, separate from the sporophytic generation of plants, and it must remain viable during dispersal against environmental stress (e.g., heat, pathogens) (Hafidh and Honys, 2021). The large numbers of haploid male gametes and their genetic diversity may drive competition between pollen and ultimately evolutionary selection to promote plant survival. Specifically, individual pollen may compete with one another for improved viability during dispersal, while pollen tubes may compete for successful elongation and delivery of nuclei to awaiting eggs (Mulcahy, 1978, 1979; Walsh and Charlesworth, 1992; Beaudry et al., 2020; Williams and Oliveira, 2020).

Microbiomes are now well established as contributing to eukaryotic genetic diversity (Tierney et al., 2019). The multi-layer protective structure of the pollen wall that protects a nutrient rich core may be an ideal habitat for microbes (Pacini and Hesse, 2005; Shi et al., 2015; Obersteiner et al., 2016; Frank et al., 2017; Ourani-Pourdashti and Azadi, 2021). Indeed, a small number of pioneering studies in the literature are revealing that pollen possess microbiomes (Manirajan et al., 2016, 2018, 2019; Obersteiner et al., 2016; Oteros et al., 2018; McFrederick and Rehan, 2019; Wu et al., 2022). The host plant species and pollination type substantially impact the assemblage of microbial communities associated with pollen (Manirajan et al., 2018). However, the extent to which pollen-associated microbes are transmitted to progeny remains largely unreported, though a recent study convincingly demonstrated the transmission of a bacterial strain from maize pollen to progeny seed (Wu et al., 2022). Though pollen is known to play a fundamental role in plant genetic diversification, little is known about the extent to which the pollen microbiome contributes to this diversification.

Maize (*Zea mays* L.) is one of the world’s three most important food crops globally (Gong et al., 2015; García-Lara and Serna-Saldivar, 2019). It is a wind-pollinated grass crop, known to shed large numbers of pollen grains (10^11^ to 10^13^ per ha) that can be collected easily, each with a relatively large weight (150–500 ng) and large surface area (80−125 μm diameter) (Kalinowski et al., 2002; Hofmann et al., 2014; Gong et al., 2015; Umurzokov et al., 2021) that may facilitate microbial transmission, making it an ideal model to study pollen microbiomes. After pollination, maize style tissue (silk) has a rich and diverse microbiome (Khalaf et al., 2021); the style with its pollen tube transmits gametes to embryos and endosperm, giving rise to seeds. However, the extent to which silk and pollen tube microbiome diversity originates from pollen is not known (Cullen et al., 2021; Khalaf et al., 2021). Indeed, nothing has been reported about the maize pollen microbiome, to the best of our knowledge.

Previous studies have focused on pollen microbiome diversity across host species, but not within a host species; whether pollen microbiomes reflect evolutionary and migratory relationships of their host plants is unknown. Previously, our lab provided initial evidence that *Zea* seeds possess microbiomes that may have co-evolved with the host during natural selection, domestication, and migration across the Americas (Johnston-Monje and Raizada, 2011), suggestive of long-term inheritance via gametes. Maize has a well-documented history of evolution, domestication, migration, and selection by humans (Bedoya et al., 2017). Around 9,000 years ago, maize was domesticated in southwestern Mexico from tropical wild teosinte grasses, primarily *Zea mays* ssp. *parviglumis* with a minor contribution from *Zea mays* ssp. *mexicana* (Matsuoka et al., 2002; Hastorf, 2009; Piperno et al., 2009; Van Heerwaarden et al., 2011; Warburton et al., 2011). Following subsequent diversification in the Oaxaca region of the central highland plateau of Mexico, maize was migrated north and south across different latitudes and altitudes, and developed by indigenous peoples into landraces adapted to local environments and needs (Matsuoka et al., 2002; Perales et al., 2005; Ruiz Corral et al., 2008; Vigouroux et al., 2008; Bedoya et al., 2017; Kistler et al., 2020).

Microbiome profiling is based on throughput sequencing (HTS) of the 16S rRNA gene (Zhang et al., 2023). The past decade has witnessed unprecedented evolution in HTS platforms (Gupta and Verma, 2019; Satam et al., 2023). Technically, there are two generations of HTS: short-read (e.g. Illumina MiSeq), and long-read [e.g., Pacific Biosciences (PacBio)] sequencing (Franzén et al., 2015; Wagner et al., 2016; Callahan et al., 2019). Most prior microbiome reports were limited by short-read 16S rRNA gene amplicons [e.g., hypervariable V4 region (V4-MiSeq)] (Greay et al., 2019; Palkova et al., 2021) that lacked the fine resolution captured by the long-read sequencing platform [full-length 16S rRNA gene -PacBio (FL-PacBio)] needed to accurately reveal taxonomic diversity at the species and subspecies level (Callahan et al., 2019). Only a few studies have compared microbiome results from full-length 16S rRNA gene PacBio to partial 16S rRNA gene MiSeq sequencing (Buetas et al., 2023). Furthermore, to the best of our knowledge, the pollen microbiome has only been reported in the literature by using partial 16S rRNA gene sequencing (Manirajan et al., 2016, 2018, 2019; Obersteiner et al., 2016; Oteros et al., 2018; McFrederick and Rehan, 2019; Wu et al., 2022).

Here we hypothesized that: (1) maize pollen from across the Americas has a core pollen microbiome; (2) that full-length 16S rRNA gene sequencing would reveal diversity at the sub-species level than previously reported in pollen using V4-16S rRNA gene sequencing; and (3) that the relationships between pollen microbiomes amongst maize landraces reflect evolutionary and migratory relationships of their hosts.

## Materials and methods

### Selection criteria and sources of maize accessions

**Figure 1:**
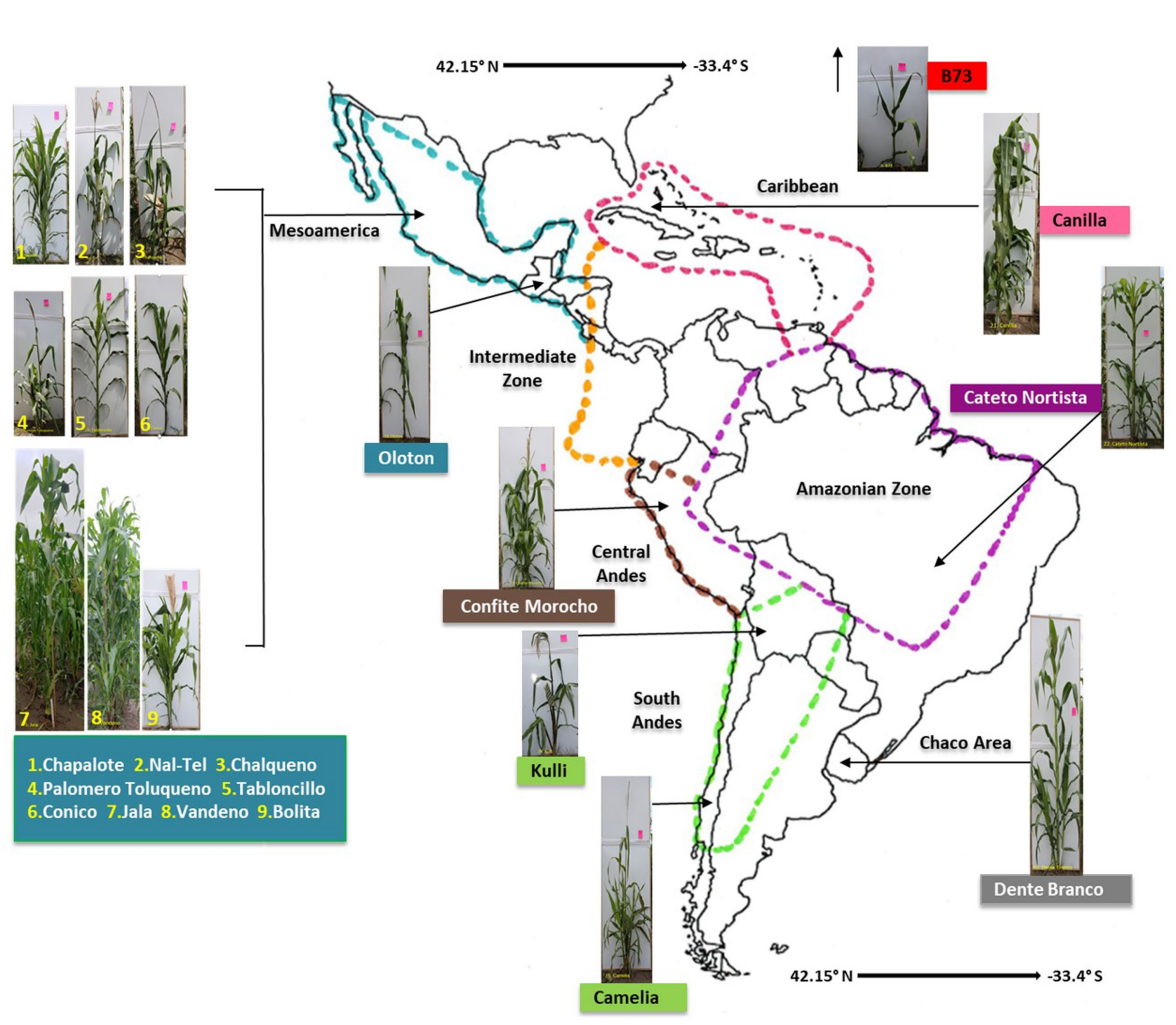
A map of the Americas locating the origin of the 17 maize accessions used in this study. The map is adapted from Bedoya et al. (2017). Please see Table 1 and Supplementary Table S1 for complete source details.

In total, 17 maize accessions were used in this study (Figure 1, Table 1), which were selected based on balancing multiple criteria. Specifically, the accessions selected spanned wide genetic diversity in maize with clear phylogenetic relationships among one another (Matsuoka et al., 2002; Bedoya et al., 2017), representing diverse latitudes, altitudes, agro-ecological environments, and timespan since cultivation. Importance was placed on accessions that belonged to the center of maize diversification in Oaxaca, Mexico, and/or which belonged to different migration routes from this center, i.e., north, or south. Finally, accessions were selected based on their unique phenotypes and historical importance to their respective indigenous peoples (Supplementary Table S1).

**Table 1:** Information about maize accessions used in this study.

*Zea* Genotype	Category	Seed Origin^#^	Latitude	Longitude	Rainfall	Population^*^	Accession ID
B73	Modern Inbred	United States, Iowa	42.15	−95.84	Moderate	Derived from Highland Mexico	PI 550473^1^
Chapalote	Landrace, Primitive	Mexico (SMO)	24.86	−107.42	Moderate	Lowland Western & Northern Mexico	NSL 2833^1^
Jala	Landrace	Mexico (SMO)	21.09	−104.43	Moderate	Lowland Western & Northern Mexico	CIMMYTMA 2246^2^
Canilla	Landrace	Cuba	20.9	−76.25	Moderate	Caribbean^†^	CIMMYTMA 5393^2^
Nal-Tel	Landrace, Primitive	Mexico (S/SW)	20.25	−89.65	Dry	South America - Non-Andean South America	CIMMYTMA 2357^2^
Tabloncillo	Landrace, Primitive	Mexico (SMO)	19.45	−103.28	Wet	Highland Mexico	CIMMYTMA 10493^2^
Chalqueno	Landrace	Mexico (CV)	19.283	−99.65	Wet	Highland Mexico	CIMMYTMA 8851^2^
Conico	Landrace	Mexico (CV)	19.283	−99.65	Wet	Highland Mexico	CIMMYTMA 2232^2^
Palomero Toluqueno	Landrace, Primitive	Mexico (CV)	19.283	−99.667	Wet	Highland Mexico	CIMMYTMA 6756^2^
Vandeno	Landrace	Mexico (S/SW)	17.52	−101.28	Moderate	Guatemala and Southern Lowland Mexico	CIMMYTMA 177^2^
Bolita	Landrace	Mexico (SMO)	15.75	−96.517	Dry	Highland Mexico	CIMMYTMA 2592^2^
Oloton	Landrace	Guatemala	14.633	−90.517	Moderate	Guatemala and Southern Lowland Mexico	CIMMYTMA 2510^2^
Confite Morocho	Landrace, Primitive	Peru	−12.77	−75.03	Wet	South America - Andean or Other South America^†^	CIMMYTMA 8381^2^
Cateto Nortista	Landrace	Brazil	−16.667	−49.255	Wet	South America - Non-Andean South America^†^	CIMMYTMA 26373^2^
Kulli	Landrace, Primitive	Bolivia	−18.18	−65	Moderate	South America - Andean or Other South America^†^	CIMMYTMA 14235^2^
Dente Branco	Landrace	Uruguay	−32.683	−58.133	Wet	South America - Non-Andean South America^†^	CIMMYTMA 6162^2^
Camelia	Landrace, Primitive	Chile	−33.45	−70.667	Dry	South America - Andean or Non-Andean South America^†^	CIMMYTMA 15218^2^

1 Denotes seeds obtained from USDA.

2 Denotes seeds obtained from CIMMYT, Mexico.

# S/SW = Southern and Southwestern; CV = Central Valleys; SMO=Sierra Madre Occidental.

* Population structure is from Vigouroux et al. (2008) and Matsuoka et al. (2002).

^†^These accessions were not in Vigouroux et al. (2008) or Matsuoka et al. (2002), but were placed here into Matsuoka classifications based on their geographic origin.

Included in the final choice of accessions at the center of maize diversification in Mexico and Central America was the ancient Mexican Jala landrace which possesses the largest cobs in the world (Rice, 2007). Vandeno was selected, because it is a Mexican ancestor of multiple modern maize varieties (Bedoya et al., 2017). Nal-Tel was selected as it is a primitive popcorn that formed the basis of the ancient Mayan peoples’ diets in the Yucatan Peninsula (Wellhausen et al., 1952; Turrent-Fernández and Serratos-Hernández, 2004; Hufford et al., 2012; Santillán-Fernández et al., 2021). Palomero Toluqueno was selected as it is the ancient progenitor of modern landraces in the Mexican highlands (Hufford et al., 2012; Perez-Limón et al., 2022). Landrace Oloton was selected, because it is a landrace of the Guatemalan highlands that formed the staple food for the indigenous Mixe people in the mountains of the Oaxaca region in southern Mexico (Pskowski, 2019).

We then selected accessions that resulted from the northward and southward migration of maize by indigenous peoples, away from the diversification center. On the northern route, we selected Chapalote (Hufford et al., 2012), one of the first maize accessions introduced to the United States over 2000 years ago (Da Fonseca et al., 2015). We also selected the U.S inbred B73, as it was the founder of North American commercial hybrid maize varieties, and it was included to have an accession resulting from modern breeding (Bornowski et al., 2021). On the southern migration route, we selected landraces migrated by indigenous peoples into South America, including: Confite Morocho, a primitive ancestor of maize grown in the Andes mountains of central Peru (Grobman et al., 1961); Cateto Nortista, a mid-altitude landrace from central Brazil (Bedoya et al., 2017); Kulli, a landrace from the Bolivian highlands (Cuevas Montilla et al., 2011); Dente Branco, a Uruguayan lowland landrace with origins in the United States (Bedoya et al., 2017); and Camelia, a lowland landrace from Chile (Timothy et al., 1961).

Landraces were also dispersed into the Caribbean, and to include this region, we selected Canilla, a lowland landrace from Cuba, grown by the Taino peoples in pre-Columbian times (Hatheway, 1957).

Among the 17 accessions, 15 were provided by the Maize Germplasm Bank of the International Maize and Wheat Improvement Center (CIMMYT), Mexico, and the remaining two were received from the National Plant Germplasm System (NPGS) of the U.S. Department of Agriculture (Supplementary Table S1).

### Growth conditions and experimental design

Indoor growth room: As noted above, all these maize accessions (except B73) were tropical plants and required a short-day length between V5 to V8 growth stages to induce flowering. V5 and V8 growth stages are vegetative stages of maize, defined as having 5 or 8 leaves with visible leaf collars, respectively. Therefore, to precisely control daylength requirements, the seeds were initially grown under growth room conditions (lacking ambient sunlight) in 5×5 inch biodegradable pots filled with a mixture of Sunshine Mix (LA4, Sungrow®Horticulture, Brantford, Ontario, Canada) and field soil from the Elora Research Station (latitude: 43°41’ 3.59” N; longitude: -80° 25’ 22.79” W), Elora, Ontario, Canada. A 14/10-h day-night period was adjusted until the seedlings reached the V5 growth stage and then the photoperiod was reduced to 10 h until the V8 growth stage. The source of light in the growth room included fluorescent lighting (18 ET9/4/850 bulbs, GE), supplemented with LED lights (9.5 A19/DIM/0/827/G4 1,100 Lumen 2,700 K bulbs, Osram) to achieve a light intensity of 425-515 μmol m^-2^ s^-1^ at pot level. To promote uniform distribution of light to each plant, the pots were moved around twice per week. Irrigation and fertilization (20:20:20 fertilizer with micronutrients) (Plant-Prod 20-20-20 Classic, Product Number 10529, Brampton, Ontario, Canada) were done manually.

Field conditions: At the V8 growth stage, 780 large maize seedlings were transported to the field at the Elora Research Station, University of Guelph, and were left in the field for 3 days (2-3 h/day under direct sunlight and remaining time under shade) in a trailer to ensure the plants acclimated to the low humidity and summer sunlight conditions before transplanting in the field on July 10, 2019. A randomized block design with five replications with six plants per block was used. The field was fertilized with pre-application of 160 kg/ha of N, 60 kg/ha of P_2_O_5_, 80 kg/ha of K_2_O, and 10 kg/ha of S along with pre-application of the herbicides, Primextra (4.0 l/ha) and Callisto (0.3 l/ha). Irrigation was supplied manually every day for one week after transplanting. Relevant soil test results at two depths (n = 6) prior to the addition of fertilizers is provided in Supplementary Table S2. Weather data during the field trial were obtained from the Elora Weather Station^1^ and are provided in Supplementary Tables S3A−D.

### Pollen harvesting

The harvesting of pollen was done from August 13 until October 15, 2019, except for rainy days where pollen collection was avoided. Upon pollen shedding, tassels were bagged every afternoon and then collected the next morning. A single tassel was bagged 5 times on average. Hence, from 6 plants of each maize accession in each block, pollen was harvested 5 times (17 accessions × 5 blocks × 6 plants × 5 times), resulting in over 2,500 collected pollen bags. The field trial was designed to obtain 5 replicates (represented here as blocks) per each maize accession. However, zero or tiny amounts of pollen could be harvested from some plants, and hence we estimate that each pollen sample represented pollen pooled from 3-5 plants per block. Furthermore, pollen from some blocks was insufficient, and hence out of 85 attempted samples, 54 were successful. Furthermore, the maize accessions varied in pollen shed per block, from five full 2 mL screw-capped tubes for good pollen shedding accessions (e.g., B73, Chalqueno, Bolita, Cateto Nortista, Dente Branco, and Conico), compared to approximately half of the tube for poor shedders (e.g., Jala, Nal-Tel, Canilla, Tabloncillo, Vandeno, Camelia). After collection, the bags were brought to the lab and then the anthers and other debris were removed using a fine wire mesh. Samples were stored at -80°C for later DNA isolation.

### DNA isolation and quantification

ZymoBIOMIC ™ DNA Miniprep Kits were used to extract DNA from pollen samples (50-100 mg of tissue used) following the manufacturer’s protocol with some steps adapted from a previous study (Simel et al., 1997). Briefly, frozen pollen samples were added to pre-chilled 2.0 mL screw-capped tubes with one-third volume filled with 0.5 mm sterilized glass beads. Lysis buffer from the kit (750 μL) was added immediately to each screw-capped tube and vortexed so that pollen was exposed to lysis buffer before thawing. The samples were processed in a bead mill homogenizer (Catalog #15340163, Fisherbrand™, Ontario, Canada) using pre-optimized conditions (speed = 4.5 strength for 45 s) followed by centrifugation at 12,000−16,000 × *g* for 3 min. The samples were again processed in the bead beater at the same strength for 30 s followed by centrifugation at 12,000−16,000 × *g* for 1 min. An equivalent volume (750 μL) of 25:1 chloroform: isoamyl solution was added, and then the mixture was placed horizontally in an orbital shaker for 10 min at 100 rpm at room temperature, followed by centrifugation at 5500 × *g* for 15 min. The top aqueous solution/supernatant (550−600 μL) from each tube was transferred to new tubes, and then an equal volume of 25:1 chloroform: isoamyl solution was added, followed by shaking horizontally for 10 min at 100 rpm and then centrifugation at 5500 X *g* for 15 min. The supernatant (approx. 400 μL) from each tube was transferred to a Zymo-Spin ™ III-F Filter in a collection tube and then processed using the remaining standard kit protocol. DNA samples were quantified using a Qubit v1.2 fluorometer (Catalog #Q32857, Molecular Probes, Invitrogen by Life Technologies).

### 16S rRNA gene high throughput sequencing

DNA samples were submitted to Genome Quebec (Montreal, Canada) for high throughput 16S rRNA gene sequencing using Illumina MiSeq and the Pacific Bioscience (PacBio) single-molecule real-time (SMRT) - Sequel II system. For Illumina MiSeq (Illumina MiSeq PE250), the hypervariable region V4 of 16S rRNA gene was amplified using the barcoded primer set, 515F 5′-GTGCCAGCMGCCGCGGTAA-3′, 806R 5′-GGACTACHVHHHTWTCTAAT-3′. The reaction mixture for each sample contained 25 μL of GoTaq® Colorless Master Mix (Promega, Madison, Wisconsin, United States), 5 μL of BSA (5 ng/μl), 1 μL of 10 μM forward primer, 1 μL of 10 μM reverse primer, 1 μL of 25 μM pPNA, 1 μL of 25 μM mPNA71 (Lundberg et al., 2013), 13 μL of PCR-grade water, and 3 μL of genomic DNA (5 ng/μl). For bacterial PCR, DNA was denatured at 94°C for 3 min, followed by 30 cycles of 94°C for 45 s, 50°C for 60 s and 72°C for 90 s, with a final extension at 72°C for 10 min. Using agarose gel electrophoresis, the PCR products were evaluated. To fuse CS1/CS2 linker primers to the indices and adapters, additional PCR cycles were applied as follows: an initial denaturation and enzyme activation at 95°C for 10 min, followed by 15 cycles at 95°C for 15 s, 60°C for 30 s, and 72°C for 60 s with a final extension at 72°C for 10 min. Then, the library DNA was sequenced with a MiSeq Reagent Kit v2 (2 × 250 cycles), and FASTQ files were generated for taxonomic analysis. Sequences were generated on a single sequencing run. Demultiplexed Fastq files were received for data analysis.

To obtain taxonomic assignments of higher accuracy, the full-length (V1-V9) 16S rRNA genes were amplified and sequenced using the PacBio SMRT Sequel II system. The following primers were used: 27F 5′-AGRGTTYGATYMTGGCTCAG-3′ and 1492R 5′- RGYTACCTTGTTACGACTT-3′. The libraries were prepared following the standard protocol of Pacific Biosciences (Pacific Biosciences, 2012, 2020) (Accessed April 28, 2023). The DNA damage repair, end repair and SMRT bell ligation steps were performed as described in the standard template preparation protocol provided with the SMRTbell Template Prep Kit 2.0 (Pacific Biosciences, Menlo Park, CA, USA). The first PCR was performed in a final reaction volume of 25 μL using the following components in their final concentrations (information between brackets is the final concentration): 2.5 μL of Qiagen 10X Buffer with 15 mM MgCl_2_ (1X), 1.25 μL of Roche DMSO (5%), 0.5 μL of dNTP mix, 10 mM NEB buffer (0.2 mM), 0.1 μL of Qiagen HotStarTaq 5 U/μl (0.02 U/μl), 0.15 μL of 27Fb-CS1 (0.6 μM), 0.15 μL of 1492Rb-CS2 (0.6 μM), 0.3 μL of PNA clamp − mitochondrial blocker (mPNA 50uM) 5′-GGCAAGTGTTCTTCGGA-3′ (1.2 μM), 0.3 μL of the plastid-blocker (pPNA 50uM) 5′-GGCTCAACCCTGGACAG-3′ (1.2 μM), 1 μL of DNA, and 18.75 μL of H_2_O. For bacterial PCR, DNA was denatured at 96°C for 15 min, followed by 33 cycles of 96°C for 30 s, 75°C for 10 s, 60°C for 30 s, and 72°C for 60 s, with a final extension at 72°C for 10 min. The libraries went through an AMPure bead cleanup (following the SMRTlink calculator procedure) before being sequenced on a PacBio Sequel II instrument at a loading concentration (on-plate) of 200 pM (192 samples pooled) using the standard diffusion loading protocol provided with the Sequel II Sequencing Kit 2.0. A SMRT Cell 8 M with 10-h movies was used with a pre-extension of 30 min (192 samples pooled). The BAM data were generated using SMRT Link v.10.1.0 to create separate BAM files for each sample. The CCS tool from SMRT Link was used to derive the circular consensus sequences (ccs). The Lima tool from SMRT Link was used for demultiplexing to identify barcode sequences and remove them. The demultiplexed BAM files were received from the sequencing facility for subsequent data analysis and interpretation.

### Sequencing data processing and analyses

Sequences generated from PacBio Sequel II and Illumina MiSeq sequencing technologies were received and curated using DADA2 R package (version 1.22.0) (Callahan et al., 2016).

#### V4-Miseq

For V4-MiSeq Illumina sequences, demultiplexed fastq.gz files for forward and reverse sequences were processed using the DADA2 R package (version 1.22.0) (Callahan et al., 2016). Sequences were trimmed and filtered using filterAndTRim function (truncLen = c(245,245), trimLeft = c(19, 20), maxN = 0, maxEE = c(2.2), truncQ = 2, rm.phix = TRUE). To infer amplicon sequence variants (ASVs), independent sample inference was used. Then, the chimeras were removed using the removeBimeraDenovo function. The taxonomy was assigned using the assignTaxonomy function on a training set (silva_nr99_v138.1_train_set.fa.gz and minBoot = 50), followed by applying the addSpecies function on silva_species_assignment_v138.1.fa.gz to assign the taxonomy to the species level with exact matching (100% identity) (McLaren and Callahan, 2021).

#### FL-PacBio

For FL-PacBio sequences, BAM files of CCS were received from Genome Quebec, then FASTQ records were extracted from sequence alignments in BAM files using bedtools bamtofastq conversion utility. FASTQ files were processed using the DADA2 R package (version 1.22.0). Primers 27F and 1492R were removed using the removePrimers function, and sequences were oriented in a consistent direction. Sequences were then filtered and trimmed using the filterAndTRim function (nops, filts, minQ = 3, minLen = 1,000, maxLen = 1,600, maxN = 0, rm.phix = FALSE, maxEE = 2). To infer ASVs, full pooling was used. Then, chimeras were removed using the removeBimeraDenovo function. To assign the taxonomy to the species level, the assignTaxonomy function was applied on the training set (silva_nr99_v138.1_wSpecies_train_set.fa.gz with minBoot = 80).

Singletons were not considered in processing sequences from both sequencing technologies. For sequence alignment and generation of the phylogenetic tree in R, DECIPHER (version 2.22.0) (Wright, 2016) and phangorn (version 2.8.1) R packages (Schliep, 2011) were used, respectively.

A phyloseq object was generated from the output files from each sequencing technology including ASVs, taxonomy, metadata tables, and a phylogenetic Newick tree using the Phyloseq R package (McMurdie and Holmes, 2013). All ASVs that were classified as mitochondrial or chloroplast 16S were filtered along with sequences that belonged to non-bacteria/archaea kingdoms. To manipulate biological strings, the Biostrings R package (version 2.62.0) (Pagès et al., 2022) was used. Alpha diversity metrics were calculated on raw reads from each technology using the microbiome R package (version 1.16.0). Venn diagrams were generated using the VennDiagram R package (version 1.7.1) (Chen and Boutros, 2011). Subsequently, dplyr (1.0.8) and tidyverse (1.3.1) R packages were used for further data manipulation. Extracted ASVs sequences were aligned using MUSCLE v3.8.1551 (Edgar, 2004) in command line, and phylogenetic trees were generated using RAxML-NG v. 1.1.0 (29.11.2021 release) by the Exelixis Lab (Kozlov et al., 2019). Phylogenetic trees were annotated using iTOL online software (Letunic and Bork, 2021). To visualize comparative pollen microbiomes identified by FL-PacBio versus V4-MiSeq sequencing at all bacterial taxonomic levels, microbiome data were normalized using relative abundances (RAs). However, to generate heatmaps associated with phylogenetic trees and other hierarchical clustering heatmaps, inverse hyperbolic sine (IHS) transformation was applied (Bellemare and Wichman, 2020).

### Statistical analyses

To compare the calculated alpha diversity metrics richness, diversity, and evenness of pollen microbiome from each sequencing technology, the Mann–Whitney U test (compare ranks) was applied on calculated alpha diversity metrics at the taxa level; the test parameters were as follows: nonparametric, experimental design: unpaired, *p* value: two-tailed, statistical significance at *p* < 0.05. Statistical mean comparisons of *P. ananatis* taxa and read counts across geographical populations were also calculated using the Mann–Whitney U test. All statistical tests were calculated and visualized in R using dplyr (version 1.1.2) and ggpubr (version 0.6.0) packages.

## Results

### Overview of the Pan-American maize pollen microbiome community

**Figure 2:**
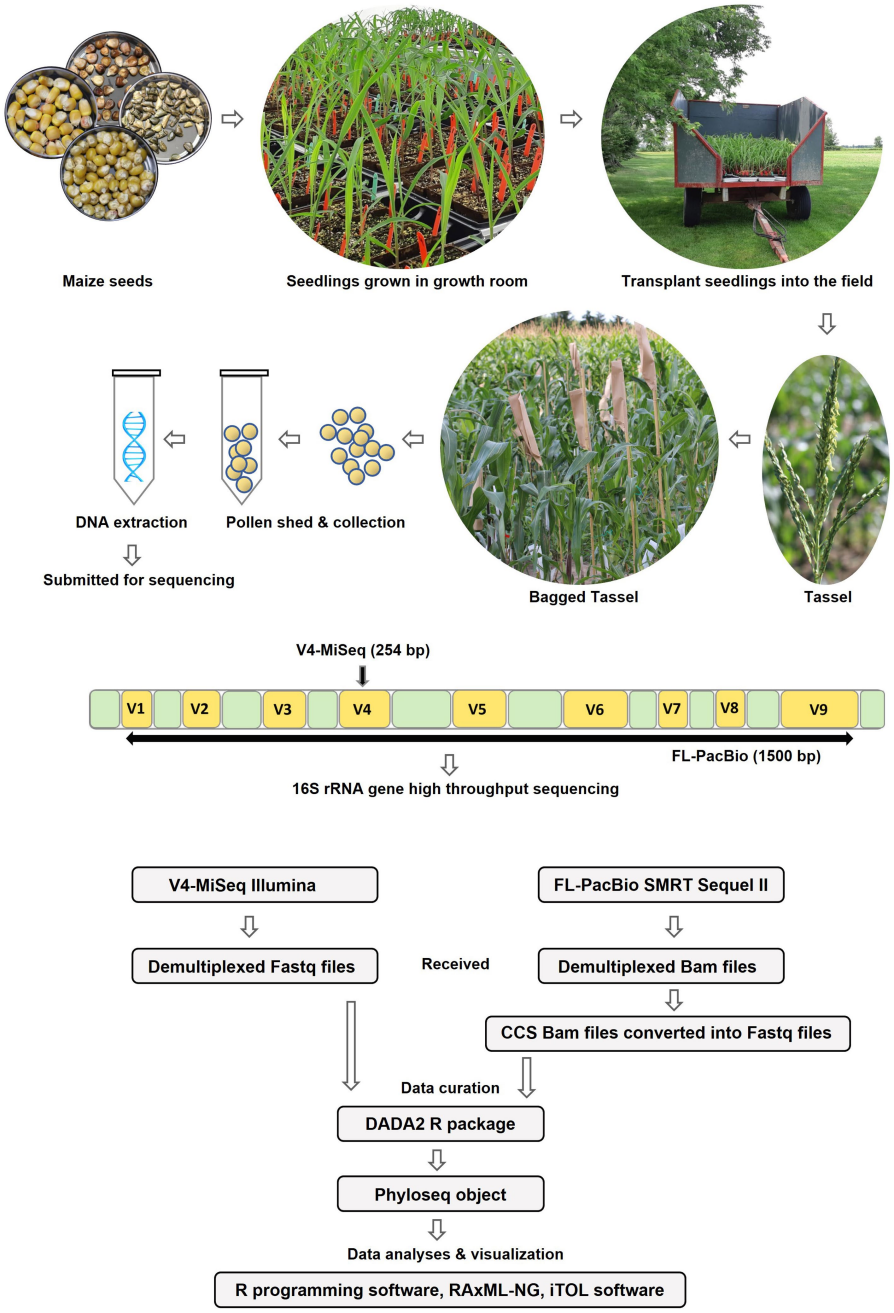
Diagrammatic sketch illustrating the workflow of the study.

To determine the conservation and diversity of the pollen microbiome of maize, the U.S. inbred B73 and 16 primitive and traditional Pan-American landraces spanning diverse latitudes, altitudes, agro-ecological environments, and timespan since cultivation (Figure 1 and Table 1), were grown in a common field in 2019 in 5 randomized blocks (Figure 2). See Methods for criteria of maize selection. Attempts were made to collect pollen from each block (randomly pooled from 3 to 5 plants but not necessarily balanced per plant), but due to very low pollen shed, combined with low genomic DNA yield from pollen, only 54 samples sufficient for high throughput 16S rRNA gene sequencing could be obtained, resulting in a variable number of replicates per maize accession (Figure 2 and Supplementary Table S1).

**Figure 3:**
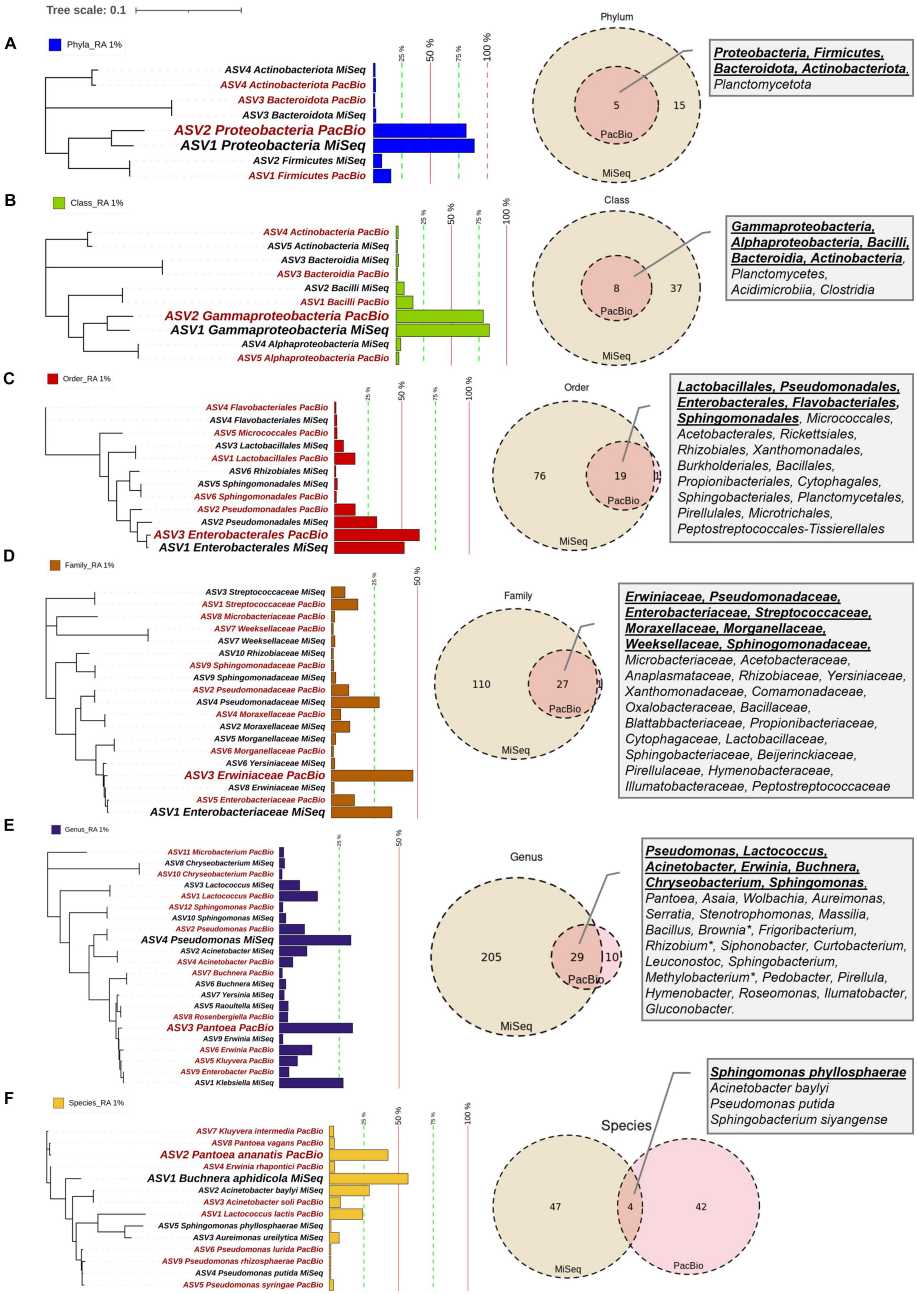
Maize pollen microbiomes identified by FL-PacBio and V4-MiSeq sequencing at multiple taxonomic levels. The taxonomic levels shown are: **(A)** phylum, **(B)** class, **(C)** order, **(D)** family, **(E)** genus, and **(F)** species. At each taxonomic level (row), the panels are, from L-R: the phylogenetic tree, relative abundance (RA) as a bar chart, Venn diagram comparison of results from FL-PacBio and V4-MiSeq sequencing, and textboxes displaying shared taxa from both sequencing platforms. Each phylogenetic tree represents aggregated, merged taxa [relative abundance (RA) threshold ≥1%] that are annotated with a corresponding bar chart showing the relative abundance at that taxonomic level based on FL-PacBio and V4-MiSeq sequencing. The most dominant taxa at each taxonomic level from each sequencing platform are displayed in a weighted font. Taxa displayed in red font denote those identified by FL-PacBio, whereas the black font denotes those identified by V4-MiSeq. The Venn-diagrams represent the counts of identified taxa from both sequencing platforms at each taxonomic level when the entire microbiome is included. Textboxes display the shared taxa from both sequencing platforms where underlined taxa denote the shared taxa at RA ≥ 1%.

**Figure 4:**
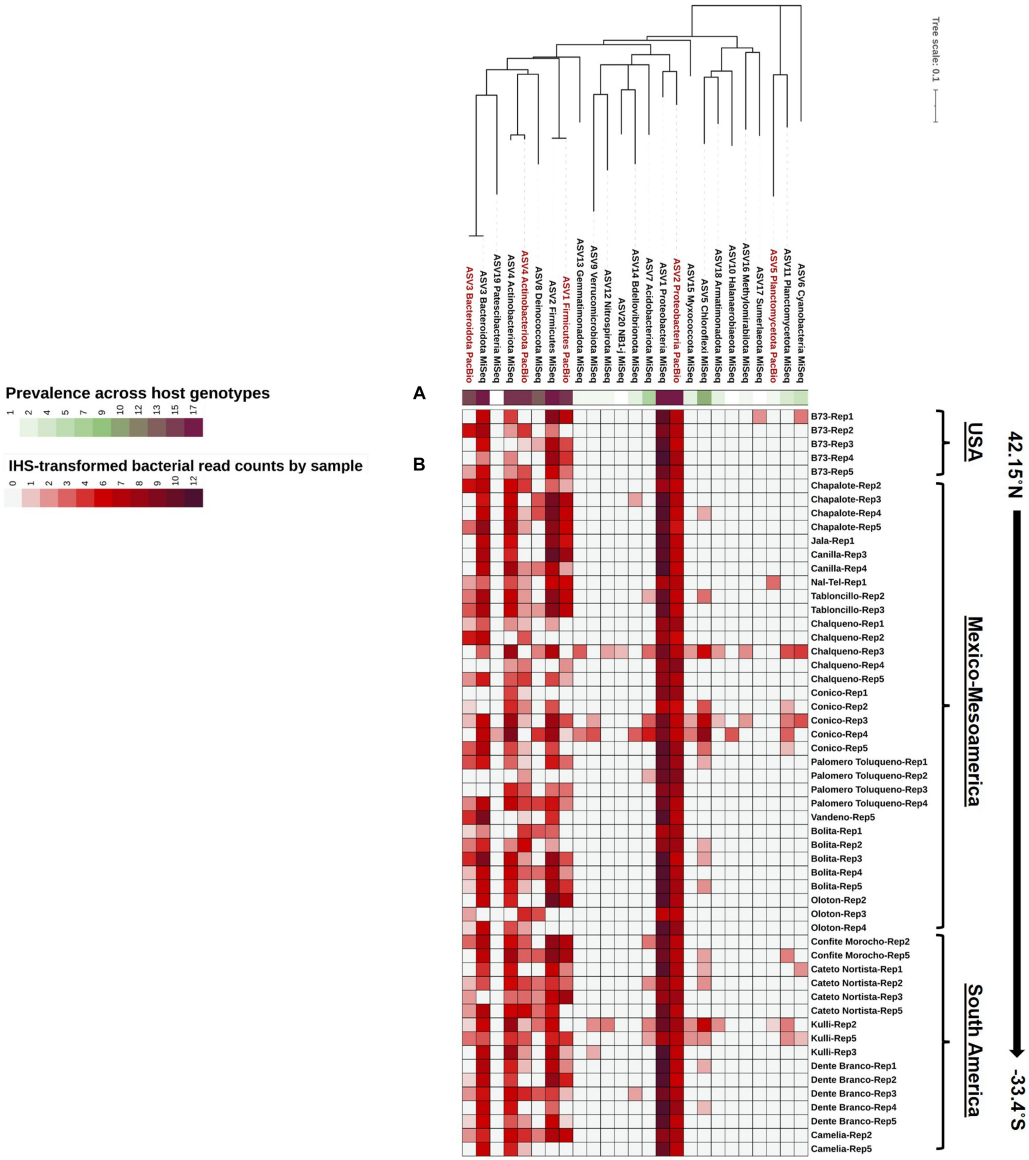
A phylogenetic tree of the Pan-American maize pollen microbiomes identified and merged from FL-PacBio and V4-MiSeq sequencing platforms and aggregated to the phylum level. The phylogenetic tree is annotated with **(A)** a single-colored bar chart showing the prevalence of phyla across maize accessions and **(B)** a vertical heatmap of IHS-transformed read counts for each phylum. Pollen samples are grouped by maize accession and replicate number, arranged from North to South according to the latitude at which they originated.

**Figure 5:**
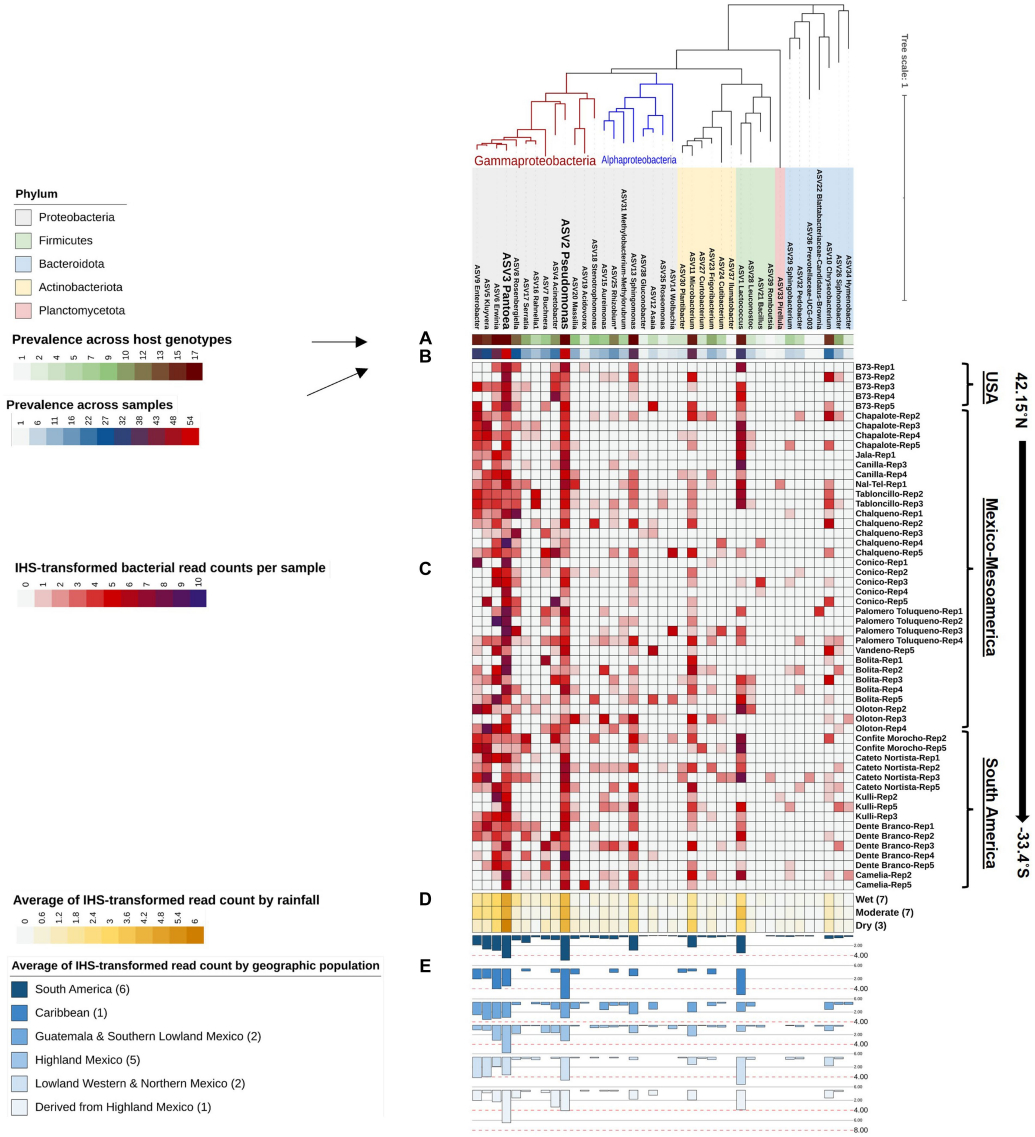
A phylogenetic tree of all bacterial genera composing the Pan-American maize pollen microbiome identified by FL-PacBio sequencing. The phylogenetic tree is annotated as follows: **(A)** a single-colored barchart showing the prevalence of genera across maize accessions; **(B)** a single-colored barchart showing the prevalence of each bacterial genus across pollen samples; **(C)** a vertical heatmap of IHS-transformed read counts for each genus. Pollen samples are grouped by maize accession and replicate number, arranged from north to south according to the latitude at which they originated; **(D)** a heatmap of IHS-transformed bacterial read counts at the genus level across maize accessions clustered by rainfall; **(E)** a multi-bar chart displaying the average of IHS-transformed bacterial read counts for maize accessions clustered by their geographical population based on Matsuoka et al. (2002). In the geographic population legend, the number in brackets denotes the number of maize accessions in that population. ASV2_*Pseudomonas*, and ASV3_*Pantoea* are displayed in weighted font to represent the most dominant and prevalent genera in the pollen microbiome across all Pan-American maize accessions.

**Figure 6:**
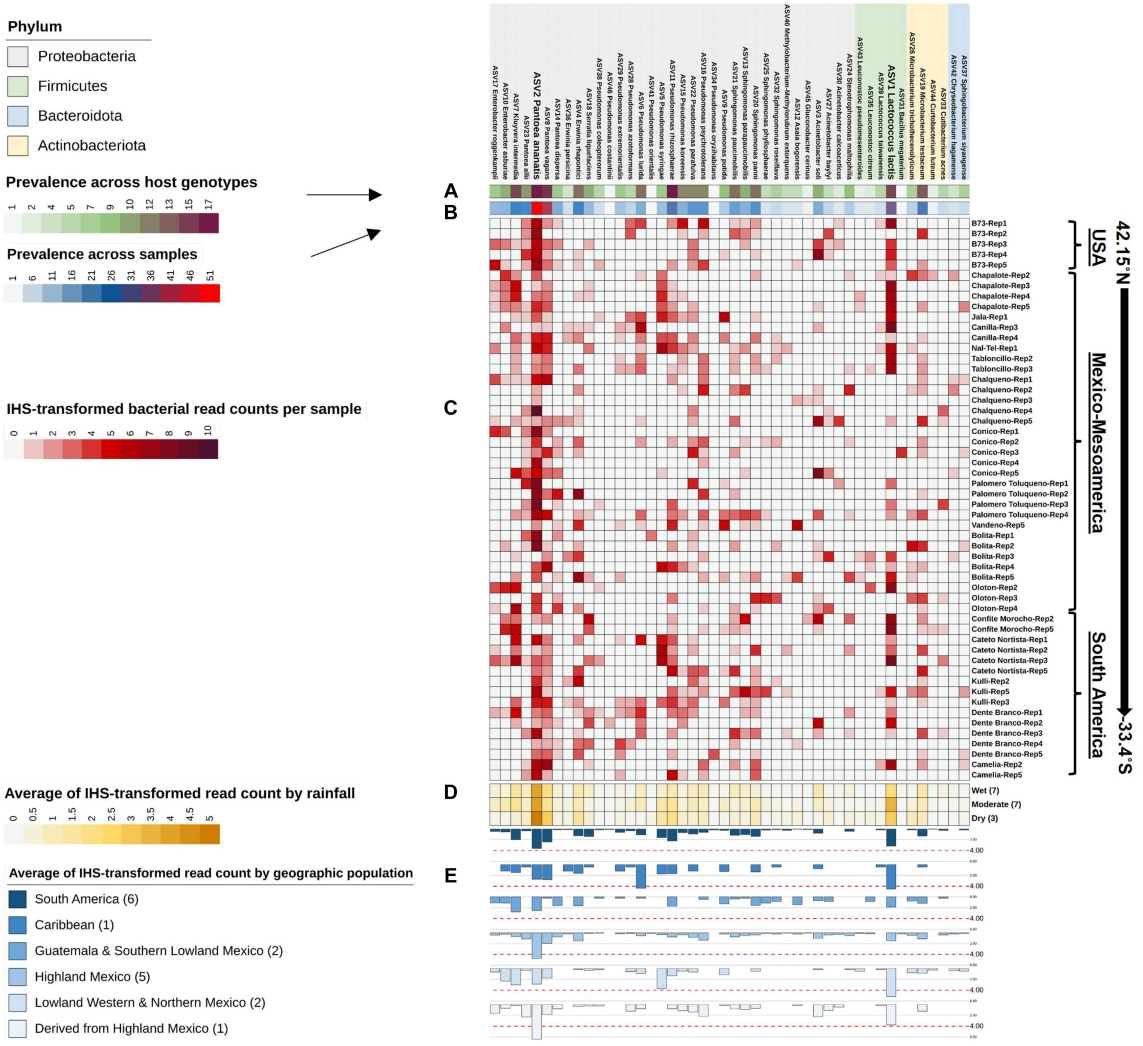
Taxonomic clustering of all bacterial species composing the Pan-American maize pollen microbiome identified by FL-PacBio sequencing. The bacterial species are annotated as follows: **(A)** a single-colored barchart showing the prevalence of species across maize accessions; **(B)** a single-colored barchart showing the prevalence of each bacterial bacteria across pollen samples; **(C)** a vertical heatmap of IHS-transformed read counts for each species. Pollen samples are grouped by maize accession and replicate number, arranged from north to south according to the latitude at which they originated; **(D)** a heatmap of IHS-transformed bacterial read counts at the species level across maize accessions clustered by rainfall; **(E)** a multi-bar chart displaying the average of IHS-transformed bacterial read counts for maize accessions clustered by their geographical population based on Matsuoka et al. (2002). In the geographic population legend, the number in brackets denotes the number of maize accessions in that population. ASV1_*Lactococcus lactis*, and ASV2_*Pantoea ananatis* are displayed in weighted font to represent the most dominant and prevalent bacterial species in the pollen microbiome across all Pan-American maize accessions.

DNA samples were split, with half used for paired-end 16S hypervariable V4 region sequencing (V4-MiSeq) (submitted to NCBI database under PRJNA766023) and half used for full-length 16S rRNA gene sequencing (FL-PacBio) (submitted to NCBI database under PRJNA773232) (Figure 2, Supplementary Table S1). After filtering organelle reads, the pollen microbiome from V4-MiSeq was taxonomically classified into 99.93% bacterial taxa and 0.07% Archaeal taxa (Supplementary Table S4), whereas the entire sequences generated from FL-PacBio exclusively belonged to the kingdom Bacteria. When excluding archaeal taxa, V4-MiSeq generated 1,432 taxa accounting for 1,598,857 reads, while FL-PacBio generated 765 taxa accounting for 66,327 reads (Supplementary Table S4). V4-MiSeq data identified 20 phyla, 234 genera and 51 species belonging to the Pan-American maize pollen microbiome, when combined (Figure 3, Supplementary Figure S1, and Supplementary Table S4). However, the longer-read FL-PacBio data identified only 5 phyla (Figures 3, 4) and 39 genera (Figure 5) but 46 species (Figure 6).

### Discrepancies between 16S rRNA gene V4-MiSeq and FL-PacBio results

**Figure 7:**
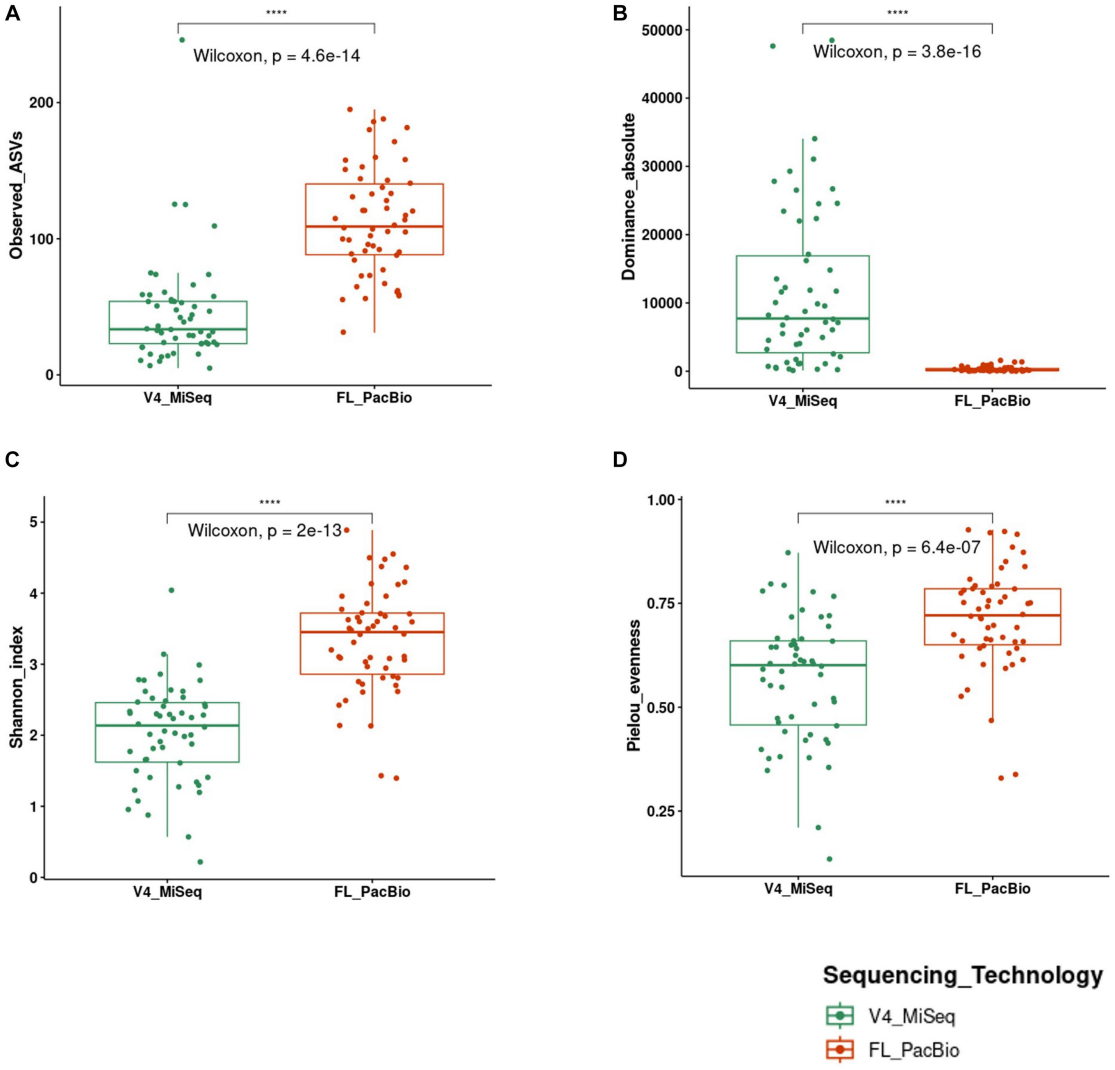
Statistical comparison of alpha diversity scores calculated on 16S raw reads generated from FL-PacBio and V4-MiSeq sequencing using the Mann–Whitney U test for independent samples (unpaired samples). Statistical comparisons were displayed using boxplots that represent the following alpha diversity metrics: **(A)** Observed ASVs (richness), **(B)** Dominance absolute (read count), **(C)** Shannon index (diversity), **(D)** Pielou evenness index (evenness). Significance levels: *p* < 0.05 (*), *p* < 0.01 (**), *p* < 0.001 (***), *p* < 0.0001 (****).

To assess the impact of 16S rRNA gene targeted amplicon region and sequencing technology used, alpha diversity metrics were calculated (richness, diversity, and evenness) to compare the pollen microbiome identified from V4-MiSeq versus FL-PacBio. Mann–Whitney U tests were conducted on calculated alpha metrics from each sequencing technology at the taxa level (Figure 7). The two sequencing technologies were significantly different for every metric (Figure 7). Pollen samples sequenced by V4-MiSeq showed greater richness (Observed ASVs, *p* < 0.0001) and read count (Absolute-dominance, p < 0.0001) compared to the calculated alpha measures from FL-PacBio. However, FL-PacBio showed greater diversity (Shannon diversity index, *p* < 0.0001) and evenness (Pielou, *p* < 0.0001) at the taxa level compared to the calculated metrics from V4-MiSeq.

At the phyla, class, order and family levels, V4-MiSeq predicted much greater overall diversity than FL-PacBio (Figures 3A−D, 4). The finest taxonomic level in which FL-PacBio predictions were consistently predicted by V4-MiSeq was at the order level. There were considerable taxonomic differences between the two technologies at the family, genus and species levels (Figures 3E,F and Supplementary Figures S1−S5).

The limitations of V4-MiSeq at the genus and species levels were demonstrated by the observation that FL-PacBio was internally consistent across taxonomic levels, whereas V4-MiSeq was not. FL-PacBio sequencing predicted that the pollen microbiome was dominated by the family *Erwiniaceae* (relative abundance (RA), 47.35%), the genus *Pantoea* (30.58%) and the species *Pantoea ananatis* (42.45%); the percentages were calculated within each taxonomic level. *Pantoea* lies within *Erwiniaceae*, and clearly *P. ananatis* is a member of the genus *Pantoea* (Figures 3D−F). By contrast, the dominant taxa obtained from V4-MiSeq was *Enterobacteriaceae* (35.15%), *Pseudomonas* (29.9%), and *Buchnera aphidicola* (56.84%) at the family, genus and species levels, respectively. However, *Pseudomonas* does not belong to the *Enterobacteriaceae,* and *Buchnera aphidicola* neither belongs to the genus *Pseudomonas* or the family *Enterobacteriaceae* (Figures 3D−F). Consistent with these observations, whereas FL-PacBio could assign 95.38% of reads to the genus level, and 58.39% to the species level, V4-MiSeq assignments were limited to 75.62 and 3.58%, respectively (Supplementary Table S4).

The limitation of V4-MiSeq at the genus level was further demonstrated by examining the dominant genus predicted by each method. V4-MiSeq identified *Klebsiella* as the most prevalent genus across samples (50/54 samples) with an average RA >25%, but it was not identified by FL-PacBio (Table 2). Instead, the V4-*Klebsiella* sequence 100% matched the full-length 16S rRNA gene *Enterobacter* and *Erwinia* sequences (Supplementary Figure S6) which combined constituted >15% RA in the PacBio data; *Klebsiella* and *Enterobacter* belong to the family *Enterobacteriaceae*, while *Erwinia* belongs to the family *Erwiniaceae*. The result therefore shows that the short V4 sequence was not able to accurately discriminate at either the genus or family level. Similarly worrisome, using FL-PacBio, *Pantoea* was the most prevalent genus (54/54 samples), with an average RA of >25%, but V4-MiSeq identified it as <1% RA (Table 2). We found no exact sequence matches for the V4 *Pantoea* portion of the full-length 16S rRNA gene sequence (Supplementary Figure S6). These results are consistent with the prior literature which showed that V4-MiSeq mis-identifies *Enterobacteriaceae* family members (which would include *Klebsiella*) and over-estimates their prevalence (Greay et al., 2019), and furthermore, 16S V4 primers bias against *Pantoea* [along with *Microbacterium* (underrepresented using V3-V4 sequencing)] (Abellan-Schneyder et al., 2021; Palkova et al., 2021). To cross-check the microbiome results, we examined the full-length 16S rRNA gene sequences of cultured isolates from the pollen of Pan-American accessions, of which 12 were in this study (same samples). This parallel study will be reported in a later publication. Of 298 isolates, zero were identified as *Klebsiella,* whereas 42/298 were identified as *Pantoea* (Shrestha, 2023), consistent with FL-PacBio results and contrary to the V4-MiSeq data.

**Table 2:** Percentage average relative abundance (RA) of genera identified from FL-PacBio sequencing (all genera) and V4-MiSeq sequencing (only with RA ≥ 0.1)^1^.

Family	Genus	FL-PacBio			V4-MiSeq		
		Sample count	Taxa count	Average% of RA (% min RA– %max RA)	Sample count	Taxa count	Average% of RA (% min RA– %max RA)
*Enterobacteriaceae*	*Klebsiella*	0	−	−	50	7	25.71 (0 − 93.58)
*Enterobacteriaceae*	*Enterobacter*	34	14	3.98 (0 − 49.20)	0	−	−
*Enterobacteriaceae*	*Kluyvera*	30	41	6.08 (0 - 88.52)	0	−	−
*Enterobacteriaceae*	*Citrobacter*	0	−	−	2	3	0.103 (0 − 4.31)
*Enterobacteriaceae*	*Raoultella*	0	−	−	32	8	5.65 (0 − 74.39)
*Erwiniaceae*	*Pantoea*	54	215	25.62 (0.062 − 99.17)	2	2	0.39 (0 - 20.73)
*Erwiniaceae*	*Erwinia*	41	75	11.47 (0 − 92.96)	10	4	0.70 (0 - 15.96)
*Erwiniaceae*	*Rosenbergiella*	27	-	3.69 (0 − 95.63)	0	−	−
*Xanthomonadaceae*	*Stenotrophomonas*	10	2	0.32 (0 − 12.76)	28	12	0.64 (0 - 10.39)
*Morganellaceae*	*Buchnera*	15	9	1.52 (0 − 32.57)	34	7	6.28 (0 - 94.65)
*Yersiniaceae*	*Yersinia*	0	−	−	24	7	1.37 (0 - 16.29)
*Yersiniaceae*	*Rahnella1*	10	3	0.48 (0 − 11.68)	0	−	−
*Yersiniaceae*	*Serratia*	14	3	0.39 (0 − 11.16)	RA < 0.1	2	−
*Pseudomonadaceae*	*Pseudomonas*	53	80	14.21 (0 − 96.32)	52	80	24.47 (0 - 76.41)
*Moraxellaceae*	*Acinetobacter*	22	33	5.34 (0 − 80.18)	45	35	8.61 (0 - 91.27)
*Oxalobacteraceae*	*Massilia*	13	4	0.55 (0 − 19.96)	31	18	0.70 (0 − 12.85)
*Oxalobacteraceae*	*Duganella*	0	−	−	12	8	0.28 (0 − 8.11)
*Comamonadaceae*	*Xylophilus*	0	−	−	24	14	0.41 (0 - 11.60)
*Comamonadaceae*	*Acidovorax*	3	1	−	−	−	−
*Acetobacteraceae*	*Asaia*	6	3	0.72 (0 − 28.09)	14	10	0.55 (0 − 15.48)
*Acetobacteraceae*	*Roseomonas*	3	1	0.01 (0 − 0.36)	RA < 0.1	13	−
*Acetobacteraceae*	*Gluconobacter*	2	1	0.006 (0 − 0.17)	RA < 0.1	4	−
*Sphingomonadaceae*	*Sphingomonas*	39	28	2.72 (0 − 33.17)	52	53	6.83 (0 − 53.28)
*Rhizobiaceae*	*Rhizobium* *	17	4	0.15 (0 − 2.54)	6	6	0.08 (0 − 2.14)
*Rhizobiaceae*	*Neorhizobium*	0	−	−	39	11	0.89 (0 − 4.76)
*Rhizobiaceae*	*Aureimonas*	12	3	0.50 (0 − 21.19)	43	15	1.86 (0 − 28.28)
*Beijerinckiaceae*	*Methylobacterium* *	9	2	0.04 (0 − 1.23)	40	39	0.38 (0 − 5.95)
*Caulobacteraceae*	*Brevundimonas*	0	−	−	31	12	0.17 (0 − 1.95)
*Anaplasmataceae*	*Wolbachia*	4	2	0.40 (0 − 15.64)	22	12	0.41 (0 − 9.16)
*Microbacteriaceae*	*Curtobacterium*	4	3	0.03 (0 − 1.12)	26	10	0.92 (0 − 14.69)
*Microbacteriaceae*	*Microbacterium*	40	30	2.80 (0 − 32.95)	0	−	−
*Microbacteriaceae*	*Herbiconiux*	0	−	−	27	8	0.43 (0 - 5.17)
*Microbacteriaceae*	*Frigoribacterium*	12	3	0.14 (0 − 1.85)	RA < 0.1	1	−
*Microbacteriaceae*	*Plantibacter*	5	1	0.03 (0 − 0.65)	0	−	−
*Geodermatophilaceae*	*Blastococcus*	0	−	−	12	16	0.89 (0 − 33.43)
*Nocardioidaceae*	*Nocardioides*	0	−	−	17	40	0.85 (0 − 26.96)
*Propionibacteriaceae*	*Cutibacterium*	7	2	0.04 (0 − 1.06)	0	−	−
*Weeksellaceae*	*Chryseobacterium*	24	10	2.56 (0 − 38.28)	44	37	2.65 (0-25.03)
*Sphingobacteriaceae*	*Sphingobacterium*	7	1	0.05 (0 − 1.26)	25	21	0.25 (0 − 4.71)
*Sphingobacteriaceae*	*Pedobacter*	6	2	0.04 (0 − 0.65)	30	13	0.28 (0 − 4.26)
*Cytophagaceae*	*Siphonobacter*	11	3	0.09 (0 − 1.47)	36	15	0.30 (0 − 4.17)
*Hymenobacteraceae*	*Hymenobacter*	3	2	0.02 (0 − 0.62)	RA < 0.1	16	−
*Streptococcaceae*	*Lactococcus*	35	115	15.45 (0 − 81.72)	40	11	6.50 (0-46.20)
*Lactobacillaceae*	*Leuconostoc*	7	3	0.05 (0 − 1.19)	17	13	0.18 (0 − 2.72)
*Paenibacillaceae*	*Paenibacillus*	0	−	−	17	14	0.22 (0 − 4.58)
*Bacillaceae*	*Bacillus*	3	2	0.17 (0 − 8.96)	RA < 0.1	6	−
*Pirellulaceae*	*Pirellula*	2	1	0.01 (0 − 0.65)	RA < 0.1	4	−

1 Genera that were present only in one sample from FL-PacBio and either having RA < 0.1 (Ilumatobacter, Candidatus Brownia) or absent from V4-MiSeq (Romboutsia, Prevotellaceae UCG-003) were removed.

*These names were shortened. The complete names are: Rhizobium*: Allorhizobium-Neorhizobium-Pararhizobium-Rhizobium, Methylobacterium*: Methylobacterium-Methylorubrum.

Furthermore, despite having a magnitude more read counts, V4-MiSeq missed several dominant genera (>1% RA) identified by FL-PacBio, specifically *Enterobacter*, *Kluyvera*, *Rosenbergiella*, and *Microbacterium* (Table 2).

In several but not all instances, FL-PacBio sequencing could reveal greater intra-genus level diversity than V4-MiSeq. For example, FL-PacBio identified 215 unique taxa within *Pantoea*, 115 taxa within *Lactococcus*, and 75 within *Erwinia*, whereas V4-MiSeq only identified 2, 11 and 4 taxa, respectively (Table 2). However, both primer sets identified similar levels of diversity within *Pseudomonas, Acinetobacter*, and Sphingomonas.

Taken together, the simplest explanation is that a longer amplicon length is required for finer taxonomic resolution. However, the challenge here was that FL-PacBio had few reads which may have missed rare taxa. Balancing these two issues, in this study we defer to V4-MiSeq to be comprehensive at higher taxonomic levels (phyla, class, order), but then to FL-PacBio for accuracy at the family-genus-species-ASV levels. Accordingly, at finer taxonomic levels, V4-MiSeq is presented only as Supplementary files (Supplementary Figures S1, S2).

### Identification of the core Pan-American maize pollen microbiome

We asked if any taxa were highly conserved (prevalent) across pollen from Pan-American maize accessions and samples, within the study limitations. At the phyla level, *Proteobacteria* was the most dominant (RA ≥ 1%) and prevalent phylum (present in 54/54 samples, spanning all host accessions) from both V4-MiSeq and FL-PacBio, followed by *Firmicutes*, *Bacteriodota*, and *Actinobacteriota*, especially when deferring to V4-MiSeq (Figures 3, 4). There were 16 orders that were prevalent across >70% of maize accessions and 50% of samples using V4-MiSeq; in rank order of prevalence, these were: *Enterobacterales*, *Pseudomonadales*, *Sphingomonadales*, *Cytophagales*, *Flavobacteriales*, *Rhizobiales*, *Burkholderiales*, *Lactobacillales*, *Sphringobacteriales, Micrococcales, Caulobacterales*, *Xanthomonadales, Acetobacterales*, *Rickettsiales, Propionibacteriales, Azospirillales* (Figure 3C and Supplementary Figure S4). Most were also observed by FL-PacBio (Figure 3C and Supplementary Figure S4).

**Figure 8:**
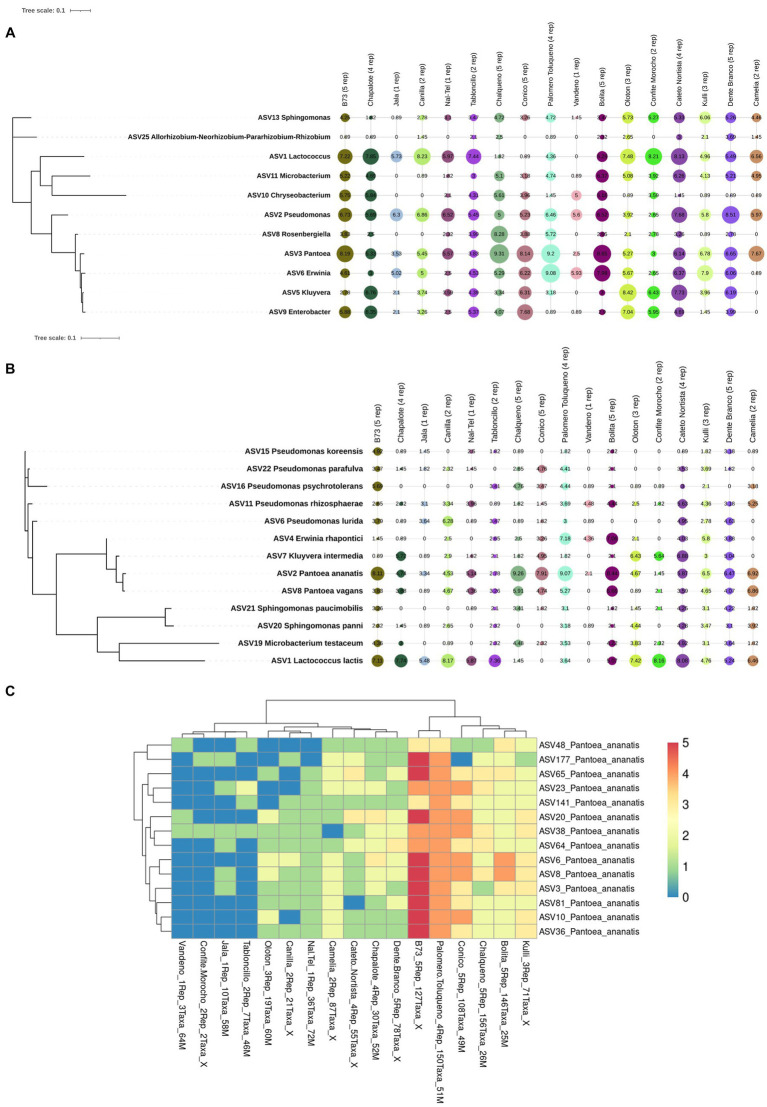
Phylogenetic trees and a hierarchical clustering heatmap of the Pan-American maize pollen core microbiome generated from FL-PacBio sequencing. Shown is the core microbiome at the **(A)** genus level, **(B)** species level and **(C)** taxa level within the species, *Pantoea ananatis* identified at a prevalence threshold ≥50% across samples and 70% of host accessions. In **(A,B)**, the geometric shapes represent IHS-transformed read counts and their sizes are proportional to the calculated values. In **(C)**, the color scale bar (1−5) represents the count of pollen sample replicates within each maize accession. Along the *x*-axis, the maize accession name is noted, followed by the number of replicates, then the count of *P. ananatis* taxa, followed by a code (a number followed by M) where the number represents the placement of the maize accession on the phylogenetic tree of Matsuoka et al. (2002) constructed based on microsatellite data for the number, see supporting information, Figure 4B and Table 1 in Matsuoka et al. (2002). The X means the maize accession was not included in the Matsuoka study.

At the genus level, of the 39 unique pollen-associated bacterial genera identified by FL-PacBio (Figure 5), 11 genera were found to be prevalent across maize accessions (at a threshold of >50% of samples and 70% of host accessions) (Figure 8A). These constitute the Pan-American core pollen microbiome under the conditions of this study. Four genera were observed in 17/17 maize accessions: *Pantoea, Pseudomonas, Erwinia* and *Sphingomonas*. *Lactococcus, Enterobacter, Microbacterium* were found in 16/17 host accessions. *Kluyvera* and *Chryseobacterium* were present in 15 host accessions, *Rosenbergiella* in 13 accessions and *Rhizobium* in 12 accessions. In terms of prevalence across pollen samples, 5 genera were identified at a prevalence threshold of 40%; these were *Pantoea* (present in 54/54 samples), *Pseudomonas* (53/54), *Erwinia* (41/54), *Lactococcus* (35/54), and *Kluyvera* (30/54). Overall, *Pantoea* and *Pseudomonas* dominated the Pan-American maize pollen microbiome (Figures 5, 8 and Table 2).

The corresponding V4-MiSeq data at the genus level is located in Supplementary Figures S1, S2 with the genera shared by both technologies in Supplementary Figure S3 and the core V4-MiSeq microbiome shown in Supplementary Figure S7.

At the species level, of the 46 pollen-associated species identified by FL-PacBio (Figure 6), 13 constituted the Pan-American core microbiome, defined as being present in >50% of pollen samples and > 70% of host accessions (Figure 8B). Of these, *Pantoea ananatis* and *Pseudomonas rhizosphaerae* were present in all 17 maize accessions (Figure 8C and Supplementary Figure S8), followed by *Lactococcus lactis* in 15/17 accessions. At the sample level, the most prevalent species (at 50% threshold), in rank order were: *Pantoea ananatis* (present in 51/54 samples)*, Pantoea vagans* (40/54)*, Lactococcus lactis* (33/54), *Pseudomonas rhizosphaerae* (29/54), and *Microbacterium testaceum* (28/54).

### Taxa-level conservation and diversity within The core Pan-American maize pollen microbiome

In terms of diversity, of the 765 taxa identified by FL-PacBio, four genera belonging to the core microbiome accounted for 63% of the taxa-level diversity (485/765) across the Pan-American pollen microbiome, with 215 taxa (28.1%) found within the genus *Pantoea* (4 species), 115 taxa (15.0%) within *Lactococcus* (2 species), 80 taxa (10.5%) within *Pseudomonas* (13 species), and 75 taxa (9.8%) within *Erwinia* (2 species).

Of the 215 *Pantoea* taxa, 180 belonged to a single species, *P. ananatis*. Hence, *P. ananatis* was not only the most conserved species across the Pan-American maize pollen microbiome, but also the most diverse. From this diversity, 14 taxa were conserved in 70% of maize accessions surveyed (Figure 8C), showing that specific intra-species taxa within *P. ananatis* are fundamental to the Pan-American core pollen microbiome; lack of conservation in specific maize accessions was associated with low host replicate number.

**Figure 9:**
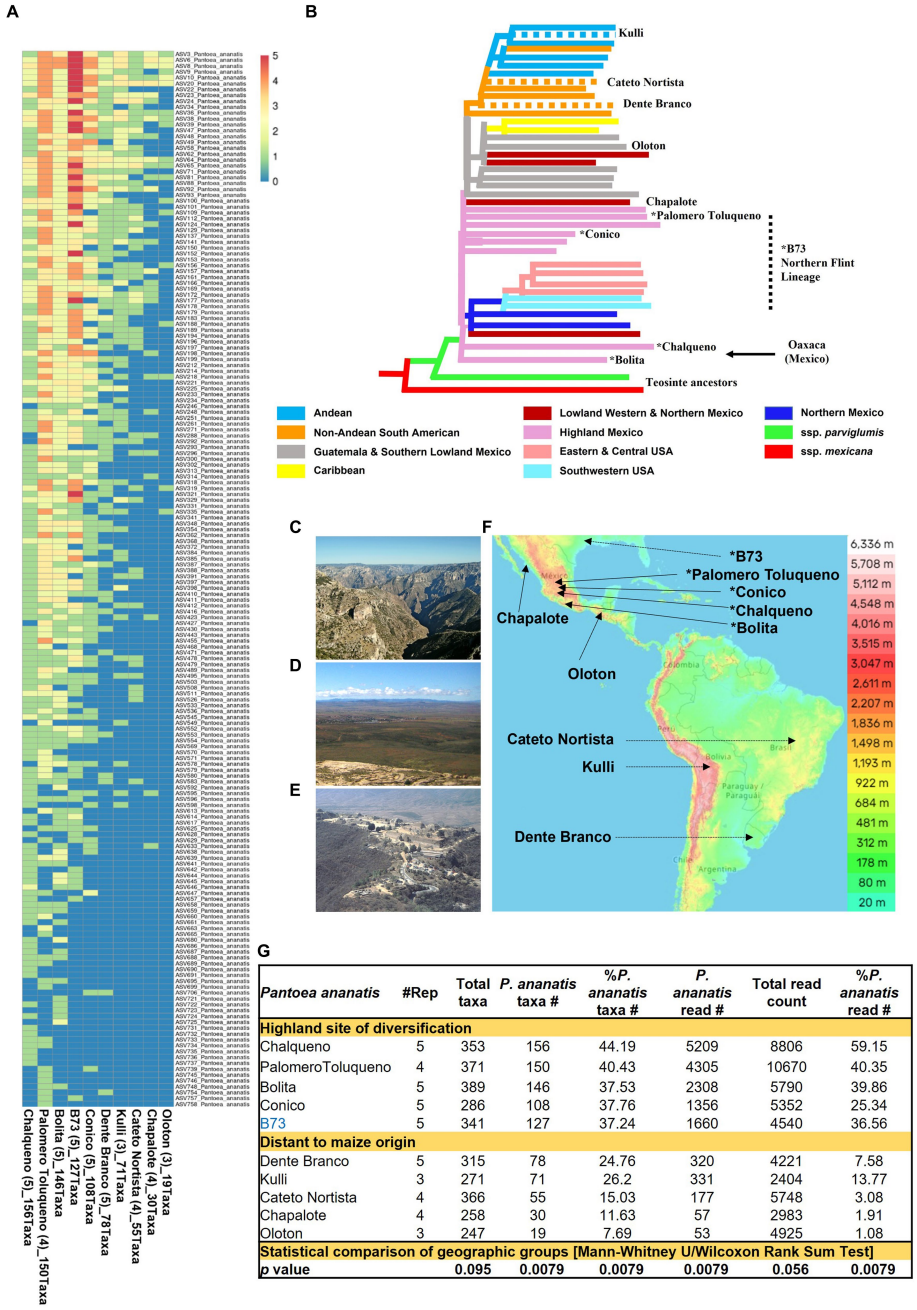
Diversity and prevalence of *Pantoea ananatis* taxa from the pollen microbiome across maize accessions. **(A)** Hierarchical clustering heatmap of 180 *P. ananatis* taxa identified across maize accessions (those with ≥3 replicates). **(B)** Genetic relatedness of the maize accessions in this study, adapted from Matsuoka et al. (2002). The dotted branches indicate these accessions were not in Matsuoka et al. (2002) but were placed here based on their immediate geographic origin or reported long-term derivation in the case of B73 (Swarts et al., 2017). The asterisk indicates that this accession originated or was derived from the Mexican Highlands. The arrow indicates the region of maize diversification (Oaxaca, Mexican) in the Mexican Highlands. **(C)** Picture to illustrate that the route from Western Highland Mexico (e.g., Palomero Toluqueno origin) to the Lowlands (e.g., Chapalote origin) includes multiple mountain ranges and deep canyons, presumably creating geographic isolation between these regions in ancient times. Shown is a picture from Copper Canyon (Barranca del Cobre) in Chihuahua, Mexico. **(D)** A picture of the Highland Mexican plateau (Zacatecas, Zacatecas, Mexico) which may have permitted sharing of maize accessions across this region in ancient times. **(E)** A picture of Oaxaca, Mexico, the origin of ancient maize diversification in the Mexican Highlands. Shown is a picture of the ancient Oaxacan city, Monte Albán. **(F)** A topographic map of the Americas showing the ancestral origins of the maize accessions used in the *P. ananatis* taxa analysis (i.e., those >3 replicates). **(G)** A summary table illustrating the contribution of *P. ananatis* to the pollen microbiome for maize accessions with ≥3 replicates categorized into two groups according to their geographic distance from the site of maize diversification. The bottom row indicates whether the mean of the two groups for each column is statistically different (*p* value). Picture **(C)** was taken by Jens Uhlenbrock from Wikimedia Commons (public domain). Picture **(D)** is by Katie Yaeger Rotramel and made available under the license (CC BY-NC-SA 2.0). Picture **(E)** is by Mannheim Reinhard Jahn from Wikimedia Commons and made available under the license (CC BY-SA 2.0 de). The map in panel **(F)** is adapted from topographic-map.com, with the data from TessaDEM which is licensed under the Open Database License (ODbL) v1.0, and from Open Street Map under the license (CC BY-SA 2.0).

Notably, the diversity within *P. ananatis* ranged nearly 10-fold among the 10 maize accessions with ≥3 replicates, from 156 to 19 taxa (Figure 9A). This diversity was geographically non-random (Figures 9B−D): based on the phylogenetic classification of Matsuoka (Matsuoka et al., 2002), the accessions with the highest diversity within *P. ananatis* (Chalqueno, Palomero toluqueno, Bolita, Conico) originated near the center of diversity of domesticated maize from central Highland Mexico and included inbred B73 which has its primitive ancestry from that region (clustered with Palomero Toluqueno) through its Northern flint lineage (da Fonseca et al., 2015). By contrast, the landraces with the lowest *P. ananatis* diversity were those that dispersed to the lowlands, either north (Chapalote, Lowland Northern Mexico) or south (Oloton, Guatemala) eventually into South America [Dente Branco (Uruguay, Non-Andean South America), Kulli (Bolivia, Andean-South America), Cateto Nortista (Brazil, non-Andean South America)] (Figures 9A−F). These trends were also observed with the percentage read count of *P. ananatis* (Figure 9G and Supplementary Figure S9). The decline in *P. ananatis* sub-species diversity outside of the Mexican Highlands was not consistently due to a general decline in bacterial diversity or total read counts (Figure 9G). *P. ananatis* was not replaced by other *Pantoea* species (Supplementary Table S5). Rather in these non-Highland accessions, *P. ananatis* diversity and read count in maize pollen were partially replaced by *Lactococcus*, especially *L. lactis* in Cateto Nortista, Chapalote and Oloton (Figure 9G and Supplementary Table S5), and by *Erwinia* in Kulli (Supplementary Table S5). Pollen of the maize inbred B73 also had a moderate diversity of *L. lactis* (Supplementary Figure S9 and Supplementary Table S5).

## Discussion

### Pollen as a vector for heritable diversification of plant genetic material

Here we undertook 2,500+ pollen bag collections from the U.S. inbred B73 and 16 primitive and traditional Pan-American landraces selected by indigenous peoples from across the Americas, to provide the first report of the maize pollen microbiome, pollen microbiome diversity within a host species, and critically, pollen microbiome profiling based on full-length 16S rRNA gene sequencing (FL-16S). The results reveal that maize pollen has high intra-species diversity within its core microbiome which varies between host accessions. Specifically, the FL-16S results show that the pollen of Pan-American maize collectively possess bacteria spanning 39 genera, 46 species and 765 taxa. Of these taxa, 63% belonged to only 4 genera: 215 taxa (28.1%) within *Pantoea*, 115 taxa (15.0%) within *Lactococcus,* 80 taxa (10.5%) within *Pseudomonas* and 75 taxa (9.8%) within *Erwinia*. A prior study from our group showed that *Pantoea* was the most diverse seed-associated genus, and among the most prevalent, across wild *Zea* relatives and ancient Pan-American maize landraces, across two generations (Johnston-Monje and Raizada, 2011). *Pantoea* was also shown to be present in multiple generations of rice seeds (Kim et al., 2022).

Furthermore, of the 215 *Pantoea* taxa in maize pollen, 180 taxa (24% of all taxa) belonged to a single species, *P. ananatis.* This intra-species diversity was missed using the standard 16S V4-MiSeq primers but was only identified using full-length 16S rRNA gene primers (PacBio), due to primer bias and read length differences (Abellan-Schneyder et al., 2021; Palkova et al., 2021). Fourteen *P. ananatis* taxa were identified in at least 70% of maize accessions, and in almost all Mexican Highland replicates (Figure 8C); these may be the retained founders of the maize pollen core microbiome, suggestive of an intimate and ancient co-evolutionary relationship (Zilber-Rosenberg and Rosenberg, 2021). Indeed*, P. ananatis* is found in maize seeds in the Americas (Rijavec et al., 2007; Johnston-Monje and Raizada, 2011) and is perhaps the only bacterial species convincingly demonstrated, using bacterial whole genome sequencing, to be inherited (vertically transmitted) in plants, specifically in rice (Zhang et al., 2022), a relative of maize – though whether it is paternally or maternally inherited is unknown.

Our findings suggest that the diversity within *P. ananatis* ranges nearly 10-fold among ten maize accessions (those with ≥3 replicates) despite plants being grown side by side in a common field. The highest diversity occurred in landraces originating near the center of domestication and diversification of maize in the Mexican Highlands (Piperno and Flannery, 2001; Matsuoka et al., 2002; Piperno et al., 2009; Kistler et al., 2018) with less diversity observed in landraces originating from lowland Mexico, Central America and South America (Figure 9A). The archeological record and genomic evidence suggest that maize diversified in the Highlands of Mexico before spreading to the lowlands (Vallebueno-Estrada et al., 2016). Therefore, one possible explanation is that diversity within *P. ananatis* in pollen was lost as domesticated maize migrated away from its geographic center of diversification thousands of years ago. If true, then similar to plant intra-species diversity being highest near the domestication origin of a crop (Smith, 1969), we hypothesize that bacterial intra-species diversity within a pollen microbiome is also greatest there – but specifically for a microbe that is heritable.

However, all evolutionary interpretations in this study should be viewed cautiously, only as hypotheses since all plants were grown for a single season in a common Canadian field which may have served as a source of some microbes. Nevertheless, the non-random diversity of *P. ananatis* amongst maize accessions combined with the findings for its vertical transmission in rice (Zhang et al., 2022) suggest that at least this species is less influenced by the environment. However, more evidence is required, including testing for vertical transmission, collecting ancient pollen samples from archeological sites, and collecting pollen in their native habitats which could serve as sources of microbes.

### Was there inadvertent farmer and breeder selection of *Pantoea ananatis* in the maize pollen microbiome?

Approximately 40% of all pollen microbiome taxa across maize landraces that originated near the origin of maize domestication at the site of diversification in highland Mexico were consistently *P. ananatis* but reduced outside of this region (Figures 9C−G). The simplest interpretation is that pollen-associated *P. ananatis* was present in the earliest domesticated maize and thus had the longest time to mutate and hence diversify. *P. ananatis* diversification may have been selected by cycles of climate change that may have impacted the morphology, physiology and biochemistry of host maize plants in this region. Indeed, ancient human agricultural societies in the Americas faced dramatic climate change (Degroot et al., 2022). For example, there is historical evidence of intermittent droughts in Oaxaca (Mexico), the center of maize Highland diversity (Matsuoka et al., 2002), between 1,500 and 1800 (Mendoza et al., 2006). In terms of why common *P. ananatis* taxa were shared across diverse Mexican Highland sites, evidence suggests there was considerable gene flow among maize landraces within this region (Kistler et al., 2020). Seed trading by farmers would have been facilitated by the plateau topography of the Mexican Highlands (Figure 9F). However, it is also interesting to speculate that pollen dispersal itself may have maintained pollen *P. ananatis* diversity in the Highlands, since maize is a wind-pollinated crop and its pollen can travel up to 32 km (Luna et al., 2001). From the Highlands, farmers likely migrated small amounts of seed to new locations, which may have created bottlenecks in *P. ananatis* diversity, i.e., resulting in a founder effect. The journey out of the Mexican Highlands involves multiple mountain ranges and deep valleys (Figure 9C) and was historically difficult (Frolich and Schmidt, 2022). This might explain why pollen from the landrace Chapalote from the Mexican lowlands, and Oloton from Guatemala, had the lowest *P. ananatis* diversity, despite being proximal to the Mexican Highlands – their microbiomes became isolated. Interestingly, the landraces Dente Branco, Kulli and Cateto Nortista had intermediate levels of *P. ananatis* diversity even though they originate from South America – far from the center of maize diversification. One explanation may come from a recent report that South America served as a second site for ancient maize improvement that may have boosted Pan-American maize diversity (Kistler et al., 2020). However, if the maize pollen microbiome outside of the Mexican Highlands became geographically isolated from its origin, *P. ananatis* should have diversified independently, resulting in unique taxa – however, every non-Highland originating taxa was also present in at least one Highland originating landrace, i.e., there was a one-way decline in diversity.

Furthermore, the pollen of the U.S. modern maize inbred B73, released recently in 1972 (Schnable et al., 2009), contained high diversity of *P. ananatis* (Figure 9A). This is of interest, because B73 is the founder of the North American maize heterotic group Stiff Stalk which continues to be a parent in the majority of modern commercial hybrids (Bornowski et al., 2021). B73 originates from the Mexican highlands via its Northern flint lineage (Swarts et al., 2017), which might explain the origin of *P. ananatis* diversity in this inbred. Alternatively, since B73 includes multiple genotypes in its pedigree (Perez-Limón et al., 2022), perhaps breeders unknowingly restored ancestral *P. ananatis* diversity in maize pollen.

Outside of the Mexican Highlands, our preliminary evidence suggests that *P. ananatis* may have been replaced by other bacterial species (Figure 9G and Supplementary Table S5), thus maintaining overall pollen microbiome diversity. Specifically, *P. ananatis* taxa may have been partially replaced by *Lactococcus* taxa in 3 geographically distant landraces, namely Chapalote in lowland Northern Mexico, Oloton in Central America and Cateto Nortista in lowland Brazil; *P. ananatis* may have been partially replaced by *Erwinia* taxa in landrace Kulli in Bolivia in terms of read count and diversity, and by *Pseudomonas* in Dente Branco in Uruguay in terms of read count but not diversity (Figure 9G and Supplementary Table S5). If true, these observations would suggest that *P. ananatis* was only under active selection by farmers in the Mexican Highlands, i.e., because it provided benefits specific to that region due to selection pressures (e.g., tolerance to local disease or abiotic stress) that did not exist outside this region at the time of historical maize migrations. For example, there is evidence of an unusual extended drought in the ancient Mexican Highland city of Cantona from 200 B.C. to 1,300 A.D. (Bhattacharya et al., 2015). Ancient Mexican Highland soils may have been the original source of the *P. ananatis* diversity associated with maize, since soil is a well-known habitat for this bacterial species (Coutinho and Venter, 2009). *P. ananatis* can also originate from insect pests of crops (Sauer et al., 2015; Bing et al., 2022).

Furthermore, one interpretation of the apparent maintenance or reconstitution of the Mexican Highland diversity of *P. ananatis* in the modern U.S. inbred B73, along with moderate diversity of *Lactococcus* (Figures 9B−G), is that U.S. breeders, after mating diverse genotypes, phenotypically selected for traits associated with pollen bacteria that were favored by both traditional Mexican Highland farmers and non-Highland farmers.

A previous study attributed *Pantoea* speciation to the possession of the universal plasmid family, Large *Pantoea* Plasmids (LPP-1) (size range ~ 281-794 kb, and 310 kb in *P. ananatis*), derived from an ancestral plasmid that endured the expanded diversification (De Maayer et al., 2012). Interestingly, the data mining of this plasmid revealed genes that encode bundles of proteins essential for ecological adaptability and functional diversification (De Maayer et al., 2012). By analyzing the pan-genome of 19 *P. ananatis* strains, another study explained the high potential of *P. ananatis* to diversify and inhabit a wide range of ecological niches; the results showed two distinct clades, in addition to a horizontally transmissible accessory genome (mobilome) including plasmids, integrated prophages, integrative and conjugative elements and insertion elements (De Maayer et al., 2017). The ability of *P. ananatis* to diversify to adapt to different environments contradicts the one-way loss of diversity reported here, though supports its role in B73.

Perhaps, additional landraces or commercial genotypes will demonstrate novel diversification in *P. ananatis* not present in the Mexican Highlands. Alternatively, perhaps the *P. ananatis* reported here are ancestral, dominant, vertically transmitted strains, while new strain diversity is horizontally transmitted from native soils.

### The potential role of *Pantoea ananatis* in host maize plants

The genus *Pantoea* and the species *P. ananatis* can exhibit beneficial, mutualistic, or pathogenic associations with host plants including maize (Coutinho and Venter, 2009; Sheibani-Tezerji et al., 2015; de Maayer et al., 2017; Smits et al., 2019; Legein et al., 2020). Coutinho and Venter (2009) further reviewed that *P. ananatis* strains can be latent pathogens and saprophytes in plants. Differences in bacterial secretion systems have been proposed to modulate the precise type of interaction between *P. ananatis* and maize host plants (Sheibani-Tezerji et al., 2015).

In terms of whether pollen-associated *P. ananatis* taxa are maize pathogens, it is noteworthy that in maize, *P. ananatis* causes foliar White Spot disease, as well as Brown stalk rot, and necrotic spots and streaks (Coutinho and Venter, 2009; Sauer et al., 2015). However, we never observed any disease symptoms during our study. Furthermore, the maize seeds in this study came from major seedbanks which would have selected against plants with obvious visual disease symptoms.

Therefore, it is reasonable to suggest that some *P. ananatis* taxa associated with maize pollen may be commensals or possibly beneficial to host plants, following colonization of vegetative plant organs. Strains of *P. ananatis* have been shown to provide benefits to diverse host plants including growth promotion, ACC-deaminase activity, auxin production, nitrogen fixation, siderophore production, phosphorous solubilization, and biocontrol activities (Sheibani-Tezerji et al., 2015; Usuda et al., 2022). Notably, *P. ananatis* was isolated from seeds of the ancient maize landrace Jala and shown to have nitrogen fixation and auxin production potential (Johnston-Monje and Raizada, 2011); here, *P. ananatis* was shown to be present in Jala pollen. In wheat, *P. ananatis,* isolated from wheat spikes, exhibited biocontrol activity against *Fusarium graminearum* and its mycotoxins (Deroo et al., 2022) which is noteworthy since *F. graminearum* is an important fungal pathogen of maize seeds; it enters through silks (style), the same route used by pollen tubes to transmit sperm nuclei to ovules (Thompson and Raizada, 2018).

In terms of co-evolution, the presence of *P. ananatis* in onion, rice, maize, and sorghum (all monocots) and eucalyptus (a dicot) (Bragard et al., 2023) suggests that its association with plants began >100 million years ago. Interestingly, pathogenic *P. ananatis* has been shown to induce/trigger volatile organic compound (VOC) production from maize including common terpenes (e.g., linalool, pinene) known to attract pollinators (Delaney et al., 2015). *P. ananatis* is also present in the gut microbiome of some insects such as honeybee (Scheiner et al., 2020). Perhaps *P. ananatis* began its association with plants as a pathogen but was domesticated as an endophyte that could be vertically transmitted to seeds as shown in rice (Zhang et al., 2022), reinforced by its presence in maize seeds (Johnston-Monje and Raizada, 2011) initially to attract pollinators, but maintained in wind pollinated plants such as maize. Indeed, rather than bringing in novel traits by recruiting other risky bacterial species, host plants may have selected for *P. ananatis* diversification as a safe vector to acquire novel beneficial traits and to compete against potential pathogens attempting to be transmitted by pollen.

### The predicted maize Pan-American core pollen microbiome

Consistent with other plant microbiomes (Lynch and Neufeld, 2015; Jousset et al., 2017; Pascoal et al., 2021), the Pan-American maize pollen microbiome contained more rare taxa (with relative abundance <1%) than dominant taxa (Supplementary Table 4). Nevertheless, a core microbiome was predicted based on FL-PacBio, constituting 11 genera, most of which were Gram negative (Figures 5, 8A). Four of these were identified in all maize accessions tested: *Pantoea, Pseudomonas, Erwinia* and *Sphingomonas*; of these *Pantoea* and *Pseudomonas* dominated the Pan-American pollen microbiome in terms of prevalence and read counts. Additionally, *Lactococcus, Enterobacter, Microbacterium, Kluyvera*, *Chryseobacterium, Rosenbergiella*, and *Rhizobium* were prevalent. Only limited information exists about pollen microbiomes; however, a prior study of 12 plant species showed that *Pseudomonas*, *Rosenbergiella* and *Bradyrhizobium* were the most abundant bacterial genera out of 13 conserved core taxa (Manirajan et al., 2018). Here, at the species level, the Pan-American core pollen microbiome comprised 13 species based on full-length 16S rRNA gene sequencing (Figures 6, 8B). Of these, *P. ananatis* and *Pseudomonas rhizosphaerae* were present in all 17 maize accessions, *Pantoea vagans* in 16 accessions, *Lactococcus lactis* and *Kluyvera intermedia* in 15 accessions, *Microbacterium testaceum* in 14 accessions, followed by *Erwinia rhapontici, Pseudomonas lurida, Sphingomonas panni*, and *Sphingomonas paucimobilis* in 13 accessions.

Previous reports showed the potential contribution of pollen to vertical and/or horizontal transmission of plant pathogens, particularly fungi (Card et al., 2007). However, of the 13 bacterial species identified in the predicted Pan-American core pollen microbiome, only a subset of strains of two species are known maize pathogens: *P. ananatis* (Paccola-Meirelles et al., 2001) and *P. vagans* (maize stalk rot) (Brady et al., 2009). As discussed above, it seems unlikely that our seeds contained pathogenic strains. Similar to *P. ananatis,* some *P. vagans* strains can be beneficial to plants (Smits et al., 2010, 2019) which seems more likely here. Furthermore, of the 45 remaining bacterial species identified in the predicted Pan-American pollen microbiome, only some subspecies of *Pseudomonas syringae* (present in 13/54 pollen samples) have been identified as maize pathogens to the best of our knowledge (Jardine and Claflin, 2016).

Almost half of the core species of the predicted Pan-American maize pollen microbiome belonged to the genus *Pseudomonas*, including *Ps. rhizosphaerae*, as already noted. The genus *Pseudomonas* has remarkable versatility, allowing its species to adapt to diverse ecological niches (Teoh et al., 2021). *Ps. rhizosphaerae* is a growth promoting rhizobacterium, abundant in the rhizosphere of diverse plants including maize where it has the potential to fix nitrogen, solubilize phosphate, and produce siderophore and indole-3-acetic acid (Babalola et al., 2021).

*L. lactis* isolates were cultivated from the aerial root mucilage of Sierra Mixe, a Mexican highland landrace of maize from Oaxaca, proximal to several landraces in this study; Sierra Mixe was shown to have significant nitrogen fixation activity (Higdon et al., 2020). *L. lactis* isolates from cucurbit seeds were shown to secrete auxin and acetoin, solubilize phosphate and have antifungal activities (Khalaf and Raizada, 2016, 2018, 2020).

In terms of the remaining pollen core species, little has been reported about *K. intermedia* in maize, though it has been shown to enhance phosphate mobilization by promoting mycorrhizal colonization (Liu et al., 2021)*. M. testaceum* was identified from the roots and leaves of domesticated rice and shown to exhibit diverse growth promoting traits including mineral solubilization (phosphate, potassium, and zinc) and production of gibberellic acid and auxin (Borah et al., 2021). *E. rhapontici* has been reported as a pathogen of diverse crops (Hsieh et al., 2010; Tambong, 2022); however, we could not find any reports of it acting as a maize pathogen. *Sphingomonas paucimobilis* was identified as a widespread nitrogen-fixing endophyte of wild and cultivated rice roots in Nepal (Engelhard et al., 2000). Finally, *Sphingomonas panii* has been identified as a root endophyte of wheat, along with soybean and lettuce, and shown to have plant growth promoting activities (Li et al., 2023).

At the genus level, nitrogen-fixing rhizobium also constituted part of the predicted Pan-American core microbiome, which was also previously observed in pollen across plant species (Manirajan et al., 2018), suggestive that rhizobia can be vertically transmitted. In addition to these core species, pollen from some of the maize accessions also carried bacteria previously shown to be seed endophytes of maize landraces and wild relatives (Johnston-Monje and Raizada, 2011). These maize seed endophytes exhibited potent plant phosphate and nitrogen acquisition benefits, including, respectively, *Enterobacter asburiae* (Shehata et al., 2017) and *E. roggenkampii* (originally assigned *E. cloacea*) (Dumigan et al., 2018; Shehata et al., 2018).

With respect to connecting this study to seeds, since pollen tubes extend through the silk channel to deliver sperm nuclei to ovules (Zhou et al., 2017), it is noteworthy that several of the predicted pollen core taxa at the genus level (*Pantoea, Pseudomonas, Sphingomonas, Lactococcus, Rhizobium, Chryseobacterium*) were previously observed in pollinated silk core microbiomes; of these *Pantoea* was the most prevalent and dominant genus (Khalaf et al., 2021). This raises the possibility that these pollen-associated bacteria can migrate toward the ovule, but whether they ultimately colonize the embryo or endosperm is not known. However, several of the pollen-associated taxa in our current study were previously shown to be maize seed endophytes in a screen of Pan-American landraces, including: *Stenotrophomonas maltophilia*, *Enterobacter asburiae*, *Pseudomonas oryzihabitans*, *Ps. putida*, *Pantoea dispersa*, *E. roggenkampii* (formerly *E. cloacea*), and *Bacillus megaterium* (non-core pollen taxa), as well as *P. ananatis* and *P. vagans* (pollen core taxa) (Johnston-Monje and Raizada, 2011). Moving forward, we suggest detailed tracking of the transgenerational transmission of *P. ananatis* and other pollen core members across maize silks and seeds.

Ultimately, the extent to which the common field environment in which the maize plants were grown in this study affected this core will need to be determined by sampling pollen in other environments. However, pollen is a reproductive tissue which presumably would have selected against host-microbe promiscuity. Consistent with this, pollen is known to express a high number of anti-microbial peptides that can act as antibiotics (Zasloff, 2017). In terms of the contribution of airborne bacteria to the pollen microbiome, it should be noted that maize pollen is enclosed in anthers until release and thereafter only exposed to air currents for minutes to hours, with viability lost rapidly within 1-2 h (Schoper et al., 1986). Nevertheless, additional years of field sampling will be required to distinguish environmental impacts from stable, core taxa.

### Inconsistency in the maize pollen microbiome between biological replicates: technical or a deliberate driver of host genetic diversification?

In this study, there was inconsistency in the pollen microbiome between biological replicates. As a result, we were cautious in our interpretation, primarily focusing on consistent results related to the predicted core microbiome and patterns. The variation between replicates could potentially be explained by several factors:

First, and most significantly, the 16S read count from pollen was very low, especially using PacBio (Supplementary Table S4), which may have resulted in stochasticity in the initial PCR cycles (Kebschull and Zador, 2015). As described above, to the best of our knowledge, this study was the first to use PacBio SMRT technology with pollen samples; however, replicate variation was also observed using Illumina MiSeq (Supplementary Figures S1, S2). Pollen may inherently have a low bacterial titer. Furthermore, despite best attempts, many samples had very low DNA yields, likely related to technical challenges in breaking the exine, a degradation-resistant sporopollenin biopolymer that constitutes the outermost wall of the pollen grain (Diego-Taboada et al., 2014; Park et al., 2016). Further optimization of the pollen DNA extraction protocol may be helpful (Swenson and Gemeinholzer, 2021).

Variation between biological replicates is normal in microbiome studies, especially under field conditions due to high spatial variability in the soil microbiome (Alberdi et al., 2019) which may have contributed to the pollen microbiome. Here the high-labor (e.g., digging large, deep holes) required to transplant hundreds of late-stage maize plants to the field to induce flowering prevented us from having a higher replicate number which could have buffered across this variability. Pollen microbes have been observed on the pollen surface (Manirajan et al., 2016; Obersteiner et al., 2016), which may have been affected by the randomness of insect visitation and airborne spores. In this context, it was noteworthy that the genera *Buchnera* and *Wolbachia,* which are well known insect endosymbionts, essential for insect nourishment and reproduction (Sharma et al., 2021), were identified as members of the maize pollen microbiome from both primer sets; whether these were contaminants or pollen endophytes however requires future experiments. Despite a quality control strategy to remove non-pollen debris including insects, there may have been sporadic insect microbiome contamination. Furthermore, explorations of the pollen mycobiome and the potential influence of the surrounding microflora, notably the air-borne fungal spores, were lacking here, and worth investigating in future experiments. It would also be interesting to study the surface morphology and biochemistry of the pollen among these maize accessions (e.g., using scanning electron microscopy) to determine if they affect the pollen microbiome.

Third, except for B73, all the host accessions in this study were primarily ancient and/or pre-Columbian landraces which are known to have high levels of intra-landrace genetic variability (Zerjal et al., 2012), which in turn may have influenced pollen microbiome composition between biological replicates. Siblings grew at different rates, leading to variability in pollen collection times between replicates; variation in the temperature/humidity may have especially affected the epiphytic bacteria. Replicate diversity was observed despite attempts to pool pollen from 3 to 5 plants, likely because some plants produced little pollen due to transplant shock and poor adaptability of these tropical and sub-tropical landraces to a new temperate climate and to a non-native soil. To compensate, we collected and pooled pollen from individual plants daily, resulting in 2500+ pollen bags collections. In the future, as already noted, it will be interesting to repeat this study using pollen collected in their native habitats across the Americas. However, as microbiome variability was also observed within the temperate inbred B73, it is interesting to speculate whether pollen microbiome diversity is programmed, as an evolutionary driver of host phenotypic diversity, parallel to meiosis.

### Comparison of FL-PacBio versus V4-MiSeq

Microbiome taxonomic discrepancies have been reported when using different primers targeting different 16S rRNA gene regions within different sequencing platforms (Wagner et al., 2016). However, few microbiome studies have used multiple high throughput sequencing (HTS) platforms simultaneously, particularly Illumina MiSeq (short-read sequencing/s generation) and PacBio SMRT (long-read sequencing/third generation), for the taxonomic profiling of microbial communities (D’Amore et al., 2016; Hahn et al., 2016; Brede et al., 2020). PacBio-SMRT sequences the target amplicon multiple times in tandem, and then a consensus sequence is generated to achieve high accuracy (Fichot and Norman, 2013; Schloss et al., 2016). To the best of our knowledge, our study is the first to explore a pollen microbiome using both approaches.

In prior studies, V4-MiSeq and PacBio sequencing technologies were reported to be bias-prone in different ways and levels (Hahn et al., 2016; Wagner et al., 2016). Here, FL-PacBio lagged far behind V4-MiSeq in terms of generated read counts (Supplementary Table S4, Figure 7B), and the assigned number of taxa was approximately half those identified by V4-MiSeq (Figure 6A and Supplementary Table S4), which upon data analysis dramatically influenced the identification of pollen core members (Figures 8A,B and Supplementary Figure S7). These discrepancies significantly influenced the calculated alpha diversity metrics (Figures 7A−D). These results were expected and consistent with comparative benchmarking studies (Schirmer et al., 2015; D’Amore et al., 2016; Hahn et al., 2016). As a short-read sequencing platform, Illumina MiSeq is advantageous to FL-PacBio by generating high read counts, up to 25 million reads per run (Pichler et al., 2018), and as a result, higher taxonomic diversity (Hahn et al., 2016). Indeed, here, V4-MiSeq predicted much greater diversity than FL-PacBio at the phyla down to the genus level. The taxonomic assignment of pollen microbiota identified from the sequencing platforms was consistent at higher taxonomic ranks until/up to the order level, whereas significant taxonomic mismatches were noticed starting from the family level down to the species level. Though the 16S V4 hypervariable region is the most commonly used in microbiome studies, such short-read sequencing has previously been reported to be ineffective in assigning taxonomy below the genus level compared to FL-PacBio (Hahn et al., 2016; Johnson et al., 2019). By contrast, the longer read length achieved by FL-PacBio (1,500 bp) achieves finer taxonomic resolution (Fichot and Norman, 2013; Schloss et al., 2016). To minimize discrepancies between V4-MiSeq versus FL-PacBio generated microbiomes introduced after sequencing during data processing, in this study, both sets of reads were similarly curated and analyzed (see Materials and Methods).

Here, V4-MiSeq missed and/or under-estimated dominant genera (>1% RA) identified by FL-PacBio, specifically *Pantoea*, *Enterobacter*, *Kluyvera*, *Rosenbergiella*, and *Microbacterium,* while it dramatically over-represented *Klebsiella* (Figure 3E and Table 2). Critically, *Pantoea* was identified as a rare taxon of the pollen microbiome using V4-MiSeq but a prominent core member using FL-PacBio, as detailed above (e.g., Figure 9), when accounting for both abundance and prevalence, in agreement with a prior study (Palkova et al., 2021). 16S V4 primers have been shown to bias against *Pantoea* along with *Microbacterium* (V3-V4) (Abellan-Schneyder et al., 2021; Palkova et al., 2021). Furthermore, V4-MiSeq exclusively assigned (> 0.1 RA) some genera including *Klebsiella*, *Citrobacter*, *Raoultella*, *Yersinia*, *Duganella*, *Xylophilus*, *Neorhizobium*, *Brevundimonas*, *Herbiconiux*, *Blastococcus*, *Nocardioides*, and *Paenibacillus* (Table 2). These results are consistent with the prior literature which showed that V4-MiSeq mis-identifies *Enterobacteriaceae* family members including *Klebsiella* and over-estimates their prevalence (Greay et al., 2019). Finally, V4-MiSeq assigned archaea which were not identified by FL-PacBio; a prior study (Lam et al., 2020) showed that PacBio could be effectively combined with archaea-specific primers.

With respect to *Pantoea*, we previously used V4-MiSeq to explore the pollinated silk microbiome, but there, *Pantoea* was the most prevalent and abundant taxon across all sample groups (Khalaf et al., 2021). Perhaps the current discrepancy between pollen and silk microbiomes regarding *Pantoea* was due to: differential abundance of specific taxa that compete with *Pantoea* for 16S primer annealing; inherent tissue differences (e.g., metabolites affecting primer annealing) (Bell et al., 2016); use of different DNA extraction kits (Qiagen and CTAB for silks, versus ZymoBIOMIC ™ for pollen); and different bead-beating methods (Swenson and Gemeinholzer, 2021; Prudnikow et al., 2023). Furthermore, processing and sequencing were performed at different third-party core facilities which may have varied in sample processing techniques, technical protocols, kits, reagents and equipment (Hiergeist et al., 2016). These results suggest that multiple factors contribute to reported microbiome taxonomy, but in general, full-length 16S rRNA gene sequencing is more effective than partial 16S rRNA gene sequencing with respect to achieving higher and accurate taxonomic resolution (Franzén et al., 2015).

Collectively, these results show that MiSeq and PacBio NGS platforms are complementary microbiome sequencing techniques. Perhaps V4-MiSeq is useful for initial surveys of new microbiomes to obtain an overview of the diversity present, but it should be followed by FL-PacBio for high resolution taxonomic profiling. The low error rates of FL-PacBio-SMRT, combined with its long sequence reads, permit it to reveal diversity within microbial species (e.g., *Pantoea ananatis*), especially important when comparing closely related host accessions (e.g., landraces).

## Conclusion

Here we explored the diversity and conservation of the pollen microbiome of maize in the Americas and discovered that maize pollen carries a rich diversity of bacteria, of which ~20% belong to a single species, *Pantoea ananatis.* The diversity within *P. ananatis* did not appear to be random, but rather reflected the phylogenetic and migratory history of its host. There was significant variation in pollen microbiome communities between replicates, which may have been technical, or preliminary evidence that the pollen microbiome contributes to host diversification. The results also demonstrated the benefit of combining Illumina MiSeq and PacBio-SMRT to achieve a comprehensive understanding of the pollen microbiome. Future studies are needed to explore maize pollen microbiomes in their native habitats across multiple seasons, and to determine whether the pollen associated bacteria identified here are transmitted to seeds and their impact on host plants.

## Data availability statement

The datasets presented in this study can be found in online repositories. The names of the repository/repositories and accession number(s) can be found at: https://www.ncbi.nlm.nih.gov/, PRJNA766023 https://www.ncbi.nlm.nih.gov/, PRJNA773232.

## Author contributions

EK: Conceptualization, Data curation, Formal analysis, Investigation, Methodology, Validation, Visualization, Writing – original draft, Software. AS: Formal analysis, Investigation, Methodology, Writing – original draft, Conceptualization. MR: Investigation, Methodology, Writing – review & editing. BMc: Investigation, Methodology, Writing – review & editing. MNR: Writing – review & editing, Conceptualization, Funding acquisition, Project administration, Supervision.

## Footnotes

^1^https://climate.weather.gc.ca/

## Supplementary material

Supplementary Figure S1 A phylogenetic tree of all bacterial genera (234 genera) composing the Pan-American maize pollen microbiome as identified by V4-MiSeq sequencing. **(A)** Circular barchart showing the prevalence of each bacterial genus across maize accessions. **(B)** Circular barchart showing the prevalence of each bacterial genus across pollen samples. The genera which are bolded are the most prevalent.

Supplementary Figure S2 A phylogenetic tree of bacterial genera with RA ≥ 0.1% (32 genera) present in the Pan-American maize pollen microbiome as identified by V4-MiSeq sequencing. The phylogenetic tree is annotated as follows: **(A)** a single-colored barchart showing the prevalence of genera across maize accessions; **(B)** a single-colored barchart showing the prevalence of each bacterial genus across pollen samples; **(C)** a vertical heatmap of IHS-transformed read counts for each genus. Pollen samples are grouped by maize accession and replicate number, arranged from north to south according to the latitude at which they originated; **(D)** a heatmap of IHS-transformed bacterial read counts at the genus level across maize accessions clustered by rainfall; **(E)** a multi-bar chart displaying the average of IHS-transformed bacterial read counts for maize accessions clustered by their geographical population based on Matsuoka et al. (2002). In the geographic population legend, the number in brackets denotes the number of maize accessions in that population. ASV1_Klebsiella, ASV_4 Pseudomonas, and ASV11_Sphingomonas are displayed in weighted font to represent the most dominant and prevalent genera in the pollen microbiome across all Pan-American maize accessions.

Supplementary Figure S3 A phylogenetic tree of the consensus of bacterial genera sequences identified by FL-PacBio (39 genera) and V4-MiSeq (with threshold of RA ≥ 0.1%, 32 genera). The phylogenetic tree is annotated as follows: **(A)** a single-colored barchart showing the prevalence of genera across maize accessions; **(B)** a single-colored barchart showing the prevalence of each bacterial genus across pollen samples; **(C)** a vertical heatmap of IHS-transformed read counts for each genus. Pollen samples are grouped by maize accession and replicate number, arranged from north to south according to the latitude at which they originated.

Supplementary Figure S4 A phylogenetic tree of all bacterial orders composing the Pan-American maize pollen microbiome as identified by V4-MiSeq sequencing. The phylogenetic tree is annotated as follows: **(A)** a single-colored barchart showing the prevalence of bacterial orders across maize accessions; **(B)** a single-colored barchart showing the prevalence of each bacterial order across pollen samples; **(C)** a vertical heatmap of IHS-transformed read counts for each order. Pollen samples are grouped by maize accession and replicate number, arranged from north to south according to the latitude at which they originated; **(D)** a heatmap of IHS-transformed bacterial read counts at the order level across maize accessions clustered by rainfall; **(E)** a multi-bar chart displaying the average of IHS-transformed bacterial read counts for maize accessions clustered by their geographical population based on Matsuoka et al. (2002). In the geographic population legend, the number in brackets denotes the number of maize accessions in that population. Bacterial orders displayed in weighted red font represent the combined most dominant and prevalent bacterial orders with RA ≥ 1% from V4-MiSeq and/or FL-PacBio sequencing across all Pan-American maize accessions. Those that are dominant/prevalent: from both sequencing technologies, they were ASV1_Enterobacterales, ASV2_Pseudomonadales, ASV3_Lactobacillales, ASV4_Flavobacteriales, ASV6_Sphingomonadales; from V4-MiSeq, it was ASV11_Rhizobiales; from FL-PacBio, it was ASV12_Micrococcales.

Supplementary Figure S5 Phylogenetic tree of all bacterial genera composing the Pan-American maize pollen microbiome identified by FL-PacBio sequencing and arranged by rainfall at the sites where the accessions originated. The phylogenetic tree is annotated as follows: **(A)** a single-colored barchart showing the prevalence of genera across maize accessions; **(B)** a single-colored barchart showing the prevalence of each bacterial genus across pollen samples; **(C)** a vertical heatmap of IHS-transformed read counts for each genus. Pollen samples are grouped by maize accession and replicate number, arranged by rainfall at the sites where the accessions originated; **(D)** a heatmap of IHS-transformed bacterial read counts at the genus level across maize accessions clustered by rainfall; **(E)** a multi-bar chart displaying the average of IHS-transformed bacterial read counts for maize accessions clustered by their geographical population based on Matsuoka et al. (2002). In the geographic population legend, the number in brackets denotes the number of maize accessions in that population. ASV2_Pseudomonas, and ASV3_Pantoea are displayed in weighted font represent the most dominant and prevalent genera in the pollen microbiome across all Pan-American maize accessions.

Supplementary Figure S6 Visualization of multiple sequence alignment (MSA) of taxonomically closely related genera identified by FL-PacBio and V4-MiSeq. The red boxed sequences represent 100% identical sequences for ASV6_Erwinia_PacBio, ASV9_Enterobacter_PacBio, and ASV1_Klebsiella_MiSeq. The latter 2 taxa names are highlighted in black to denote closely related genera of the Enterobacteriaceae family. The alignment was visualized using SeaView software (version 4.7) (Gouy et al., 2010).

Supplementary Figure S7 A phylogenetic tree of the Pan-American pollen core microbiome at the genus level identified by V4-MiSeq sequencing. The core microbiome was identified at a prevalence threshold ≥ 50% across samples and 70% of host accessions. The geometric shapes represent IHS-transformed read counts and their sizes are proportional to the calculated values.

Supplementary Figure S8 A hierarchical clustering heatmap of Pseudomonas rhizosphareae taxa as a highly prevalent pollen core member. The figure shows the distribution of nine taxa of *Ps. rhizosphareae* across maize accessions at a prevalence threshold ≥ 50% across samples and 70% of host accessions. The color scale bar (1–4) represents the count of pollen sample replicates within each maize accession. Along the *x*-axis, the maize accession name is noted, followed by the number of replicates, then the count of *Ps. rhizosphareae* taxa, followed by a code (a number followed by M) where the number represents the placement of the maize accession on the phylogenetic tree of Matsuoka et al. (2002) constructed based on microsatellite data (for the number, see supporting information, Figure 4B and Table 1 in Matsuoka et al. (2002). The X means the maize accession was not included in the Matsuoka study.

Supplementary Figure S9 Statistical comparisons of select core members of the pollen microbiome of maize landraces clustered by their geographical origin of diversification. The statistical comparisons were performed on the taxa and read counts of *P. ananatis* and *L. lactis* which were the most prevalent and diverse bacterial species identified by FL-PacBio. The values of each microbiome parameter are displayed as boxplots where each dot represents a unique landrace. The mean of the two geographic populations is compared using the Mann-Whitney U / Wilcoxon Rank Sum Test. Shown are **(A)** *P. ananatis* taxa count, **(B)** *P. ananatis* read count, **(C)** *L. lactis* taxa count, **(D)** *L. lactis* read count.
