# Supplementary figures and images for: Conservation and diversity of the pollen microbiome of Pan-American maize using PacBio and MiSeq
Source: Front Microbiol. 2023 Dec 21;14:1276241. doi: 10.3389/fmicb.2023.1276241 (PMC10764481; doi:10.3389/fmicb.2023.1276241)

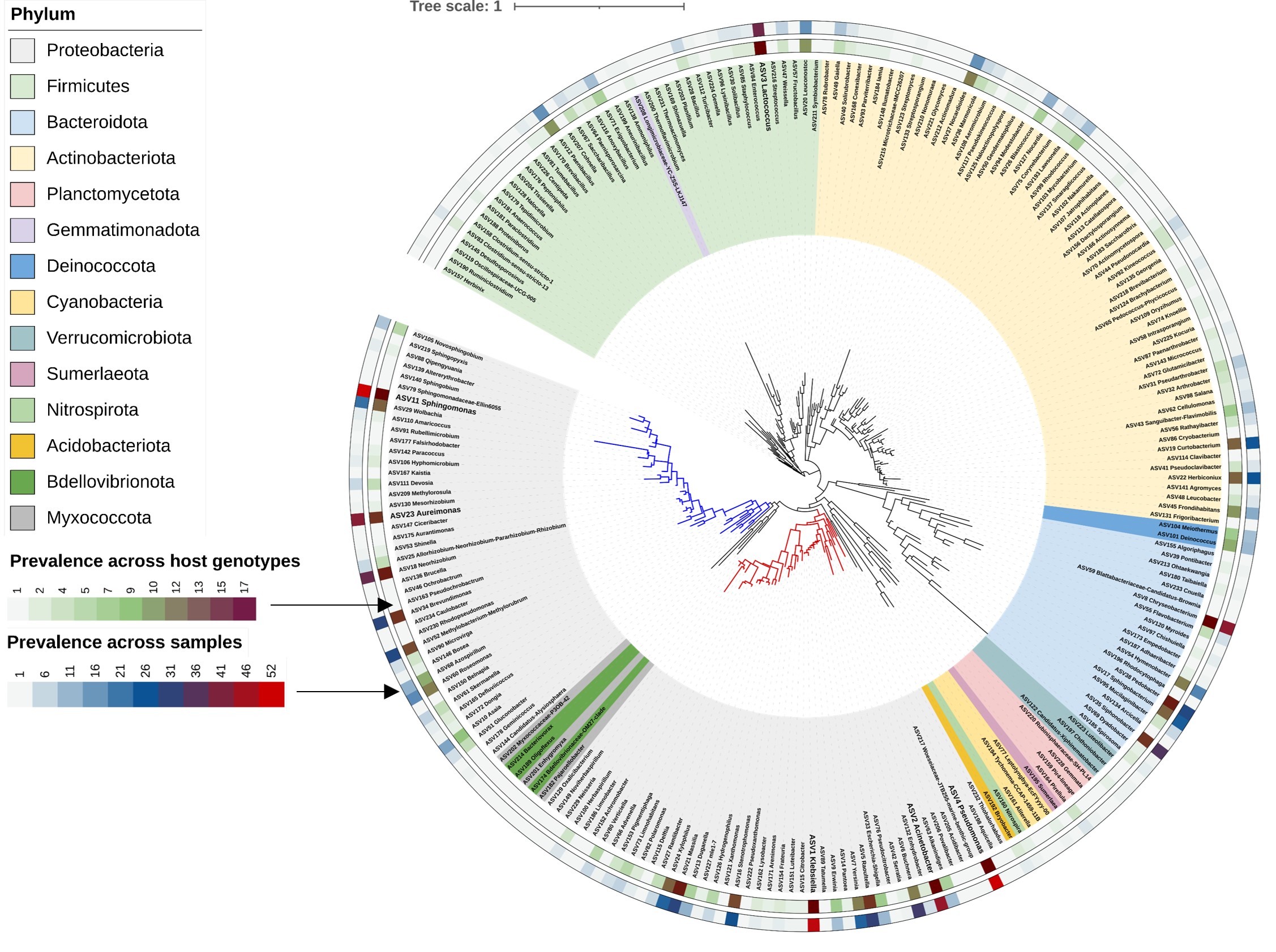

Supplement: Supplementary Figure S1 — A phylogenetic tree of all bacterial genera (234 genera) composing the Pan-American maize pollen microbiome as identified by V4-MiSeq sequencing. (A) Circular barchart showing the prevalence of each bacterial genus across maize accessions. (B) Circular barchart showing the prevalence of each bacterial genus across pollen samples. The genera which are bolded are the most prevalent. [file Image_1.JPEG]

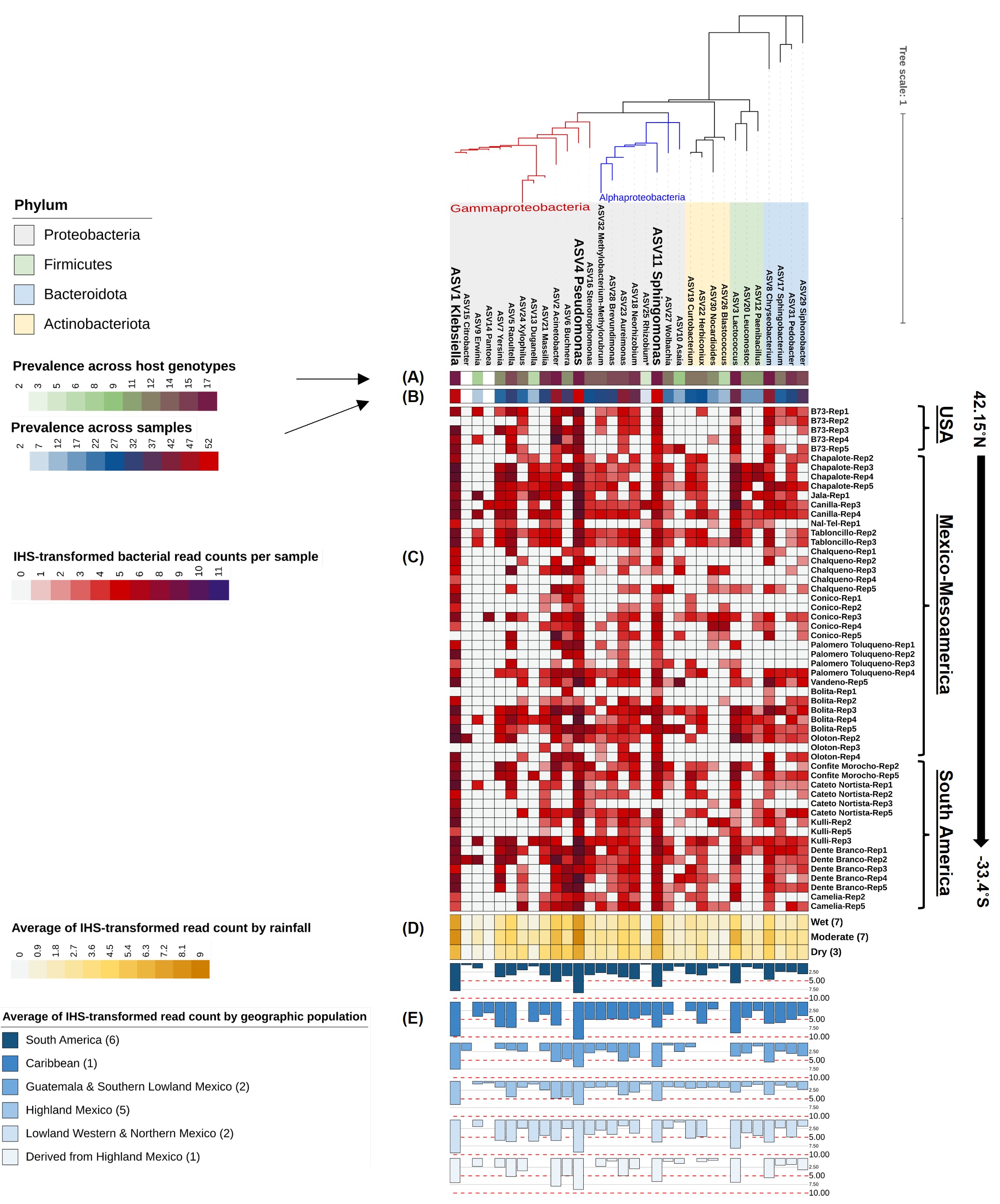

Supplement: Supplementary Figure S2 — A phylogenetic tree of bacterial genera with RA ≥ 0.1% (32 genera) present in the Pan-American maize pollen microbiome as identified by V4-MiSeq sequencing. The phylogenetic tree is annotated as follows: (A) a single-colored barchart showing the prevalence of genera across maize accessions; (B) a single-colored barchart showing the prevalence of each bacterial genus across pollen samples; (C) a vertical heatmap of IHS-transformed read counts for each genus. Pollen samples are grouped by maize accession and replicate number, arranged from north to south according to the latitude at which they originated; (D) a heatmap of IHS-transformed bacterial read counts at the genus level across maize accessions clustered by rainfall; (E) a multi-bar chart displaying the average of IHS-transformed bacterial read counts for maize accessions clustered by their geographical population based on Matsuoka et al. (2002). In the geographic population legend, the number in brackets denotes the number of maize accessions in that population. ASV1_Klebsiella, ASV_4 Pseudomonas, and ASV11_Sphingomonas are displayed in weighted font to represent the most dominant and prevalent genera in the pollen microbiome across all Pan-American maize accessions. [file Image_2.JPEG]

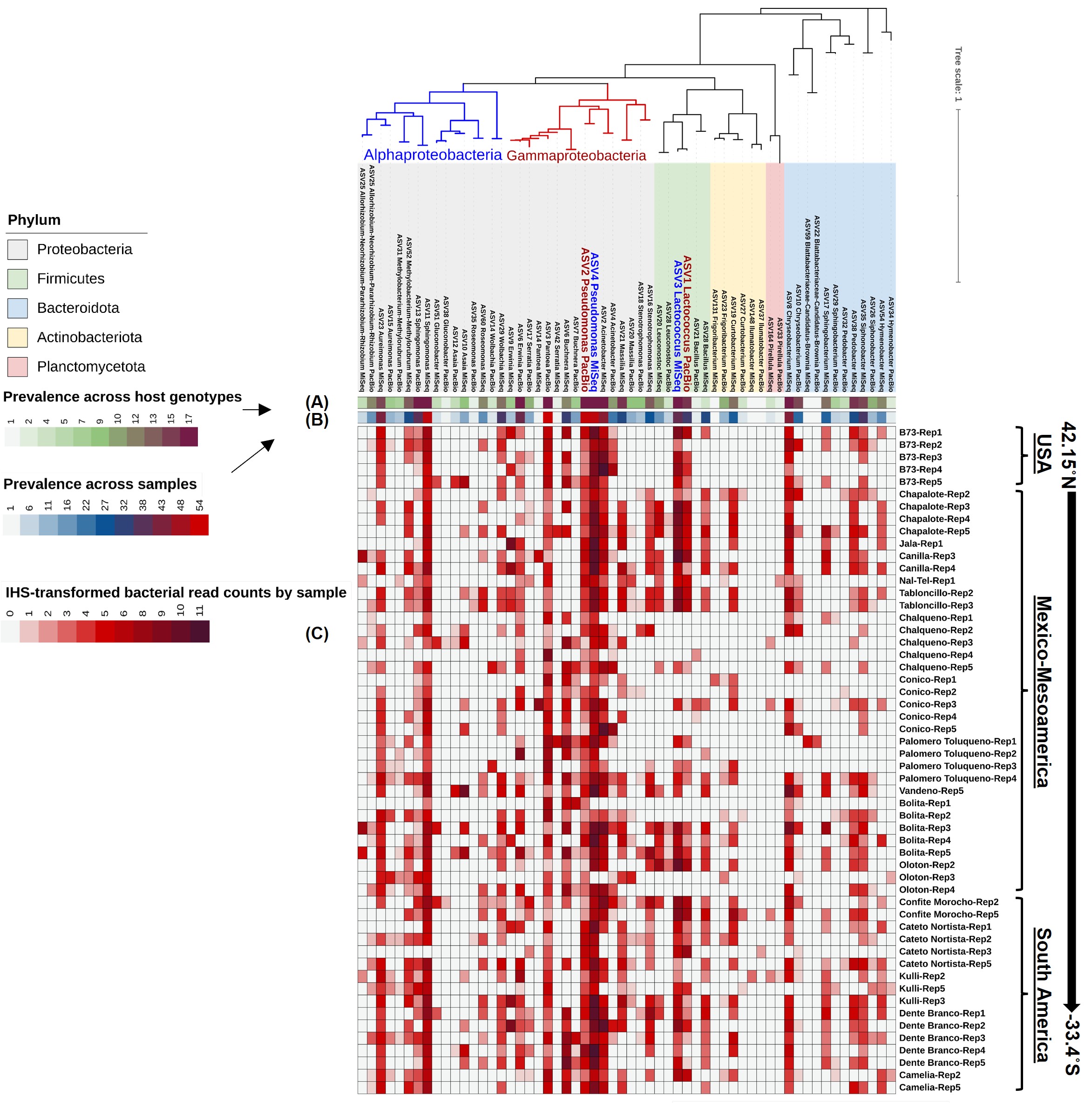

Supplement: Supplementary Figure S3 — A phylogenetic tree of the consensus of bacterial genera sequences identified by FL-PacBio (39 genera) and V4-MiSeq (with threshold of RA ≥ 0.1%, 32 genera). The phylogenetic tree is annotated as follows: (A) a single-colored barchart showing the prevalence of genera across maize accessions; (B) a single-colored barchart showing the prevalence of each bacterial genus across pollen samples; (C) a vertical heatmap of IHS-transformed read counts for each genus. Pollen samples are grouped by maize accession and replicate number, arranged from north to south according to the latitude at which they originated. [file Image_3.JPEG]

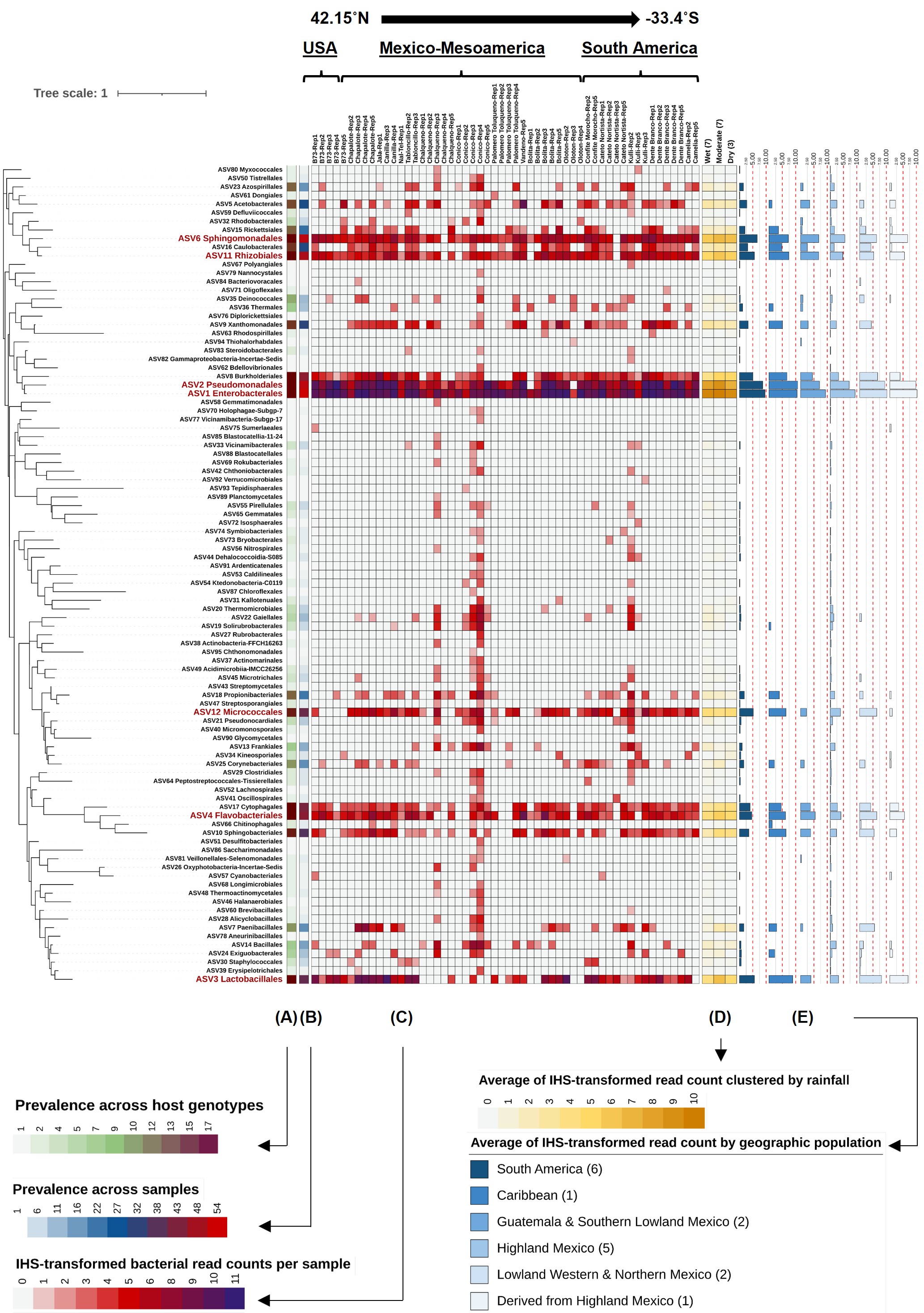

Supplement: Supplementary Figure S4 — A phylogenetic tree of all bacterial orders composing the Pan-American maize pollen microbiome as identified by V4-MiSeq sequencing. The phylogenetic tree is annotated as follows: (A) a single-colored barchart showing the prevalence of bacterial orders across maize accessions; (B) a single-colored barchart showing the prevalence of each bacterial order across pollen samples; (C) a vertical heatmap of IHS-transformed read counts for each order. Pollen samples are grouped by maize accession and replicate number, arranged from north to south according to the latitude at which they originated; (D) a heatmap of IHS-transformed bacterial read counts at the order level across maize accessions clustered by rainfall; (E) a multi-bar chart displaying the average of IHS-transformed bacterial read counts for maize accessions clustered by their geographical population based on Matsuoka et al. (2002). In the geographic population legend, the number in brackets denotes the number of maize accessions in that population. Bacterial orders displayed in weighted red font represent the combined most dominant and prevalent bacterial orders with RA ≥ 1% from V4-MiSeq and/or FL-PacBio sequencing across all Pan-American maize accessions. Those that are dominant/prevalent: from both sequencing technologies, they were ASV1_Enterobacterales, ASV2_Pseudomonadales, ASV3_Lactobacillales, ASV4_Flavobacteriales, ASV6_Sphingomonadales; from V4-MiSeq, it was ASV11_Rhizobiales; from FL-PacBio, it was ASV12_Micrococcales. [file Image_4.JPEG]

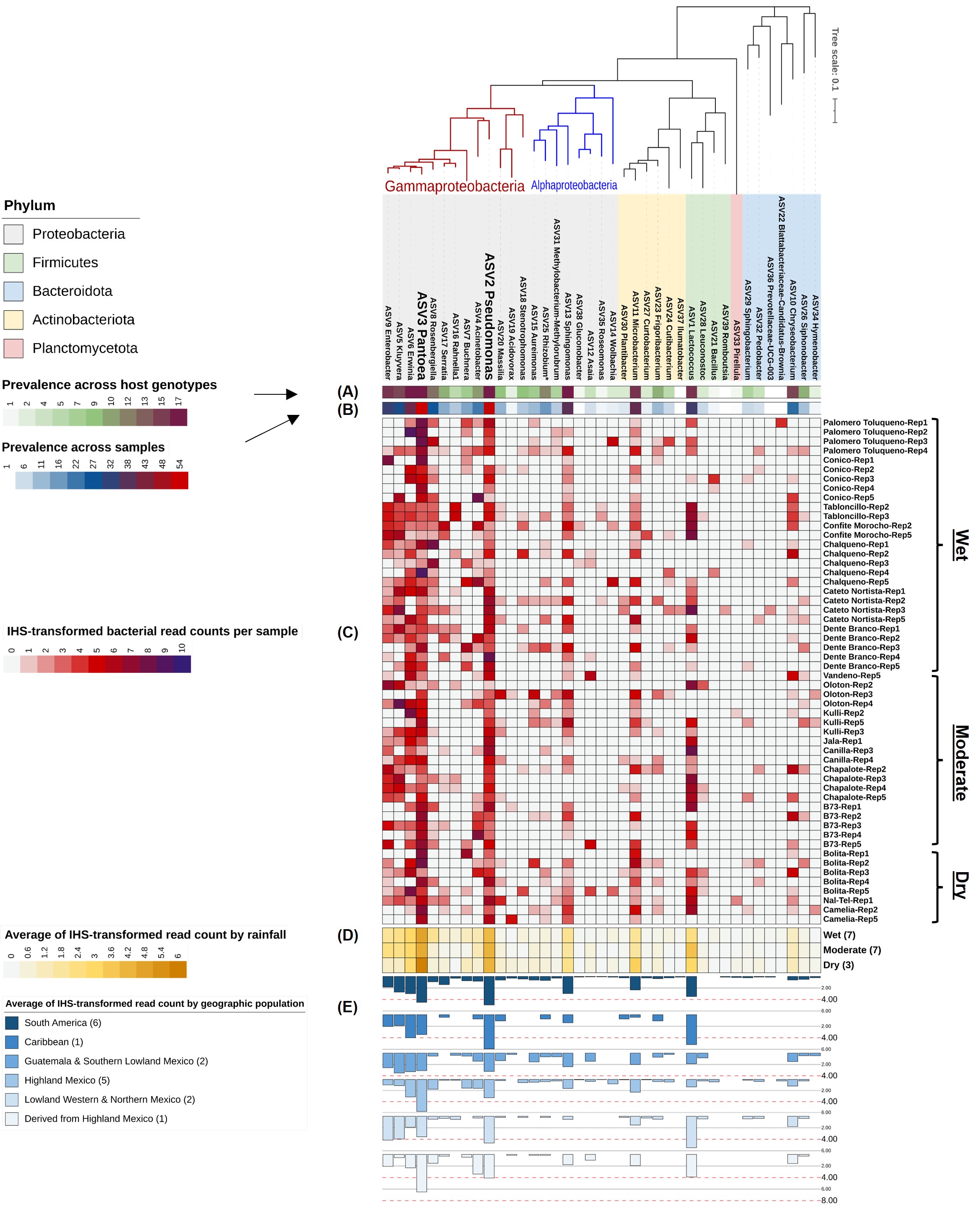

Supplement: Supplementary Figure S5 — Phylogenetic tree of all bacterial genera composing the Pan-American maize pollen microbiome identified by FL-PacBio sequencing and arranged by rainfall at the sites where the accessions originated. The phylogenetic tree is annotated as follows: (A) a single-colored barchart showing the prevalence of genera across maize accessions; (B) a single-colored barchart showing the prevalence of each bacterial genus across pollen samples; (C) a vertical heatmap of IHS-transformed read counts for each genus. Pollen samples are grouped by maize accession and replicate number, arranged by rainfall at the sites where the accessions originated; (D) a heatmap of IHS-transformed bacterial read counts at the genus level across maize accessions clustered by rainfall; (E) a multi-bar chart displaying the average of IHS-transformed bacterial read counts for maize accessions clustered by their geographical population based on Matsuoka et al. (2002). In the geographic population legend, the number in brackets denotes the number of maize accessions in that population. ASV2_Pseudomonas, and ASV3_Pantoea are displayed in weighted font represent the most dominant and prevalent genera in the pollen microbiome across all Pan-American maize accessions. [file Image_5.JPEG]

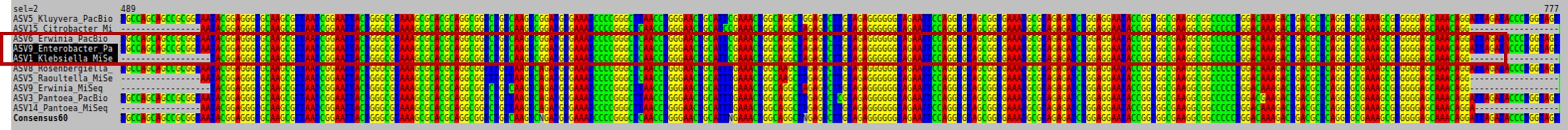

Supplement: Supplementary Figure S6 — Visualization of multiple sequence alignment (MSA) of taxonomically closely related genera identified by FL-PacBio and V4-MiSeq. The red boxed sequences represent 100% identical sequences for ASV6_Erwinia_PacBio, ASV9_Enterobacter_PacBio, and ASV1_Klebsiella_MiSeq. The latter 2 taxa names are highlighted in black to denote closely related genera of the Enterobacteriaceae family. The alignment was visualized using SeaView software (version 4.7) (Gouy et al., 2010). [file Image_6.JPEG]

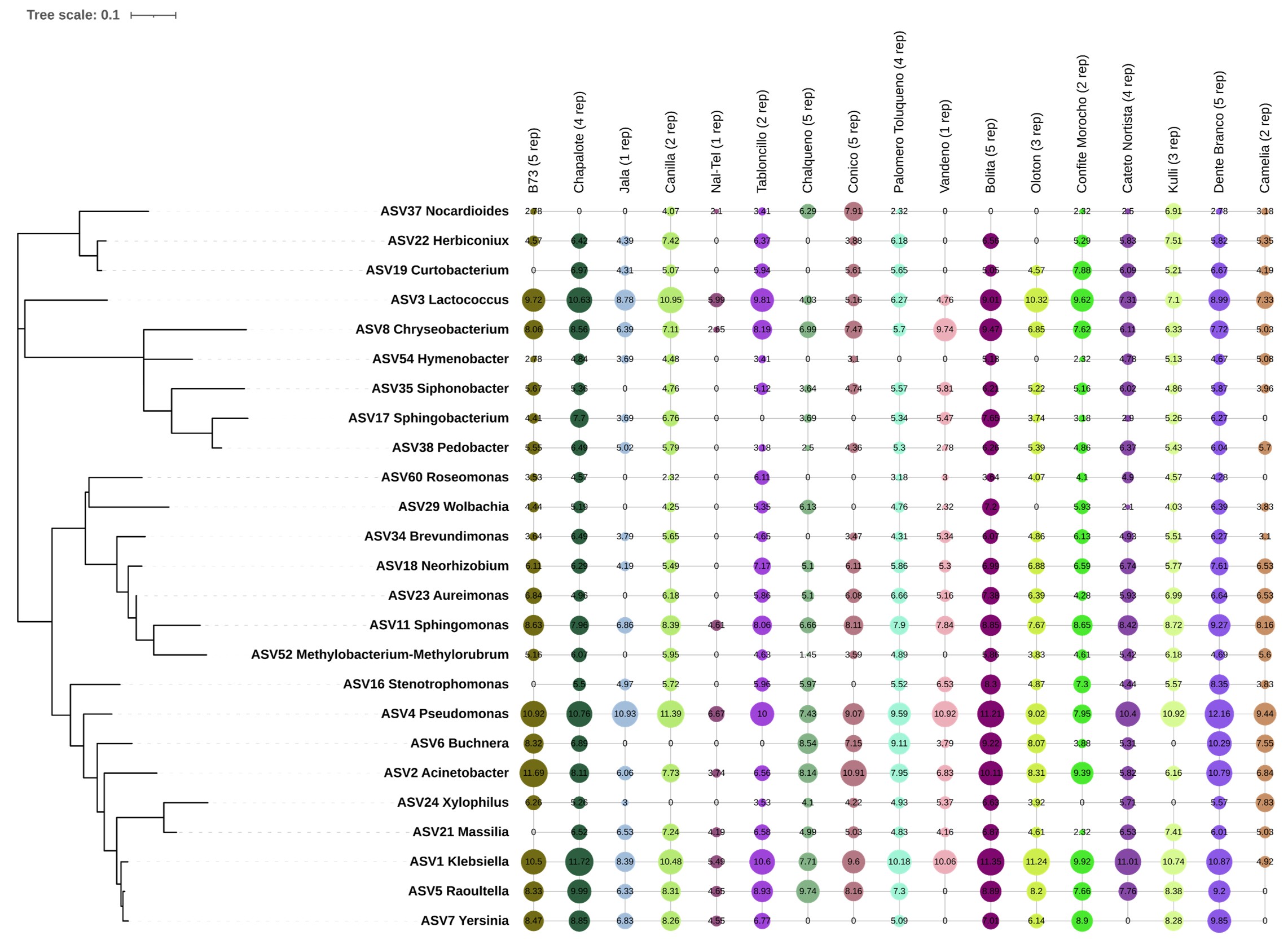

Supplement: Supplementary Figure S7 — A phylogenetic tree of the Pan-American pollen core microbiome at the genus level identified by V4-MiSeq sequencing. The core microbiome was identified at a prevalence threshold ≥ 50% across samples and 70% of host accessions. The geometric shapes represent IHS-transformed read counts and their sizes are proportional to the calculated values. [file Image_7.JPEG]

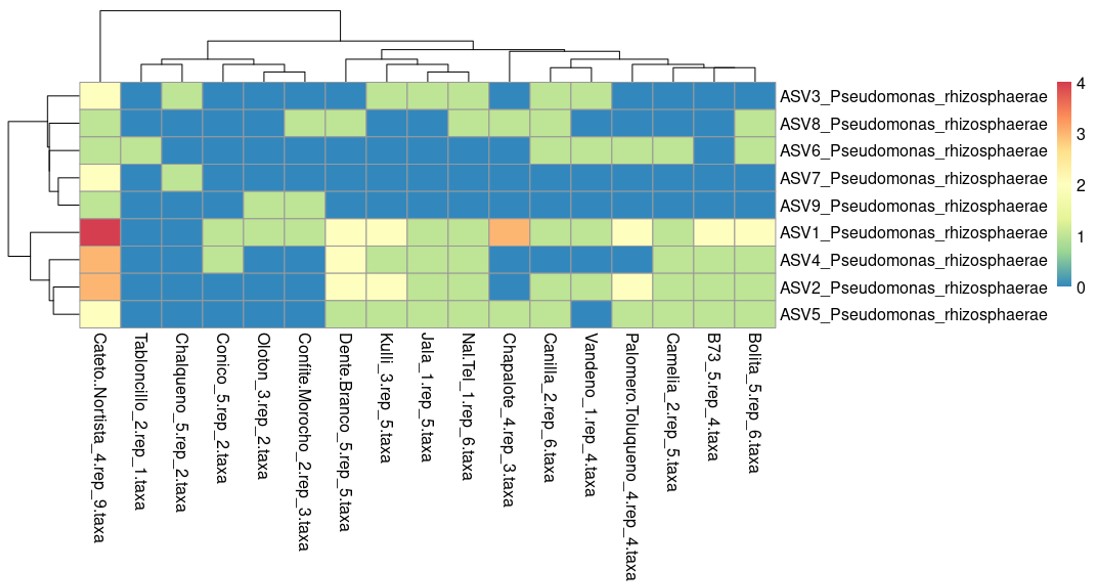

Supplement: Supplementary Figure S8 — A hierarchical clustering heatmap of Pseudomonas rhizosphareae taxa as a highly prevalent pollen core member. The figure shows the distribution of nine taxa of Ps. rhizosphareae across maize accessions at a prevalence threshold ≥ 50% across samples and 70% of host accessions. The color scale bar (1–4) represents the count of pollen sample replicates within each maize accession. Along the x-axis, the maize accession name is noted, followed by the number of replicates, then the count of Ps. rhizosphareae taxa, followed by a code (a number followed by M) where the number represents the placement of the maize accession on the phylogenetic tree of Matsuoka et al. (2002) constructed based on microsatellite data (for the number, see supporting information, Figure 4B and Table 1 in Matsuoka et al. (2002). The X means the maize accession was not included in the Matsuoka study. [file Image_8.JPEG]

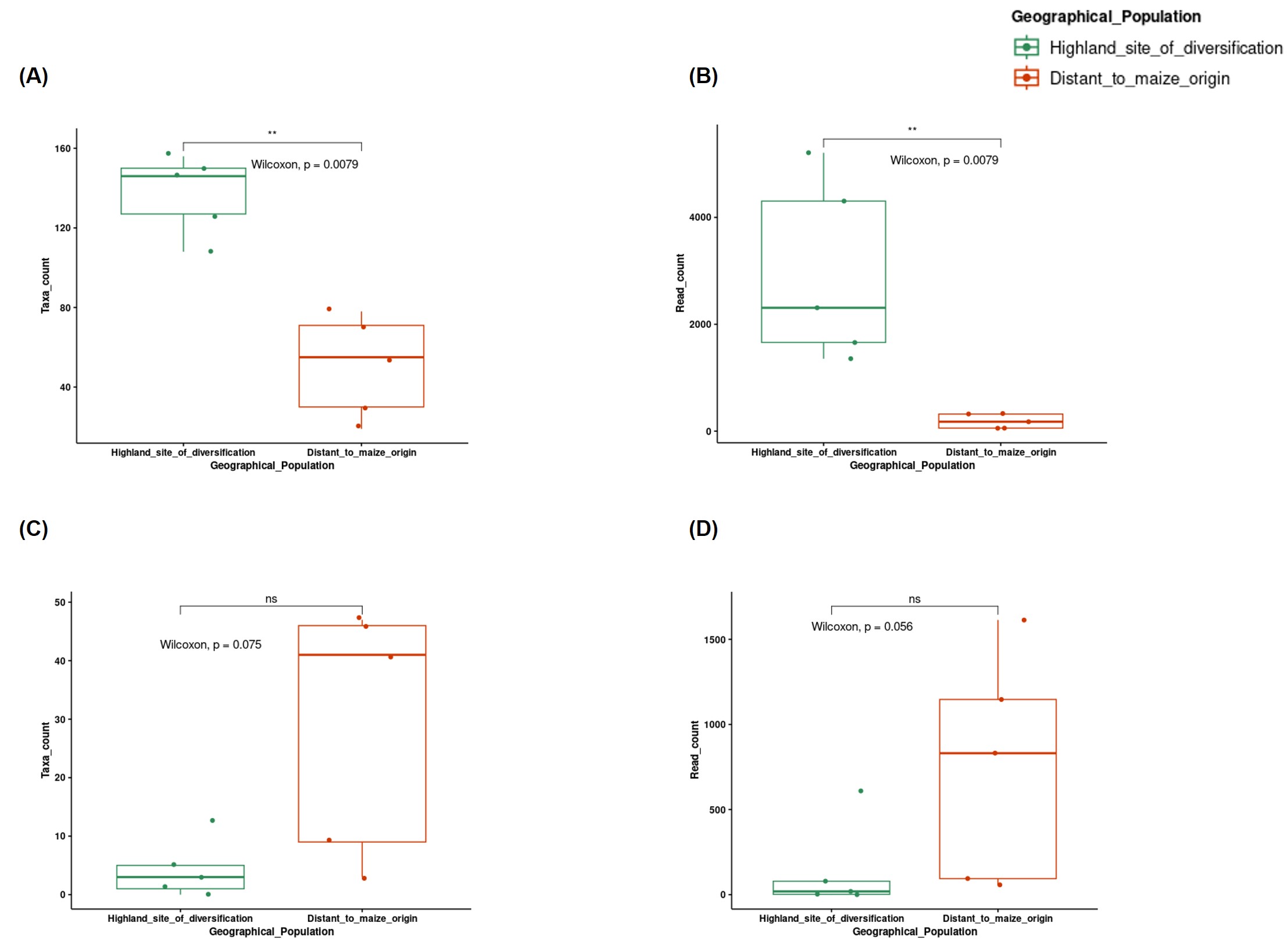

Supplement: Supplementary Figure S9 — Statistical comparisons of select core members of the pollen microbiome of maize landraces clustered by their geographical origin of diversification. The statistical comparisons were performed on the taxa and read counts of P. ananatis and L. lactis which were the most prevalent and diverse bacterial species identified by FL-PacBio. The values of each microbiome parameter are displayed as boxplots where each dot represents a unique landrace. The mean of the two geographic populations is compared using the Mann-Whitney U / Wilcoxon Rank Sum Test. Shown are (A) P. ananatis taxa count, (B) P. ananatis read count, (C) L. lactis taxa count, (D) L. lactis read count. [file Image_9.jpg]
