# Supplementary material for: Conservation and diversity of the pollen microbiome of Pan-American maize using PacBio and MiSeq
Source: Front Microbiol. 2023 Dec 21;14:1276241. doi: 10.3389/fmicb.2023.1276241 (PMC10764481; doi:10.3389/fmicb.2023.1276241)
Supplement: Supplementary file 10 [file Table_3.PDF]

Table S2. Weather data of the field trial

Table S2A. Hourly recorded weather data of the field trial in July 2019

Longitude	Latitude (	Station Na	Climate ID	Date/Time	Year	Month	Day	Time (LST	Temp (°C)	Temp Flag	Dew Point	Dew Point	Rel Hum (	Rel Hum F	Precip. Am	Precip. Am	Wind Dir (	Wind Dir I	Wind Spd	Wind Spd	Visibility (	Visibility F	Stn Press (	Stn Press	Hmdx	Hmdx Flag	Wind Chill	Wind Chill	Weather
-80.42	43.65	ELORA RC	6142286	43647	2019	7	1	0	13		11.2		89		0				0				97.32						NA
-80.42	43.65	ELORA RC	6142286	43647	2019	7	1	0.04167	11.9		10.9		94		0		29		4				97.31						NA
-80.42	43.65	ELORA RC	6142286	43647.1	2019	7	1	0.08333	11.2		10.3		94		0				0				97.25						NA
-80.42	43.65	ELORA RC	6142286	43647.1	2019	7	1	0.125	10.7		9.9		95		0				0				97.19						NA
-80.42	43.65	ELORA RC	6142286	43647.2	2019	7	1	0.16667	10.2		9.5		95		0		33		6				97.22						NA
-80.42	43.65	ELORA RC	6142286	43647.2	2019	7	1	0.20833	10.6		9.6		93		0		36		4				97.29						NA
-80.42	43.65	ELORA RC	6142286	43647.3	2019	7	1	0.25	13.6		11.6		88		1.7				0				97.28						NA
-80.42	43.65	ELORA RC	6142286	43647.3	2019	7	1	0.29167	17.3		13		76		0		11		3				97.32						NA
-80.42	43.65	ELORA RC	6142286	43647.3	2019	7	1	0.33333	18.7		13.5		72		0		14		6				97.28						NA
-80.42	43.65	ELORA RC	6142286	43647.4	2019	7	1	0.375	20		13.1		64		0		22		12				97.29						NA
-80.42	43.65	ELORA RC	6142286	43647.4	2019	7	1	0.41667	21.2		14.2		64		0		23		10				97.31		25				NA
-80.42	43.65	ELORA RC	6142286	43647.5	2019	7	1	0.45833	22.7		12.9		54		0		23		11				97.27		25				NA
-80.42	43.65	ELORA RC	6142286	43647.5	2019	7	1	0.5	23.6		13.5		53		0		25		12				97.2		27				NA
-80.42	43.65	ELORA RC	6142286	43647.5	2019	7	1	0.54167	24.2		14.1		53		0		24		13				97.16		28				NA
-80.42	43.65	ELORA RC	6142286	43647.6	2019	7	1	0.58333	25.1		16.4		59		0		25		13				97.09		30				NA
-80.42	43.65	ELORA RC	6142286	43647.6	2019	7	1	0.625	25.2		16.2		58		0		24		11				97.02		30				NA
-80.42	43.65	ELORA RC	6142286	43647.7	2019	7	1	0.66667	26.2		15.6		52		0		24		16				96.97		31				NA
-80.42	43.65	ELORA RC	6142286	43647.7	2019	7	1	0.70833	26.8		16		52		0		24		18				96.89		31				NA
-80.42	43.65	ELORA RC	6142286	43647.8	2019	7	1	0.75	26.6		16.2		53		0		24		12				96.85		31				NA
-80.42	43.65	ELORA RC	6142286	43647.8	2019	7	1	0.79167	25.1		16.8		60		0		22		18				96.87		30				NA
-80.42	43.65	ELORA RC	6142286	43647.8	2019	7	1	0.83333	23		16.5		67		0		22		12				96.88		28				NA
-80.42	43.65	ELORA RC	6142286	43647.9	2019	7	1	0.875	21.3		16.4		74		0		22		7				96.9		26				NA
-80.42	43.65	ELORA RC	6142286	43647.9	2019	7	1	0.91667	21.4		16.2		72		0		24		5				96.88		26				NA
-80.42	43.65	ELORA RC	6142286	43648	2019	7	1	0.95833	20.5		16.2		76		0		27		6				96.86		25				NA
-80.42	43.65	ELORA RC	6142286	43648	2019	7	2	0	20.1		16.1		78		0		28		5				96.84		25				NA
-80.42	43.65	ELORA RC	6142286	43648	2019	7	2	0.04167	19.9		16		78		0		27		9				96.81						NA
-80.42	43.65	ELORA RC	6142286	43648.1	2019	7	2	0.08333	19.4		16.6		84		0		25		9				96.77						NA
-80.42	43.65	ELORA RC	6142286	43648.1	2019	7	2	0.125	19.3		17.1		87		0		29		7				96.77						NA
-80.42	43.65	ELORA RC	6142286	43648.2	2019	7	2	0.16667	19.3		17.4		89		0		35		4				96.75						NA
-80.42	43.65	ELORA RC	6142286	43648.2	2019	7	2	0.20833	19.6		17.2		86		0		32		9				96.78						NA
-80.42	43.65	ELORA RC	6142286	43648.3	2019	7	2	0.25	19.4		17.6		90		0		27		9				96.79						NA
-80.42	43.65	ELORA RC	6142286	43648.3	2019	7	2	0.29167	20.2		17.6		85		0		30		6				96.77		26				NA
-80.42	43.65	ELORA RC	6142286	43648.3	2019	7	2	0.33333	21.4		17.9		81		0		29		10				96.81		27				NA
-80.42	43.65	ELORA RC	6142286	43648.4	2019	7	2	0.375	21.5		17.2		76		0		28		10				96.82		27				NA
-80.42	43.65	ELORA RC	6142286	43648.4	2019	7	2	0.41667	22.2		17.7		76		0		29		13				96.85		28				NA
-80.42	43.65	ELORA RC	6142286	43648.5	2019	7	2	0.45833	21.9		18.3		80		0		30		11				96.9		28				NA
-80.42	43.65	ELORA RC	6142286	43648.5	2019	7	2	0.5	21.8		19.6		88		0		30		11				96.88		29				NA
-80.42	43.65	ELORA RC	6142286	43648.5	2019	7	2	0.54167	21.9		19.4		86		0.7		28		11				96.81		29				NA
-80.42	43.65	ELORA RC	6142286	43648.6	2019	7	2	0.58333	22.6		19.6		83		0		29		12				96.8		30				NA
-80.42	43.65	ELORA RC	6142286	43648.6	2019	7	2	0.625	23.6		19.4		77		0		29		17				96.76		31				NA
-80.42	43.65	ELORA RC	6142286	43648.7	2019	7	2	0.66667	23.2		19.1		78		0		29		15				96.71		30				NA
-80.42	43.65	ELORA RC	6142286	43648.7	2019	7	2	0.70833	22.1		19.9		88		0		32		10				96.69		30				NA
-80.42	43.65	ELORA RC	6142286	43648.8	2019	7	2	0.75	22		19.4		85		0		33		10				96.65		29				NA
-80.42	43.65	ELORA RC	6142286	43648.8	2019	7	2	0.79167	21.5		19.2		87		0		36		6				96.68		28				NA
-80.42	43.65	ELORA RC	6142286	43648.8	2019	7	2	0.83333	20.5		18.8		90		0		32		5				96.63		27				NA
-80.42	43.65	ELORA RC	6142286	43648.9	2019	7	2	0.875	19.7		18.6		93		0		34		3				96.67						NA
-80.42	43.65	ELORA RC	6142286	43648.9	2019	7	2	0.91667	18.3		17.6		96		0		26		4				96.71						NA
-80.42	43.65	ELORA RC	6142286	43649	2019	7	2	0.95833	17.9		17.3		96		0		31		3				96.74						NA
-80.42	43.65	ELORA RC	6142286	43649	2019	7	3	0	18		17.4		97		0				0				96.75						NA
-80.42	43.65	ELORA RC	6142286	43649	2019	7	3	0.04167	16.8		16.2		97		0.8		30		3				96.73						NA
-80.42	43.65	ELORA RC	6142286	43649.1	2019	7	3	0.08333	17.3		16.8		97		0		1		5				96.74						NA
-80.42	43.65	ELORA RC	6142286	43649.1	2019	7	3	0.125	17.3		16.8		97		0		1		8				96.73						NA
-80.42	43.65	ELORA RC	6142286	43649.2	2019	7	3	0.16667	17.9		17.5		97		0		36		9				96.78						NA
-80.42	43.65	ELORA RC	6142286	43649.2	2019	7	3	0.20833	18		17.6		97		0		35		6				96.82						NA
-80.42	43.65	ELORA RC	6142286	43649.3	2019	7	3	0.25	18.1		17.7		97		0		31		7				96.89						NA
-80.42	43.65	ELORA RC	6142286	43649.3	2019	7	3	0.29167	18.4		18		97		0		34		5				96.93						NA
-80.42	43.65	ELORA RC	6142286	43649.3	2019	7	3	0.33333	19.6		19.1		97		0		1		6				96.94						NA
-80.42	43.65	ELORA RC	6142286	43649.4	2019	7	3	0.375	21.7		20.7		94		0		32		3				96.95		30				NA
-80.42	43.65	ELORA RC	6142286	43649.4	2019	7	3	0.41667	23.5		19.6		79		0		1		3				96.96		31				NA
-80.42	43.65	ELORA RC	6142286	43649.5	2019	7	3	0.45833	24		19.3		75		0		18		5				96.98		31				NA
-80.42	43.65	ELORA RC	6142286	43649.5	2019	7	3	0.5	25.6		18.8		66		0		11		7				96.99		32				NA
-80.42	43.65	ELORA RC	6142286	43649.5	2019	7	3	0.54167	26		19.2		66		0		32		4				96.98		33				NA
-80.42	43.65	ELORA RC	6142286	43649.6	2019	7	3	0.58333	27.5		18.6		58		0		33		6				96.93		34				NA
-80.42	43.65	ELORA RC	6142286	43649.6	2019	7	3	0.625	27.3		17.6		56		0		26		7				96.95		33				NA
-80.42	43.65	ELORA RC	6142286	43649.7	2019	7	3	0.66667	28		15.7		47		0		22		3				96.92		32				NA
-80.42	43.65	ELORA RC	6142286	43649.7	2019	7	3	0.70833	28.6		15.1		44		0		34		9				96.92		33				NA
-80.42	43.65	ELORA RC	6142286	43649.8	2019	7	3	0.75	26.7		17.8		58		0		10		12				96.92		33				NA
-80.42	43.65	ELORA RC	6142286	43649.8	2019	7	3	0.79167	25.8		19.2		67		0		14		8				96.96		33				NA
-80.42	43.65	ELORA RC	6142286	43649.8	2019	7	3	0.83333	24.3		18.7		71		0		13		7				97.02		31				NA
-80.42	43.65	ELORA RC	6142286	43649.9	2019	7	3	0.875	22		18.7		82		0		8		4				97.11		29				NA
-80.42	43.65	ELORA RC	6142286	43649.9	2019	7	3	0.91667	21.4		17.9		80		0		3		4				97.16		27				NA
-80.42	43.65	ELORA RC	6142286	43650	2019	7	3	0.95833	20.5		16.5		78		0		5		8				97.21		25				NA
-80.42	43.65	ELORA RC	6142286	43650	2019	7	4	0	18.9		16.8		88		0		6		8				97.22						NA
-80.42	43.65	ELORA RC	6142286	43650	2019	7	4	0.04167	18.3		15.3		83		0		8		10				97.23						NA
-80.42	43.65	ELORA RC	6142286	43650.1	2019	7	4	0.08333	17.6		12.4		72		0		7		8				97.25						NA
-80.42	43.65	ELORA RC	6142286	43650.1	2019	7	4	0.125	16.4		11.1		71		0		6		8				97.25						NA
-80.42	43.65	ELORA RC	6142286	43650.2	2019	7	4	0.16667	16.1		11.3		73		0		6		8				97.25						NA
-80.42	43.65	ELORA RC	6142286	43650.2	2019	7	4	0.20833	15.8		11.7		77		0		7		8				97.26						NA
-80.42	43.65	ELORA RC	6142286	43650.3	2019	7	4	0.25	17.9		13.7		77		1		7		4				97.34						NA
-80.42	43.65	ELORA RC	6142286	43650.3	2019	7	4	0.29167	21.5		15.2		67		0		9		5				97.4		26				NA
-80.42	43.65	ELORA RC	6142286	43650.3	2019	7	4	0.33333	23.3		16.4		65		0		10		8				97.39		28				NA
-80.42	43.65	ELORA RC	6142286	43650.4	2019	7	4	0.375	24.8		17.2		63		0		13		12				97.37		30				NA
-80.42	43.65	ELORA RC	6142286	43650.4	2019	7	4	0.41667	26.1		19.2		65		0		10		11				97.4		33				NA
-80.42	43.65	ELORA RC	6142286	43650.5	2019	7	4	0.45833	26.9		18.9		61		0		11		10				97.39		34				NA
-80.42	43.65	ELORA RC	6142286	43650.5	2019	7	4	0.5	27.5		20.3		65		0		11		9				97.37		35				NA
-80.42	43.65	ELORA RC	6142286	43650.5	2019	7	4	0.54167	27.8		20.3		64		0		11		9				97.33		36				NA
-80.42	43.65	ELORA RC	6142286	43650.6	2019	7	4	0.58333	28.5		21		64		0		11		9				97.27		37				NA
-80.42	43.65	ELORA RC	6142286	43650.6	2019	7	4	0.625	28.2		19.1		58		0		15		8				97.24		35				NA
-80.42	43.65	ELORA RC	6142286	43650.7	2019	7	4	0.66667	29.1		19.8		57		0		13		9				97.25		37				NA
-80.42	43.65	ELORA RC	6142286	43650.7	2019	7	4	0.70833	28.5		19.1		57		0		13		5				97.21		35				NA
-80.42	43.65	ELORA RC	6142286	43650.8	2019	7	4	0.75	27.2		21.4		71		0		11		12				97.18		36				NA
-80.42	43.65	ELORA RC	6142286	43650.8	2019	7	4	0.79167	26.4		20.9		72		0		11		13				97.23		35				NA
-80.42	43.65	ELORA RC	6142286	43650.8	2019	7	4	0.83333	24.4		20		76		0		11		10				97.32		32				NA
-80.42	43.65	ELORA RC	6142286	43650.9	2019	7	4	0.875	23.1		19.7		81		0		11		6				97.35		30				NA
-80.42	43.65	ELORA RC	6142286	43650.9	2019	7	4	0.91667	22		19.1		84		0		10		8				97.36		29				NA
-80.42	43.65	ELORA RC	6142286	43651	2019	7	4	0.95833	21.1		18.9		87		0		5		3				97.39		28				NA
-80.42	43.65	ELORA RC	6142286	43651	2019	7	5	0	20		18.5		91		1.2		6		3				97.38		26				NA
-80.42	43.65	ELORA RC	6142286	43651	2019	7	5	0.04167	20.6		18.6		89		0		1		6				97.34		27				NA
-80.42	43.65	ELORA RC	6142286	43651.1	2019	7	5	0.08333	19.6		18.4		93		0				0				97.32						NA
-80.42	43.65	ELORA RC	6142286	43651.1	2019	7	5	0.125	18.9		17.9		94		0				0				97.32						NA
-80.42	43.65	ELORA RC	6142286	43651.2	2019	7	5	0.16667	18.4		17.4		94		0		9		4				97.31						NA
-80.42	43.65	ELORA RC	6142286	43651.2	2019	7	5	0.20833	18.2		17.3		95		0		11		4				97.33						NA
-80.42	43.65	ELORA RC	6142286	43651.3	2019	7	5	0.25	19.5		18.7		95		0.9		2		4				97.34						NA
-80.42	43.65	ELORA RC	6142286	43651.3	2019	7	5	0.29167	23		20.7		87		0				0				97.38		31				NA
-80.42	43.65	ELORA RC	6142286	43651.3	2019	7	5	0.33333	24.8		21.8		83		0		23		9				97.4		34				NA
-80.42	43.65	ELORA RC	6142286	43651.4	2019	7	5	0.375	26.2		21.8		77		0		24		10				97.38		35				NA
-80.42	43.65	ELORA RC	6142286	43651.4	2019	7	5	0.41667	28.2		21.9		69		0		25		12				97.36		37				NA
-80.42	43.65	ELORA RC	6142286	43651.5	2019	7	5	0.45833	28.6		21.4		65		0		25		15				97.36		37				NA
-80.42	43.65	ELORA RC	6142286	43651.5	2019	7	5	0.5	28.6		21.4		65		0		26		13				97.33		37				NA
-80.42	43.65	ELORA RC	6142286	43651.5	2019	7	5	0.54167	30.1		20.8		57		0		24		19				97.26		38				NA
-80.42	43.65	ELORA RC	6142286	43651.6	2019	7	5	0.58333	30.6		20.8		56		0		24		11				97.18		39				NA
-80.42	43.65	ELORA RC	6142286	43651.6	2019	7	5	0.625	30.8		20.2		53		0		27		15				97.18		39				NA
-80.42	43.65	ELORA RC	6142286	43651.7	2019	7	5	0.66667	31.2		20.4		53		0		27		20				97.14		39				NA
-80.42	43.65	ELORA RC	6142286	43651.7	2019	7	5	0.70833	30.8		20.7		55		0		27		14				97.06		39				NA
-80.42	43.65	ELORA RC	6142286	43651.8	2019	7	5	0.75	28.9		20.1		59		0		30		13				97.08		37				NA
-80.42	43.65	ELORA RC	6142286	43651.8	2019	7	5	0.79167	26.9		21.4		72		0		19		8				97.13		36				NA
-80.42	43.65	ELORA RC	6142286	43651.8	2019	7	5	0.83333	25.6		20.8		75		0		22		18				97.15		34				NA
-80.42	43.65	ELORA RC	6142286	43651.9	2019	7	5	0.875	23.6		20.2		82		0		17		9				97.18		31				NA
-80.42	43.65	ELORA RC	6142286	43651.9	2019	7	5	0.91667	23.1		20.5		85		0		18		8				97.19		31				NA
-80.42	43.65	ELORA RC	6142286	43652	2019	7	5	0.95833	22.5		20.5		89		0		23		7				97.16		30				NA
-80.42	43.65	ELORA RC	6142286	43652	2019	7	6	0	22.4		20.3		88		0		25		7				97.16		30				NA
-80.42	43.65	ELORA RC	6142286	43652	2019	7	6	0.04167	22.5		20.5		89		0		27		9				97.12		30				NA
-80.42	43.65	ELORA RC	6142286	43652.1	2019	7	6	0.08333	22.1		20.8		92		0		25		4				97.09		30				NA
-80.42	43.65	ELORA RC	6142286	43652.1	2019	7	6	0.125	22		20.7		93		0				0				97.06		30				NA
-80.42	43.65	ELORA RC	6142286	43652.2	2019	7	6	0.16667	21.1		20.3		95		0		27		4				97.02		29				NA
-80.42	43.65	ELORA RC	6142286	43652.2	2019	7	6	0.20833	20.6		19.9		96		0		26		4				97.05		28				NA
-80.42	43.65	ELORA RC	6142286	43652.3	2019	7	6	0.25	21.6		20.8		96		0		29		3				97.09		30				NA
-80.42	43.65	ELORA RC	6142286	43652.3	2019	7	6	0.29167	22.4		20.4		88		0		30		3				97.11		30				NA
-80.42	43.65	ELORA RC	6142286	43652.3	2019	7	6	0.33333	24.3		21.2		83		0		32		6				97.12		33				NA
-80.42	43.65	ELORA RC	6142286	43652.4	2019	7	6	0.375	26.1		20.7		73		0		31		6				97.1		34				NA
-80.42	43.65	ELORA RC	6142286	43652.4	2019	7	6	0.41667	26.3		20.2		69		0		31		13				97.11		34				NA
-80.42	43.65	ELORA RC	6142286	43652.5	2019	7	6	0.45833	27		20		66		0		35		9				97.07		35				NA
-80.42	43.65	ELORA RC	6142286	43652.5	2019	7	6	0.5	27.7		20.1		63		0		34		12				97.06		35				NA

-80.42	43.65	ELORA RC	6142286	43652.5	2019	7	6	0.54167	28		20.5		63		0		35		9				97		36				NA
-80.42	43.65	ELORA RC	6142286	43652.6	2019	7	6	0.58333	28.9		20.4		60		0		36		13				96.95		37				NA
-80.42	43.65	ELORA RC	6142286	43652.6	2019	7	6	0.625	28.3		20.2		62		0		4		15				96.94		36				NA
-80.42	43.65	ELORA RC	6142286	43652.7	2019	7	6	0.66667	27.4		19.2		61		0		4		17				96.94		34				NA
-80.42	43.65	ELORA RC	6142286	43652.7	2019	7	6	0.70833	27.6		18.8		59		0		3		17				96.93		34				NA
-80.42	43.65	ELORA RC	6142286	43652.8	2019	7	6	0.75	26.8		18.4		60		0		3		20				96.96		33				NA
-80.42	43.65	ELORA RC	6142286	43652.8	2019	7	6	0.79167	25.3		18.4		65		0		1		18				97		32				NA
-80.42	43.65	ELORA RC	6142286	43652.8	2019	7	6	0.83333	23.7		17.1		66		0		2		15				97.07		29				NA
-80.42	43.65	ELORA RC	6142286	43652.9	2019	7	6	0.875	22.7		15.6		64		0		3		19				97.11		27				NA
-80.42	43.65	ELORA RC	6142286	43652.9	2019	7	6	0.91667	21.5		15.1		67		0		3		16				97.16		26				NA
-80.42	43.65	ELORA RC	6142286	43653	2019	7	6	0.95833	21.4		14.2		64		0		4		16				97.2		25				NA
-80.42	43.65	ELORA RC	6142286	43653	2019	7	7	0	20.9		13.8		64		0		4		17				97.2						NA
-80.42	43.65	ELORA RC	6142286	43653	2019	7	7	0.04167	20.3		13.3		64		0		5		17				97.2						NA
-80.42	43.65	ELORA RC	6142286	43653.1	2019	7	7	0.08333	19.6		12.8		65		0		5		20				97.19						NA
-80.42	43.65	ELORA RC	6142286	43653.1	2019	7	7	0.125	19.2		12.2		64		0		6		16				97.22						NA
-80.42	43.65	ELORA RC	6142286	43653.2	2019	7	7	0.16667	18.7		12.1		66		0		7		18				97.26						NA
-80.42	43.65	ELORA RC	6142286	43653.2	2019	7	7	0.20833	17.6		12.4		72		0		6		12				97.3						NA
-80.42	43.65	ELORA RC	6142286	43653.3	2019	7	7	0.25	17.3		12.5		74		0		6		10				97.34						NA
-80.42	43.65	ELORA RC	6142286	43653.3	2019	7	7	0.29167	18		11.4		65		0		6		17				97.34						NA
-80.42	43.65	ELORA RC	6142286	43653.3	2019	7	7	0.33333	19.2		10.2		56		0		7		19				97.37						NA
-80.42	43.65	ELORA RC	6142286	43653.4	2019	7	7	0.375	19.8		10.6		55		0		7		19				97.39						NA
-80.42	43.65	ELORA RC	6142286	43653.4	2019	7	7	0.41667	20.8		11.2		54		0		8		16				97.39						NA
-80.42	43.65	ELORA RC	6142286	43653.5	2019	7	7	0.45833	21.9		12.2		54		0		8		16				97.39						NA
-80.42	43.65	ELORA RC	6142286	43653.5	2019	7	7	0.5	22.5		12.3		53		0		8		12				97.36		25				NA
-80.42	43.65	ELORA RC	6142286	43653.5	2019	7	7	0.54167	23.3		11.5		47		0		10		11				97.34		25				NA
-80.42	43.65	ELORA RC	6142286	43653.6	2019	7	7	0.58333	23.9		11.6		46		0		8		11				97.27		26				NA
-80.42	43.65	ELORA RC	6142286	43653.6	2019	7	7	0.625	24.5		13.1		49		0		7		11				97.22		27				NA
-80.42	43.65	ELORA RC	6142286	43653.7	2019	7	7	0.66667	24.1		11.6		46		0		4		11				97.2		26				NA
-80.42	43.65	ELORA RC	6142286	43653.7	2019	7	7	0.70833	23.6		12.1		49		0		4		8				97.19		26				NA
-80.42	43.65	ELORA RC	6142286	43653.8	2019	7	7	0.75	22.2		11.6		51		0		6		15				97.21						NA
-80.42	43.65	ELORA RC	6142286	43653.8	2019	7	7	0.79167	20.7		11.3		55		0		10		8				97.23						NA
-80.42	43.65	ELORA RC	6142286	43653.8	2019	7	7	0.83333	18.8		12.2		66		0		11		7				97.25						NA
-80.42	43.65	ELORA RC	6142286	43653.9	2019	7	7	0.875	15.9		12		78		0		7		6				97.29						NA
-80.42	43.65	ELORA RC	6142286	43653.9	2019	7	7	0.91667	15.2		11.8		81		0		2		4				97.33						NA
-80.42	43.65	ELORA RC	6142286	43654	2019	7	7	0.95833	14.4		10.9		80		2.4		2		3				97.31						NA
-80.42	43.65	ELORA RC	6142286	43654	2019	7	8	0	12.9		10.9		88		0		32		5				97.32						NA
-80.42	43.65	ELORA RC	6142286	43654	2019	7	8	0.04167	12.4		10.3		87		0		3		5				97.28						NA
-80.42	43.65	ELORA RC	6142286	43654.1	2019	7	8	0.08333	12.2		9.5		84		0		1		4				97.27						NA
-80.42	43.65	ELORA RC	6142286	43654.1	2019	7	8	0.125	11.7		8.6		81		0		1		3				97.26						NA
-80.42	43.65	ELORA RC	6142286	43654.2	2019	7	8	0.16667	10.6		8.5		87		0				0				97.28						NA
-80.42	43.65	ELORA RC	6142286	43654.2	2019	7	8	0.20833	10.1		8.5		90		0				0				97.32						NA
-80.42	43.65	ELORA RC	6142286	43654.3	2019	7	8	0.25	11.6		9.7		88		0				0				97.36						NA
-80.42	43.65	ELORA RC	6142286	43654.3	2019	7	8	0.29167	14.7		10.6		76		2.4		3		5				97.42						NA
-80.42	43.65	ELORA RC	6142286	43654.3	2019	7	8	0.33333	18.6		12.4		67		0		5		5				97.43						NA
-80.42	43.65	ELORA RC	6142286	43654.4	2019	7	8	0.375	20		11.4		57		0		2		7				97.45						NA
-80.42	43.65	ELORA RC	6142286	43654.4	2019	7	8	0.41667	21.6		11		51		0		3		9				97.39						NA
-80.42	43.65	ELORA RC	6142286	43654.5	2019	7	8	0.45833	22.7		11.3		48		0		7		5				97.37		25				NA
-80.42	43.65	ELORA RC	6142286	43654.5	2019	7	8	0.5	23.3		10.9		46		0		8		3				97.38		25				NA
-80.42	43.65	ELORA RC	6142286	43654.5	2019	7	8	0.54167	23.8		10.9		44		0		23		7				97.35		26				NA
-80.42	43.65	ELORA RC	6142286	43654.6	2019	7	8	0.58333	24.1		8.2		36		0		4		3				97.33		25				NA
-80.42	43.65	ELORA RC	6142286	43654.6	2019	7	8	0.625	24.4		8.7		37		0		34		7				97.35		25				NA
-80.42	43.65	ELORA RC	6142286	43654.7	2019	7	8	0.66667	25.4		11.3		41		0		24		3				97.33		27				NA
-80.42	43.65	ELORA RC	6142286	43654.7	2019	7	8	0.70833	25.2		11.5		42		0		31		8				97.3		27				NA
-80.42	43.65	ELORA RC	6142286	43654.8	2019	7	8	0.75	24		12		47		0		31		7				97.31		26				NA
-80.42	43.65	ELORA RC	6142286	43654.8	2019	7	8	0.79167	21.9		12.5		55		0		32		11				97.34						NA
-80.42	43.65	ELORA RC	6142286	43654.8	2019	7	8	0.83333	20.4		12.5		60		0		36		7				97.35						NA
-80.42	43.65	ELORA RC	6142286	43654.9	2019	7	8	0.875	18.7		11.7		64		0		2		10				97.37						NA
-80.42	43.65	ELORA RC	6142286	43654.9	2019	7	8	0.91667	17.1		12.4		74		0		10		4				97.35						NA
-80.42	43.65	ELORA RC	6142286	43655	2019	7	8	0.95833	15.3		11.4		78		0		4		7				97.36						NA
-80.42	43.65	ELORA RC	6142286	43655	2019	7	9	0	14.4		10.8		79		0		4		7				97.43						NA
-80.42	43.65	ELORA RC	6142286	43655	2019	7	9	0.04167	13.4		10.4		82		0		5		7				97.43						NA
-80.42	43.65	ELORA RC	6142286	43655.1	2019	7	9	0.08333	14.9		9.7		71		1.6		2		10				97.45						NA
-80.42	43.65	ELORA RC	6142286	43655.1	2019	7	9	0.125	13		10		82		0		4		7				97.46						NA
-80.42	43.65	ELORA RC	6142286	43655.2	2019	7	9	0.16667	13.3		9.8		79		0		3		6				97.49						NA
-80.42	43.65	ELORA RC	6142286	43655.2	2019	7	9	0.20833	13.1		10		81		0		2		5				97.54						NA
-80.42	43.65	ELORA RC	6142286	43655.3	2019	7	9	0.25	13.7		10.7		83		0.8		2		5				97.58						NA
-80.42	43.65	ELORA RC	6142286	43655.3	2019	7	9	0.29167	15.9		12.1		78		0.6		6		4				97.57						NA
-80.42	43.65	ELORA RC	6142286	43655.3	2019	7	9	0.33333	19		12.2		65		0		10		5				97.59						NA
-80.42	43.65	ELORA RC	6142286	43655.4	2019	7	9	0.375	20.6		12.2		59		0		16		5				97.57						NA
-80.42	43.65	ELORA RC	6142286	43655.4	2019	7	9	0.41667	21.9		12.3		54		0		19		5				97.53						NA
-80.42	43.65	ELORA RC	6142286	43655.5	2019	7	9	0.45833	22.9		11.7		49		0		22		7				97.53		25				NA
-80.42	43.65	ELORA RC	6142286	43655.5	2019	7	9	0.5	24		12.4		48		0		19		7				97.52		26				NA
-80.42	43.65	ELORA RC	6142286	43655.5	2019	7	9	0.54167	24.4		10.9		43		0		16		5				97.49		26				NA
-80.42	43.65	ELORA RC	6142286	43655.6	2019	7	9	0.58333	25		11.6		43		0		10		7				97.41		27				NA
-80.42	43.65	ELORA RC	6142286	43655.6	2019	7	9	0.625	25.6		11.4		41		0		20		6				97.4		28				NA
-80.42	43.65	ELORA RC	6142286	43655.7	2019	7	9	0.66667	26.1		13.4		46		0		16		5				97.37		29				NA
-80.42	43.65	ELORA RC	6142286	43655.7	2019	7	9	0.70833	25.8		10.3		38		0		15		4				97.36		27				NA
-80.42	43.65	ELORA RC	6142286	43655.8	2019	7	9	0.75	25.1		12.8		46		0		18		8				97.36		28				NA
-80.42	43.65	ELORA RC	6142286	43655.8	2019	7	9	0.79167	23.5		14.4		57		0		16		7				97.35		27				NA
-80.42	43.65	ELORA RC	6142286	43655.8	2019	7	9	0.83333	21.6		13.9		62		0		19		7				97.36		25				NA
-80.42	43.65	ELORA RC	6142286	43655.9	2019	7	9	0.875	20		13		64		1.8		13		6				97.36						NA
-80.42	43.65	ELORA RC	6142286	43655.9	2019	7	9	0.91667	17		12.9		77		0				0				97.37						NA
-80.42	43.65	ELORA RC	6142286	43656	2019	7	9	0.95833	15.8		12.8		82		0		6		4				97.34						NA
-80.42	43.65	ELORA RC	6142286	43656	2019	7	10	0	14.7		12.1		84		0		2		3				97.35						NA
-80.42	43.65	ELORA RC	6142286	43656	2019	7	10	0.04167	12.9		11.2		89		0		6		5				97.31						NA
-80.42	43.65	ELORA RC	6142286	43656.1	2019	7	10	0.08333	12.8		11.3		90		0		5		3				97.29						NA
-80.42	43.65	ELORA RC	6142286	43656.1	2019	7	10	0.125	13.5		11.9		90		1.4		4		4				97.26						NA
-80.42	43.65	ELORA RC	6142286	43656.2	2019	7	10	0.16667	12.8		11.6		92		0		6		5				97.27						NA
-80.42	43.65	ELORA RC	6142286	43656.2	2019	7	10	0.20833	11.9		10.6		92		0		35		4				97.31						NA
-80.42	43.65	ELORA RC	6142286	43656.3	2019	7	10	0.25	14.8		13.3		91		0		6		9				97.29						NA
-80.42	43.65	ELORA RC	6142286	43656.3	2019	7	10	0.29167	18.9		15.6		81		0.6		6		4				97.3						NA
-80.42	43.65	ELORA RC	6142286	43656.3	2019	7	10	0.33333	22		15.2		65		0		13		5				97.29		26				NA
-80.42	43.65	ELORA RC	6142286	43656.4	2019	7	10	0.375	22.8		14.4		59		0		16		9				97.27		26				NA
-80.42	43.65	ELORA RC	6142286	43656.4	2019	7	10	0.41667	24.4		15		56		0		19		7				97.24		28				NA
-80.42	43.65	ELORA RC	6142286	43656.5	2019	7	10	0.45833	26.1		17.1		58		0		24		8				97.2		31				NA
-80.42	43.65	ELORA RC	6142286	43656.5	2019	7	10	0.5	27.4		16.6		52		0		20		13				97.15		32				NA
-80.42	43.65	ELORA RC	6142286	43656.5	2019	7	10	0.54167	28.5		16.1		47		0		21		14				97.09		33				NA
-80.42	43.65	ELORA RC	6142286	43656.6	2019	7	10	0.58333	29.1		16.6		47		0		21		12				97.04		34				NA
-80.42	43.65	ELORA RC	6142286	43656.6	2019	7	10	0.625	29.3		14.3		40		0		20		17				97.02		33				NA
-80.42	43.65	ELORA RC	6142286	43656.7	2019	7	10	0.66667	29.2		15.6		44		0		22		15				96.98		34				NA
-80.42	43.65	ELORA RC	6142286	43656.7	2019	7	10	0.70833	29		16.9		48		0		19		15				96.92		34				NA
-80.42	43.65	ELORA RC	6142286	43656.8	2019	7	10	0.75	27.7		16.8		52		0		21		12				96.92		33				NA
-80.42	43.65	ELORA RC	6142286	43656.8	2019	7	10	0.79167	26.4		17		56		0		23		13				96.93		32				NA
-80.42	43.65	ELORA RC	6142286	43656.8	2019	7	10	0.83333	22.1		19.8		87		0		30		18				96.92		30				NA
-80.42	43.65	ELORA RC	6142286	43656.9	2019	7	10	0.875	21.6		20		91		0		11		6				96.89		29				NA
-80.42	43.65	ELORA RC	6142286	43656.9	2019	7	10	0.91667	21		19.8		93		0		14		9				96.84		28				NA
-80.42	43.65	ELORA RC	6142286	43657	2019	7	10	0.95833	21.2		19.6		91		0		12		6				96.79		28				NA
-80.42	43.65	ELORA RC	6142286	43657	2019	7	11	0	21.1		19.5		91		0		16		5				96.78		28				NA
-80.42	43.65	ELORA RC	6142286	43657	2019	7	11	0.04167	20.4		19.1		93		0		16		4				96.72		27				NA
-80.42	43.65	ELORA RC	6142286	43657.1	2019	7	11	0.08333	19.8		18.7		94		0		18		4				96.68						NA
-80.42	43.65	ELORA RC	6142286	43657.1	2019	7	11	0.125	19.6		18.5		93		0		15		4				96.65						NA
-80.42	43.65	ELORA RC	6142286	43657.2	2019	7	11	0.16667	19.7		18.6		93		0		14		7				96.61						NA
-80.42	43.65	ELORA RC	6142286	43657.2	2019	7	11	0.20833	19.7		18.4		92		0		13		4				96.6						NA
-80.42	43.65	ELORA RC	6142286	43657.3	2019	7	11	0.25	19.9		18.9		94		0		12		3				96.59						NA
-80.42	43.65	ELORA RC	6142286	43657.3	2019	7	11	0.29167	20.7		19.4		92		0		17		6				96.59		28				NA
-80.42	43.65	ELORA RC	6142286	43657.3	2019	7	11	0.33333	21.9		20.2		91		0		20		6				96.58		30				NA
-80.42	43.65	ELORA RC	6142286	43657.4	2019	7	11	0.375	23.7		21.2		86		0		23		9				96.56		32				NA
-80.42	43.65	ELORA RC	6142286	43657.4	2019	7	11	0.41667	25.8		21.9		79		0		20		18				96.49		35				NA
-80.42	43.65	ELORA RC	6142286	43657.5	2019	7	11	0.45833	26.4		20.8		72		0		24		15				96.47		35				NA
-80.42	43.65	ELORA RC	6142286	43657.5	2019	7	11	0.5	24.8		20.2		75		0		32		28				96.5		33				NA
-80.42	43.65	ELORA RC	6142286	43657.5	2019	7	11	0.54167	26		19.3		67		0.8		31		22				96.51		33				NA
-80.42	43.65	ELORA RC	6142286	43657.6	2019	7	11	0.58333	24.4		18.2		68		0		31		25				96.48		31				NA
-80.42	43.65	ELORA RC	6142286	43657.6	2019	7	11	0.625	24.3		17		64		0		31		21				96.5		30				NA
-80.42	43.65	ELORA RC	6142286	43657.7	2019	7	11	0.66667	22.6		16.7		70		0		31		22				96.52		28				NA
-80.42	43.65	ELORA RC	6142286	43657.7	2019	7	11	0.70833	21		15.2		70		0		31		30				96.54		25				NA
-80.42	43.65	ELORA RC	6142286	43657.8	2019	7	11	0.75	20.7		14.7		68		0		31		23				96.56						NA
-80.42	43.65	ELORA RC	6142286	43657.8	2019	7	11	0.79167	18.8		13.8		73		0		31		16				96.59						NA
-80.42	43.65	ELORA RC	6142286	43657.8	2019	7	11	0.83333	17.5		12.6		73		0		30		20				96.64						NA
-80.42	43.65	ELORA RC	6142286	43657.9	2019	7	11	0.875	16.7		12.6		77		0		31		21				96.65						NA
-80.42	43.65	ELORA RC	6142286	43657.9	2019	7	11	0.91667	16.2		13.2		82		0		31		24				96.67						NA
-80.42	43.65	ELORA RC	6142286	43658	2019	7	11	0.95833	15.5		13.4		87		0.9		32		20				96.61						NA
-80.42	43.65	ELORA RC	6142286	43658	2019	7	12	0	15.5		13.4		87		0		31		22				96.61						NA
-80.42	43.65	ELORA RC	6142286	43658	2019	7	12	0.04167	15.6		13.5		87		0		31		17				96.63						NA
-80.42	43.65	ELORA RC	6142286	43658.1	2019	7	12	0.08333	15.3		13.5		89		0		31		14				96.63						NA
-80.42	43.65	ELORA RC	6142286	43658.1	2019	7	12	0.125	15.4		13.8		90		0		31		14				96.65						NA
-80.42	43.65	ELORA RC	6142286	43658.2	2019	7	12	0.16667	15.2		13.9		92		0		32		9				96.67						NA
-80.42	43.65	ELORA RC	6142286	43658.2	2019	7	12	0.20833	15		13.7		92		0		32		8				96.7						NA
-80.42	43.65	ELORA RC	6142286	43658.3	2019	7	12	0.25	16.3		14.4		88		0		31		14				96.75						NA

-80.42	43.65	ELORA RC	6142286	43658.3	2019	7	12	0.29167	16.5		14.5		88		0		30		15				96.8						NA
-80.42	43.65	ELORA RC	6142286	43658.3	2019	7	12	0.33333	16.8		13.6		82		0		30		20				96.83						NA
-80.42	43.65	ELORA RC	6142286	43658.4	2019	7	12	0.375	16.8		14		84		0		30		19				96.84						NA
-80.42	43.65	ELORA RC	6142286	43658.4	2019	7	12	0.41667	16.5		13.5		83		0		29		17				96.87						NA
-80.42	43.65	ELORA RC	6142286	43658.5	2019	7	12	0.45833	17		13.9		82		0		32		16				96.92						NA
-80.42	43.65	ELORA RC	6142286	43658.5	2019	7	12	0.5	16.5		13.7		84		0		33		17				96.94						NA
-80.42	43.65	ELORA RC	6142286	43658.5	2019	7	12	0.54167	17.6		13.6		78		0		32		15				96.94						NA
-80.42	43.65	ELORA RC	6142286	43658.6	2019	7	12	0.58333	18.3		13.5		74		0		31		13				96.93						NA
-80.42	43.65	ELORA RC	6142286	43658.6	2019	7	12	0.625	19.2		14		72		0		32		10				96.91						NA
-80.42	43.65	ELORA RC	6142286	43658.7	2019	7	12	0.66667	20.2		14.1		68		0		32		13				96.9						NA
-80.42	43.65	ELORA RC	6142286	43658.7	2019	7	12	0.70833	20.4		14.8		70		0		29		10				96.88						NA
-80.42	43.65	ELORA RC	6142286	43658.8	2019	7	12	0.75	21.4		14.7		66		0		28		12				96.86		25				NA
-80.42	43.65	ELORA RC	6142286	43658.8	2019	7	12	0.79167	21		14.9		68		0		27		13				96.91		25				NA
-80.42	43.65	ELORA RC	6142286	43658.8	2019	7	12	0.83333	18.7		14.8		78		0		29		10				96.93						NA
-80.42	43.65	ELORA RC	6142286	43658.9	2019	7	12	0.875	16.8		14.2		85		0		29		8				96.95						NA
-80.42	43.65	ELORA RC	6142286	43658.9	2019	7	12	0.91667	15.1		13.5		90		0		24		6				96.95						NA
-80.42	43.65	ELORA RC	6142286	43659	2019	7	12	0.95833	14.7		13.7		94		0				0				96.95						NA
-80.42	43.65	ELORA RC	6142286	43659	2019	7	13	0	12.8		12.1		96		0		26		5				96.96						NA
-80.42	43.65	ELORA RC	6142286	43659	2019	7	13	0.04167	12.4		11.7		96		0				0				96.99						NA
-80.42	43.65	ELORA RC	6142286	43659.1	2019	7	13	0.08333	12.1		11.3		95		1.2				0				96.98						NA
-80.42	43.65	ELORA RC	6142286	43659.1	2019	7	13	0.125	12		11.4		96		0				0				96.96						NA
-80.42	43.65	ELORA RC	6142286	43659.2	2019	7	13	0.16667	12.1		11.5		96		0				0				96.96						NA
-80.42	43.65	ELORA RC	6142286	43659.2	2019	7	13	0.20833	12.7		11.9		95		0				0				96.97						NA
-80.42	43.65	ELORA RC	6142286	43659.3	2019	7	13	0.25	14.1		13.2		94		0.8				0				96.97						NA
-80.42	43.65	ELORA RC	6142286	43659.3	2019	7	13	0.29167	17.1		15.4		90		0		21		5				96.97						NA
-80.42	43.65	ELORA RC	6142286	43659.3	2019	7	13	0.33333	20.3		17.2		82		0		23		12				96.97		26				NA
-80.42	43.65	ELORA RC	6142286	43659.4	2019	7	13	0.375	23.4		17.3		69		0		23		18				96.94		29				NA
-80.42	43.65	ELORA RC	6142286	43659.4	2019	7	13	0.41667	24.8		15.4		56		0		23		24				96.9		29				NA
-80.42	43.65	ELORA RC	6142286	43659.5	2019	7	13	0.45833	25.9		15.9		54		0		24		27				96.89		30				NA
-80.42	43.65	ELORA RC	6142286	43659.5	2019	7	13	0.5	27.1		16.8		53		0		24		27				96.86		32				NA
-80.42	43.65	ELORA RC	6142286	43659.5	2019	7	13	0.54167	27.7		17.8		55		0		24		23				96.81		34				NA
-80.42	43.65	ELORA RC	6142286	43659.6	2019	7	13	0.58333	27.4		18.8		59		0		25		33				96.79		34				NA
-80.42	43.65	ELORA RC	6142286	43659.6	2019	7	13	0.625	24.6		21.6		84		0		28		18				96.84		34				NA
-80.42	43.65	ELORA RC	6142286	43659.7	2019	7	13	0.66667	26.6		20		67		0		30		22				96.82		34				NA
-80.42	43.65	ELORA RC	6142286	43659.7	2019	7	13	0.70833	26.4		20.4		70		0		32		19				96.84		34				NA
-80.42	43.65	ELORA RC	6142286	43659.8	2019	7	13	0.75	25.9		19.7		69		0		30		16				96.84		33				NA
-80.42	43.65	ELORA RC	6142286	43659.8	2019	7	13	0.79167	24.9		19.6		72		0		30		14				96.88		32				NA
-80.42	43.65	ELORA RC	6142286	43659.8	2019	7	13	0.83333	22.6		17.3		72		0		33		19				96.96		28				NA
-80.42	43.65	ELORA RC	6142286	43659.9	2019	7	13	0.875	20.1		14.1		68		0		33		13				97.05						NA
-80.42	43.65	ELORA RC	6142286	43659.9	2019	7	13	0.91667	18.4		14.4		77		0		31		14				97.11						NA
-80.42	43.65	ELORA RC	6142286	43660	2019	7	13	0.95833	16.5		12		75		0		31		12				97.16						NA
-80.42	43.65	ELORA RC	6142286	43660	2019	7	14	0	16.2		11.2		72		0		32		17				97.17						NA
-80.42	43.65	ELORA RC	6142286	43660	2019	7	14	0.04167	15.1		9.6		70		0		30		16				97.21						NA
-80.42	43.65	ELORA RC	6142286	43660.1	2019	7	14	0.08333	14.3		9.4		72		0		31		16				97.23						NA
-80.42	43.65	ELORA RC	6142286	43660.1	2019	7	14	0.125	13.5		10		80		0		32		11				97.26						NA
-80.42	43.65	ELORA RC	6142286	43660.2	2019	7	14	0.16667	13.3		11		86		0		34		14				97.3						NA
-80.42	43.65	ELORA RC	6142286	43660.2	2019	7	14	0.20833	12.9		11.2		90		0		35		9				97.3						NA
-80.42	43.65	ELORA RC	6142286	43660.3	2019	7	14	0.25	14		11.8		86		0		35		10				97.35						NA
-80.42	43.65	ELORA RC	6142286	43660.3	2019	7	14	0.29167	16.7		12.9		79		0		34		5				97.43						NA
-80.42	43.65	ELORA RC	6142286	43660.3	2019	7	14	0.33333	18.3		12.5		69		0		36		8				97.51						NA
-80.42	43.65	ELORA RC	6142286	43660.4	2019	7	14	0.375	20.1		13.1		64		0		30		9				97.56						NA
-80.42	43.65	ELORA RC	6142286	43660.4	2019	7	14	0.41667	21.5		13.9		62		0		30		16				97.54		25				NA
-80.42	43.65	ELORA RC	6142286	43660.5	2019	7	14	0.45833	22.2		12.8		55		0		32		18				97.56		25				NA
-80.42	43.65	ELORA RC	6142286	43660.5	2019	7	14	0.5	22.4		12.9		55		0		31		18				97.53		25				NA
-80.42	43.65	ELORA RC	6142286	43660.5	2019	7	14	0.54167	23.5		13.1		52		0		30		19				97.53		26				NA
-80.42	43.65	ELORA RC	6142286	43660.6	2019	7	14	0.58333	24.2		13.4		51		0		32		17				97.5		27				NA
-80.42	43.65	ELORA RC	6142286	43660.6	2019	7	14	0.625	24.5		13.6		50		0		30		14				97.51		28				NA
-80.42	43.65	ELORA RC	6142286	43660.7	2019	7	14	0.66667	24.5		12.5		47		0		32		13				97.51		27				NA
-80.42	43.65	ELORA RC	6142286	43660.7	2019	7	14	0.70833	24.4		12.5		48		0		32		11				97.47		27				NA
-80.42	43.65	ELORA RC	6142286	43660.8	2019	7	14	0.75	23.7		11.7		47		0		30		14				97.47		26				NA
-80.42	43.65	ELORA RC	6142286	43660.8	2019	7	14	0.79167	22		12.4		54		0		32		10				97.48						NA
-80.42	43.65	ELORA RC	6142286	43660.8	2019	7	14	0.83333	18.9		12.2		65		0		31		11				97.53						NA
-80.42	43.65	ELORA RC	6142286	43660.9	2019	7	14	0.875	17.6		11.7		68		0		33		5				97.54						NA
-80.42	43.65	ELORA RC	6142286	43660.9	2019	7	14	0.91667	16.4		11.5		73		2.9		31		6				97.56						NA
-80.42	43.65	ELORA RC	6142286	43661	2019	7	14	0.95833	15		10.7		76		0		34		4				97.57						NA
-80.42	43.65	ELORA RC	6142286	43661	2019	7	15	0	12.4		9.4		82		0		36		4				97.55						NA
-80.42	43.65	ELORA RC	6142286	43661	2019	7	15	0.04167	11.8		9.8		88		0		34		3				97.58						NA
-80.42	43.65	ELORA RC	6142286	43661.1	2019	7	15	0.08333	10.8		9.1		89		0				0				97.57						NA
-80.42	43.65	ELORA RC	6142286	43661.1	2019	7	15	0.125	8.9		7.8		93		0		33		4				97.52						NA
-80.42	43.65	ELORA RC	6142286	43661.2	2019	7	15	0.16667	11.1		10.3		95		0				0				97.55						NA
-80.42	43.65	ELORA RC	6142286	43661.2	2019	7	15	0.20833	9.6		9		96		0				0				97.58						NA
-80.42	43.65	ELORA RC	6142286	43661.3	2019	7	15	0.25	10.9		10.2		95		0				0				97.6						NA
-80.42	43.65	ELORA RC	6142286	43661.3	2019	7	15	0.29167	14.9		12.6		86		0				0				97.63						NA
-80.42	43.65	ELORA RC	6142286	43661.3	2019	7	15	0.33333	18.3		14.2		77		0		15		5				97.63						NA
-80.42	43.65	ELORA RC	6142286	43661.4	2019	7	15	0.375	19.9		13.1		65		0		18		7				97.64						NA
-80.42	43.65	ELORA RC	6142286	43661.4	2019	7	15	0.41667	21.9		11.4		51		0		20		8				97.64						NA
-80.42	43.65	ELORA RC	6142286	43661.5	2019	7	15	0.45833	24.3		11.6		45		0		23		5				97.61		26				NA
-80.42	43.65	ELORA RC	6142286	43661.5	2019	7	15	0.5	24.7		12.2		46		0					M			97.57		27				NA
-80.42	43.65	ELORA RC	6142286	43661.5	2019	7	15	0.54167	26.5			M		M	0					M			97.52						NA
-80.42	43.65	ELORA RC	6142286	43661.6	2019	7	15	0.58333		M		M		M	0					M			97.45						NA
-80.42	43.65	ELORA RC	6142286	43661.6	2019	7	15	0.625		M		M		M	0					M			97.42						NA
-80.42	43.65	ELORA RC	6142286	43661.7	2019	7	15	0.66667		M		M		M	0			M		M				M					NA
-80.42	43.65	ELORA RC	6142286	43661.7	2019	7	15	0.70833		M		M		M	0					M			97.33						NA
-80.42	43.65	ELORA RC	6142286	43661.8	2019	7	15	0.75		M		M		M	0					M			97.28						NA
-80.42	43.65	ELORA RC	6142286	43661.8	2019	7	15	0.79167		M		M		M	0					M			97.25						NA
-80.42	43.65	ELORA RC	6142286	43661.8	2019	7	15	0.83333																					
-80.42	43.65	ELORA RC	6142286	43661.9	2019	7	15	0.875																					
-80.42	43.65	ELORA RC	6142286	43661.9	2019	7	15	0.91667																					
-80.42	43.65	ELORA RC	6142286	43662	2019	7	15	0.95833																					
-80.42	43.65	ELORA RC	6142286	43662	2019	7	16	0																					
-80.42	43.65	ELORA RC	6142286	43662	2019	7	16	0.04167																					
-80.42	43.65	ELORA RC	6142286	43662.1	2019	7	16	0.08333																					
-80.42	43.65	ELORA RC	6142286	43662.1	2019	7	16	0.125																					
-80.42	43.65	ELORA RC	6142286	43662.2	2019	7	16	0.16667																					
-80.42	43.65	ELORA RC	6142286	43662.2	2019	7	16	0.20833																					
-80.42	43.65	ELORA RC	6142286	43662.3	2019	7	16	0.25																					
-80.42	43.65	ELORA RC	6142286	43662.3	2019	7	16	0.29167																					
-80.42	43.65	ELORA RC	6142286	43662.3	2019	7	16	0.33333																					
-80.42	43.65	ELORA RC	6142286	43662.4	2019	7	16	0.375																					
-80.42	43.65	ELORA RC	6142286	43662.4	2019	7	16	0.41667																					
-80.42	43.65	ELORA RC	6142286	43662.5	2019	7	16	0.45833																					
-80.42	43.65	ELORA RC	6142286	43662.5	2019	7	16	0.5																					
-80.42	43.65	ELORA RC	6142286	43662.5	2019	7	16	0.54167																					
-80.42	43.65	ELORA RC	6142286	43662.6	2019	7	16	0.58333																					
-80.42	43.65	ELORA RC	6142286	43662.6	2019	7	16	0.625																					
-80.42	43.65	ELORA RC	6142286	43662.7	2019	7	16	0.66667																					
-80.42	43.65	ELORA RC	6142286	43662.7	2019	7	16	0.70833																					
-80.42	43.65	ELORA RC	6142286	43662.8	2019	7	16	0.75																					
-80.42	43.65	ELORA RC	6142286	43662.8	2019	7	16	0.79167																					
-80.42	43.65	ELORA RC	6142286	43662.8	2019	7	16	0.83333																					
-80.42	43.65	ELORA RC	6142286	43662.9	2019	7	16	0.875																					
-80.42	43.65	ELORA RC	6142286	43662.9	2019	7	16	0.91667																					
-80.42	43.65	ELORA RC	6142286	43663	2019	7	16	0.95833																					
-80.42	43.65	ELORA RC	6142286	43663	2019	7	17	0																					
-80.42	43.65	ELORA RC	6142286	43663	2019	7	17	0.04167																					
-80.42	43.65	ELORA RC	6142286	43663.1	2019	7	17	0.08333																					
-80.42	43.65	ELORA RC	6142286	43663.1	2019	7	17	0.125																					
-80.42	43.65	ELORA RC	6142286	43663.2	2019	7	17	0.16667																					
-80.42	43.65	ELORA RC	6142286	43663.2	2019	7	17	0.20833																					
-80.42	43.65	ELORA RC	6142286	43663.3	2019	7	17	0.25																					
-80.42	43.65	ELORA RC	6142286	43663.3	2019	7	17	0.29167																					
-80.42	43.65	ELORA RC	6142286	43663.3	2019	7	17	0.33333																					
-80.42	43.65	ELORA RC	6142286	43663.4	2019	7	17	0.375																					
-80.42	43.65	ELORA RC	6142286	43663.4	2019	7	17	0.41667																					
-80.42	43.65	ELORA RC	6142286	43663.5	2019	7	17	0.45833																					
-80.42	43.65	ELORA RC	6142286	43663.5	2019	7	17	0.5																					
-80.42	43.65	ELORA RC	6142286	43663.5	2019	7	17	0.54167																					
-80.42	43.65	ELORA RC	6142286	43663.6	2019	7	17	0.58333																					
-80.42	43.65	ELORA RC	6142286	43663.6	2019	7	17	0.625																					
-80.42	43.65	ELORA RC	6142286	43663.7	2019	7	17	0.66667																					
-80.42	43.65	ELORA RC	6142286	43663.7	2019	7	17	0.70833																					
-80.42	43.65	ELORA RC	6142286	43663.8	2019	7	17	0.75																					
-80.42	43.65	ELORA RC	6142286	43663.8	2019	7	17	0.79167																					
-80.42	43.65	ELORA RC	6142286	43663.8	2019	7	17	0.83333																					
-80.42	43.65	ELORA RC	6142286	43663.9	2019	7	17	0.875																					
-80.42	43.65	ELORA RC	6142286	43663.9	2019	7	17	0.91667																					
-80.42	43.65	ELORA RC	6142286	43664	2019	7	17	0.95833																					
-80.42	43.65	ELORA RC	6142286	43664	2019	7	18	0																					

-80.42	43.65	ELORA RC	6142286	43664	2019	7	18	0.04167
-80.42	43.65	ELORA RC	6142286	43664.1	2019	7	18	0.08333
-80.42	43.65	ELORA RC	6142286	43664.1	2019	7	18	0.125
-80.42	43.65	ELORA RC	6142286	43664.2	2019	7	18	0.16667
-80.42	43.65	ELORA RC	6142286	43664.2	2019	7	18	0.20833
-80.42	43.65	ELORA RC	6142286	43664.3	2019	7	18	0.25
-80.42	43.65	ELORA RC	6142286	43664.3	2019	7	18	0.29167
-80.42	43.65	ELORA RC	6142286	43664.3	2019	7	18	0.33333
-80.42	43.65	ELORA RC	6142286	43664.4	2019	7	18	0.375
-80.42	43.65	ELORA RC	6142286	43664.4	2019	7	18	0.41667
-80.42	43.65	ELORA RC	6142286	43664.5	2019	7	18	0.45833
-80.42	43.65	ELORA RC	6142286	43664.5	2019	7	18	0.5
-80.42	43.65	ELORA RC	6142286	43664.5	2019	7	18	0.54167
-80.42	43.65	ELORA RC	6142286	43664.6	2019	7	18	0.58333
-80.42	43.65	ELORA RC	6142286	43664.6	2019	7	18	0.625
-80.42	43.65	ELORA RC	6142286	43664.7	2019	7	18	0.66667
-80.42	43.65	ELORA RC	6142286	43664.7	2019	7	18	0.70833
-80.42	43.65	ELORA RC	6142286	43664.8	2019	7	18	0.75
-80.42	43.65	ELORA RC	6142286	43664.8	2019	7	18	0.79167
-80.42	43.65	ELORA RC	6142286	43664.8	2019	7	18	0.83333
-80.42	43.65	ELORA RC	6142286	43664.9	2019	7	18	0.875
-80.42	43.65	ELORA RC	6142286	43664.9	2019	7	18	0.91667
-80.42	43.65	ELORA RC	6142286	43665	2019	7	18	0.95833
-80.42	43.65	ELORA RC	6142286	43665	2019	7	19	0
-80.42	43.65	ELORA RC	6142286	43665	2019	7	19	0.04167
-80.42	43.65	ELORA RC	6142286	43665.1	2019	7	19	0.08333
-80.42	43.65	ELORA RC	6142286	43665.1	2019	7	19	0.125
-80.42	43.65	ELORA RC	6142286	43665.2	2019	7	19	0.16667
-80.42	43.65	ELORA RC	6142286	43665.2	2019	7	19	0.20833
-80.42	43.65	ELORA RC	6142286	43665.3	2019	7	19	0.25
-80.42	43.65	ELORA RC	6142286	43665.3	2019	7	19	0.29167
-80.42	43.65	ELORA RC	6142286	43665.3	2019	7	19	0.33333
-80.42	43.65	ELORA RC	6142286	43665.4	2019	7	19	0.375
-80.42	43.65	ELORA RC	6142286	43665.4	2019	7	19	0.41667
-80.42	43.65	ELORA RC	6142286	43665.5	2019	7	19	0.45833
-80.42	43.65	ELORA RC	6142286	43665.5	2019	7	19	0.5
-80.42	43.65	ELORA RC	6142286	43665.5	2019	7	19	0.54167
-80.42	43.65	ELORA RC	6142286	43665.6	2019	7	19	0.58333
-80.42	43.65	ELORA RC	6142286	43665.6	2019	7	19	0.625
-80.42	43.65	ELORA RC	6142286	43665.7	2019	7	19	0.66667
-80.42	43.65	ELORA RC	6142286	43665.7	2019	7	19	0.70833
-80.42	43.65	ELORA RC	6142286	43665.8	2019	7	19	0.75
-80.42	43.65	ELORA RC	6142286	43665.8	2019	7	19	0.79167
-80.42	43.65	ELORA RC	6142286	43665.8	2019	7	19	0.83333
-80.42	43.65	ELORA RC	6142286	43665.9	2019	7	19	0.875
-80.42	43.65	ELORA RC	6142286	43665.9	2019	7	19	0.91667
-80.42	43.65	ELORA RC	6142286	43666	2019	7	19	0.95833
-80.42	43.65	ELORA RC	6142286	43666	2019	7	20	0
-80.42	43.65	ELORA RC	6142286	43666	2019	7	20	0.04167
-80.42	43.65	ELORA RC	6142286	43666.1	2019	7	20	0.08333
-80.42	43.65	ELORA RC	6142286	43666.1	2019	7	20	0.125
-80.42	43.65	ELORA RC	6142286	43666.2	2019	7	20	0.16667
-80.42	43.65	ELORA RC	6142286	43666.2	2019	7	20	0.20833
-80.42	43.65	ELORA RC	6142286	43666.3	2019	7	20	0.25
-80.42	43.65	ELORA RC	6142286	43666.3	2019	7	20	0.29167
-80.42	43.65	ELORA RC	6142286	43666.3	2019	7	20	0.33333
-80.42	43.65	ELORA RC	6142286	43666.4	2019	7	20	0.375
-80.42	43.65	ELORA RC	6142286	43666.4	2019	7	20	0.41667
-80.42	43.65	ELORA RC	6142286	43666.5	2019	7	20	0.45833
-80.42	43.65	ELORA RC	6142286	43666.5	2019	7	20	0.5
-80.42	43.65	ELORA RC	6142286	43666.5	2019	7	20	0.54167
-80.42	43.65	ELORA RC	6142286	43666.6	2019	7	20	0.58333
-80.42	43.65	ELORA RC	6142286	43666.6	2019	7	20	0.625
-80.42	43.65	ELORA RC	6142286	43666.7	2019	7	20	0.66667
-80.42	43.65	ELORA RC	6142286	43666.7	2019	7	20	0.70833
-80.42	43.65	ELORA RC	6142286	43666.8	2019	7	20	0.75
-80.42	43.65	ELORA RC	6142286	43666.8	2019	7	20	0.79167
-80.42	43.65	ELORA RC	6142286	43666.8	2019	7	20	0.83333
-80.42	43.65	ELORA RC	6142286	43666.9	2019	7	20	0.875
-80.42	43.65	ELORA RC	6142286	43666.9	2019	7	20	0.91667
-80.42	43.65	ELORA RC	6142286	43667	2019	7	20	0.95833
-80.42	43.65	ELORA RC	6142286	43667	2019	7	21	0
-80.42	43.65	ELORA RC	6142286	43667	2019	7	21	0.04167
-80.42	43.65	ELORA RC	6142286	43667.1	2019	7	21	0.08333
-80.42	43.65	ELORA RC	6142286	43667.1	2019	7	21	0.125
-80.42	43.65	ELORA RC	6142286	43667.2	2019	7	21	0.16667
-80.42	43.65	ELORA RC	6142286	43667.2	2019	7	21	0.20833
-80.42	43.65	ELORA RC	6142286	43667.3	2019	7	21	0.25
-80.42	43.65	ELORA RC	6142286	43667.3	2019	7	21	0.29167
-80.42	43.65	ELORA RC	6142286	43667.3	2019	7	21	0.33333
-80.42	43.65	ELORA RC	6142286	43667.4	2019	7	21	0.375
-80.42	43.65	ELORA RC	6142286	43667.4	2019	7	21	0.41667
-80.42	43.65	ELORA RC	6142286	43667.5	2019	7	21	0.45833
-80.42	43.65	ELORA RC	6142286	43667.5	2019	7	21	0.5
-80.42	43.65	ELORA RC	6142286	43667.5	2019	7	21	0.54167
-80.42	43.65	ELORA RC	6142286	43667.6	2019	7	21	0.58333
-80.42	43.65	ELORA RC	6142286	43667.6	2019	7	21	0.625
-80.42	43.65	ELORA RC	6142286	43667.7	2019	7	21	0.66667
-80.42	43.65	ELORA RC	6142286	43667.7	2019	7	21	0.70833
-80.42	43.65	ELORA RC	6142286	43667.8	2019	7	21	0.75
-80.42	43.65	ELORA RC	6142286	43667.8	2019	7	21	0.79167
-80.42	43.65	ELORA RC	6142286	43667.8	2019	7	21	0.83333
-80.42	43.65	ELORA RC	6142286	43667.9	2019	7	21	0.875
-80.42	43.65	ELORA RC	6142286	43667.9	2019	7	21	0.91667
-80.42	43.65	ELORA RC	6142286	43668	2019	7	21	0.95833
-80.42	43.65	ELORA RC	6142286	43668	2019	7	22	0
-80.42	43.65	ELORA RC	6142286	43668	2019	7	22	0.04167
-80.42	43.65	ELORA RC	6142286	43668.1	2019	7	22	0.08333
-80.42	43.65	ELORA RC	6142286	43668.1	2019	7	22	0.125
-80.42	43.65	ELORA RC	6142286	43668.2	2019	7	22	0.16667
-80.42	43.65	ELORA RC	6142286	43668.2	2019	7	22	0.20833
-80.42	43.65	ELORA RC	6142286	43668.3	2019	7	22	0.25
-80.42	43.65	ELORA RC	6142286	43668.3	2019	7	22	0.29167
-80.42	43.65	ELORA RC	6142286	43668.3	2019	7	22	0.33333
-80.42	43.65	ELORA RC	6142286	43668.4	2019	7	22	0.375
-80.42	43.65	ELORA RC	6142286	43668.4	2019	7	22	0.41667
-80.42	43.65	ELORA RC	6142286	43668.5	2019	7	22	0.45833
-80.42	43.65	ELORA RC	6142286	43668.5	2019	7	22	0.5
-80.42	43.65	ELORA RC	6142286	43668.5	2019	7	22	0.54167
-80.42	43.65	ELORA RC	6142286	43668.6	2019	7	22	0.58333
-80.42	43.65	ELORA RC	6142286	43668.6	2019	7	22	0.625
-80.42	43.65	ELORA RC	6142286	43668.7	2019	7	22	0.66667
-80.42	43.65	ELORA RC	6142286	43668.7	2019	7	22	0.70833
-80.42	43.65	ELORA RC	6142286	43668.8	2019	7	22	0.75
-80.42	43.65	ELORA RC	6142286	43668.8	2019	7	22	0.79167
-80.42	43.65	ELORA RC	6142286	43668.8	2019	7	22	0.83333
-80.42	43.65	ELORA RC	6142286	43668.9	2019	7	22	0.875
-80.42	43.65	ELORA RC	6142286	43668.9	2019	7	22	0.91667
-80.42	43.65	ELORA RC	6142286	43669	2019	7	22	0.95833
-80.42	43.65	ELORA RC	6142286	43669	2019	7	23	0
-80.42	43.65	ELORA RC	6142286	43669	2019	7	23	0.04167
-80.42	43.65	ELORA RC	6142286	43669.1	2019	7	23	0.08333
-80.42	43.65	ELORA RC	6142286	43669.1	2019	7	23	0.125
-80.42	43.65	ELORA RC	6142286	43669.2	2019	7	23	0.16667
-80.42	43.65	ELORA RC	6142286	43669.2	2019	7	23	0.20833
-80.42	43.65	ELORA RC	6142286	43669.3	2019	7	23	0.25
-80.42	43.65	ELORA RC	6142286	43669.3	2019	7	23	0.29167
-80.42	43.65	ELORA RC	6142286	43669.3	2019	7	23	0.33333
-80.42	43.65	ELORA RC	6142286	43669.4	2019	7	23	0.375
-80.42	43.65	ELORA RC	6142286	43669.4	2019	7	23	0.41667
-80.42	43.65	ELORA RC	6142286	43669.5	2019	7	23	0.45833
-80.42	43.65	ELORA RC	6142286	43669.5	2019	7	23	0.5
-80.42	43.65	ELORA RC	6142286	43669.5	2019	7	23	0.54167
-80.42	43.65	ELORA RC	6142286	43669.6	2019	7	23	0.58333
-80.42	43.65	ELORA RC	6142286	43669.6	2019	7	23	0.625
-80.42	43.65	ELORA RC	6142286	43669.7	2019	7	23	0.66667
-80.42	43.65	ELORA RC	6142286	43669.7	2019	7	23	0.70833
-80.42	43.65	ELORA RC	6142286	43669.8	2019	7	23	0.75

-80.42	43.65	ELORA RC	6142286	43669.8	2019	7	23	0.79167																					
-80.42	43.65	ELORA RC	6142286	43669.8	2019	7	23	0.83333																					
-80.42	43.65	ELORA RC	6142286	43669.9	2019	7	23	0.875																					
-80.42	43.65	ELORA RC	6142286	43669.9	2019	7	23	0.91667																					
-80.42	43.65	ELORA RC	6142286	43670	2019	7	23	0.95833																					
-80.42	43.65	ELORA RC	6142286	43670	2019	7	24	0																					
-80.42	43.65	ELORA RC	6142286	43670	2019	7	24	0.04167																					
-80.42	43.65	ELORA RC	6142286	43670.1	2019	7	24	0.08333																					
-80.42	43.65	ELORA RC	6142286	43670.1	2019	7	24	0.125																					
-80.42	43.65	ELORA RC	6142286	43670.2	2019	7	24	0.16667																					
-80.42	43.65	ELORA RC	6142286	43670.2	2019	7	24	0.20833																					
-80.42	43.65	ELORA RC	6142286	43670.3	2019	7	24	0.25																					
-80.42	43.65	ELORA RC	6142286	43670.3	2019	7	24	0.29167																					
-80.42	43.65	ELORA RC	6142286	43670.3	2019	7	24	0.33333																					
-80.42	43.65	ELORA RC	6142286	43670.4	2019	7	24	0.375																					
-80.42	43.65	ELORA RC	6142286	43670.4	2019	7	24	0.41667																					
-80.42	43.65	ELORA RC	6142286	43670.5	2019	7	24	0.45833																					
-80.42	43.65	ELORA RC	6142286	43670.5	2019	7	24	0.5																					
-80.42	43.65	ELORA RC	6142286	43670.5	2019	7	24	0.54167																					
-80.42	43.65	ELORA RC	6142286	43670.6	2019	7	24	0.58333																					
-80.42	43.65	ELORA RC	6142286	43670.6	2019	7	24	0.625																					
-80.42	43.65	ELORA RC	6142286	43670.7	2019	7	24	0.66667																					
-80.42	43.65	ELORA RC	6142286	43670.7	2019	7	24	0.70833																					
-80.42	43.65	ELORA RC	6142286	43670.8	2019	7	24	0.75																					
-80.42	43.65	ELORA RC	6142286	43670.8	2019	7	24	0.79167																					
-80.42	43.65	ELORA RC	6142286	43670.8	2019	7	24	0.83333																					
-80.42	43.65	ELORA RC	6142286	43670.9	2019	7	24	0.875																					
-80.42	43.65	ELORA RC	6142286	43670.9	2019	7	24	0.91667																					
-80.42	43.65	ELORA RC	6142286	43671	2019	7	24	0.95833																					
-80.42	43.65	ELORA RC	6142286	43671	2019	7	25	0																					
-80.42	43.65	ELORA RC	6142286	43671	2019	7	25	0.04167																					
-80.42	43.65	ELORA RC	6142286	43671.1	2019	7	25	0.08333																					
-80.42	43.65	ELORA RC	6142286	43671.1	2019	7	25	0.125																					
-80.42	43.65	ELORA RC	6142286	43671.2	2019	7	25	0.16667																					
-80.42	43.65	ELORA RC	6142286	43671.2	2019	7	25	0.20833																					
-80.42	43.65	ELORA RC	6142286	43671.3	2019	7	25	0.25																					
-80.42	43.65	ELORA RC	6142286	43671.3	2019	7	25	0.29167																					
-80.42	43.65	ELORA RC	6142286	43671.3	2019	7	25	0.33333																					
-80.42	43.65	ELORA RC	6142286	43671.4	2019	7	25	0.375																					
-80.42	43.65	ELORA RC	6142286	43671.4	2019	7	25	0.41667																					
-80.42	43.65	ELORA RC	6142286	43671.5	2019	7	25	0.45833																					
-80.42	43.65	ELORA RC	6142286	43671.5	2019	7	25	0.5																					
-80.42	43.65	ELORA RC	6142286	43671.5	2019	7	25	0.54167																					
-80.42	43.65	ELORA RC	6142286	43671.6	2019	7	25	0.58333																					
-80.42	43.65	ELORA RC	6142286	43671.6	2019	7	25	0.625																					
-80.42	43.65	ELORA RC	6142286	43671.7	2019	7	25	0.66667																					
-80.42	43.65	ELORA RC	6142286	43671.7	2019	7	25	0.70833																					
-80.42	43.65	ELORA RC	6142286	43671.8	2019	7	25	0.75																					
-80.42	43.65	ELORA RC	6142286	43671.8	2019	7	25	0.79167																					
-80.42	43.65	ELORA RC	6142286	43671.8	2019	7	25	0.83333																					
-80.42	43.65	ELORA RC	6142286	43671.9	2019	7	25	0.875																					
-80.42	43.65	ELORA RC	6142286	43671.9	2019	7	25	0.91667																					
-80.42	43.65	ELORA RC	6142286	43672	2019	7	25	0.95833																					
-80.42	43.65	ELORA RC	6142286	43672	2019	7	26	0																					
-80.42	43.65	ELORA RC	6142286	43672	2019	7	26	0.04167																					
-80.42	43.65	ELORA RC	6142286	43672.1	2019	7	26	0.08333																					
-80.42	43.65	ELORA RC	6142286	43672.1	2019	7	26	0.125																					
-80.42	43.65	ELORA RC	6142286	43672.2	2019	7	26	0.16667																					
-80.42	43.65	ELORA RC	6142286	43672.2	2019	7	26	0.20833																					
-80.42	43.65	ELORA RC	6142286	43672.3	2019	7	26	0.25																					
-80.42	43.65	ELORA RC	6142286	43672.3	2019	7	26	0.29167																					
-80.42	43.65	ELORA RC	6142286	43672.3	2019	7	26	0.33333																					
-80.42	43.65	ELORA RC	6142286	43672.4	2019	7	26	0.375																					
-80.42	43.65	ELORA RC	6142286	43672.4	2019	7	26	0.41667																					
-80.42	43.65	ELORA RC	6142286	43672.5	2019	7	26	0.45833	26.1		16		54		0		22		12				98.08		31				NA
-80.42	43.65	ELORA RC	6142286	43672.5	2019	7	26	0.5																					
-80.42	43.65	ELORA RC	6142286	43672.5	2019	7	26	0.54167	26.6		16.2		53		0		21		15				98.06		31				NA
-80.42	43.65	ELORA RC	6142286	43672.6	2019	7	26	0.58333	27		14.9		48		0		22		10				98.02		31				NA
-80.42	43.65	ELORA RC	6142286	43672.6	2019	7	26	0.625	27.4		15.4		48		0		25		7				97.98		32				NA
-80.42	43.65	ELORA RC	6142286	43672.7	2019	7	26	0.66667	27.3		14.6		46		0		21		6				97.91		31				NA
-80.42	43.65	ELORA RC	6142286	43672.7	2019	7	26	0.70833	24.2		18.1		69		0		14		16				97.93		30				NA
-80.42	43.65	ELORA RC	6142286	43672.8	2019	7	26	0.75	25.7		15.7		54		0		20		11				97.9		30				NA
-80.42	43.65	ELORA RC	6142286	43672.8	2019	7	26	0.79167	24		16.3		62		0		21		11				97.92		29				NA
-80.42	43.65	ELORA RC	6142286	43672.8	2019	7	26	0.83333	20.9		16		74		0		22		8				97.95		26				NA
-80.42	43.65	ELORA RC	6142286	43672.9	2019	7	26	0.875	19.1		15.8		81		0		19		9				97.95						NA
-80.42	43.65	ELORA RC	6142286	43672.9	2019	7	26	0.91667	18.1		14.3		79		0		19		4				97.95						NA
-80.42	43.65	ELORA RC	6142286	43673	2019	7	26	0.95833	15.7		13.6		87		0		9		3				97.93						NA
-80.42	43.65	ELORA RC	6142286	43673	2019	7	27	0	16.3		14.6		90		0				0				97.93						NA
-80.42	43.65	ELORA RC	6142286	43673	2019	7	27	0.04167	14.4		12.9		91		0				0				97.9						NA
-80.42	43.65	ELORA RC	6142286	43673.1	2019	7	27	0.08333	14.3		13.8		97		0		11		3				97.89						NA
-80.42	43.65	ELORA RC	6142286	43673.1	2019	7	27	0.125	14.3		13.7		96		0		8		3				97.86						NA
-80.42	43.65	ELORA RC	6142286	43673.2	2019	7	27	0.16667	13.8		13.1		96		0		9		5				97.85						NA
-80.42	43.65	ELORA RC	6142286	43673.2	2019	7	27	0.20833	14		13.6		97		0		10		3				97.86						NA
-80.42	43.65	ELORA RC	6142286	43673.3	2019	7	27	0.25	15.8		15.2		96		0		13		5				97.86						NA
-80.42	43.65	ELORA RC	6142286	43673.3	2019	7	27	0.29167	17.7		15.7		88		0		15		5				97.85						NA
-80.42	43.65	ELORA RC	6142286	43673.3	2019	7	27	0.33333	21.4		18		81		0		19		10				97.84		27				NA
-80.42	43.65	ELORA RC	6142286	43673.4	2019	7	27	0.375	23.3		17.2		69		0		22		17				97.82		29				NA
-80.42	43.65	ELORA RC	6142286	43673.4	2019	7	27	0.41667	23.8		16.7		65		0		22		18				97.81		29				NA
-80.42	43.65	ELORA RC	6142286	43673.5	2019	7	27	0.45833	23.9		17		65		0		22		14				97.8		29				NA
-80.42	43.65	ELORA RC	6142286	43673.5	2019	7	27	0.5	26.5		16		53		0		21		23				97.72		31				NA
-80.42	43.65	ELORA RC	6142286	43673.5	2019	7	27	0.54167	26.6		15.9		52		0		21		25				97.62		31				NA
-80.42	43.65	ELORA RC	6142286	43673.6	2019	7	27	0.58333	25.7		16.6		57		0		20		22				97.58		31				NA
-80.42	43.65	ELORA RC	6142286	43673.6	2019	7	27	0.625	26.9		16.3		52		0		21		28				97.53		32				NA
-80.42	43.65	ELORA RC	6142286	43673.7	2019	7	27	0.66667	26.8		15.7		51		0		20		27				97.47		31				NA
-80.42	43.65	ELORA RC	6142286	43673.7	2019	7	27	0.70833	25.7		15.3		53		0		22		29				97.43		30				NA
-80.42	43.65	ELORA RC	6142286	43673.8	2019	7	27	0.75	25.3		15.9		56		0		21		28				97.42		30				NA
-80.42	43.65	ELORA RC	6142286	43673.8	2019	7	27	0.79167	24.2		16		60		0		20		21				97.4		29				NA
-80.42	43.65	ELORA RC	6142286	43673.8	2019	7	27	0.83333	23.3		16.1		64		0		20		16				97.4		28				NA
-80.42	43.65	ELORA RC	6142286	43673.9	2019	7	27	0.875	22.8		16.2		66		0		21		15				97.43		28				NA
-80.42	43.65	ELORA RC	6142286	43673.9	2019	7	27	0.91667	22.8		15.5		63		0		21		21				97.37		27				NA
-80.42	43.65	ELORA RC	6142286	43674	2019	7	27	0.95833	21.8		15.8		69		0		22		15				97.37		26				NA
-80.42	43.65	ELORA RC	6142286	43674	2019	7	28	0	21.3		16.3		73		0		21		14				97.36		26				NA
-80.42	43.65	ELORA RC	6142286	43674	2019	7	28	0.04167	20.9		16.8		77		0		25		11				97.4		26				NA
-80.42	43.65	ELORA RC	6142286	43674.1	2019	7	28	0.08333	19.7		15.7		78		0		25		4				97.29						NA
-80.42	43.65	ELORA RC	6142286	43674.1	2019	7	28	0.125	19.7		17.2		85		0		20		6				97.27						NA
-80.42	43.65	ELORA RC	6142286	43674.2	2019	7	28	0.16667	19.9		17.3		85		0		25		7				97.23						NA
-80.42	43.65	ELORA RC	6142286	43674.2	2019	7	28	0.20833	20		17.9		88		0		22		13				97.27		26				NA
-80.42	43.65	ELORA RC	6142286	43674.3	2019	7	28	0.25	20.3		17.7		85		0		22		13				97.29		26				NA
-80.42	43.65	ELORA RC	6142286	43674.3	2019	7	28	0.29167	20.8		18.5		87		0		22		17				97.3		27				NA
-80.42	43.65	ELORA RC	6142286	43674.3	2019	7	28	0.33333	21.4		18.9		86		0		23		15				97.34		28				NA
-80.42	43.65	ELORA RC	6142286	43674.4	2019	7	28	0.375	22.5		19.1		82		0		23		16				97.34		29				NA
-80.42	43.65	ELORA RC	6142286	43674.4	2019	7	28	0.41667	24.2		19.5		75		0		24		18				97.3		31				NA
-80.42	43.65	ELORA RC	6142286	43674.5	2019	7	28	0.45833	24.9		18.7		68		0		25		18				97.31		31				NA
-80.42	43.65	ELORA RC	6142286	43674.5	2019	7	28	0.5	26.3		18.8		64		0		25		18				97.28		33				NA
-80.42	43.65	ELORA RC	6142286	43674.5	2019	7	28	0.54167	27.3		18		57		0		26		20				97.25		33				NA
-80.42	43.65	ELORA RC	6142286	43674.6	2019	7	28	0.58333	28.2		16.3		48		0		26		24				97.2		33				NA
-80.42	43.65	ELORA RC	6142286	43674.6	2019	7	28	0.625	29.2		15.6		44		0		26		25				97.17		34				NA
-80.42	43.65	ELORA RC	6142286	43674.7	2019	7	28	0.66667	28.6		14.5		42		0		28		24				97.16		32				NA
-80.42	43.65	ELORA RC	6142286	43674.7	2019	7	28	0.70833	28.4		14.5		43		0		28		17				97.13		32				NA
-80.42	43.65	ELORA RC	6142286	43674.8	2019	7	28	0.75	27.4		15.2		47		0		30		18				97.12		31				NA
-80.42	43.65	ELORA RC	6142286	43674.8	2019	7	28	0.79167	25.6		16.3		56		0		30		7				97.2		30				NA
-80.42	43.65	ELORA RC	6142286	43674.8	2019	7	28	0.83333	23.5		15.9		63		0		28		3				97.18		28				NA
-80.42	43.65	ELORA RC	6142286	43674.9	2019	7	28	0.875	21.3		17.4		78		0		19		8				97.21		27				NA
-80.42	43.65	ELORA RC	6142286	43674.9	2019	7	28	0.91667	20.9		18.4		86		0				0				97.21		27				NA
-80.42	43.65	ELORA RC	6142286	43675	2019	7	28	0.95833	18.8		16.9		89		0				0				97.18						NA
-80.42	43.65	ELORA RC	6142286	43675	2019	7	29	0	18.3		17.2		94		0				0				97.17						NA
-80.42	43.65	ELORA RC	6142286	43675	2019	7	29	0.04167	18.7		17.3		91		0		17		4				97.14						NA
-80.42	43.65	ELORA RC	6142286	43675.1	2019	7	29	0.08333	17.7		16.6		94		0		15		3				97.09						NA
-80.42	43.65	ELORA RC	6142286	43675.1	2019	7	29	0.125	18.7		17.9		95		0		21		4				97.08						NA
-80.42	43.65	ELORA RC	6142286	43675.2	2019	7	29	0.16667	18.4		17.9		97		0		23		4				97.05						NA
-80.42	43.65	ELORA RC	6142286	43675.2	2019	7	29	0.20833	17.6		17.2		98		0				0				97.02						NA
-80.42	43.65	ELORA RC	6142286	43675.3	2019	7	29	0.25	17.8		17.4		98		0				0				97.03						NA
-80.42	43.65	ELORA RC	6142286	43675.3	2019	7	29	0.29167	19.9		18.5		92		0		19		6				97.05						NA
-80.42	43.65	ELORA RC	6142286	43675.3	2019	7	29	0.33333	23.6		19.7		79		0		22		12				97.02		31				NA
-80.42	43.65	ELORA RC	6142286	43675.4	2019	7	29	0.375	25.6		19.6		70		0		23		18				96.99		33				NA
-80.42	43.65	ELORA RC	6142286	43675.4	2019	7	29	0.41667	26.8		19.7		65		0		22		19				96.96		34				NA
-80.42	43.65	ELORA RC	6142286	43675.5	2019	7	29	0.45833	27.1		19.6		63		0		22		26				96.95		34				NA
-80.42	43.65	ELORA RC	6142286	43675.5	2019	7	29	0.5	27.6		17.9		55		0		24		31				96.92		34				NA

-80.42	43.65	ELORA RC	6142286	43675.5	2019	7	29	0.54167	27.9		18.2		56		0		22		27				96.85		34				NA
-80.42	43.65	ELORA RC	6142286	43675.6	2019	7	29	0.58333	28.5		17.9		53		0		24		26				96.8		34				NA
-80.42	43.65	ELORA RC	6142286	43675.6	2019	7	29	0.625	28		18.4		56		0		21		24				96.76		34				NA
-80.42	43.65	ELORA RC	6142286	43675.7	2019	7	29	0.66667	27.1		19		61		0		21		27				96.77		34				NA
-80.42	43.65	ELORA RC	6142286	43675.7	2019	7	29	0.70833	26.1		19		65		0		21		28				96.76		33				NA
-80.42	43.65	ELORA RC	6142286	43675.8	2019	7	29	0.75	25.7		18.7		65		0		23		24				96.84		32				NA
-80.42	43.65	ELORA RC	6142286	43675.8	2019	7	29	0.79167	20		18		88		0		25		10				96.85		26				NA
-80.42	43.65	ELORA RC	6142286	43675.8	2019	7	29	0.83333	19.6		18.5		94		0.3		31		6				96.83						NA
-80.42	43.65	ELORA RC	6142286	43675.9	2019	7	29	0.875	19.5		18.7		95		0.3		27		4				96.9						NA
-80.42	43.65	ELORA RC	6142286	43675.9	2019	7	29	0.91667	19		18.3		96		0		20		5				96.88						NA
-80.42	43.65	ELORA RC	6142286	43676	2019	7	29	0.95833	19.4		18.7		96		0		22		8				96.86						NA
-80.42	43.65	ELORA RC	6142286	43676	2019	7	30	0	19.5		18.8		95		0		19		10				96.85						NA
-80.42	43.65	ELORA RC	6142286	43676	2019	7	30	0.04167	19.4		19		97		0		17		9				96.82						NA
-80.42	43.65	ELORA RC	6142286	43676.1	2019	7	30	0.08333	19.6		19		97		0		19		10				96.8						NA
-80.42	43.65	ELORA RC	6142286	43676.1	2019	7	30	0.125	19.3		18.6		95		0		20		12				96.82						NA
-80.42	43.65	ELORA RC	6142286	43676.2	2019	7	30	0.16667	19.2		18.6		96		0		19		10				96.8						NA
-80.42	43.65	ELORA RC	6142286	43676.2	2019	7	30	0.20833	19.2		18.7		97		0.5		18		9				96.81						NA
-80.42	43.65	ELORA RC	6142286	43676.3	2019	7	30	0.25	19.3		18.9		98		5.4		23		4				96.87						NA
-80.42	43.65	ELORA RC	6142286	43676.3	2019	7	30	0.29167	19.8		19.3		97		0		26		9				96.9						NA
-80.42	43.65	ELORA RC	6142286	43676.3	2019	7	30	0.33333	20.6		19.1		91		0		28		9				96.94		27				NA
-80.42	43.65	ELORA RC	6142286	43676.4	2019	7	30	0.375	21.5		18.2		82		0		29		8				96.99		28				NA
-80.42	43.65	ELORA RC	6142286	43676.4	2019	7	30	0.41667	22.4		18.7		79		0		29		12				97.02		29				NA
-80.42	43.65	ELORA RC	6142286	43676.5	2019	7	30	0.45833	23.7		17.2		67		0		26		13				97.01		29				NA
-80.42	43.65	ELORA RC	6142286	43676.5	2019	7	30	0.5	24.7		16		58		0		27		16				97.02		29				NA
-80.42	43.65	ELORA RC	6142286	43676.5	2019	7	30	0.54167	26.3		17		57		0		30		17				97.01		32				NA
-80.42	43.65	ELORA RC	6142286	43676.6	2019	7	30	0.58333	26.4		16.5		55		0		26		21				97		31				NA
-80.42	43.65	ELORA RC	6142286	43676.6	2019	7	30	0.625	23.6		17.8		70		0		32		20				97.02		29				NA
-80.42	43.65	ELORA RC	6142286	43676.7	2019	7	30	0.66667	24.7		16.9		62		0		34		18				97.02		30				NA
-80.42	43.65	ELORA RC	6142286	43676.7	2019	7	30	0.70833	24.3		17		63		0		34		17				97.01		30				NA
-80.42	43.65	ELORA RC	6142286	43676.8	2019	7	30	0.75	23.1		16.4		66		0		33		16				97.09		28				NA
-80.42	43.65	ELORA RC	6142286	43676.8	2019	7	30	0.79167	21.9		16.3		70		0		34		12				97.14		27				NA
-80.42	43.65	ELORA RC	6142286	43676.8	2019	7	30	0.83333	19.6		15.7		78		0		32		11				97.21						NA
-80.42	43.65	ELORA RC	6142286	43676.9	2019	7	30	0.875	18		15		83		0		33		10				97.28						NA
-80.42	43.65	ELORA RC	6142286	43676.9	2019	7	30	0.91667	16.9		14.1		84		0		33		9				97.31						NA
-80.42	43.65	ELORA RC	6142286	43677	2019	7	30	0.95833	16.4		13.3		82		0		35		6				97.34						NA
-80.42	43.65	ELORA RC	6142286	43677	2019	7	31	0	15.5		13.4		87		0		34		6				97.38						NA
-80.42	43.65	ELORA RC	6142286	43677	2019	7	31	0.04167	14.9		12.6		86		0		33		6				97.38						NA
-80.42	43.65	ELORA RC	6142286	43677.1	2019	7	31	0.08333	14.7		13.1		90		0		32		8				97.36						NA
-80.42	43.65	ELORA RC	6142286	43677.1	2019	7	31	0.125	13.5		12		91		0		33		7				97.39						NA
-80.42	43.65	ELORA RC	6142286	43677.2	2019	7	31	0.16667	13.1		12		93		0		34		10				97.39						NA
-80.42	43.65	ELORA RC	6142286	43677.2	2019	7	31	0.20833	12.5		11.6		94		0				0				97.43						NA
-80.42	43.65	ELORA RC	6142286	43677.3	2019	7	31	0.25	12.1		11.3		95		0				0				97.46						NA
-80.42	43.65	ELORA RC	6142286	43677.3	2019	7	31	0.29167	14.3		12.3		88		0		31		7				97.51						NA
-80.42	43.65	ELORA RC	6142286	43677.3	2019	7	31	0.33333	16.3		13.1		82		0		31		10				97.55						NA
-80.42	43.65	ELORA RC	6142286	43677.4	2019	7	31	0.375	17		13.3		79		0		34		15				97.6						NA
-80.42	43.65	ELORA RC	6142286	43677.4	2019	7	31	0.41667	18.4		14.4		78		0		36		12				97.61						NA
-80.42	43.65	ELORA RC	6142286	43677.5	2019	7	31	0.45833	20.2		14.2		69		0		36		11				97.6						NA
-80.42	43.65	ELORA RC	6142286	43677.5	2019	7	31	0.5	22.2		13.6		58		0		1		8				97.58		25				NA
-80.42	43.65	ELORA RC	6142286	43677.5	2019	7	31	0.54167	23.7		13.4		52		0		35		9				97.55		27				NA
-80.42	43.65	ELORA RC	6142286	43677.6	2019	7	31	0.58333	24.5		12.7		48		0		33		9				97.54		27				NA
-80.42	43.65	ELORA RC	6142286	43677.6	2019	7	31	0.625	24.2		13.2		50		0		35		10				97.51		27				NA
-80.42	43.65	ELORA RC	6142286	43677.7	2019	7	31	0.66667	24		12.2		47		0		35		12				97.48		26				NA
-80.42	43.65	ELORA RC	6142286	43677.7	2019	7	31	0.70833	24.1		13.2		51		0		36		14				97.48		27				NA
-80.42	43.65	ELORA RC	6142286	43677.8	2019	7	31	0.75	23.9		12.3		48		0		34		11				97.49		26				NA
-80.42	43.65	ELORA RC	6142286	43677.8	2019	7	31	0.79167	22.4		12.4		53		0		35		8				97.52		25				NA
-80.42	43.65	ELORA RC	6142286	43677.8	2019	7	31	0.83333	19.6		12.8		65		0		36		10				97.55						NA
-80.42	43.65	ELORA RC	6142286	43677.9	2019	7	31	0.875	17.4		11.6		69		0		2		12				97.6						NA
-80.42	43.65	ELORA RC	6142286	43677.9	2019	7	31	0.91667	15.9		10.2		69		0		3		11				97.62						NA
-80.42	43.65	ELORA RC	6142286	43678	2019	7	31	0.95833	14.5		9.7		73		0		5		4				97.66						NA

Table S2B. Hourly recorded weather data of the field trial in August 2019

Longitude	Latitude (	Station Na	Climate ID	Date/Tim	Year	Month	Day	Time (LST	Temp (°C)	Temp Flag	Dew Poin	Dew Poin	Rel Hum (	Rel Hum F	Precip. Ar	Precip. Ar	Wind Dir (	Wind Dir	Wind Spd	Wind Spd	Visibility (	Visibility I	Stn Press	Stn Press	Hmdx	Hmdx Flag	Wind Chil	Wind Chil	Weather
-80.42	43.65	ELORA RC	6142286	43678	2019	8	1	0	11.8		9.5		86		0		36		4				97.68						NA
-80.42	43.65	ELORA RC	6142286	43678	2019	8	1	0.04167	13		9		77		0		1		10				97.68						NA
-80.42	43.65	ELORA RC	6142286	43678.1	2019	8	1	0.08333	13.3		9.1		76		0		2		13				97.69						NA
-80.42	43.65	ELORA RC	6142286	43678.1	2019	8	1	0.125	13.3		10.2		82		0		2		13				97.7						NA
-80.42	43.65	ELORA RC	6142286	43678.2	2019	8	1	0.16667	13.4		10.8		85		0		2		13				97.69						NA
-80.42	43.65	ELORA RC	6142286	43678.2	2019	8	1	0.20833	13.5		10.9		84		0		4		10				97.71						NA
-80.42	43.65	ELORA RC	6142286	43678.3	2019	8	1	0.25	14.1		11.2		83		0		3		10				97.74						NA
-80.42	43.65	ELORA RC	6142286	43678.3	2019	8	1	0.29167	16.8		12.1		74		0		4		11				97.77						NA
-80.42	43.65	ELORA RC	6142286	43678.3	2019	8	1	0.33333	19.5		10.9		58		0		7		12				97.83						NA
-80.42	43.65	ELORA RC	6142286	43678.4	2019	8	1	0.375	21.5		11.1		52		0		10		14				97.84						NA
-80.42	43.65	ELORA RC	6142286	43678.4	2019	8	1	0.41667	22.4		8.9		42		0		8		15				97.85						NA
-80.42	43.65	ELORA RC	6142286	43678.5	2019	8	1	0.45833	22.9		9.6		43		0		9		10				97.84						NA
-80.42	43.65	ELORA RC	6142286	43678.5	2019	8	1	0.5	23.8		8.9		39		0		10		8				97.84		25				NA
-80.42	43.65	ELORA RC	6142286	43678.5	2019	8	1	0.54167	24.7		9.9		39		0		11		7				97.84		26				NA
-80.42	43.65	ELORA RC	6142286	43678.6	2019	8	1	0.58333	24.7		9.8		39		0		11		6				97.78		26				NA
-80.42	43.65	ELORA RC	6142286	43678.6	2019	8	1	0.625	24.7		9.5		38		0		18		9				97.75		26				NA
-80.42	43.65	ELORA RC	6142286	43678.7	2019	8	1	0.66667	25.2		10.3		39		0		14		10				97.69		27				NA
-80.42	43.65	ELORA RC	6142286	43678.7	2019	8	1	0.70833	25		10.4		40		0		11		6				97.65		26				NA
-80.42	43.65	ELORA RC	6142286	43678.8	2019	8	1	0.75	24.6		9.4		38		0		17		4				97.62		26				NA
-80.42	43.65	ELORA RC	6142286	43678.8	2019	8	1	0.79167	23.3		10.5		44		0		11		4				97.63		25				NA
-80.42	43.65	ELORA RC	6142286	43678.8	2019	8	1	0.83333	19.1		12.5		66		0		8		7				97.66						NA
-80.42	43.65	ELORA RC	6142286	43678.9	2019	8	1	0.875	18.2		11.8		66		0		4		10				97.75						NA
-80.42	43.65	ELORA RC	6142286	43678.9	2019	8	1	0.91667	16.3		10.6		69		0		5		8				97.74						NA
-80.42	43.65	ELORA RC	6142286	43679	2019	8	1	0.95833	15.2		10.3		72		0		3		6				97.76						NA
-80.42	43.65	ELORA RC	6142286	43679	2019	8	2	0	13.8		9.9		77		0				0				97.78						NA
-80.42	43.65	ELORA RC	6142286	43679	2019	8	2	0.04167	13.9		9.4		74		0		4		6				97.76						NA
-80.42	43.65	ELORA RC	6142286	43679.1	2019	8	2	0.08333	13.8		9.2		74		0		5		5				97.74						NA
-80.42	43.65	ELORA RC	6142286	43679.1	2019	8	2	0.125	12.2		8.4		77		0		35		4				97.73						NA
-80.42	43.65	ELORA RC	6142286	43679.2	2019	8	2	0.16667	13		9.4		79		0		5		5				97.73						NA
-80.42	43.65	ELORA RC	6142286	43679.2	2019	8	2	0.20833	12.1		9.2		83		0		3		5				97.74						NA
-80.42	43.65	ELORA RC	6142286	43679.3	2019	8	2	0.25	13.9		10.4		79		0		3		9				97.75						NA
-80.42	43.65	ELORA RC	6142286	43679.3	2019	8	2	0.29167	16.9		10.9		68		0		5		5				97.77						NA
-80.42	43.65	ELORA RC	6142286	43679.3	2019	8	2	0.33333	20		10.1		53		0		5		8				97.76						NA
-80.42	43.65	ELORA RC	6142286	43679.4	2019	8	2	0.375	21.8		8		41		0		10		8				97.75						NA
-80.42	43.65	ELORA RC	6142286	43679.4	2019	8	2	0.41667	23.3		9.4		41		0		16		5				97.77						NA
-80.42	43.65	ELORA RC	6142286	43679.5	2019	8	2	0.45833	23.7		8.7		38		0		6		6				97.73						NA
-80.42	43.65	ELORA RC	6142286	43679.5	2019	8	2	0.5	24.6		9.2		38		0		12		8				97.68		26				NA
-80.42	43.65	ELORA RC	6142286	43679.5	2019	8	2	0.54167	25		8.9		36		0		13		5				97.66		26				NA
-80.42	43.65	ELORA RC	6142286	43679.6	2019	8	2	0.58333	25.7		9.4		36		0		19		6				97.62		27				NA
-80.42	43.65	ELORA RC	6142286	43679.6	2019	8	2	0.625	26		9.3		35		0		25		6				97.56		27				NA
-80.42	43.65	ELORA RC	6142286	43679.7	2019	8	2	0.66667	26.1		9.9		36		0		14		3				97.51		27				NA
-80.42	43.65	ELORA RC	6142286	43679.7	2019	8	2	0.70833	26.6		9.8		35		0		33		4				97.46		28				NA
-80.42	43.65	ELORA RC	6142286	43679.8	2019	8	2	0.75	25.5		10.5		39		0		32		11				97.44		27				NA
-80.42	43.65	ELORA RC	6142286	43679.8	2019	8	2	0.79167	23.7		11.2		46		0		34		6				97.46		26				NA
-80.42	43.65	ELORA RC	6142286	43679.8	2019	8	2	0.83333	20.2		12.6		62		0		15		8				97.49						NA
-80.42	43.65	ELORA RC	6142286	43679.9	2019	8	2	0.875	20.2		10.9		55		0		1		7				97.55						NA
-80.42	43.65	ELORA RC	6142286	43679.9	2019	8	2	0.91667	18.1		10.1		60		0		1		9				97.57						NA
-80.42	43.65	ELORA RC	6142286	43680	2019	8	2	0.95833	18.2		11.2		64		0		3		13				97.53						NA
-80.42	43.65	ELORA RC	6142286	43680	2019	8	3	0	15.4		11		75		0		5		8				97.48						NA
-80.42	43.65	ELORA RC	6142286	43680	2019	8	3	0.04167	15		11.3		78		0		6		7				97.47						NA
-80.42	43.65	ELORA RC	6142286	43680.1	2019	8	3	0.08333	12.4		10.8		90		0				0				97.45						NA
-80.42	43.65	ELORA RC	6142286	43680.1	2019	8	3	0.125	13.2		10.3		82		0		2		5				97.41						NA
-80.42	43.65	ELORA RC	6142286	43680.2	2019	8	3	0.16667	11.3		10.3		94		0		1		3				97.4						NA
-80.42	43.65	ELORA RC	6142286	43680.2	2019	8	3	0.20833	11		9.7		92		0		36		4				97.43						NA
-80.42	43.65	ELORA RC	6142286	43680.3	2019	8	3	0.25	11.2		9.8		92		0		36		3				97.45						NA
-80.42	43.65	ELORA RC	6142286	43680.3	2019	8	3	0.29167	15.3		13.1		86		0				0				97.47						NA
-80.42	43.65	ELORA RC	6142286	43680.3	2019	8	3	0.33333	20.6		12.2		59		0		31		8				97.47						NA
-80.42	43.65	ELORA RC	6142286	43680.4	2019	8	3	0.375	22.8		8.7		41		0		31		7				97.44						NA
-80.42	43.65	ELORA RC	6142286	43680.4	2019	8	3	0.41667	24.6		10.9		42		0		29		10				97.43		26				NA
-80.42	43.65	ELORA RC	6142286	43680.5	2019	8	3	0.45833	25.4		11.8		43		0		28		10				97.37		28				NA
-80.42	43.65	ELORA RC	6142286	43680.5	2019	8	3	0.5	27		13.6		44		0		29		11				97.33		30				NA
-80.42	43.65	ELORA RC	6142286	43680.5	2019	8	3	0.54167	26.9		12.5		41		0		30		13				97.3		29				NA
-80.42	43.65	ELORA RC	6142286	43680.6	2019	8	3	0.58333	26.9		13.6		44		0		32		10				97.23		30				NA
-80.42	43.65	ELORA RC	6142286	43680.6	2019	8	3	0.625	26.9		14.2		46		0		28		16				97.2		30				NA
-80.42	43.65	ELORA RC	6142286	43680.7	2019	8	3	0.66667	27.7		14.2		44		0		29		18				97.16		31				NA
-80.42	43.65	ELORA RC	6142286	43680.7	2019	8	3	0.70833	26.8		14.4		47		0		31		17				97.15		30				NA
-80.42	43.65	ELORA RC	6142286	43680.8	2019	8	3	0.75	26		14.2		48		0		31		18				97.13		29				NA
-80.42	43.65	ELORA RC	6142286	43680.8	2019	8	3	0.79167	24.1		14.1		54		0		30		12				97.16		28				NA
-80.42	43.65	ELORA RC	6142286	43680.8	2019	8	3	0.83333	20.9		13.8		64		0		32		9				97.19						NA
-80.42	43.65	ELORA RC	6142286	43680.9	2019	8	3	0.875	19.6		14		70		0		32		7				97.24						NA
-80.42	43.65	ELORA RC	6142286	43680.9	2019	8	3	0.91667	17.9		13.9		78		0		31		6				97.24						NA
-80.42	43.65	ELORA RC	6142286	43681	2019	8	3	0.95833	17.3		14		81		0		32		4				97.23						NA
-80.42	43.65	ELORA RC	6142286	43681	2019	8	4	0	15.7		13.3		85		0		35		4				97.24						NA
-80.42	43.65	ELORA RC	6142286	43681	2019	8	4	0.04167	15.5		14		91		0		21		3				97.26						NA
-80.42	43.65	ELORA RC	6142286	43681.1	2019	8	4	0.08333	16.2		14.7		91		0		1		7				97.22						NA
-80.42	43.65	ELORA RC	6142286	43681.1	2019	8	4	0.125	14.3		13.6		96		0		35		6				97.24						NA
-80.42	43.65	ELORA RC	6142286	43681.2	2019	8	4	0.16667	16.8		15.3		91		0		2		11				97.22						NA
-80.42	43.65	ELORA RC	6142286	43681.2	2019	8	4	0.20833	16.7		15.1		91		0		3		10				97.25						NA
-80.42	43.65	ELORA RC	6142286	43681.3	2019	8	4	0.25	16.8		14.8		88		0		3		9				97.3						NA
-80.42	43.65	ELORA RC	6142286	43681.3	2019	8	4	0.29167	18.4		14.7		79		0		5		12				97.33						NA
-80.42	43.65	ELORA RC	6142286	43681.3	2019	8	4	0.33333	20.4		14		67		0		7		12				97.34						NA
-80.42	43.65	ELORA RC	6142286	43681.4	2019	8	4	0.375	21.7		14.1		62		0		7		10				97.35		25				NA
-80.42	43.65	ELORA RC	6142286	43681.4	2019	8	4	0.41667	23.2		14.8		59		0		9		6				97.37		27				NA
-80.42	43.65	ELORA RC	6142286	43681.5	2019	8	4	0.45833	23.7		11.9		47		0		5		8				97.34		26				NA
-80.42	43.65	ELORA RC	6142286	43681.5	2019	8	4	0.5	24		11.6		46		0		2		10				97.31		26				NA
-80.42	43.65	ELORA RC	6142286	43681.5	2019	8	4	0.54167	24.6		12.8		48		0		5		7				97.3		27				NA
-80.42	43.65	ELORA RC	6142286	43681.6	2019	8	4	0.58333	24.5		11.8		45		0		35		8				97.27		27				NA
-80.42	43.65	ELORA RC	6142286	43681.6	2019	8	4	0.625	26		12.1		42		0		4		5				97.24		28				NA
-80.42	43.65	ELORA RC	6142286	43681.7	2019	8	4	0.66667	25.1		12.5		45		0		3		9				97.21		28				NA
-80.42	43.65	ELORA RC	6142286	43681.7	2019	8	4	0.70833	26.1		11.5		40		0		36		3				97.2		28				NA
-80.42	43.65	ELORA RC	6142286	43681.8	2019	8	4	0.75	24.9		11.8		44		0		1		7				97.17		27				NA
-80.42	43.65	ELORA RC	6142286	43681.8	2019	8	4	0.79167	23		12.9		53		0		5		9				97.19		26				NA
-80.42	43.65	ELORA RC	6142286	43681.8	2019	8	4	0.83333	20.2		11.2		56		0		8		10				97.22						NA
-80.42	43.65	ELORA RC	6142286	43681.9	2019	8	4	0.875	18.4		11.4		63		0		8		7				97.27						NA
-80.42	43.65	ELORA RC	6142286	43681.9	2019	8	4	0.91667	16.4		10.6		68		0				0				97.26						NA
-80.42	43.65	ELORA RC	6142286	43682	2019	8	4	0.95833	14.7		10.8		77		0		6		5				97.27						NA
-80.42	43.65	ELORA RC	6142286	43682	2019	8	5	0	14.2		10.4		78		0				0				97.27						NA
-80.42	43.65	ELORA RC	6142286	43682	2019	8	5	0.04167	12.8		9.4		80		0		5		3				97.25						NA
-80.42	43.65	ELORA RC	6142286	43682.1	2019	8	5	0.08333	12.4		9.8		84		0		7		5				97.23						NA
-80.42	43.65	ELORA RC	6142286	43682.1	2019	8	5	0.125	12.5		9.6		83		0				0				97.24						NA
-80.42	43.65	ELORA RC	6142286	43682.2	2019	8	5	0.16667	12.3		9.9		86		0		3		3				97.22						NA
-80.42	43.65	ELORA RC	6142286	43682.2	2019	8	5	0.20833	13		10.6		85		0		2		3				97.21						NA
-80.42	43.65	ELORA RC	6142286	43682.3	2019	8	5	0.25	13.6		10.9		84		0		5		3				97.19						NA
-80.42	43.65	ELORA RC	6142286	43682.3	2019	8	5	0.29167	17.3		12.5		73		0		8		3				97.19						NA
-80.42	43.65	ELORA RC	6142286	43682.3	2019	8	5	0.33333	20.1		15.5		75		0		20		5				97.17						NA
-80.42	43.65	ELORA RC	6142286	43682.4	2019	8	5	0.375	22.3		16.9		71		0		22		10				97.18		28				NA
-80.42	43.65	ELORA RC	6142286	43682.4	2019	8	5	0.41667	23.6		16.2		63		0		22		8				97.16		28				NA
-80.42	43.65	ELORA RC	6142286	43682.5	2019	8	5	0.45833	25.5		16.1		56		0		21		14				97.11		30				NA
-80.42	43.65	ELORA RC	6142286	43682.5	2019	8	5	0.5	25.9		15.3		52		0		23		14				97.07		30				NA
-80.42	43.65	ELORA RC	6142286	43682.5	2019	8	5	0.54167	27.3		15.5		49		0		23		13				97.03		32				NA
-80.42	43.65	ELORA RC	6142286	43682.6	2019	8	5	0.58333	27.3		16.1		50		0		21		14				96.96		32				NA
-80.42	43.65	ELORA RC	6142286	43682.6	2019	8	5	0.625	27.2		15.6		49		0		22		15				96.91		32				NA
-80.42	43.65	ELORA RC	6142286	43682.7	2019	8	5	0.66667	26.2		17.8		60		0		20		14				96.88		32				NA
-80.42	43.65	ELORA RC	6142286	43682.7	2019	8	5	0.70833	25.4		18.1		64		0		21		19				96.82		31				NA
-80.42	43.65	ELORA RC	6142286	43682.8	2019	8	5	0.75	25.1		17.6		63		0		25		14				96.77		31				NA
-80.42	43.65	ELORA RC	6142286	43682.8	2019	8	5	0.79167	22.4		16.6		70		0		24		10				96.77		27				NA
-80.42	43.65	ELORA RC	6142286	43682.8	2019	8	5	0.83333	19.8		16.6		82		0		23		9				96.77						NA
-80.42	43.65	ELORA RC	6142286	43682.9	2019	8	5	0.875	19.7		17.2		85		0		25		5				96.81						NA
-80.42	43.65	ELORA RC	6142286	43682.9	2019	8	5	0.91667	19.2		17.1		88		0				0				96.76						NA
-80.42	43.65	ELORA RC	6142286	43683	2019	8	5	0.95833	17.9		17		94		0				0				96.75						NA
-80.42	43.65	ELORA RC	6142286	43683	2019	8	6	0	16.7		15.7		94		0				0				96.7						NA
-80.42	43.65	ELORA RC	6142286	43683	2019	8	6	0.04167	17.3		16.8		97		0		14		6				96.64						NA
-80.42	43.65	ELORA RC	6142286	43683.1	2019	8	6	0.08333	16.2		15.8		97		0				0				96.58						NA
-80.42	43.65	ELORA RC	6142286	43683.1	2019	8	6	0.125	15.6		15.3		98		0		20		4				96.53						NA
-80.42	43.65	ELORA RC	6142286	43683.2	2019	8	6	0.16667	15.9		15.6		98		0		28		3				96.49						NA
-80.42	43.65	ELORA RC	6142286	43683.2	2019	8	6	0.20833	15		14.7		98		0				0				96.47						NA
-80.42	43.65	ELORA RC	6142286	43683.3	2019	8	6	0.25	17.3		17		99		0		23		5				96.46						NA
-80.42	43.65	ELORA RC	6142286	43683.3	2019	8	6	0.29167	20.1		19		93		0		23		10				96.45		27				NA
-80.42	43.65	ELORA RC	6142286	43683.3	2019	8	6	0.33333	20.8		18.9		89		0		34		6				96.51		27				NA
-80.42	43.65	ELORA RC	6142286	43683.4	2019	8	6	0.375	18.8		17.9		95		0.5		24		5				96.51						NA
-80.42	43.65	ELORA RC	6142286	43683.4	2019	8	6	0.41667	21.3		19.3		88		0		27		11				96.46		28				NA
-80.42	43.65	ELORA RC	6142286	43683.5	2019	8	6	0.45833	22		19.1		84		0		26		13				96.5		29				NA
-80.42	43.65	ELORA RC	6142286	43683.5	2019	8	6	0.5	22.7		18.5		77		0		26		16				96.46		29				NA
-80.42	43.65	ELORA RC	6142286	43683.5	2019	8	6	0.54167	23.4		17.9		71		0		30		15				96.4		29				NA

-80.42	43.65	ELORA RC	6142286	43683.6	2019	8	6	0.58333	23.4		18.2		73		0		26		13				96.4		30				NA
-80.42	43.65	ELORA RC	6142286	43683.6	2019	8	6	0.625	23.7		17.8		70		0		30		15				96.4		30				NA
-80.42	43.65	ELORA RC	6142286	43683.7	2019	8	6	0.66667	23.7		17.4		68		0		29		12				96.43		29				NA
-80.42	43.65	ELORA RC	6142286	43683.7	2019	8	6	0.70833	23.2		18.3		74		0		28		11				96.42		29				NA
-80.42	43.65	ELORA RC	6142286	43683.8	2019	8	6	0.75	22.4		17.8		75		0		30		14				96.45		28				NA
-80.42	43.65	ELORA RC	6142286	43683.8	2019	8	6	0.79167	21.4		18		81		0		30		6				96.47		27				NA
-80.42	43.65	ELORA RC	6142286	43683.8	2019	8	6	0.83333	20.4		18.3		88		0		32		4				96.48		27				NA
-80.42	43.65	ELORA RC	6142286	43683.9	2019	8	6	0.875	19.1		17.4		90		0		30		5				96.49						NA
-80.42	43.65	ELORA RC	6142286	43683.9	2019	8	6	0.91667	18.6		17.6		94		0				0				96.51						NA
-80.42	43.65	ELORA RC	6142286	43684	2019	8	6	0.95833	17.2		16.5		96		0		29		4				96.51						NA
-80.42	43.65	ELORA RC	6142286	43684	2019	8	7	0	17.8		16.9		94		0		1		5				96.52						NA
-80.42	43.65	ELORA RC	6142286	43684	2019	8	7	0.04167	17.4		16.6		95		0		1		4				96.47						NA
-80.42	43.65	ELORA RC	6142286	43684.1	2019	8	7	0.08333	16.2		15.7		97		0		31		4				96.5						NA
-80.42	43.65	ELORA RC	6142286	43684.1	2019	8	7	0.125	16.8		16.3		97		0		35		4				96.5						NA
-80.42	43.65	ELORA RC	6142286	43684.2	2019	8	7	0.16667	16.6		15.8		95		0		35		6				96.5						NA
-80.42	43.65	ELORA RC	6142286	43684.2	2019	8	7	0.20833	15.6		15.1		97		0				0				96.54						NA
-80.42	43.65	ELORA RC	6142286	43684.3	2019	8	7	0.25	15.3		15		98		0		35		4				96.57						NA
-80.42	43.65	ELORA RC	6142286	43684.3	2019	8	7	0.29167	17.1		15.6		91		0		1		10				96.54						NA
-80.42	43.65	ELORA RC	6142286	43684.3	2019	8	7	0.33333	19.7		16		80		0		2		4				96.56						NA
-80.42	43.65	ELORA RC	6142286	43684.4	2019	8	7	0.375	21		15.5		70		0		2		6				96.56		25				NA
-80.42	43.65	ELORA RC	6142286	43684.4	2019	8	7	0.41667	22.7		15.3		63		0		36		8				96.53		27				NA
-80.42	43.65	ELORA RC	6142286	43684.5	2019	8	7	0.45833	23.9		16.4		63		0		32		10				96.55		29				NA
-80.42	43.65	ELORA RC	6142286	43684.5	2019	8	7	0.5	24.1		16.6		63		0		34		12				96.53		29				NA
-80.42	43.65	ELORA RC	6142286	43684.5	2019	8	7	0.54167	24.8		15.4		56		0		33		14				96.53		29				NA
-80.42	43.65	ELORA RC	6142286	43684.6	2019	8	7	0.58333	25.5		14		49		0		31		13				96.51		29				NA
-80.42	43.65	ELORA RC	6142286	43684.6	2019	8	7	0.625	25		13.5		49		0		29		16				96.46		28				NA
-80.42	43.65	ELORA RC	6142286	43684.7	2019	8	7	0.66667	25.2		14.4		51		0		31		12				96.43		29				NA
-80.42	43.65	ELORA RC	6142286	43684.7	2019	8	7	0.70833	25.5		13.6		48		0		29		11				96.41		29				NA
-80.42	43.65	ELORA RC	6142286	43684.8	2019	8	7	0.75	24.6		13.1		49		0		30		13				96.41		27				NA
-80.42	43.65	ELORA RC	6142286	43684.8	2019	8	7	0.79167	22.5		14.1		59		0		30		10				96.41		26				NA
-80.42	43.65	ELORA RC	6142286	43684.8	2019	8	7	0.83333	20.3		13		63		0		29		5				96.47						NA
-80.42	43.65	ELORA RC	6142286	43684.9	2019	8	7	0.875	17.4		12.2		71		0				0				96.53						NA
-80.42	43.65	ELORA RC	6142286	43684.9	2019	8	7	0.91667	15.1		12.2		83		0		4		3				96.47						NA
-80.42	43.65	ELORA RC	6142286	43685	2019	8	7	0.95833	14		12.2		89		0				0				96.43						NA
-80.42	43.65	ELORA RC	6142286	43685	2019	8	8	0	13.5		11.5		88		0				0				96.41						NA
-80.42	43.65	ELORA RC	6142286	43685	2019	8	8	0.04167	12.5		11.2		92		0		28		4				96.4						NA
-80.42	43.65	ELORA RC	6142286	43685.1	2019	8	8	0.08333	13.3		11.6		90		0		9		3				96.35						NA
-80.42	43.65	ELORA RC	6142286	43685.1	2019	8	8	0.125	13.1		11.2		89		0				0				96.27						NA
-80.42	43.65	ELORA RC	6142286	43685.2	2019	8	8	0.16667	12.8		11.4		91		0		13		3				96.25						NA
-80.42	43.65	ELORA RC	6142286	43685.2	2019	8	8	0.20833	12.6		11.2		91		0				0				96.22						NA
-80.42	43.65	ELORA RC	6142286	43685.3	2019	8	8	0.25	14.3		13.3		94		0		8		3				96.16						NA
-80.42	43.65	ELORA RC	6142286	43685.3	2019	8	8	0.29167	17.1		16.3		95		0		24		5				96.17						NA
-80.42	43.65	ELORA RC	6142286	43685.3	2019	8	8	0.33333	18.1		17.2		94		0.9		24		8				96.13						NA
-80.42	43.65	ELORA RC	6142286	43685.4	2019	8	8	0.375	20		17.4		85		0.1		24		16				96.02		26				NA
-80.42	43.65	ELORA RC	6142286	43685.4	2019	8	8	0.41667	20.4		17.3		83		0		25		14				96.1		26				NA
-80.42	43.65	ELORA RC	6142286	43685.5	2019	8	8	0.45833	19.8		16.9		83		0		26		20				96.08						NA
-80.42	43.65	ELORA RC	6142286	43685.5	2019	8	8	0.5	21.1		17.7		81		0.3		27		19				96.08		27				NA
-80.42	43.65	ELORA RC	6142286	43685.5	2019	8	8	0.54167	20.2		18.4		90		1.7		27		14				96.12		27				NA
-80.42	43.65	ELORA RC	6142286	43685.6	2019	8	8	0.58333	21.9		19.1		84		4.2		30		22				96.16		29				NA
-80.42	43.65	ELORA RC	6142286	43685.6	2019	8	8	0.625	22.5		16		67		0		30		30				96.18		27				NA
-80.42	43.65	ELORA RC	6142286	43685.7	2019	8	8	0.66667	22.5		13.7		58		0		30		30				96.24		26				NA
-80.42	43.65	ELORA RC	6142286	43685.7	2019	8	8	0.70833	22.7		12.5		53		0		30		25				96.26		25				NA
-80.42	43.65	ELORA RC	6142286	43685.8	2019	8	8	0.75	21.7		11.6		53		0		29		21				96.28						NA
-80.42	43.65	ELORA RC	6142286	43685.8	2019	8	8	0.79167	20.3		11.8		58		0		30		14				96.32						NA
-80.42	43.65	ELORA RC	6142286	43685.8	2019	8	8	0.83333	18.4		12.5		68		0		31		5				96.34						NA
-80.42	43.65	ELORA RC	6142286	43685.9	2019	8	8	0.875	16.7		13.2		79		0		32		11				96.43						NA
-80.42	43.65	ELORA RC	6142286	43685.9	2019	8	8	0.91667	15		12.3		84		0		30		8				96.46						NA
-80.42	43.65	ELORA RC	6142286	43686	2019	8	8	0.95833	14.5		12.6		88		0		28		7				96.49						NA
-80.42	43.65	ELORA RC	6142286	43686	2019	8	9	0	13.3		12.1		93		0		29		8				96.53						NA
-80.42	43.65	ELORA RC	6142286	43686	2019	8	9	0.04167	12.4		11.5		94		0		28		9				96.53						NA
-80.42	43.65	ELORA RC	6142286	43686.1	2019	8	9	0.08333	11.4		10.6		95		0		27		9				96.52						NA
-80.42	43.65	ELORA RC	6142286	43686.1	2019	8	9	0.125	11.2		10.6		96		0		27		9				96.47						NA
-80.42	43.65	ELORA RC	6142286	43686.2	2019	8	9	0.16667	10		9.3		95		0		23		9				96.48						NA
-80.42	43.65	ELORA RC	6142286	43686.2	2019	8	9	0.20833	10		9.6		97		0		25		7				96.53						NA
-80.42	43.65	ELORA RC	6142286	43686.3	2019	8	9	0.25	10.4		10		97		0		24		8				96.59						NA
-80.42	43.65	ELORA RC	6142286	43686.3	2019	8	9	0.29167	13.5		12.2		92		0		25		8				96.6						NA
-80.42	43.65	ELORA RC	6142286	43686.3	2019	8	9	0.33333	16.3		13.4		83		0		25		11				96.63						NA
-80.42	43.65	ELORA RC	6142286	43686.4	2019	8	9	0.375	19.3		12.7		65		0		29		17				96.63						NA
-80.42	43.65	ELORA RC	6142286	43686.4	2019	8	9	0.41667	21.1		11.6		55		0		29		21				96.65						NA
-80.42	43.65	ELORA RC	6142286	43686.5	2019	8	9	0.45833	20.4		10.7		54		0		27		24				96.64						NA
-80.42	43.65	ELORA RC	6142286	43686.5	2019	8	9	0.5	21.5		11.6		53		0		28		28				96.62						NA
-80.42	43.65	ELORA RC	6142286	43686.5	2019	8	9	0.54167	21.9		11		50		0		27		30				96.6						NA
-80.42	43.65	ELORA RC	6142286	43686.6	2019	8	9	0.58333	22.2		11.2		50		0		29		31				96.58						NA
-80.42	43.65	ELORA RC	6142286	43686.6	2019	8	9	0.625	22.5		12.4		53		0		30		25				96.59		25				NA
-80.42	43.65	ELORA RC	6142286	43686.7	2019	8	9	0.66667	21		10.8		52		0		27		23				96.59						NA
-80.42	43.65	ELORA RC	6142286	43686.7	2019	8	9	0.70833	22.1		11.7		52		0		29		22				96.57						NA
-80.42	43.65	ELORA RC	6142286	43686.8	2019	8	9	0.75	20.5		11.8		58		0		31		21				96.62						NA
-80.42	43.65	ELORA RC	6142286	43686.8	2019	8	9	0.79167	19.7		11.2		58		0		30		20				96.66						NA
-80.42	43.65	ELORA RC	6142286	43686.8	2019	8	9	0.83333	16.1		11.4		74		0		31		11				96.7						NA
-80.42	43.65	ELORA RC	6142286	43686.9	2019	8	9	0.875	14.8		11.4		80		0		29		8				96.78						NA
-80.42	43.65	ELORA RC	6142286	43686.9	2019	8	9	0.91667	13.4		10.8		85		0		28		11				96.78						NA
-80.42	43.65	ELORA RC	6142286	43687	2019	8	9	0.95833	12.3		10.4		88		0		30		9				96.8						NA
-80.42	43.65	ELORA RC	6142286	43687	2019	8	10	0	12.9		10.8		87		0		30		12				96.81						NA
-80.42	43.65	ELORA RC	6142286	43687	2019	8	10	0.04167	11.7		10.1		90		0		30		8				96.82						NA
-80.42	43.65	ELORA RC	6142286	43687.1	2019	8	10	0.08333	10.7		9.8		94		0		27		6				96.83						NA
-80.42	43.65	ELORA RC	6142286	43687.1	2019	8	10	0.125	10.1		9.4		95		0		28		8				96.85						NA
-80.42	43.65	ELORA RC	6142286	43687.2	2019	8	10	0.16667	9.8		9.1		95		0		28		9				96.89						NA
-80.42	43.65	ELORA RC	6142286	43687.2	2019	8	10	0.20833	9.8		9.4		97		0				0				96.93						NA
-80.42	43.65	ELORA RC	6142286	43687.3	2019	8	10	0.25	10		9.4		96		0		32		4				96.95						NA
-80.42	43.65	ELORA RC	6142286	43687.3	2019	8	10	0.29167	12.8		11.5		92		0		26		6				97						NA
-80.42	43.65	ELORA RC	6142286	43687.3	2019	8	10	0.33333	15.9		13.1		84		0		30		13				97.04						NA
-80.42	43.65	ELORA RC	6142286	43687.4	2019	8	10	0.375	17.7		13.3		76		0		27		19				97.06						NA
-80.42	43.65	ELORA RC	6142286	43687.4	2019	8	10	0.41667	19		11.4		61		0		29		21				97.07						NA
-80.42	43.65	ELORA RC	6142286	43687.5	2019	8	10	0.45833	20		10.7		55		0		28		20				97.05						NA
-80.42	43.65	ELORA RC	6142286	43687.5	2019	8	10	0.5	19.8		10.4		55		0		30		25				97.04						NA
-80.42	43.65	ELORA RC	6142286	43687.5	2019	8	10	0.54167	20.6		8.3		45		0		30		29				97.03						NA
-80.42	43.65	ELORA RC	6142286	43687.6	2019	8	10	0.58333	20.7		9.6		49		0		31		24				97.04						NA
-80.42	43.65	ELORA RC	6142286	43687.6	2019	8	10	0.625	21.7		9.6		46		0		32		24				97.03						NA
-80.42	43.65	ELORA RC	6142286	43687.7	2019	8	10	0.66667	20.4		8.7		47		0		32		27				97.03						NA
-80.42	43.65	ELORA RC	6142286	43687.7	2019	8	10	0.70833	20.6		7.9		44		0		30		27				97.04						NA
-80.42	43.65	ELORA RC	6142286	43687.8	2019	8	10	0.75	19.7		7.9		46		0		32		20				97.06						NA
-80.42	43.65	ELORA RC	6142286	43687.8	2019	8	10	0.79167	18.2		8.1		52		0		32		16				97.13						NA
-80.42	43.65	ELORA RC	6142286	43687.8	2019	8	10	0.83333	15.8		8.7		63		0		31		7				97.16						NA
-80.42	43.65	ELORA RC	6142286	43687.9	2019	8	10	0.875	14		8.7		71		0		31		5				97.19						NA
-80.42	43.65	ELORA RC	6142286	43687.9	2019	8	10	0.91667	11.9		8.6		80		0				0				97.21						NA
-80.42	43.65	ELORA RC	6142286	43688	2019	8	10	0.95833	10		8.2		89		0				0				97.21						NA
-80.42	43.65	ELORA RC	6142286	43688	2019	8	11	0	9.9		8.4		90		0				0				97.26						NA
-80.42	43.65	ELORA RC	6142286	43688	2019	8	11	0.04167	9		7.3		89		0				0				97.28						NA
-80.42	43.65	ELORA RC	6142286	43688.1	2019	8	11	0.08333	9.4		8.4		94		0		30		4				97.29						NA
-80.42	43.65	ELORA RC	6142286	43688.1	2019	8	11	0.125	9.6		9		96		0		31		3				97.3						NA
-80.42	43.65	ELORA RC	6142286	43688.2	2019	8	11	0.16667	9.1		8.5		96		0				0				97.32						NA
-80.42	43.65	ELORA RC	6142286	43688.2	2019	8	11	0.20833	9.2		8.4		95		0		32		3				97.32						NA
-80.42	43.65	ELORA RC	6142286	43688.3	2019	8	11	0.25	10		8.7		92		0		30		5				97.38						NA
-80.42	43.65	ELORA RC	6142286	43688.3	2019	8	11	0.29167	13.8		10.7		82		0				0				97.41						NA
-80.42	43.65	ELORA RC	6142286	43688.3	2019	8	11	0.33333	17.2		12.1		72		0		24		3				97.43						NA
-80.42	43.65	ELORA RC	6142286	43688.4	2019	8	11	0.375	20.7		12.7		60		0		26		16				97.41						NA
-80.42	43.65	ELORA RC	6142286	43688.4	2019	8	11	0.41667	22.4		12.4		53		0		26		14				97.43		25				NA
-80.42	43.65	ELORA RC	6142286	43688.5	2019	8	11	0.45833	22.9		11.9		50		0		26		15				97.4		25				NA
-80.42	43.65	ELORA RC	6142286	43688.5	2019	8	11	0.5	23.8		12.5		49		0		28		15				97.37		26				NA
-80.42	43.65	ELORA RC	6142286	43688.5	2019	8	11	0.54167	24.3		12.2		47		0		26		19				97.33		27				NA
-80.42	43.65	ELORA RC	6142286	43688.6	2019	8	11	0.58333	24.3		12.5		48		0		26		19				97.31		27				NA
-80.42	43.65	ELORA RC	6142286	43688.6	2019	8	11	0.625	25.3		12.2		44		0		27		15				97.28		28				NA
-80.42	43.65	ELORA RC	6142286	43688.7	2019	8	11	0.66667	25.4		10.6		40		0		27		13				97.21		27				NA
-80.42	43.65	ELORA RC	6142286	43688.7	2019	8	11	0.70833	25.5		11.7		42		0		24		16				97.2		28				NA
-80.42	43.65	ELORA RC	6142286	43688.8	2019	8	11	0.75	24.3		12.2		47		0		26		14				97.18		27				NA
-80.42	43.65	ELORA RC	6142286	43688.8	2019	8	11	0.79167	22.2		12.5		54		0		32		7				97.15		25				NA
-80.42	43.65	ELORA RC	6142286	43688.8	2019	8	11	0.83333	19.2		12.9		67		0		21		9				97.14						NA
-80.42	43.65	ELORA RC	6142286	43688.9	2019	8	11	0.875	18.1		13.3		74		0		22		5				97.16						NA
-80.42	43.65	ELORA RC	6142286	43688.9	2019	8	11	0.91667	16.8		13.9		83		0				0				97.14						NA
-80.42	43.65	ELORA RC	6142286	43689	2019	8	11	0.95833	16.4		14.3		87		0				0				97.12						NA
-80.42	43.65	ELORA RC	6142286	43689	2019	8	12	0	16.3		14.4		88		0				0				97.1						NA
-80.42	43.65	ELORA RC	6142286	43689	2019	8	12	0.04167	16.7		14.7		88		0				0				97.09						NA
-80.42	43.65	ELORA RC	6142286	43689.1	2019	8	12	0.08333	17.4		15.3		87		0				0				97.1						NA
-80.42	43.65	ELORA RC	6142286	43689.1	2019	8	12	0.125	17.3		15.4		89		0		28		5				97.08						NA
-80.42	43.65	ELORA RC	6142286	43689.2	2019	8	12	0.16667	17.6		15.7		89		0		23		7				97.03						NA
-80.42	43.65	ELORA RC	6142286	43689.2	2019	8	12	0.20833	17.4		15.8		90		0		26		8				97.07						NA
-80.42	43.65	ELORA RC	6142286	43689.3	2019	8	12	0.25	17.7		16		90		0				0				97.06						NA
-80.42	43.65	ELORA RC	6142286	43689.3	2019	8	12	0.29167	17.9		16.6		92		0		18		3				97.03						NA

-80.42	43.65	ELORA RC	6142286	43689.3	2019	8	12	0.33333	18.7		16.4		86		0				0				97.04						NA
-80.42	43.65	ELORA RC	6142286	43689.4	2019	8	12	0.375	20.5		17.2		81		0		20		3				97		26				NA
-80.42	43.65	ELORA RC	6142286	43689.4	2019	8	12	0.41667	23.4		17.2		68		0		20		10				97		29				NA
-80.42	43.65	ELORA RC	6142286	43689.5	2019	8	12	0.45833	23.6		16		63		0		19		9				96.94		28				NA
-80.42	43.65	ELORA RC	6142286	43689.5	2019	8	12	0.5	26.3		16.9		56		0		20		11				96.86		32				NA
-80.42	43.65	ELORA RC	6142286	43689.5	2019	8	12	0.54167	26.7		16.3		53		0		24		18				96.82		32				NA
-80.42	43.65	ELORA RC	6142286	43689.6	2019	8	12	0.58333	26.8		16.8		54		0		24		20				96.77		32				NA
-80.42	43.65	ELORA RC	6142286	43689.6	2019	8	12	0.625	26.9		17.2		56		0		24		22				96.72		32				NA
-80.42	43.65	ELORA RC	6142286	43689.7	2019	8	12	0.66667	26.5		16.7		55		0		26		14				96.67		32				NA
-80.42	43.65	ELORA RC	6142286	43689.7	2019	8	12	0.70833	26.4		16.5		55		0		30		13				96.63		31				NA
-80.42	43.65	ELORA RC	6142286	43689.8	2019	8	12	0.75	24.1		18.3		70		0		21		14				96.64		30				NA
-80.42	43.65	ELORA RC	6142286	43689.8	2019	8	12	0.79167	22.2		19		82		0		22		14				96.67		29				NA
-80.42	43.65	ELORA RC	6142286	43689.8	2019	8	12	0.83333	20.7		18.6		88		0		18		7				96.66		27				NA
-80.42	43.65	ELORA RC	6142286	43689.9	2019	8	12	0.875	20.4		18.7		90		0		22		9				96.69		27				NA
-80.42	43.65	ELORA RC	6142286	43689.9	2019	8	12	0.91667	19.4		18.1		92		0		27		7				96.69						NA
-80.42	43.65	ELORA RC	6142286	43690	2019	8	12	0.95833	19.3		18.3		94		0		28		4				96.67						NA
-80.42	43.65	ELORA RC	6142286	43690	2019	8	13	0	18.6		18.1		97		0		31		5				96.64						NA
-80.42	43.65	ELORA RC	6142286	43690	2019	8	13	0.04167	18.2		17.1		93		0		36		5				96.6						NA
-80.42	43.65	ELORA RC	6142286	43690.1	2019	8	13	0.08333	17.1		15.9		93		0				0				96.6						NA
-80.42	43.65	ELORA RC	6142286	43690.1	2019	8	13	0.125	17.5		14.9		84		0		2		7				96.57						NA
-80.42	43.65	ELORA RC	6142286	43690.2	2019	8	13	0.16667	16.5		14.1		86		0		1		11				96.52						NA
-80.42	43.65	ELORA RC	6142286	43690.2	2019	8	13	0.20833	15.5		13.5		88		0		8		4				96.54						NA
-80.42	43.65	ELORA RC	6142286	43690.3	2019	8	13	0.25	14.6		13.2		91		0		5		3				96.58						NA
-80.42	43.65	ELORA RC	6142286	43690.3	2019	8	13	0.29167	17.2		13.2		77		0		36		4				96.61						NA
-80.42	43.65	ELORA RC	6142286	43690.3	2019	8	13	0.33333	20.2		12		59		0		4		6				96.64						NA
-80.42	43.65	ELORA RC	6142286	43690.4	2019	8	13	0.375	22.9		10.5		46		0		8		9				96.63						NA
-80.42	43.65	ELORA RC	6142286	43690.4	2019	8	13	0.41667	24		14.7		56		0		13		9				96.6		28				NA
-80.42	43.65	ELORA RC	6142286	43690.5	2019	8	13	0.45833	24.8		14.8		54		0		2		4				96.61		29				NA
-80.42	43.65	ELORA RC	6142286	43690.5	2019	8	13	0.5	25.1		14.4		51		0		17		10				96.59		29				NA
-80.42	43.65	ELORA RC	6142286	43690.5	2019	8	13	0.54167	26.3		14.6		49		0		14		5				96.56		30				NA
-80.42	43.65	ELORA RC	6142286	43690.6	2019	8	13	0.58333	25.6		14.1		49		0		5		7				96.53		29				NA
-80.42	43.65	ELORA RC	6142286	43690.6	2019	8	13	0.625	27.5		12.2		39		0		4		7				96.48		30				NA
-80.42	43.65	ELORA RC	6142286	43690.7	2019	8	13	0.66667	27.8		9.4		32		0		1		11				96.5		29				NA
-80.42	43.65	ELORA RC	6142286	43690.7	2019	8	13	0.70833	27.1		5.9		26		0		36		7				96.54		27				NA
-80.42	43.65	ELORA RC	6142286	43690.8	2019	8	13	0.75	25.2		12.5		45		0		4		14				96.58		28				NA
-80.42	43.65	ELORA RC	6142286	43690.8	2019	8	13	0.79167	23.1		12.1		50		0		4		19				96.64		25				NA
-80.42	43.65	ELORA RC	6142286	43690.8	2019	8	13	0.83333	20.8		12.3		58		0		4		9				96.7						NA
-80.42	43.65	ELORA RC	6142286	43690.9	2019	8	13	0.875	19.2		10.6		58		0		3		10				96.78						NA
-80.42	43.65	ELORA RC	6142286	43690.9	2019	8	13	0.91667	17.7		10.5		62		0		2		13				96.82						NA
-80.42	43.65	ELORA RC	6142286	43691	2019	8	13	0.95833	17		11.3		69		0		2		14				96.81						NA
-80.42	43.65	ELORA RC	6142286	43691	2019	8	14	0	17.5		11.3		67		0		3		14				96.84						NA
-80.42	43.65	ELORA RC	6142286	43691	2019	8	14	0.04167	17.3		10.7		65		0		3		13				96.85						NA
-80.42	43.65	ELORA RC	6142286	43691.1	2019	8	14	0.08333	16.9		10.7		67		0		2		14				96.84						NA
-80.42	43.65	ELORA RC	6142286	43691.1	2019	8	14	0.125	17.1		10.8		66		0		4		15				96.84						NA
-80.42	43.65	ELORA RC	6142286	43691.2	2019	8	14	0.16667	16.6		10.8		69		0		4		14				96.86						NA
-80.42	43.65	ELORA RC	6142286	43691.2	2019	8	14	0.20833	15.5		10.9		74		0		3		14				96.92						NA
-80.42	43.65	ELORA RC	6142286	43691.3	2019	8	14	0.25	15.3		10.8		75		0		3		16				96.97						NA
-80.42	43.65	ELORA RC	6142286	43691.3	2019	8	14	0.29167	16.6		11.2		70		0		3		14				97.02						NA
-80.42	43.65	ELORA RC	6142286	43691.3	2019	8	14	0.33333	18.5		11.6		64		0		4		14				97.07						NA
-80.42	43.65	ELORA RC	6142286	43691.4	2019	8	14	0.375	20.2		11.1		56		0		4		16				97.1						NA
-80.42	43.65	ELORA RC	6142286	43691.4	2019	8	14	0.41667	21.1		10.9		52		0		4		18				97.1						NA
-80.42	43.65	ELORA RC	6142286	43691.5	2019	8	14	0.45833	21.9		8.9		43		0		4		13				97.11						NA
-80.42	43.65	ELORA RC	6142286	43691.5	2019	8	14	0.5	23.4		9.4		41		0		6		10				97.09						NA
-80.42	43.65	ELORA RC	6142286	43691.5	2019	8	14	0.54167	23.2		7.8		37		0		1		12				97.07						NA
-80.42	43.65	ELORA RC	6142286	43691.6	2019	8	14	0.58333	23.7		6.2		32		0		35		11				97.08						NA
-80.42	43.65	ELORA RC	6142286	43691.6	2019	8	14	0.625	24.1		7.6		35		0		36		8				97.05						NA
-80.42	43.65	ELORA RC	6142286	43691.7	2019	8	14	0.66667	24.1		6.6		32		0		35		11				97.08						NA
-80.42	43.65	ELORA RC	6142286	43691.7	2019	8	14	0.70833	23.8		7.8		36		0		3		13				97.08						NA
-80.42	43.65	ELORA RC	6142286	43691.8	2019	8	14	0.75	22.1		8		40		0		2		14				97.1						NA
-80.42	43.65	ELORA RC	6142286	43691.8	2019	8	14	0.79167	20.3		7.7		44		0		2		14				97.11						NA
-80.42	43.65	ELORA RC	6142286	43691.8	2019	8	14	0.83333	18.3		8		51		0		3		13				97.16						NA
-80.42	43.65	ELORA RC	6142286	43691.9	2019	8	14	0.875	16.6		9		61		0		4		15				97.23						NA
-80.42	43.65	ELORA RC	6142286	43691.9	2019	8	14	0.91667	15.6		9.5		67		0		3		15				97.26						NA
-80.42	43.65	ELORA RC	6142286	43692	2019	8	14	0.95833	14.1		10.2		77		0		4		11				97.3						NA
-80.42	43.65	ELORA RC	6142286	43692	2019	8	15	0	14.4		10.4		77		0		5		11				97.31						NA
-80.42	43.65	ELORA RC	6142286	43692	2019	8	15	0.04167	15		9		68		0		5		16				97.3						NA
-80.42	43.65	ELORA RC	6142286	43692.1	2019	8	15	0.08333	14.1		8.6		69		0		7		13				97.3						NA
-80.42	43.65	ELORA RC	6142286	43692.1	2019	8	15	0.125	13.9		8.2		69		0		7		12				97.29						NA
-80.42	43.65	ELORA RC	6142286	43692.2	2019	8	15	0.16667	14.5		8.1		65		0		7		15				97.26						NA
-80.42	43.65	ELORA RC	6142286	43692.2	2019	8	15	0.20833	14.1		7.9		66		0		8		12				97.26						NA
-80.42	43.65	ELORA RC	6142286	43692.3	2019	8	15	0.25	14.1		8.3		68		0		7		10				97.26						NA
-80.42	43.65	ELORA RC	6142286	43692.3	2019	8	15	0.29167	15.5		8.4		63		0		7		12				97.28						NA
-80.42	43.65	ELORA RC	6142286	43692.3	2019	8	15	0.33333	17.6		10.6		64		0		9		12				97.28						NA
-80.42	43.65	ELORA RC	6142286	43692.4	2019	8	15	0.375	19		11.9		64		0		11		15				97.28						NA
-80.42	43.65	ELORA RC	6142286	43692.4	2019	8	15	0.41667	20.1		12.7		63		0		13		14				97.31						NA
-80.42	43.65	ELORA RC	6142286	43692.5	2019	8	15	0.45833	20.9		12.6		59		0		10		11				97.27						NA
-80.42	43.65	ELORA RC	6142286	43692.5	2019	8	15	0.5	20.4		12.7		61		0		9		9				97.26						NA
-80.42	43.65	ELORA RC	6142286	43692.5	2019	8	15	0.54167	18.9		15.4		80		0.9		10		4				97.25						NA
-80.42	43.65	ELORA RC	6142286	43692.6	2019	8	15	0.58333	19.2		13.2		68		0.1		8		15				97.16						NA
-80.42	43.65	ELORA RC	6142286	43692.6	2019	8	15	0.625	19.2		13.1		68		0		7		11				97.15						NA
-80.42	43.65	ELORA RC	6142286	43692.7	2019	8	15	0.66667	18.4		16.3		87		0.5		7		10				97.12						NA
-80.42	43.65	ELORA RC	6142286	43692.7	2019	8	15	0.70833	19		14.6		76		0		8		13				97.11						NA
-80.42	43.65	ELORA RC	6142286	43692.8	2019	8	15	0.75	18.2		14		76		0		6		10				97.13						NA
-80.42	43.65	ELORA RC	6142286	43692.8	2019	8	15	0.79167	17.4		13.6		78		0		6		7				97.14						NA
-80.42	43.65	ELORA RC	6142286	43692.8	2019	8	15	0.83333	15.7		13		84		0		5		9				97.16						NA
-80.42	43.65	ELORA RC	6142286	43692.9	2019	8	15	0.875	15.2		13.3		89		0		8		6				97.15						NA
-80.42	43.65	ELORA RC	6142286	43692.9	2019	8	15	0.91667	14.7		12.6		87		0		4		4				97.15						NA
-80.42	43.65	ELORA RC	6142286	43693	2019	8	15	0.95833	15.5		11.3		76		0		3		9				97.14						NA
-80.42	43.65	ELORA RC	6142286	43693	2019	8	16	0	13.9		11.1		83		0		7		8				97.12						NA
-80.42	43.65	ELORA RC	6142286	43693	2019	8	16	0.04167	13.2		11.1		87		0		6		9				97.08						NA
-80.42	43.65	ELORA RC	6142286	43693.1	2019	8	16	0.08333	13.6		12.1		91		0		3		7				97.11						NA
-80.42	43.65	ELORA RC	6142286	43693.1	2019	8	16	0.125	14.6		12.9		90		0		5		5				97.15						NA
-80.42	43.65	ELORA RC	6142286	43693.2	2019	8	16	0.16667	14.3		12.7		90		0		4		9				97.1						NA
-80.42	43.65	ELORA RC	6142286	43693.2	2019	8	16	0.20833	13.8		12.1		89		0		2		6				97.12						NA
-80.42	43.65	ELORA RC	6142286	43693.3	2019	8	16	0.25	13.9		11.7		86		0		4		7				97.09						NA
-80.42	43.65	ELORA RC	6142286	43693.3	2019	8	16	0.29167	15.7		12.6		82		0		2		4				97.13						NA
-80.42	43.65	ELORA RC	6142286	43693.3	2019	8	16	0.33333	17.4		13		76		0		7		9				97.16						NA
-80.42	43.65	ELORA RC	6142286	43693.4	2019	8	16	0.375	19.4		13.6		69		0		8		13				97.16						NA
-80.42	43.65	ELORA RC	6142286	43693.4	2019	8	16	0.41667	21.6		14.5		64		0		11		9				97.2		25				NA
-80.42	43.65	ELORA RC	6142286	43693.5	2019	8	16	0.45833	22.5		14		59		0		10		12				97.19		26				NA
-80.42	43.65	ELORA RC	6142286	43693.5	2019	8	16	0.5	23		13.1		53		0		11		12				97.17		26				NA
-80.42	43.65	ELORA RC	6142286	43693.5	2019	8	16	0.54167	23.3		13.9		55		0		12		12				97.09		27				NA
-80.42	43.65	ELORA RC	6142286	43693.6	2019	8	16	0.58333	23.7		14.2		55		0		15		12				97.1		27				NA
-80.42	43.65	ELORA RC	6142286	43693.6	2019	8	16	0.625	23.7		14.9		58		0		14		7				97.08		28				NA
-80.42	43.65	ELORA RC	6142286	43693.7	2019	8	16	0.66667	24.6		14.9		55		0		10		9				96.98		29				NA
-80.42	43.65	ELORA RC	6142286	43693.7	2019	8	16	0.70833	23.4		15.3		60		0		11		16				96.99		28				NA
-80.42	43.65	ELORA RC	6142286	43693.8	2019	8	16	0.75	20.8		16.7		78		0		16		11				96.97		26				NA
-80.42	43.65	ELORA RC	6142286	43693.8	2019	8	16	0.79167	19.4		16.6		84		0		14		11				96.97						NA
-80.42	43.65	ELORA RC	6142286	43693.8	2019	8	16	0.83333	18.9		16.9		88		0		26		6				97.02						NA
-80.42	43.65	ELORA RC	6142286	43693.9	2019	8	16	0.875	18.9		17		88		0		10		7				97.06						NA
-80.42	43.65	ELORA RC	6142286	43693.9	2019	8	16	0.91667	17.1		16		93		0		14		4				97.04						NA
-80.42	43.65	ELORA RC	6142286	43694	2019	8	16	0.95833	16.6		16.1		97		0		12		3				97.07						NA
-80.42	43.65	ELORA RC	6142286	43694	2019	8	17	0	15.2		14.7		97		0		10		5				97.04						NA
-80.42	43.65	ELORA RC	6142286	43694	2019	8	17	0.04167	14.6		14.2		98		0		8		5				97.01						NA
-80.42	43.65	ELORA RC	6142286	43694.1	2019	8	17	0.08333	13.7		13.4		98		0		7		4				97						NA
-80.42	43.65	ELORA RC	6142286	43694.1	2019	8	17	0.125	14.7		14.4		98		0		10		6				96.97						NA
-80.42	43.65	ELORA RC	6142286	43694.2	2019	8	17	0.16667	15.8		15.6		99		0		10		8				96.92						NA
-80.42	43.65	ELORA RC	6142286	43694.2	2019	8	17	0.20833	16.7		16.4		98		0.4		17		6				96.95						NA
-80.42	43.65	ELORA RC	6142286	43694.3	2019	8	17	0.25	17.3		17		98		9.1		13		9				96.9						NA
-80.42	43.65	ELORA RC	6142286	43694.3	2019	8	17	0.29167	17.8		17.4		97		1.4		17		13				96.89						NA
-80.42	43.65	ELORA RC	6142286	43694.3	2019	8	17	0.33333	18.9		18.2		95		0		18		12				96.91						NA
-80.42	43.65	ELORA RC	6142286	43694.4	2019	8	17	0.375	19.9		18.7		93		0.5		20		9				96.96						NA
-80.42	43.65	ELORA RC	6142286	43694.4	2019	8	17	0.41667	22.2		19.9		87		0		22		15				96.89		30				NA
-80.42	43.65	ELORA RC	6142286	43694.5	2019	8	17	0.45833	23.5		18.7		75		0		24		17				96.85		30				NA
-80.42	43.65	ELORA RC	6142286	43694.5	2019	8	17	0.5	25		17.6		63		0		24		23				96.9		31				NA
-80.42	43.65	ELORA RC	6142286	43694.5	2019	8	17	0.54167	25.6		17.2		60		0		26		20				96.91		31				NA
-80.42	43.65	ELORA RC	6142286	43694.6	2019	8	17	0.58333	25.3		16.4		58		0		26		23				96.92		30				NA
-80.42	43.65	ELORA RC	6142286	43694.6	2019	8	17	0.625	25.2		15.4		55		0		27		23				96.93		29				NA
-80.42	43.65	ELORA RC	6142286	43694.7	2019	8	17	0.66667	25		16		57		0		26		23				96.89		30				NA
-80.42	43.65	ELORA RC	6142286	43694.7	2019	8	17	0.70833	24.7		16		58		0		26		21				96.92		29				NA
-80.42	43.65	ELORA RC	6142286	43694.8	2019	8	17	0.75	24.1		14.4		55		0		28		16				96.91		28				NA
-80.42	43.65	ELORA RC	6142286	43694.8	2019	8	17	0.79167	22.6		15.2		63		0		33		4				96.94		27				NA
-80.42	43.65	ELORA RC	6142286	43694.8	2019	8	17	0.83333	19.9		15.3		75		0				0				96.92						NA
-80.42	43.65	ELORA RC	6142286	43694.9	2019	8	17	0.875	17.1		15.1		88		0		5		4				96.99						NA
-80.42	43.65	ELORA RC	6142286	43694.9	2019	8	17	0.91667	16.8		15.6		92		0				0				97.05						NA
-80.42	43.65	ELORA RC	6142286	43695	2019	8	17	0.95833	16.6		15.4		93		0		3		4				97.08						NA
-80.42	43.65	ELORA RC	6142286	43695	2019	8	18	0	16.2		15		93		0		6		5				97.06						NA
-80.42	43.65	ELORA RC	6142286	43695	2019	8	18	0.04167	16.7		15.3		92		0		4		4				97.06						NA

-80.42	43.65	ELORA RC	6142286	43695.1	2019	8	18	0.08333	16.6		15.4		93		0		7		7				97.05						NA
-80.42	43.65	ELORA RC	6142286	43695.1	2019	8	18	0.125	17.6		15.8		89		0		15		10				97.07						NA
-80.42	43.65	ELORA RC	6142286	43695.2	2019	8	18	0.16667	18.2		17.1		94		0.1		17		10				97.03						NA
-80.42	43.65	ELORA RC	6142286	43695.2	2019	8	18	0.20833	17.8		17.4		97		2.3		9		5				96.91						NA
-80.42	43.65	ELORA RC	6142286	43695.3	2019	8	18	0.25	18		17.6		98		0		12		5				97.01						NA
-80.42	43.65	ELORA RC	6142286	43695.3	2019	8	18	0.29167	18.2		17.6		96		0		15		11				97						NA
-80.42	43.65	ELORA RC	6142286	43695.3	2019	8	18	0.33333	18.6		18		96		0		16		10				96.98						NA
-80.42	43.65	ELORA RC	6142286	43695.4	2019	8	18	0.375	20.6		18.2		86		0		15		8				96.94		27				NA
-80.42	43.65	ELORA RC	6142286	43695.4	2019	8	18	0.41667	22.6		19.7		84		0		20		11				97		30				NA
-80.42	43.65	ELORA RC	6142286	43695.5	2019	8	18	0.45833	22.8		20.1		85		0		16		15				96.93		30				NA
-80.42	43.65	ELORA RC	6142286	43695.5	2019	8	18	0.5	24.6		20.2		77		0		18		15				96.89		32				NA
-80.42	43.65	ELORA RC	6142286	43695.5	2019	8	18	0.54167	25.8		19.9		70		0		20		18				96.94		33				NA
-80.42	43.65	ELORA RC	6142286	43695.6	2019	8	18	0.58333	26.9		20.1		67		0		22		16				96.85		35				NA
-80.42	43.65	ELORA RC	6142286	43695.6	2019	8	18	0.625	27.5		20.4		66		0		23		19				96.85		35				NA
-80.42	43.65	ELORA RC	6142286	43695.7	2019	8	18	0.66667	23		20.9		88		1.8		19		31				96.9		31				NA
-80.42	43.65	ELORA RC	6142286	43695.7	2019	8	18	0.70833	19.8		18.6		93		7		9		8				96.73						NA
-80.42	43.65	ELORA RC	6142286	43695.8	2019	8	18	0.75	20.2		19		93		0		19		11				96.77		27				NA
-80.42	43.65	ELORA RC	6142286	43695.8	2019	8	18	0.79167	20.2		19.2		93		0		13		6				96.7		27				NA
-80.42	43.65	ELORA RC	6142286	43695.8	2019	8	18	0.83333	19.9		19.1		95		0		14		8				96.66						NA
-80.42	43.65	ELORA RC	6142286	43695.9	2019	8	18	0.875	19.9		19.2		96		0		16		9				96.68						NA
-80.42	43.65	ELORA RC	6142286	43695.9	2019	8	18	0.91667	19.1		18.5		96		8.1		25		28				96.87						NA
-80.42	43.65	ELORA RC	6142286	43696	2019	8	18	0.95833	18.7		18.2		97		18.4		23		17				96.87						NA
-80.42	43.65	ELORA RC	6142286	43696	2019	8	19	0	18.5		17.8		96		3.2		24		5				96.66						NA
-80.42	43.65	ELORA RC	6142286	43696	2019	8	19	0.04167	18.5		18		97		0.1		22		11				96.75						NA
-80.42	43.65	ELORA RC	6142286	43696.1	2019	8	19	0.08333	19.2		18.7		97		0		22		14				96.84						NA
-80.42	43.65	ELORA RC	6142286	43696.1	2019	8	19	0.125	19.5		18.8		96		0		26		14				96.85						NA
-80.42	43.65	ELORA RC	6142286	43696.2	2019	8	19	0.16667	19.4		18.7		96		0		26		15				96.89						NA
-80.42	43.65	ELORA RC	6142286	43696.2	2019	8	19	0.20833	19.1		18.3		95		0		27		14				96.91						NA
-80.42	43.65	ELORA RC	6142286	43696.3	2019	8	19	0.25	18.6		17.8		95		0		28		12				96.98						NA
-80.42	43.65	ELORA RC	6142286	43696.3	2019	8	19	0.29167	19.3		18.2		93		0		29		14				97.06						NA
-80.42	43.65	ELORA RC	6142286	43696.3	2019	8	19	0.33333	20.4		18		86		0		30		18				97.11		26				NA
-80.42	43.65	ELORA RC	6142286	43696.4	2019	8	19	0.375	20.4		16.1		76		0		32		14				97.21		25				NA
-80.42	43.65	ELORA RC	6142286	43696.4	2019	8	19	0.41667	21.1		16.1		73		0		31		14				97.29		26				NA
-80.42	43.65	ELORA RC	6142286	43696.5	2019	8	19	0.45833	22.3		15.9		67		0		31		11				97.31		27				NA
-80.42	43.65	ELORA RC	6142286	43696.5	2019	8	19	0.5	22.8		14.4		59		0		31		13				97.34		26				NA
-80.42	43.65	ELORA RC	6142286	43696.5	2019	8	19	0.54167	23.4		13.3		53		0		26		13				97.33		26				NA
-80.42	43.65	ELORA RC	6142286	43696.6	2019	8	19	0.58333	23.9		13.4		52		0		30		16				97.33		27				NA
-80.42	43.65	ELORA RC	6142286	43696.6	2019	8	19	0.625	24.1		14.3		54		0		25		16				97.31		28				NA
-80.42	43.65	ELORA RC	6142286	43696.7	2019	8	19	0.66667	24.1		13.1		50		0		27		18				97.32		27				NA
-80.42	43.65	ELORA RC	6142286	43696.7	2019	8	19	0.70833	24		14.5		55		0		27		15				97.32		28				NA
-80.42	43.65	ELORA RC	6142286	43696.8	2019	8	19	0.75	22.5		11.6		50		0		28		18				97.33		25				NA
-80.42	43.65	ELORA RC	6142286	43696.8	2019	8	19	0.79167	20.2		11.7		58		0		30		8				97.35						NA
-80.42	43.65	ELORA RC	6142286	43696.8	2019	8	19	0.83333	17.4		12		71		0		32		6				97.36						NA
-80.42	43.65	ELORA RC	6142286	43696.9	2019	8	19	0.875	16.5		12.2		76		0		28		3				97.41						NA
-80.42	43.65	ELORA RC	6142286	43696.9	2019	8	19	0.91667	15.3		12.2		82		0		2		5				97.43						NA
-80.42	43.65	ELORA RC	6142286	43697	2019	8	19	0.95833	14.4		12.8		90		0				0				97.48						NA
-80.42	43.65	ELORA RC	6142286	43697	2019	8	20	0	14.3		12.6		89		0				0				97.49						NA
-80.42	43.65	ELORA RC	6142286	43697	2019	8	20	0.04167	13.6		12.7		94		0				0				97.47						NA
-80.42	43.65	ELORA RC	6142286	43697.1	2019	8	20	0.08333	13.4		12.2		92		0				0				97.5						NA
-80.42	43.65	ELORA RC	6142286	43697.1	2019	8	20	0.125	14.2		12.8		92		0		7		5				97.52						NA
-80.42	43.65	ELORA RC	6142286	43697.2	2019	8	20	0.16667	13.4		12.1		92		0				0				97.53						NA
-80.42	43.65	ELORA RC	6142286	43697.2	2019	8	20	0.20833	13.6		12.1		91		0		8		3				97.53						NA
-80.42	43.65	ELORA RC	6142286	43697.3	2019	8	20	0.25	13.9		12.4		90		0		8		6				97.52						NA
-80.42	43.65	ELORA RC	6142286	43697.3	2019	8	20	0.29167	16.9		14.6		86		0		9		6				97.51						NA
-80.42	43.65	ELORA RC	6142286	43697.3	2019	8	20	0.33333	18.9		14.9		78		0		14		6				97.49						NA
-80.42	43.65	ELORA RC	6142286	43697.4	2019	8	20	0.375	20.5		16.3		77		0		17		9				97.42		25				NA
-80.42	43.65	ELORA RC	6142286	43697.4	2019	8	20	0.41667																				
-80.42	43.65	ELORA RC	6142286	43697.5	2019	8	20	0.45833																				
-80.42	43.65	ELORA RC	6142286	43697.5	2019	8	20	0.5																				
-80.42	43.65	ELORA RC	6142286	43697.5	2019	8	20	0.54167																				
-80.42	43.65	ELORA RC	6142286	43697.6	2019	8	20	0.58333	26.5		20.4		69		0		22		17				97.31		34				NA
-80.42	43.65	ELORA RC	6142286	43697.6	2019	8	20	0.625	26.5		20.1		68		0		21		14				97.23		34				NA
-80.42	43.65	ELORA RC	6142286	43697.7	2019	8	20	0.66667	26.5		19.4		65		0		21		15				97.2		34				NA
-80.42	43.65	ELORA RC	6142286	43697.7	2019	8	20	0.70833	26.1		20.2		70		0		21		12				97.15		34				NA
-80.42	43.65	ELORA RC	6142286	43697.8	2019	8	20	0.75	25.4		20.3		73		0		19		9				97.07		33				NA
-80.42	43.65	ELORA RC	6142286	43697.8	2019	8	20	0.79167	23.9		19.4		76		0		18		10				97.08		31				NA
-80.42	43.65	ELORA RC	6142286	43697.8	2019	8	20	0.83333	23.2		18.9		77		0		14		6				97.03		30				NA
-80.42	43.65	ELORA RC	6142286	43697.9	2019	8	20	0.875	22.1		18.8		82		0		14		7				97.04		29				NA
-80.42	43.65	ELORA RC	6142286	43697.9	2019	8	20	0.91667	20.3		18.8		91		0		9		5				97.04		27				NA
-80.42	43.65	ELORA RC	6142286	43698	2019	8	20	0.95833	21		19.2		89		0		14		7				96.95		28				NA
-80.42	43.65	ELORA RC	6142286	43698	2019	8	21	0	20.4		18.2		87		0		22		23				97.07		27				NA
-80.42	43.65	ELORA RC	6142286	43698	2019	8	21	0.04167	19.7		18.3		92		0		25		7				97.03						NA
-80.42	43.65	ELORA RC	6142286	43698.1	2019	8	21	0.08333	20		18.7		93		0.1		3		15				96.98		27				NA
-80.42	43.65	ELORA RC	6142286	43698.1	2019	8	21	0.125	19.5		18		91		0.1		6		11				96.88						NA
-80.42	43.65	ELORA RC	6142286	43698.2	2019	8	21	0.16667	18.1		17.3		95		0		26		6				96.82						NA
-80.42	43.65	ELORA RC	6142286	43698.2	2019	8	21	0.20833	18		17.4		97		0		24		5				96.85						NA
-80.42	43.65	ELORA RC	6142286	43698.3	2019	8	21	0.25	18.2		17.8		98		0		26		10				96.87						NA
-80.42	43.65	ELORA RC	6142286	43698.3	2019	8	21	0.29167	19.1		18.6		97		0				0				96.88						NA
-80.42	43.65	ELORA RC	6142286	43698.3	2019	8	21	0.33333	20.7		18.3		86		0		23		9				96.84		27				NA
-80.42	43.65	ELORA RC	6142286	43698.4	2019	8	21	0.375	22.7		19.4		82		0		24		10				96.81		30				NA
-80.42	43.65	ELORA RC	6142286	43698.4	2019	8	21	0.41667	23.2		18.8		76		0		22		13				96.79		30				NA
-80.42	43.65	ELORA RC	6142286	43698.5	2019	8	21	0.45833	24.3		19.9		76		0		24		16				96.84		32				NA
-80.42	43.65	ELORA RC	6142286	43698.5	2019	8	21	0.5	25.6		18.6		65		0		25		19				96.81		32				NA
-80.42	43.65	ELORA RC	6142286	43698.5	2019	8	21	0.54167	26.4		19.8		67		0		26		21				96.8		34				NA
-80.42	43.65	ELORA RC	6142286	43698.6	2019	8	21	0.58333	26.6		19.1		64		0		27		18				96.76		33				NA
-80.42	43.65	ELORA RC	6142286	43698.6	2019	8	21	0.625	26.2		18.5		63		0		27		22				96.73		33				NA
-80.42	43.65	ELORA RC	6142286	43698.7	2019	8	21	0.66667	25.3		17.6		62		0		26		18				96.71		31				NA
-80.42	43.65	ELORA RC	6142286	43698.7	2019	8	21	0.70833	25.5		17.3		61		0		25		18				96.7		31				NA
-80.42	43.65	ELORA RC	6142286	43698.8	2019	8	21	0.75	24		18		69		0		25		15				96.67		30				NA
-80.42	43.65	ELORA RC	6142286	43698.8	2019	8	21	0.79167	21.6		18.6		83		0		22		16				96.66		28				NA
-80.42	43.65	ELORA RC	6142286	43698.8	2019	8	21	0.83333	20.4		18.4		88		0		24		9				96.7		27				NA
-80.42	43.65	ELORA RC	6142286	43698.9	2019	8	21	0.875	20.6		19.1		91		0		25		17				96.72		27				NA
-80.42	43.65	ELORA RC	6142286	43698.9	2019	8	21	0.91667	18.8		15.8		82		0		33		17				96.8						NA
-80.42	43.65	ELORA RC	6142286	43699	2019	8	21	0.95833	17.4		14.2		81		0		34		11				96.83						NA
-80.42	43.65	ELORA RC	6142286	43699	2019	8	22	0	16		13.2		84		0		32		7				96.87						NA
-80.42	43.65	ELORA RC	6142286	43699	2019	8	22	0.04167	15.2		13.2		88		0		28		7				96.87						NA
-80.42	43.65	ELORA RC	6142286	43699.1	2019	8	22	0.08333	15.3		12		81		0		32		16				96.84						NA
-80.42	43.65	ELORA RC	6142286	43699.1	2019	8	22	0.125	14.9		11.6		80		0		31		17				96.87						NA
-80.42	43.65	ELORA RC	6142286	43699.2	2019	8	22	0.16667	14.3		11.2		82		0		29		10				96.92						NA
-80.42	43.65	ELORA RC	6142286	43699.2	2019	8	22	0.20833	13.8		10.9		82		0		29		10				96.95						NA
-80.42	43.65	ELORA RC	6142286	43699.3	2019	8	22	0.25	13.7		10.9		84		0		30		9				96.99						NA
-80.42	43.65	ELORA RC	6142286	43699.3	2019	8	22	0.29167	14.2		11.2		82		0		29		9				97.01						NA
-80.42	43.65	ELORA RC	6142286	43699.3	2019	8	22	0.33333	15.4		11.2		76		0		32		16				97.06						NA
-80.42	43.65	ELORA RC	6142286	43699.4	2019	8	22	0.375	16.7		11		69		0		31		21				97.08						NA
-80.42	43.65	ELORA RC	6142286	43699.4	2019	8	22	0.41667	17.2		9.2		59		0		32		23				97.11						NA
-80.42	43.65	ELORA RC	6142286	43699.5	2019	8	22	0.45833	18.3		10.4		60		0		32		16				97.14						NA
-80.42	43.65	ELORA RC	6142286	43699.5	2019	8	22	0.5	18.6		10.3		58		0		31		19				97.14						NA
-80.42	43.65	ELORA RC	6142286	43699.5	2019	8	22	0.54167	19.6		9.1		51		0		30		20				97.11						NA
-80.42	43.65	ELORA RC	6142286	43699.6	2019	8	22	0.58333	19		9.6		54		0		34		16				97.11						NA
-80.42	43.65	ELORA RC	6142286	43699.6	2019	8	22	0.625	20		8.8		48		0		34		18				97.09						NA
-80.42	43.65	ELORA RC	6142286	43699.7	2019	8	22	0.66667	19.8		9.9		53		0		32		15				97.1						NA
-80.42	43.65	ELORA RC	6142286	43699.7	2019	8	22	0.70833	18.8		10.1		57		0		32		13				97.12						NA
-80.42	43.65	ELORA RC	6142286	43699.8	2019	8	22	0.75	18.3		9.6		57		0		31		18				97.13						NA
-80.42	43.65	ELORA RC	6142286	43699.8	2019	8	22	0.79167	15.7		9.6		67		0		31		13				97.19						NA
-80.42	43.65	ELORA RC	6142286	43699.8	2019	8	22	0.83333	13.9		10.1		78		0		32		12				97.26						NA
-80.42	43.65	ELORA RC	6142286	43699.9	2019	8	22	0.875	13.6		10.2		80		0		33		12				97.29						NA
-80.42	43.65	ELORA RC	6142286	43699.9	2019	8	22	0.91667	12.8		10.4		85		0		34		9				97.29						NA
-80.42	43.65	ELORA RC	6142286	43700	2019	8	22	0.95833	12.5		10.5		88		0		35		7				97.32						NA
-80.42	43.65	ELORA RC	6142286	43700	2019	8	23	0	12.3		10.3		88		0		35		7				97.36						NA
-80.42	43.65	ELORA RC	6142286	43700	2019	8	23	0.04167	11.6		10.2		91		0		34		5				97.4						NA
-80.42	43.65	ELORA RC	6142286	43700.1	2019	8	23	0.08333	11.2		9.5		89		0		35		3				97.4						NA
-80.42	43.65	ELORA RC	6142286	43700.1	2019	8	23	0.125	10.8		9		88		0		36		3				97.43						NA
-80.42	43.65	ELORA RC	6142286	43700.2	2019	8	23	0.16667	11.1		9.6		91		0		36		7				97.46						NA
-80.42	43.65	ELORA RC	6142286	43700.2	2019	8	23	0.20833	10.1		8.6		90		0		36		5				97.49						NA
-80.42	43.65	ELORA RC	6142286	43700.3	2019	8	23	0.25	10.4		9.3		93		0		36		6				97.56						NA
-80.42	43.65	ELORA RC	6142286	43700.3	2019	8	23	0.29167	12.6		10.6		87		0		36		8				97.59						NA
-80.42	43.65	ELORA RC	6142286	43700.3	2019	8	23	0.33333	15.4		11.4		77		0		36		10				97.61						NA
-80.42	43.65	ELORA RC	6142286	43700.4	2019	8	23	0.375	17.1		11.1		68		0		2		16				97.64						NA
-80.42	43.65	ELORA RC	6142286	43700.4	2019	8	23	0.41667	18.1		10.2		60		0		35		16				97.67						NA
-80.42	43.65	ELORA RC	6142286	43700.5	2019	8	23	0.45833	19.3		8.7		50		0		1		14				97.67						NA
-80.42	43.65	ELORA RC	6142286	43700.5	2019	8	23	0.5	18.4		9.3		56		0		1		19				97.69						NA
-80.42	43.65	ELORA RC	6142286	43700.5	2019	8	23	0.54167	18.6		8.3		51		0		36		16				97.71						NA
-80.42	43.65	ELORA RC	6142286	43700.6	2019	8	23	0.58333	18.8		8.8		52		0		1		12				97.7						NA
-80.42	43.65	ELORA RC	6142286	43700.6	2019	8	23	0.625	19.1		7.4		46		0		36		17				97.7						NA
-80.42	43.65	ELORA RC	6142286	43700.7	2019	8	23	0.66667	19.4		5.9		41		0		1		18				97.69						NA
-80.42	43.65	ELORA RC	6142286	43700.7	2019	8	23	0.70833	18.9		4.9		40		0		36		18				97.71						NA
-80.42	43.65	ELORA RC	6142286	43700.8	2019	8	23	0.75	17.9		5.4		44		0		1		15				97.74						NA
-80.42	43.65	ELORA RC	6142286	43700.8	2019	8	23	0.79167	16		6.1		52		0		36		9				97.77						NA

-80.42	43.65	ELORA RC	6142286	43700.8	2019	8	23	0.83333	13.7		6.6		62		0		1		9				97.83						NA
-80.42	43.65	ELORA RC	6142286	43700.9	2019	8	23	0.875	12.4		7.3		71		0		36		6				97.87						NA
-80.42	43.65	ELORA RC	6142286	43700.9	2019	8	23	0.91667	11.5		8.1		80		0		36		6				97.91						NA
-80.42	43.65	ELORA RC	6142286	43701	2019	8	23	0.95833	11.1		8		81		0		35		7				97.94						NA
-80.42	43.65	ELORA RC	6142286	43701	2019	8	24	0	10.7		8		84		0		36		6				97.98						NA
-80.42	43.65	ELORA RC	6142286	43701	2019	8	24	0.04167	9.9		7.7		86		0		3		8				98						NA
-80.42	43.65	ELORA RC	6142286	43701.1	2019	8	24	0.08333	11.2		8		81		0		3		11				98						NA
-80.42	43.65	ELORA RC	6142286	43701.1	2019	8	24	0.125	11.2		7.5		78		0		3		12				98.03						NA
-80.42	43.65	ELORA RC	6142286	43701.2	2019	8	24	0.16667	10.8		7.2		79		0		2		13				98.04						NA
-80.42	43.65	ELORA RC	6142286	43701.2	2019	8	24	0.20833	11		7.5		79		0		3		12				98.09						NA
-80.42	43.65	ELORA RC	6142286	43701.3	2019	8	24	0.25	10.5		7.4		81		0		2		9				98.14						NA
-80.42	43.65	ELORA RC	6142286	43701.3	2019	8	24	0.29167	12.5		8.5		77		0		3		9				98.2						NA
-80.42	43.65	ELORA RC	6142286	43701.3	2019	8	24	0.33333	14.8		9.4		70		0		4		7				98.24						NA
-80.42	43.65	ELORA RC	6142286	43701.4	2019	8	24	0.375	16.7		9.5		62		0		8		8				98.24						NA
-80.42	43.65	ELORA RC	6142286	43701.4	2019	8	24	0.41667	17.8		9.5		58		0		9		13				98.24						NA
-80.42	43.65	ELORA RC	6142286	43701.5	2019	8	24	0.45833	18.3		9.3		56		0		3		11				98.2						NA
-80.42	43.65	ELORA RC	6142286	43701.5	2019	8	24	0.5	19.3		8.7		50		0		7		12				98.14						NA
-80.42	43.65	ELORA RC	6142286	43701.5	2019	8	24	0.54167	19.8		9.5		51		0		5		12				98.12						NA
-80.42	43.65	ELORA RC	6142286	43701.6	2019	8	24	0.58333	20.4		10.5		53		0		5		12				98.1						NA
-80.42	43.65	ELORA RC	6142286	43701.6	2019	8	24	0.625	20.8		10		50		0		6		10				98.08						NA
-80.42	43.65	ELORA RC	6142286	43701.7	2019	8	24	0.66667	20.4		10.4		53		0		7		10				98.05						NA
-80.42	43.65	ELORA RC	6142286	43701.7	2019	8	24	0.70833	20.2		9.9		52		0		7		7				98.08						NA
-80.42	43.65	ELORA RC	6142286	43701.8	2019	8	24	0.75	18.1		11.3		64		0		14		12				98.09						NA
-80.42	43.65	ELORA RC	6142286	43701.8	2019	8	24	0.79167	17		11.2		69		0		12		5				98.12						NA
-80.42	43.65	ELORA RC	6142286	43701.8	2019	8	24	0.83333	15.1		11.5		79		0				0				98.15						NA
-80.42	43.65	ELORA RC	6142286	43701.9	2019	8	24	0.875	13.1		10.5		84		0		7		5				98.18						NA
-80.42	43.65	ELORA RC	6142286	43701.9	2019	8	24	0.91667	11.7		9.7		88		0		2		5				98.17						NA
-80.42	43.65	ELORA RC	6142286	43702	2019	8	24	0.95833	11.9		10		88		0		3		6				98.17						NA
-80.42	43.65	ELORA RC	6142286	43702	2019	8	25	0	11.6		10.1		91		0		5		6				98.17						NA
-80.42	43.65	ELORA RC	6142286	43702	2019	8	25	0.04167	10.8		9.3		91		0		7		9				98.12						NA
-80.42	43.65	ELORA RC	6142286	43702.1	2019	8	25	0.08333	10.8		9.4		91		0		5		9				98.1						NA
-80.42	43.65	ELORA RC	6142286	43702.1	2019	8	25	0.125	11.2		10		92		0		5		8				98.11						NA
-80.42	43.65	ELORA RC	6142286	43702.2	2019	8	25	0.16667	12.2		10.6		90		0		2		10				98.13						NA
-80.42	43.65	ELORA RC	6142286	43702.2	2019	8	25	0.20833	10.9		9.9		93		0		7		8				98.16						NA
-80.42	43.65	ELORA RC	6142286	43702.3	2019	8	25	0.25	11.1		9.9		93		0		7		7				98.16						NA
-80.42	43.65	ELORA RC	6142286	43702.3	2019	8	25	0.29167	14.2		11.9		86		0		6		7				98.2						NA
-80.42	43.65	ELORA RC	6142286	43702.3	2019	8	25	0.33333	16.3		13		81		0		9		14				98.18						NA
-80.42	43.65	ELORA RC	6142286	43702.4	2019	8	25	0.375	17.6		12		70		0		11		19				98.17						NA
-80.42	43.65	ELORA RC	6142286	43702.4	2019	8	25	0.41667	18.5		11.7		65		0		11		17				98.2						NA
-80.42	43.65	ELORA RC	6142286	43702.5	2019	8	25	0.45833	20.1		11.3		57		0		10		19				98.15						NA
-80.42	43.65	ELORA RC	6142286	43702.5	2019	8	25	0.5	20.7		11		54		0		10		17				98.09						NA
-80.42	43.65	ELORA RC	6142286	43702.5	2019	8	25	0.54167	21.2		11.2		53		0		11		18				98.02						NA
-80.42	43.65	ELORA RC	6142286	43702.6	2019	8	25	0.58333	21.3		10		48		0		11		19				98						NA
-80.42	43.65	ELORA RC	6142286	43702.6	2019	8	25	0.625	20.9		11.2		54		0		12		16				97.95						NA
-80.42	43.65	ELORA RC	6142286	43702.7	2019	8	25	0.66667	20.1		12.1		60		0		11		17				97.95						NA
-80.42	43.65	ELORA RC	6142286	43702.7	2019	8	25	0.70833	20.9		12		57		0		11		15				97.91						NA
-80.42	43.65	ELORA RC	6142286	43702.8	2019	8	25	0.75	19.8		11.8		60		0		10		15				97.9						NA
-80.42	43.65	ELORA RC	6142286	43702.8	2019	8	25	0.79167	17.8		11.9		68		0		10		12				97.87						NA
-80.42	43.65	ELORA RC	6142286	43702.8	2019	8	25	0.83333	15.7		11.3		75		0		9		11				97.87						NA
-80.42	43.65	ELORA RC	6142286	43702.9	2019	8	25	0.875	15		11.1		77		0		9		12				97.84						NA
-80.42	43.65	ELORA RC	6142286	43702.9	2019	8	25	0.91667	14.9		11		78		0		9		14				97.85						NA
-80.42	43.65	ELORA RC	6142286	43703	2019	8	25	0.95833	14.4		11.6		83		0		9		12				97.84						NA
-80.42	43.65	ELORA RC	6142286	43703	2019	8	26	0	14.1		12.5		90		0		9		11				97.85						NA
-80.42	43.65	ELORA RC	6142286	43703	2019	8	26	0.04167	14.3		13.3		94		0		9		12				97.85						NA
-80.42	43.65	ELORA RC	6142286	43703.1	2019	8	26	0.08333	13.9		13.1		95		0		9		10				97.81						NA
-80.42	43.65	ELORA RC	6142286	43703.1	2019	8	26	0.125	13.5		12.7		95		0		9		10				97.78						NA
-80.42	43.65	ELORA RC	6142286	43703.2	2019	8	26	0.16667	13.4		12.7		96		0		9		10				97.75						NA
-80.42	43.65	ELORA RC	6142286	43703.2	2019	8	26	0.20833	13.3		12.5		95		0		9		10				97.75						NA
-80.42	43.65	ELORA RC	6142286	43703.3	2019	8	26	0.25	13.4		12.6		95		0		8		10				97.77						NA
-80.42	43.65	ELORA RC	6142286	43703.3	2019	8	26	0.29167	15.3		13.7		90		0		9		11				97.79						NA
-80.42	43.65	ELORA RC	6142286	43703.3	2019	8	26	0.33333	17.5		14.6		83		0		10		12				97.78						NA
-80.42	43.65	ELORA RC	6142286	43703.4	2019	8	26	0.375	19.3		14.5		74		0		13		16				97.71						NA
-80.42	43.65	ELORA RC	6142286	43703.4	2019	8	26	0.41667	20		13.4		66		0		15		20				97.66						NA
-80.42	43.65	ELORA RC	6142286	43703.5	2019	8	26	0.45833	20.5		12.8		61		0		15		23				97.61						NA
-80.42	43.65	ELORA RC	6142286	43703.5	2019	8	26	0.5	21.1		11.3		53		0		15		28				97.54						NA
-80.42	43.65	ELORA RC	6142286	43703.5	2019	8	26	0.54167	22		10.3		48		0		15		30				97.49						NA
-80.42	43.65	ELORA RC	6142286	43703.6	2019	8	26	0.58333	21.6		10		48		0		16		27				97.41						NA
-80.42	43.65	ELORA RC	6142286	43703.6	2019	8	26	0.625	22.1		11.2		50		0		16		27				97.35						NA
-80.42	43.65	ELORA RC	6142286	43703.7	2019	8	26	0.66667	22		11.9		53		0		14		25				97.3						NA
-80.42	43.65	ELORA RC	6142286	43703.7	2019	8	26	0.70833	20.4		12.6		61		0		14		17				97.29						NA
-80.42	43.65	ELORA RC	6142286	43703.8	2019	8	26	0.75	19.9		13.2		65		0		12		15				97.25						NA
-80.42	43.65	ELORA RC	6142286	43703.8	2019	8	26	0.79167	18.1		14		77		0		11		16				97.24						NA
-80.42	43.65	ELORA RC	6142286	43703.8	2019	8	26	0.83333	16.9		13.9		82		0		10		12				97.21						NA
-80.42	43.65	ELORA RC	6142286	43703.9	2019	8	26	0.875	16.8		13.8		83		0		10		15				97.15						NA
-80.42	43.65	ELORA RC	6142286	43703.9	2019	8	26	0.91667	16.9		14.1		84		0		11		10				97.1						NA
-80.42	43.65	ELORA RC	6142286	43704	2019	8	26	0.95833	16.9		14		83		0		12		7				97.05						NA
-80.42	43.65	ELORA RC	6142286	43704	2019	8	27	0	17.6		13.7		78		0		14		11				97						NA
-80.42	43.65	ELORA RC	6142286	43704	2019	8	27	0.04167	17.9		13.5		76		0		15		13				96.93						NA
-80.42	43.65	ELORA RC	6142286	43704.1	2019	8	27	0.08333	17.9		13.7		77		0		16		15				96.87						NA
-80.42	43.65	ELORA RC	6142286	43704.1	2019	8	27	0.125	17.4		14.8		85		0		16		16				96.8						NA
-80.42	43.65	ELORA RC	6142286	43704.2	2019	8	27	0.16667	16.5		15.7		95		1.9		16		14				96.76						NA
-80.42	43.65	ELORA RC	6142286	43704.2	2019	8	27	0.20833	16.6		15.9		96		0		16		18				96.73						NA
-80.42	43.65	ELORA RC	6142286	43704.3	2019	8	27	0.25	16.8		16.2		96		0.1		17		19				96.71						NA
-80.42	43.65	ELORA RC	6142286	43704.3	2019	8	27	0.29167	17.1		16.5		96		0		17		19				96.71						NA
-80.42	43.65	ELORA RC	6142286	43704.3	2019	8	27	0.33333	17.5		16.9		96		0.1		17		20				96.68						NA
-80.42	43.65	ELORA RC	6142286	43704.4	2019	8	27	0.375	17.8		17.3		97		0.5		17		18				96.66						NA
-80.42	43.65	ELORA RC	6142286	43704.4	2019	8	27	0.41667	18.1		17.5		96		0.4		17		19				96.65						NA
-80.42	43.65	ELORA RC	6142286	43704.5	2019	8	27	0.45833	18.4		17.8		96		1		17		18				96.66						NA
-80.42	43.65	ELORA RC	6142286	43704.5	2019	8	27	0.5	18.9		18.1		95		2.1		17		19				96.62						NA
-80.42	43.65	ELORA RC	6142286	43704.5	2019	8	27	0.54167	19.9		18.8		93		0		18		19				96.58						NA
-80.42	43.65	ELORA RC	6142286	43704.6	2019	8	27	0.58333	19.6		19		96		0		18		16				96.57						NA
-80.42	43.65	ELORA RC	6142286	43704.6	2019	8	27	0.625	21.2		19.9		93		0.2		18		15				96.53		29				NA
-80.42	43.65	ELORA RC	6142286	43704.7	2019	8	27	0.66667	21		19.8		93		0		18		13				96.52		28				NA
-80.42	43.65	ELORA RC	6142286	43704.7	2019	8	27	0.70833	21.4		20		92		0		19		22				96.51		29				NA
-80.42	43.65	ELORA RC	6142286	43704.8	2019	8	27	0.75	21		19.7		92		0		18		18				96.52		28				NA
-80.42	43.65	ELORA RC	6142286	43704.8	2019	8	27	0.79167	21		19.6		91		0		19		18				96.57		28				NA
-80.42	43.65	ELORA RC	6142286	43704.8	2019	8	27	0.83333	20.8		19.4		92		0		20		18				96.62		28				NA
-80.42	43.65	ELORA RC	6142286	43704.9	2019	8	27	0.875	20.5		19.3		92		0		21		15				96.64		28				NA
-80.42	43.65	ELORA RC	6142286	43704.9	2019	8	27	0.91667	20.3		19.2		93		0		23		10				96.66		27				NA
-80.42	43.65	ELORA RC	6142286	43705	2019	8	27	0.95833	20.1		19		94		0		27		10				96.63		27				NA
-80.42	43.65	ELORA RC	6142286	43705	2019	8	28	0	17.3		16		92		0		24		5				96.63						NA
-80.42	43.65	ELORA RC	6142286	43705	2019	8	28	0.04167	16.6		16		96		0		24		4				96.62						NA
-80.42	43.65	ELORA RC	6142286	43705.1	2019	8	28	0.08333	16.5		16.1		98		0		22		6				96.59						NA
-80.42	43.65	ELORA RC	6142286	43705.1	2019	8	28	0.125	16.2		15.9		98		0		29		5				96.56						NA
-80.42	43.65	ELORA RC	6142286	43705.2	2019	8	28	0.16667	15.5		15.2		98		0		29		4				96.54						NA
-80.42	43.65	ELORA RC	6142286	43705.2	2019	8	28	0.20833	14.7		14.4		98		0		28		4				96.56						NA
-80.42	43.65	ELORA RC	6142286	43705.3	2019	8	28	0.25	13.5		13.3		98		0				0				96.61						NA
-80.42	43.65	ELORA RC	6142286	43705.3	2019	8	28	0.29167	15.7		15.3		98		0				0				96.64						NA
-80.42	43.65	ELORA RC	6142286	43705.3	2019	8	28	0.33333	17.7		15.4		87		0		25		9				96.66						NA
-80.42	43.65	ELORA RC	6142286	43705.4	2019	8	28	0.375	19.9		15		73		0		25		15				96.66						NA
-80.42	43.65	ELORA RC	6142286	43705.4	2019	8	28	0.41667	21.1		11.7		55		0		25		16				96.65						NA
-80.42	43.65	ELORA RC	6142286	43705.5	2019	8	28	0.45833	21.9		11.1		50		0		25		22				96.63						NA
-80.42	43.65	ELORA RC	6142286	43705.5	2019	8	28	0.5	21.9		9.2		44		0		24		26				96.61						NA
-80.42	43.65	ELORA RC	6142286	43705.5	2019	8	28	0.54167	22		10.3		47		0		26		22				96.6						NA
-80.42	43.65	ELORA RC	6142286	43705.6	2019	8	28	0.58333	21.9		10.5		48		0		25		24				96.59						NA
-80.42	43.65	ELORA RC	6142286	43705.6	2019	8	28	0.625	21.9		11.5		52		0		25		21				96.59						NA
-80.42	43.65	ELORA RC	6142286	43705.7	2019	8	28	0.66667	20.9		12		57		0		25		21				96.61						NA
-80.42	43.65	ELORA RC	6142286	43705.7	2019	8	28	0.70833	21		10.8		52		0		26		20				96.62						NA
-80.42	43.65	ELORA RC	6142286	43705.8	2019	8	28	0.75	19.7		10.7		56		0		27		20				96.65						NA
-80.42	43.65	ELORA RC	6142286	43705.8	2019	8	28	0.79167	17.4		12.2		72		0		31		11				96.72						NA
-80.42	43.65	ELORA RC	6142286	43705.8	2019	8	28	0.83333	16.8		12.4		75		0		28		9				96.75						NA
-80.42	43.65	ELORA RC	6142286	43705.9	2019	8	28	0.875	15.6		11.9		79		0		25		11				96.78						NA
-80.42	43.65	ELORA RC	6142286	43705.9	2019	8	28	0.91667	15.4		11.7		78		0		28		13				96.81						NA
-80.42	43.65	ELORA RC	6142286	43706	2019	8	28	0.95833	14.4		11.3		81		0		27		12				96.81						NA
-80.42	43.65	ELORA RC	6142286	43706	2019	8	29	0	13.6		11.6		87		0		25		9				96.84						NA
-80.42	43.65	ELORA RC	6142286	43706	2019	8	29	0.04167	13.7		11.9		89		0		25		6				96.85						NA
-80.42	43.65	ELORA RC	6142286	43706.1	2019	8	29	0.08333	12.8		11.4		91		0		21		6				96.84						NA
-80.42	43.65	ELORA RC	6142286	43706.1	2019	8	29	0.125	12.5		11		90		0		25		7				96.86						NA
-80.42	43.65	ELORA RC	6142286	43706.2	2019	8	29	0.16667	11.3		10.3		93		0		21		9				96.88						NA
-80.42	43.65	ELORA RC	6142286	43706.2	2019	8	29	0.20833	11.7		10.9		95		0		22		5				96.93						NA
-80.42	43.65	ELORA RC	6142286	43706.3	2019	8	29	0.25	12		11.2		95		0				0				96.95						NA
-80.42	43.65	ELORA RC	6142286	43706.3	2019	8	29	0.29167	13.5		12		91		0		26		5				96.98						NA
-80.42	43.65	ELORA RC	6142286	43706.3	2019	8	29	0.33333	15.9		12.2		79		0		26		16				97.02						NA
-80.42	43.65	ELORA RC	6142286	43706.4	2019	8	29	0.375	17.5		10.2		62		0		27		18				97.04						NA
-80.42	43.65	ELORA RC	6142286	43706.4	2019	8	29	0.41667	18.8		10.2		57		0		26		23				97.04						NA
-80.42	43.65	ELORA RC	6142286	43706.5	2019	8	29	0.45833	20.2		11.9		59		0		26		18				97.04						NA
-80.42	43.65	ELORA RC	6142286	43706.5	2019	8	29	0.5	20		9.9		52		0		25		20				96.97						NA
-80.42	43.65	ELORA RC	6142286	43706.5	2019	8	29	0.54167	20.5		9.6		49		0		24		22				96.93						NA

-80.42	43.65	ELORA RC	6142286	43706.6	2019	8	29	0.58333	21.5		10.9		51		0		23		20				96.91						NA
-80.42	43.65	ELORA RC	6142286	43706.6	2019	8	29	0.625	21.7		10.5		49		0		23		25				96.85						NA
-80.42	43.65	ELORA RC	6142286	43706.7	2019	8	29	0.66667	21.7		11.1		51		0		21		24				96.81						NA
-80.42	43.65	ELORA RC	6142286	43706.7	2019	8	29	0.70833	21.8		11.5		52		0		22		26				96.77						NA
-80.42	43.65	ELORA RC	6142286	43706.8	2019	8	29	0.75	20.7		11.2		55		0		23		15				96.75						NA
-80.42	43.65	ELORA RC	6142286	43706.8	2019	8	29	0.79167	18.1		14.1		78		0		19		15				96.73						NA
-80.42	43.65	ELORA RC	6142286	43706.8	2019	8	29	0.83333	17.6		14.2		80		0		18		13				96.69						NA
-80.42	43.65	ELORA RC	6142286	43706.9	2019	8	29	0.875	18.5		14.2		76		0		20		17				96.7						NA
-80.42	43.65	ELORA RC	6142286	43706.9	2019	8	29	0.91667	19.2		14.6		75		0		21		25				96.6						NA
-80.42	43.65	ELORA RC	6142286	43707	2019	8	29	0.95833	19.8		14.7		72		0		21		26				96.53						NA
-80.42	43.65	ELORA RC	6142286	43707	2019	8	30	0	20.7		14.9		69		0		21		28				96.5		25				NA
-80.42	43.65	ELORA RC	6142286	43707	2019	8	30	0.04167	20.6		15.9		74		0		23		24				96.46		25				NA
-80.42	43.65	ELORA RC	6142286	43707.1	2019	8	30	0.08333	18.2		15.1		82		0		31		23				96.58						NA
-80.42	43.65	ELORA RC	6142286	43707.1	2019	8	30	0.125	15.3		12.7		85		0		31		12				96.72						NA
-80.42	43.65	ELORA RC	6142286	43707.2	2019	8	30	0.16667	13.4		12		91		0		30		8				96.79						NA
-80.42	43.65	ELORA RC	6142286	43707.2	2019	8	30	0.20833	12.3		11.6		96		0		27		7				96.83						NA
-80.42	43.65	ELORA RC	6142286	43707.3	2019	8	30	0.25	11.6		11		96		0		28		7				96.9						NA
-80.42	43.65	ELORA RC	6142286	43707.3	2019	8	30	0.29167	13.4		12.1		92		0		26		10				97.05						NA
-80.42	43.65	ELORA RC	6142286	43707.3	2019	8	30	0.33333	15.9		12.7		81		0		28		14				97.1						NA
-80.42	43.65	ELORA RC	6142286	43707.4	2019	8	30	0.375	17.3		8.9		58		0		29		21				97.18						NA
-80.42	43.65	ELORA RC	6142286	43707.4	2019	8	30	0.41667	18.6		9.2		54		0		29		24				97.22						NA
-80.42	43.65	ELORA RC	6142286	43707.5	2019	8	30	0.45833	19.3		9.3		52		0		27		29				97.27						NA
-80.42	43.65	ELORA RC	6142286	43707.5	2019	8	30	0.5	20		7.6		45		0		27		27				97.27						NA
-80.42	43.65	ELORA RC	6142286	43707.5	2019	8	30	0.54167	19.4		8.5		49		0		30		28				97.31						NA
-80.42	43.65	ELORA RC	6142286	43707.6	2019	8	30	0.58333	19.8		8.6		48		0		29		23				97.33						NA
-80.42	43.65	ELORA RC	6142286	43707.6	2019	8	30	0.625	19.3		7.2		45		0		29		19				97.39						NA
-80.42	43.65	ELORA RC	6142286	43707.7	2019	8	30	0.66667	19.2		7.3		46		0		32		19				97.43						NA
-80.42	43.65	ELORA RC	6142286	43707.7	2019	8	30	0.70833	18.4		8		51		0		31		16				97.49						NA
-80.42	43.65	ELORA RC	6142286	43707.8	2019	8	30	0.75	17		8.2		56		0		31		11				97.52						NA
-80.42	43.65	ELORA RC	6142286	43707.8	2019	8	30	0.79167	16.3		8		58		0		29		5				97.59						NA
-80.42	43.65	ELORA RC	6142286	43707.8	2019	8	30	0.83333	13		8.8		76		0				0				97.66						NA
-80.42	43.65	ELORA RC	6142286	43707.9	2019	8	30	0.875	11.5		8.8		83		0				0				97.69						NA
-80.42	43.65	ELORA RC	6142286	43707.9	2019	8	30	0.91667	11		8.9		87		0				0				97.72						NA
-80.42	43.65	ELORA RC	6142286	43708	2019	8	30	0.95833	10.9		9.1		88		0		32		3				97.75						NA
-80.42	43.65	ELORA RC	6142286	43708	2019	8	31	0	10.2		8.2		87		0		27		5				97.83						NA
-80.42	43.65	ELORA RC	6142286	43708	2019	8	31	0.04167	12.2		9.2		82		0		36		10				97.84						NA
-80.42	43.65	ELORA RC	6142286	43708.1	2019	8	31	0.08333	11.2		8.6		84		0		35		7				97.87						NA
-80.42	43.65	ELORA RC	6142286	43708.1	2019	8	31	0.125	9.1		8		93		0		28		4				97.94						NA
-80.42	43.65	ELORA RC	6142286	43708.2	2019	8	31	0.16667	8.5		7.8		96		0				0				98						NA
-80.42	43.65	ELORA RC	6142286	43708.2	2019	8	31	0.20833	7.8		7.5		98		0		28		5				98.06						NA
-80.42	43.65	ELORA RC	6142286	43708.3	2019	8	31	0.25	6.9		6.5		97		0		36		3				98.11						NA
-80.42	43.65	ELORA RC	6142286	43708.3	2019	8	31	0.29167	9.1		8		93		0				0				98.2						NA
-80.42	43.65	ELORA RC	6142286	43708.3	2019	8	31	0.33333	12.4		8.8		79		0				0				98.21						NA
-80.42	43.65	ELORA RC	6142286	43708.4	2019	8	31	0.375	15.5		8.5		63		0		33		10				98.25						NA
-80.42	43.65	ELORA RC	6142286	43708.4	2019	8	31	0.41667	16.4		8.5		60		0		36		10				98.28						NA
-80.42	43.65	ELORA RC	6142286	43708.5	2019	8	31	0.45833	17.3		8		54		0		1		12				98.28						NA
-80.42	43.65	ELORA RC	6142286	43708.5	2019	8	31	0.5	18.5		7.3		48		0		34		15				98.24						NA
-80.42	43.65	ELORA RC	6142286	43708.5	2019	8	31	0.54167	19.2		7.9		48		0		35		13				98.23						NA
-80.42	43.65	ELORA RC	6142286	43708.6	2019	8	31	0.58333	18.8		7.6		48		0		3		12				98.23						NA
-80.42	43.65	ELORA RC	6142286	43708.6	2019	8	31	0.625	19.6		6.9		44		0		5		8				98.18						NA
-80.42	43.65	ELORA RC	6142286	43708.7	2019	8	31	0.66667	19.7		6.5		42		0		2		7				98.2						NA
-80.42	43.65	ELORA RC	6142286	43708.7	2019	8	31	0.70833	19.3		5.4		40		0		36		4				98.17						NA
-80.42	43.65	ELORA RC	6142286	43708.8	2019	8	31	0.75	18.2		8.8		54		0		5		4				98.18						NA
-80.42	43.65	ELORA RC	6142286	43708.8	2019	8	31	0.79167	16.1		7.8		58		0		4		9				98.18						NA
-80.42	43.65	ELORA RC	6142286	43708.8	2019	8	31	0.83333	13.7		8.3		70		0		6		9				98.2						NA
-80.42	43.65	ELORA RC	6142286	43708.9	2019	8	31	0.875	12.4		7.9		74		0		5		10				98.19						NA
-80.42	43.65	ELORA RC	6142286	43708.9	2019	8	31	0.91667	12.5		8		74		0		8		10				98.18						NA
-80.42	43.65	ELORA RC	6142286	43709	2019	8	31	0.95833	12.9		7.5		70		0		6		12				98.17						NA

Table S2C. Hourly recorded weather data of the field trial in September 2019

Longitude	Latitude (	Station Na	Climate ID	Date/Tim	Year	Month	Day	Time (LST	Temp (°C)	Temp Flag	Dew Poin	Dew Poin	Rel Hum (	Rel Hum F	Precip. Am	Precip. Am	Wind Dir (	Wind Dir (	Wind Spd	Wind Spd	Visibility (	Visibility F	Stn Press	Stn Press	Hmdx	Hmdx Flag	Wind Chil	Wind Chil	Weather
-80.42	43.65	ELORA RC	6142286	43709	2019	9	1	0	12.2		7.5		73		0		7		8				98.19						NA
-80.42	43.65	ELORA RC	6142286	43709	2019	9	1	0.04167	12.1		7.6		74		0		5		9				98.17						NA
-80.42	43.65	ELORA RC	6142286	43709.1	2019	9	1	0.08333	11.3		7.5		77		0		6		7				98.15						NA
-80.42	43.65	ELORA RC	6142286	43709.1	2019	9	1	0.125	11.5		8.2		80		0		6		7				98.11						NA
-80.42	43.65	ELORA RC	6142286	43709.2	2019	9	1	0.16667	12.1		8.6		79		0		7		8				98.08						NA
-80.42	43.65	ELORA RC	6142286	43709.2	2019	9	1	0.20833	12.4		9.2		81		0		6		8				98.05						NA
-80.42	43.65	ELORA RC	6142286	43709.3	2019	9	1	0.25	13.5		10.1		80		0		7		10				98.07						NA
-80.42	43.65	ELORA RC	6142286	43709.3	2019	9	1	0.29167	13.4		10.1		80		0		5		8				98.05						NA
-80.42	43.65	ELORA RC	6142286	43709.3	2019	9	1	0.33333	14.4		10.6		78		0		5		8				98.03						NA
-80.42	43.65	ELORA RC	6142286	43709.4	2019	9	1	0.375	14.6		11.3		81		0.1		9		10				97.98						NA
-80.42	43.65	ELORA RC	6142286	43709.4	2019	9	1	0.41667	16.1		12.2		77		0		10		12				97.96						NA
-80.42	43.65	ELORA RC	6142286	43709.5	2019	9	1	0.45833	17.2		13.1		77		0		11		8				97.94						NA
-80.42	43.65	ELORA RC	6142286	43709.5	2019	9	1	0.5	16.3		13.4		83		0.4		8		6				97.92						NA
-80.42	43.65	ELORA RC	6142286	43709.5	2019	9	1	0.54167	16.5		13.6		83		0.2		12		4				97.87						NA
-80.42	43.65	ELORA RC	6142286	43709.6	2019	9	1	0.58333	16.6		14.5		87		0.1		10		7				97.76						NA
-80.42	43.65	ELORA RC	6142286	43709.6	2019	9	1	0.625	16.5		15		91		0.3		10		7				97.68						NA
-80.42	43.65	ELORA RC	6142286	43709.7	2019	9	1	0.66667	16.8		15.3		91		0.3		8		8				97.61						NA
-80.42	43.65	ELORA RC	6142286	43709.7	2019	9	1	0.70833	16.6		14.6		88		0		10		7				97.6						NA
-80.42	43.65	ELORA RC	6142286	43709.8	2019	9	1	0.75	16.3		14.7		90		0		11		7				97.56						NA
-80.42	43.65	ELORA RC	6142286	43709.8	2019	9	1	0.79167	15.8		14.4		92		0		11		7				97.51						NA
-80.42	43.65	ELORA RC	6142286	43709.8	2019	9	1	0.83333	15		14.1		94		0		9		4				97.48						NA
-80.42	43.65	ELORA RC	6142286	43709.9	2019	9	1	0.875	14.7		14.1		96		0		12		3				97.43						NA
-80.42	43.65	ELORA RC	6142286	43709.9	2019	9	1	0.91667	13.9		13.5		98		0				0				97.41						NA
-80.42	43.65	ELORA RC	6142286	43710	2019	9	1	0.95833	14.4		14		98		0				0				97.39						NA
-80.42	43.65	ELORA RC	6142286	43710	2019	9	2	0	14.3		13.9		98		0				0				97.36						NA
-80.42	43.65	ELORA RC	6142286	43710	2019	9	2	0.04167	14.6		14.3		98		0.1		32		5				97.31						NA
-80.42	43.65	ELORA RC	6142286	43710.1	2019	9	2	0.08333	14.8		14.5		98		0.1		30		8				97.28						NA
-80.42	43.65	ELORA RC	6142286	43710.1	2019	9	2	0.125	14.7		13.6		93		0.2		32		8				97.24						NA
-80.42	43.65	ELORA RC	6142286	43710.2	2019	9	2	0.16667	13.8		12.8		94		0		30		6				97.21						NA
-80.42	43.65	ELORA RC	6142286	43710.2	2019	9	2	0.20833	13.9		12.6		92		0		31		8				97.2						NA
-80.42	43.65	ELORA RC	6142286	43710.3	2019	9	2	0.25	13.4		12.1		92		0		29		7				97.24						NA
-80.42	43.65	ELORA RC	6142286	43710.3	2019	9	2	0.29167	14.4		12.8		90		0		28		6				97.26						NA
-80.42	43.65	ELORA RC	6142286	43710.3	2019	9	2	0.33333	16.1		14.3		89		0		28		8				97.26						NA
-80.42	43.65	ELORA RC	6142286	43710.4	2019	9	2	0.375	17.6		14.7		83		0		30		11				97.27						NA
-80.42	43.65	ELORA RC	6142286	43710.4	2019	9	2	0.41667	19.3		14.7		75		0		30		14				97.27						NA
-80.42	43.65	ELORA RC	6142286	43710.5	2019	9	2	0.45833	20.3		14.5		70		0		29		13				97.28						NA
-80.42	43.65	ELORA RC	6142286	43710.5	2019	9	2	0.5	20		13.8		68		0		32		13				97.26						NA
-80.42	43.65	ELORA RC	6142286	43710.5	2019	9	2	0.54167	20.7		13.7		64		0		28		16				97.25						NA
-80.42	43.65	ELORA RC	6142286	43710.6	2019	9	2	0.58333	20.5		14.1		67		0		25		17				97.21						NA
-80.42	43.65	ELORA RC	6142286	43710.6	2019	9	2	0.625	21		13.6		62		0		28		20				97.21						NA
-80.42	43.65	ELORA RC	6142286	43710.7	2019	9	2	0.66667	20.6		14.3		67		0		30		21				97.21						NA
-80.42	43.65	ELORA RC	6142286	43710.7	2019	9	2	0.70833	19.6		15		74		0		30		18				97.25						NA
-80.42	43.65	ELORA RC	6142286	43710.8	2019	9	2	0.75	18.7		14.4		76		0		29		15				97.3						NA
-80.42	43.65	ELORA RC	6142286	43710.8	2019	9	2	0.79167	16.6		14.1		85		0		31		10				97.3						NA
-80.42	43.65	ELORA RC	6142286	43710.8	2019	9	2	0.83333	15		13.5		91		0		28		6				97.33						NA
-80.42	43.65	ELORA RC	6142286	43710.9	2019	9	2	0.875	14.2		13.2		93		0		29		8				97.4						NA
-80.42	43.65	ELORA RC	6142286	43710.9	2019	9	2	0.91667	13.7		13.1		96		0		33		8				97.43						NA
-80.42	43.65	ELORA RC	6142286	43711	2019	9	2	0.95833	13.5		12.8		95		0		33		4				97.37						NA
-80.42	43.65	ELORA RC	6142286	43711	2019	9	3	0	11.7		11.4		98		0				0				97.39						NA
-80.42	43.65	ELORA RC	6142286	43711	2019	9	3	0.04167	11.5		11.3		98		0				0				97.39						NA
-80.42	43.65	ELORA RC	6142286	43711.1	2019	9	3	0.08333	11.4		11.1		98		0		32		4				97.38						NA
-80.42	43.65	ELORA RC	6142286	43711.1	2019	9	3	0.125	10.6		10.3		98		0		2		5				97.36						NA
-80.42	43.65	ELORA RC	6142286	43711.2	2019	9	3	0.16667	10.1		9.9		98		0				0				97.39						NA
-80.42	43.65	ELORA RC	6142286	43711.2	2019	9	3	0.20833	9.2		9		98		0		4		4				97.38						NA
-80.42	43.65	ELORA RC	6142286	43711.3	2019	9	3	0.25	10.1		10		99		0		7		4				97.39						NA
-80.42	43.65	ELORA RC	6142286	43711.3	2019	9	3	0.29167	11.9		11.7		99		0		10		8				97.39						NA
-80.42	43.65	ELORA RC	6142286	43711.3	2019	9	3	0.33333	13.7		13.4		99		0		11		8				97.36						NA
-80.42	43.65	ELORA RC	6142286	43711.4	2019	9	3	0.375	14.8		14.5		98		0		14		11				97.32						NA
-80.42	43.65	ELORA RC	6142286	43711.4	2019	9	3	0.41667	16.4		13.6		84		0		17		18				97.27						NA
-80.42	43.65	ELORA RC	6142286	43711.5	2019	9	3	0.45833	17.8		14.8		83		0		16		14				97.21						NA
-80.42	43.65	ELORA RC	6142286	43711.5	2019	9	3	0.5	18.4		15.5		83		0		16		18				97.08						NA
-80.42	43.65	ELORA RC	6142286	43711.5	2019	9	3	0.54167	20.6		16.5		77		0		16		19				96.97		26				NA
-80.42	43.65	ELORA RC	6142286	43711.6	2019	9	3	0.58333	21.3		16.7		75		0		18		21				96.84		26				NA
-80.42	43.65	ELORA RC	6142286	43711.6	2019	9	3	0.625	21.3		16.8		76		0		19		27				96.75		26				NA
-80.42	43.65	ELORA RC	6142286	43711.7	2019	9	3	0.66667	22.3		16.7		70		0		19		25				96.68		27				NA
-80.42	43.65	ELORA RC	6142286	43711.7	2019	9	3	0.70833	22.4		16.3		69		0		20		26				96.6		27				NA
-80.42	43.65	ELORA RC	6142286	43711.8	2019	9	3	0.75	22.1		16.2		69		0		20		27				96.63		27				NA
-80.42	43.65	ELORA RC	6142286	43711.8	2019	9	3	0.79167	21.6		17.1		75		0		19		22				96.52		27				NA
-80.42	43.65	ELORA RC	6142286	43711.8	2019	9	3	0.83333	20.3		17.8		85		5.7		21		24				96.47		26				NA
-80.42	43.65	ELORA RC	6142286	43711.9	2019	9	3	0.875	20.7		17.6		82		0		21		25				96.42		26				NA
-80.42	43.65	ELORA RC	6142286	43711.9	2019	9	3	0.91667	20.5		17.8		85		0		22		26				96.38		26				NA
-80.42	43.65	ELORA RC	6142286	43712	2019	9	3	0.95833	21.1		18.1		83		0		22		29				96.31		27				NA
-80.42	43.65	ELORA RC	6142286	43712	2019	9	4	0	20.5		18.3		87		0		23		20				96.34		27				NA
-80.42	43.65	ELORA RC	6142286	43712	2019	9	4	0.04167	20		18.5		92		0		24		21				96.31		26				NA
-80.42	43.65	ELORA RC	6142286	43712.1	2019	9	4	0.08333	20		18.4		90		0		26		21				96.32		26				NA
-80.42	43.65	ELORA RC	6142286	43712.1	2019	9	4	0.125	18.6		16.4		87		0		28		19				96.32						NA
-80.42	43.65	ELORA RC	6142286	43712.2	2019	9	4	0.16667	16		13.4		85		0		27		11				96.35						NA
-80.42	43.65	ELORA RC	6142286	43712.2	2019	9	4	0.20833	15.7		13.5		87		0		29		13				96.41						NA
-80.42	43.65	ELORA RC	6142286	43712.3	2019	9	4	0.25	15.1		13.3		89		0		29		16				96.51						NA
-80.42	43.65	ELORA RC	6142286	43712.3	2019	9	4	0.29167	15.9		12.9		82		0		30		22				96.62						NA
-80.42	43.65	ELORA RC	6142286	43712.3	2019	9	4	0.33333	15.1		11.5		79		0		30		29				96.73						NA
-80.42	43.65	ELORA RC	6142286	43712.4	2019	9	4	0.375	14.9		11		77		0		29		28				96.84						NA
-80.42	43.65	ELORA RC	6142286	43712.4	2019	9	4	0.41667	14.8		10.9		77		0		30		25				96.94						NA
-80.42	43.65	ELORA RC	6142286	43712.5	2019	9	4	0.45833	14.9		11		77		0		30		24				97.01						NA
-80.42	43.65	ELORA RC	6142286	43712.5	2019	9	4	0.5	15.1		11.1		77		0		31		21				97.13						NA
-80.42	43.65	ELORA RC	6142286	43712.5	2019	9	4	0.54167	15.3		10.7		74		0		29		23				97.2						NA
-80.42	43.65	ELORA RC	6142286	43712.6	2019	9	4	0.58333	15.3		10.5		73		0		31		22				97.26						NA
-80.42	43.65	ELORA RC	6142286	43712.6	2019	9	4	0.625	16.3		10.5		68		0		30		21				97.29						NA
-80.42	43.65	ELORA RC	6142286	43712.7	2019	9	4	0.66667	15.8		10.3		70		0		31		19				97.33						NA
-80.42	43.65	ELORA RC	6142286	43712.7	2019	9	4	0.70833	15.7		9.8		68		0		31		20				97.36						NA
-80.42	43.65	ELORA RC	6142286	43712.8	2019	9	4	0.75	15.8		10.1		69		0		33		13				97.39						NA
-80.42	43.65	ELORA RC	6142286	43712.8	2019	9	4	0.79167	12.7		8.9		77		0		31		12				97.47						NA
-80.42	43.65	ELORA RC	6142286	43712.8	2019	9	4	0.83333	10.8		8.4		85		0		29		6				97.54						NA
-80.42	43.65	ELORA RC	6142286	43712.9	2019	9	4	0.875	10.1		8		87		0		31		5				97.57						NA
-80.42	43.65	ELORA RC	6142286	43712.9	2019	9	4	0.91667	9.5		8.5		93		0		33		6				97.58						NA
-80.42	43.65	ELORA RC	6142286	43713	2019	9	4	0.95833	9.1		7.9		93		0		1		6				97.55						NA
-80.42	43.65	ELORA RC	6142286	43713	2019	9	5	0	7.4		6.9		96		0		36		5				97.54						NA
-80.42	43.65	ELORA RC	6142286	43713	2019	9	5	0.04167	8.9		7.7		92		0		36		8				97.56						NA
-80.42	43.65	ELORA RC	6142286	43713.1	2019	9	5	0.08333	8.7		7.4		91		0		1		8				97.55						NA
-80.42	43.65	ELORA RC	6142286	43713.1	2019	9	5	0.125	8.1		7.8		98		0		36		7				97.58						NA
-80.42	43.65	ELORA RC	6142286	43713.2	2019	9	5	0.16667	8.5		6.9		90		0		1		8				97.6						NA
-80.42	43.65	ELORA RC	6142286	43713.2	2019	9	5	0.20833	6.2		5.6		96		0		2		5				97.63						NA
-80.42	43.65	ELORA RC	6142286	43713.3	2019	9	5	0.25	5.4		5		97		0		36		4				97.68						NA
-80.42	43.65	ELORA RC	6142286	43713.3	2019	9	5	0.29167	8.1		7.3		95		0				0				97.73						NA
-80.42	43.65	ELORA RC	6142286	43713.3	2019	9	5	0.33333	12.1		9.6		85		0		31		4				97.75						NA
-80.42	43.65	ELORA RC	6142286	43713.4	2019	9	5	0.375	15.4		9.1		66		0				0				97.76						NA
-80.42	43.65	ELORA RC	6142286	43713.4	2019	9	5	0.41667	16.9		8.5		58		0		25		4				97.73						NA
-80.42	43.65	ELORA RC	6142286	43713.5	2019	9	5	0.45833	17.4		7.9		54		0		34		5				97.74						NA
-80.42	43.65	ELORA RC	6142286	43713.5	2019	9	5	0.5	18.1		6.9		48		0		19		6				97.61						NA
-80.42	43.65	ELORA RC	6142286	43713.5	2019	9	5	0.54167	19.1		7.1		46		0		28		7				97.59						NA
-80.42	43.65	ELORA RC	6142286	43713.6	2019	9	5	0.58333	19.8		6.2		41		0		31		10				97.56						NA
-80.42	43.65	ELORA RC	6142286	43713.6	2019	9	5	0.625	20.8		8.3		45		0		29		7				97.51						NA
-80.42	43.65	ELORA RC	6142286	43713.7	2019	9	5	0.66667	20.8		6.4		39		0		29		7				97.47						NA
-80.42	43.65	ELORA RC	6142286	43713.7	2019	9	5	0.70833	20.2		6.2		40		0		32		6				97.46						NA
-80.42	43.65	ELORA RC	6142286	43713.8	2019	9	5	0.75	18.9		7.9		49		0		31		6				97.44						NA
-80.42	43.65	ELORA RC	6142286	43713.8	2019	9	5	0.79167	16.8		7.8		55		0		34		9				97.44						NA
-80.42	43.65	ELORA RC	6142286	43713.8	2019	9	5	0.83333	15.3		8.7		65		0		2		10				97.45						NA
-80.42	43.65	ELORA RC	6142286	43713.9	2019	9	5	0.875	13.9		9.8		76		0		5		11				97.41						NA
-80.42	43.65	ELORA RC	6142286	43713.9	2019	9	5	0.91667	13.7		10.3		80		0		4		10				97.4						NA
-80.42	43.65	ELORA RC	6142286	43714	2019	9	5	0.95833	13.8		10.3		79		0		2		11				97.37						NA
-80.42	43.65	ELORA RC	6142286	43714	2019	9	6	0	11.9		10		88		0		5		12				97.32						NA
-80.42	43.65	ELORA RC	6142286	43714	2019	9	6	0.04167	11.1		9.6		91		0		4		5				97.28						NA
-80.42	43.65	ELORA RC	6142286	43714.1	2019	9	6	0.08333	10.2		8.7		90		0		4		7				97.25						NA
-80.42	43.65	ELORA RC	6142286	43714.1	2019	9	6	0.125	10		8.9		93		0		5		8				97.21						NA
-80.42	43.65	ELORA RC	6142286	43714.2	2019	9	6	0.16667	9.6		8.4		92		0		4		5				97.19						NA
-80.42	43.65	ELORA RC	6142286	43714.2	2019	9	6	0.20833	8.7		7.7		93		0		5		7				97.15						NA
-80.42	43.65	ELORA RC	6142286	43714.3	2019	9	6	0.25	8.7		7.9		95		0		8		5				97.12						NA
-80.42	43.65	ELORA RC	6142286	43714.3	2019	9	6	0.29167	10.9		9.8		93		0		7		3				97.09						NA
-80.42	43.65	ELORA RC	6142286	43714.3	2019	9	6	0.33333	13.5		11.2		86		0				0				97.02						NA
-80.42	43.65	ELORA RC	6142286	43714.4	2019	9	6	0.375	15.8		11.9		78		0		12		6				97.02						NA
-80.42	43.65	ELORA RC	6142286	43714.4	2019	9	6	0.41667	16.8		11.5		71		0		5		5				97.03						NA
-80.42	43.65	ELORA RC	6142286	43714.5	2019	9	6	0.45833	17.4		10.9		66		0		3		6				97						NA
-80.42	43.65	ELORA RC	6142286	43714.5	2019	9	6	0.5	17.2		10.4		64		0		1		9				96.96						NA
-80.42	43.65	ELORA RC	6142286	43714.5	2019	9	6	0.54167	16		11		72		0		3		10				96.9						NA

-80.42	43.65	ELORA RC	6142286	43714.6	2019	9	6	0.58333	15.4		12.9		85		0.2		5		11				96.85						NA
-80.42	43.65	ELORA RC	6142286	43714.6	2019	9	6	0.625	15.6		13.3		86		0.4		5		11				96.83						NA
-80.42	43.65	ELORA RC	6142286	43714.7	2019	9	6	0.66667	16.8		13.3		80		0		8		14				96.84						NA
-80.42	43.65	ELORA RC	6142286	43714.7	2019	9	6	0.70833	15.9		12.1		78		0		6		13				96.85						NA
-80.42	43.65	ELORA RC	6142286	43714.8	2019	9	6	0.75	15.2		12		82		0		7		9				96.87						NA
-80.42	43.65	ELORA RC	6142286	43714.8	2019	9	6	0.79167	14.4		12.6		89		0		7		4				96.9						NA
-80.42	43.65	ELORA RC	6142286	43714.8	2019	9	6	0.83333	14		12.1		89		0		4		6				96.92						NA
-80.42	43.65	ELORA RC	6142286	43714.9	2019	9	6	0.875	13.5		11.8		89		0		1		5				96.93						NA
-80.42	43.65	ELORA RC	6142286	43714.9	2019	9	6	0.91667	13		11		87		0				0				96.9						NA
-80.42	43.65	ELORA RC	6142286	43715	2019	9	6	0.95833	12.6		10.4		87		0		3		5				96.91						NA
-80.42	43.65	ELORA RC	6142286	43715	2019	9	7	0	12.6		10.2		85		0		2		5				96.87						NA
-80.42	43.65	ELORA RC	6142286	43715	2019	9	7	0.04167	12.5		10.5		87		0				0				96.88						NA
-80.42	43.65	ELORA RC	6142286	43715.1	2019	9	7	0.08333	12.1		10.5		90		0				0				96.85						NA
-80.42	43.65	ELORA RC	6142286	43715.1	2019	9	7	0.125	12.1		11.1		93		0		29		6				96.84						NA
-80.42	43.65	ELORA RC	6142286	43715.2	2019	9	7	0.16667	12.5		11.2		92		0				0				96.86						NA
-80.42	43.65	ELORA RC	6142286	43715.2	2019	9	7	0.20833	12.4		11.6		95		0		26		3				96.89						NA
-80.42	43.65	ELORA RC	6142286	43715.3	2019	9	7	0.25	12.7		11.5		92		0		33		5				96.93						NA
-80.42	43.65	ELORA RC	6142286	43715.3	2019	9	7	0.29167	13.1		11.3		89		0		34		6				96.93						NA
-80.42	43.65	ELORA RC	6142286	43715.3	2019	9	7	0.33333	14.2		11.8		86		0		30		9				96.93						NA
-80.42	43.65	ELORA RC	6142286	43715.4	2019	9	7	0.375	14.9		12		83		0		27		13				96.94						NA
-80.42	43.65	ELORA RC	6142286	43715.4	2019	9	7	0.41667	15.7		12.2		80		0		32		13				96.94						NA
-80.42	43.65	ELORA RC	6142286	43715.5	2019	9	7	0.45833	17.1		12		72		0		30		13				96.94						NA
-80.42	43.65	ELORA RC	6142286	43715.5	2019	9	7	0.5	18.8		12.2		65		0		30		15				96.89						NA
-80.42	43.65	ELORA RC	6142286	43715.5	2019	9	7	0.54167	19.4		11		58		0		28		24				96.85						NA
-80.42	43.65	ELORA RC	6142286	43715.6	2019	9	7	0.58333	20.3		11.3		56		0		29		22				96.83						NA
-80.42	43.65	ELORA RC	6142286	43715.6	2019	9	7	0.625	19.3		10.1		55		0		30		21				96.84						NA
-80.42	43.65	ELORA RC	6142286	43715.7	2019	9	7	0.66667	19.8		10.9		56		0		28		21				96.84						NA
-80.42	43.65	ELORA RC	6142286	43715.7	2019	9	7	0.70833	18.8		10.8		60		0		29		22				96.88						NA
-80.42	43.65	ELORA RC	6142286	43715.8	2019	9	7	0.75	15.8		11.2		74		0		31		28				97						NA
-80.42	43.65	ELORA RC	6142286	43715.8	2019	9	7	0.79167	13.9		12		89		0		32		8				97						NA
-80.42	43.65	ELORA RC	6142286	43715.8	2019	9	7	0.83333	14.2		12		86		0		30		8				97.06						NA
-80.42	43.65	ELORA RC	6142286	43715.9	2019	9	7	0.875	14		11.3		84		0		29		9				97.08						NA
-80.42	43.65	ELORA RC	6142286	43715.9	2019	9	7	0.91667	13.6		10.6		82		0		29		11				97.09						NA
-80.42	43.65	ELORA RC	6142286	43716	2019	9	7	0.95833	11.4		9.9		90		0		29		7				97.11						NA
-80.42	43.65	ELORA RC	6142286	43716	2019	9	8	0	11		10.1		94		0				0				97.13						NA
-80.42	43.65	ELORA RC	6142286	43716	2019	9	8	0.04167	9.2		8.8		98		0		28		7				97.14						NA
-80.42	43.65	ELORA RC	6142286	43716.1	2019	9	8	0.08333	8.7		8.3		98		0		1		3				97.14						NA
-80.42	43.65	ELORA RC	6142286	43716.1	2019	9	8	0.125	10.7		10.4		98		0		35		11				97.17						NA
-80.42	43.65	ELORA RC	6142286	43716.2	2019	9	8	0.16667	11		10.7		98		0		35		3				97.21						NA
-80.42	43.65	ELORA RC	6142286	43716.2	2019	9	8	0.20833	9		8.6		98		0		28		4				97.28						NA
-80.42	43.65	ELORA RC	6142286	43716.3	2019	9	8	0.25	8.7		8.4		98		0		28		3				97.38						NA
-80.42	43.65	ELORA RC	6142286	43716.3	2019	9	8	0.29167	10.2		9.9		98		0		36		5				97.44						NA
-80.42	43.65	ELORA RC	6142286	43716.3	2019	9	8	0.33333	12.3		10.5		89		0		34		7				97.51						NA
-80.42	43.65	ELORA RC	6142286	43716.4	2019	9	8	0.375	13.2		9.8		80		0		33		12				97.6						NA
-80.42	43.65	ELORA RC	6142286	43716.4	2019	9	8	0.41667	14.4		10.3		76		0		31		13				97.59						NA
-80.42	43.65	ELORA RC	6142286	43716.5	2019	9	8	0.45833	15.3		8.6		64		0		1		13				97.59						NA
-80.42	43.65	ELORA RC	6142286	43716.5	2019	9	8	0.5	15.8		8.5		62		0		1		11				97.59						NA
-80.42	43.65	ELORA RC	6142286	43716.5	2019	9	8	0.54167	15.7		8.4		62		0		34		13				97.61						NA
-80.42	43.65	ELORA RC	6142286	43716.6	2019	9	8	0.58333	15.4		8.1		62		0		35		15				97.62						NA
-80.42	43.65	ELORA RC	6142286	43716.6	2019	9	8	0.625	15.6		7.7		60		0		33		11				97.65						NA
-80.42	43.65	ELORA RC	6142286	43716.7	2019	9	8	0.66667	15.2		7.3		59		0		35		9				97.65						NA
-80.42	43.65	ELORA RC	6142286	43716.7	2019	9	8	0.70833	14.5		7.8		64		0		33		9				97.72						NA
-80.42	43.65	ELORA RC	6142286	43716.8	2019	9	8	0.75	13.6		6.6		63		0		1		10				97.75						NA
-80.42	43.65	ELORA RC	6142286	43716.8	2019	9	8	0.79167	12.1		5.6		65		0		2		13				97.78						NA
-80.42	43.65	ELORA RC	6142286	43716.8	2019	9	8	0.83333	10.4		5.7		73		0		2		9				97.86						NA
-80.42	43.65	ELORA RC	6142286	43716.9	2019	9	8	0.875	9.3		5.7		78		0		3		10				97.91						NA
-80.42	43.65	ELORA RC	6142286	43716.9	2019	9	8	0.91667	8.7		5.8		82		0		2		10				97.93						NA
-80.42	43.65	ELORA RC	6142286	43717	2019	9	8	0.95833	7.1		4.8		85		0		3		6				97.95						NA
-80.42	43.65	ELORA RC	6142286	43717	2019	9	9	0	6.1		4.5		90		0		4		4				97.96						NA
-80.42	43.65	ELORA RC	6142286	43717	2019	9	9	0.04167	7.3		6.1		92		0		4		8				97.96						NA
-80.42	43.65	ELORA RC	6142286	43717.1	2019	9	9	0.08333	8.2		6.1		87		0		4		6				97.95						NA
-80.42	43.65	ELORA RC	6142286	43717.1	2019	9	9	0.125	8.2		5.9		85		0		6		10				97.96						NA
-80.42	43.65	ELORA RC	6142286	43717.2	2019	9	9	0.16667	8.3		6.2		86		0		7		11				97.97						NA
-80.42	43.65	ELORA RC	6142286	43717.2	2019	9	9	0.20833	7.8		6		88		0		5		9				98.02						NA
-80.42	43.65	ELORA RC	6142286	43717.3	2019	9	9	0.25	8		5.3		83		0		5		10				98.1						NA
-80.42	43.65	ELORA RC	6142286	43717.3	2019	9	9	0.29167	8.5		4.9		78		0		6		11				98.15						NA
-80.42	43.65	ELORA RC	6142286	43717.3	2019	9	9	0.33333	9.7		4.9		72		0		7		13				98.15						NA
-80.42	43.65	ELORA RC	6142286	43717.4	2019	9	9	0.375	12.4		6.5		67		0		9		15				98.21						NA
-80.42	43.65	ELORA RC	6142286	43717.4	2019	9	9	0.41667	14		7.8		66		0		10		13				98.22						NA
-80.42	43.65	ELORA RC	6142286	43717.5	2019	9	9	0.45833	14.8		8.7		67		0		11		7				98.23						NA
-80.42	43.65	ELORA RC	6142286	43717.5	2019	9	9	0.5	15.3		8.8		65		0		13		10				98.19						NA
-80.42	43.65	ELORA RC	6142286	43717.5	2019	9	9	0.54167	17.1		8.6		57		0		11		10				98.14						NA
-80.42	43.65	ELORA RC	6142286	43717.6	2019	9	9	0.58333	17.7		8.4		54		0		11		7				98.09						NA
-80.42	43.65	ELORA RC	6142286	43717.6	2019	9	9	0.625	17.9		8.7		55		0		14		4				98.1						NA
-80.42	43.65	ELORA RC	6142286	43717.7	2019	9	9	0.66667	17.9		8.4		54		0		14		9				98.09						NA
-80.42	43.65	ELORA RC	6142286	43717.7	2019	9	9	0.70833	17.7		8.3		54		0		14		7				98.1						NA
-80.42	43.65	ELORA RC	6142286	43717.8	2019	9	9	0.75	16.7		9.1		61		0		13		5				98.1						NA
-80.42	43.65	ELORA RC	6142286	43717.8	2019	9	9	0.79167	14.2		9.5		73		0		12		8				98.05						NA
-80.42	43.65	ELORA RC	6142286	43717.8	2019	9	9	0.83333	12.8		9.3		79		0		12		5				98.13						NA
-80.42	43.65	ELORA RC	6142286	43717.9	2019	9	9	0.875	11.6		9.6		87		0		13		6				98.1						NA
-80.42	43.65	ELORA RC	6142286	43717.9	2019	9	9	0.91667	11.3		9.3		88		0		11		5				98.11						NA
-80.42	43.65	ELORA RC	6142286	43718	2019	9	9	0.95833	10.2		8.8		91		0		6		4				98.14						NA
-80.42	43.65	ELORA RC	6142286	43718	2019	9	10	0	8.6		8		96		0		11		3				98.13						NA
-80.42	43.65	ELORA RC	6142286	43718	2019	9	10	0.04167	8.6		7.7		95		0		3		3				98.17						NA
-80.42	43.65	ELORA RC	6142286	43718.1	2019	9	10	0.08333	8.2		7.5		96		0		8		7				98.08						NA
-80.42	43.65	ELORA RC	6142286	43718.1	2019	9	10	0.125	9.1		8.3		94		0		9		9				98.02						NA
-80.42	43.65	ELORA RC	6142286	43718.2	2019	9	10	0.16667	9.7		8.5		92		0		10		10				97.97						NA
-80.42	43.65	ELORA RC	6142286	43718.2	2019	9	10	0.20833	9.4		8.2		92		0		9		8				97.96						NA
-80.42	43.65	ELORA RC	6142286	43718.3	2019	9	10	0.25	9.6		8.6		93		0		9		10				97.97						NA
-80.42	43.65	ELORA RC	6142286	43718.3	2019	9	10	0.29167	11.8		10.3		90		0		9		9				97.98						NA
-80.42	43.65	ELORA RC	6142286	43718.3	2019	9	10	0.33333	14.1		11.6		85		0		11		8				97.96						NA
-80.42	43.65	ELORA RC	6142286	43718.4	2019	9	10	0.375	16.4		12.3		77		0		15		14				97.89						NA
-80.42	43.65	ELORA RC	6142286	43718.4	2019	9	10	0.41667	18.5		12.8		70		0		16		14				97.84						NA
-80.42	43.65	ELORA RC	6142286	43718.5	2019	9	10	0.45833	19.9		14		69		0		18		19				97.77						NA
-80.42	43.65	ELORA RC	6142286	43718.5	2019	9	10	0.5	20.6		15.1		71		0		20		18				97.72		25				NA
-80.42	43.65	ELORA RC	6142286	43718.5	2019	9	10	0.54167	22.3		16.4		69		0		20		19				97.67		27				NA
-80.42	43.65	ELORA RC	6142286	43718.6	2019	9	10	0.58333	24.4		17.9		67		0		22		20				97.62		30				NA
-80.42	43.65	ELORA RC	6142286	43718.6	2019	9	10	0.625	24.1		17.9		68		0		21		21				97.53		30				NA
-80.42	43.65	ELORA RC	6142286	43718.7	2019	9	10	0.66667	23.7		17.9		70		0		21		22				97.49		30				NA
-80.42	43.65	ELORA RC	6142286	43718.7	2019	9	10	0.70833	23.3		18.1		73		0		20		22				97.46		29				NA
-80.42	43.65	ELORA RC	6142286	43718.8	2019	9	10	0.75	22.2		17.8		76		0		22		19				97.48		28				NA
-80.42	43.65	ELORA RC	6142286	43718.8	2019	9	10	0.79167	20.9		17.7		82		0		22		6				97.46		27				NA
-80.42	43.65	ELORA RC	6142286	43718.8	2019	9	10	0.83333	20.7		17.5		82		0		20		10				97.42		26				NA
-80.42	43.65	ELORA RC	6142286	43718.9	2019	9	10	0.875	20.6		17.3		81		0		20		11				97.38		26				NA
-80.42	43.65	ELORA RC	6142286	43718.9	2019	9	10	0.91667	21		18.5		86		0		31		25				97.43		27				NA
-80.42	43.65	ELORA RC	6142286	43719	2019	9	10	0.95833	20.2		19.2		94		13.6		25		13				97.39		27				NA
-80.42	43.65	ELORA RC	6142286	43719	2019	9	11	0	19.8		18.7		93		0.2		27		8				97.4						NA
-80.42	43.65	ELORA RC	6142286	43719	2019	9	11	0.04167	18.8		18.1		96		0		25		5				97.43						NA
-80.42	43.65	ELORA RC	6142286	43719.1	2019	9	11	0.08333	17.8		17.3		97		0		24		7				97.44						NA
-80.42	43.65	ELORA RC	6142286	43719.1	2019	9	11	0.125	17.6		17.3		98		0		23		6				97.45						NA
-80.42	43.65	ELORA RC	6142286	43719.2	2019	9	11	0.16667	17.1		16.8		98		0				0				97.47						NA
-80.42	43.65	ELORA RC	6142286	43719.2	2019	9	11	0.20833	17		16.8		98		0		27		5				97.51						NA
-80.42	43.65	ELORA RC	6142286	43719.3	2019	9	11	0.25	17.8		17.6		98		0		28		6				97.54						NA
-80.42	43.65	ELORA RC	6142286	43719.3	2019	9	11	0.29167	18.9		18.6		98		0		27		7				97.63						NA
-80.42	43.65	ELORA RC	6142286	43719.3	2019	9	11	0.33333	19.8		18.8		94		0		30		10				97.61						NA
-80.42	43.65	ELORA RC	6142286	43719.4	2019	9	11	0.375	21.1		19.6		91		0		29		12				97.63		28				NA
-80.42	43.65	ELORA RC	6142286	43719.4	2019	9	11	0.41667	22.5		19.7		84		0		28		12				97.62		30				NA
-80.42	43.65	ELORA RC	6142286	43719.5	2019	9	11	0.45833	23.3		19.8		81		0		31		13				97.62		31				NA
-80.42	43.65	ELORA RC	6142286	43719.5	2019	9	11	0.5	23.8		18.2		71		0		29		16				97.61		30				NA
-80.42	43.65	ELORA RC	6142286	43719.5	2019	9	11	0.54167	25		18.5		67		0		27		17				97.59		31				NA
-80.42	43.65	ELORA RC	6142286	43719.6	2019	9	11	0.58333	25.6		18.9		67		0		28		13				97.56		32				NA
-80.42	43.65	ELORA RC	6142286	43719.6	2019	9	11	0.625	25.1		18.1		65		0		29		9				97.46		31				NA
-80.42	43.65	ELORA RC	6142286	43719.7	2019	9	11	0.66667	25.3		19.4		70		0		32		7				97.45		32				NA
-80.42	43.65	ELORA RC	6142286	43719.7	2019	9	11	0.70833	23.9		19.3		75		0		32		6				97.47		31				NA
-80.42	43.65	ELORA RC	6142286	43719.8	2019	9	11	0.75	22		18.7		82		0		32		15				97.58		29				NA
-80.42	43.65	ELORA RC	6142286	43719.8	2019	9	11	0.79167	19.9		19.1		95		5.1		15		12				97.47						NA
-80.42	43.65	ELORA RC	6142286	43719.8	2019	9	11	0.83333	18.4		17.6		95		0.9				0				97.4						NA
-80.42	43.65	ELORA RC	6142286	43719.9	2019	9	11	0.875	18.7		18.1		97		0		1		7				97.44						NA
-80.42	43.65	ELORA RC	6142286	43719.9	2019	9	11	0.91667	18		17		94		0		4		16				97.49						NA
-80.42	43.65	ELORA RC	6142286	43720	2019	9	11	0.95833	16.9		15.8		93		0		6		12				97.52						NA
-80.42	43.65	ELORA RC	6142286	43720	2019	9	12	0	16.6		15.7		94		0		7		12				97.48						NA
-80.42	43.65	ELORA RC	6142286	43720	2019	9	12	0.04167	16.3		15.4		94		1.1		7		15				97.45						NA
-80.42	43.65	ELORA RC	6142286	43720.1	2019	9	12	0.08333	16.2		15.4		95		0.1		5		11				97.44						NA
-80.42	43.65	ELORA RC	6142286	43720.1	2019	9	12	0.125	16		15.3		95		0.5		7		16				97.49						NA
-80.42	43.65	ELORA RC	6142286	43720.2	2019	9	12	0.16667	14.9		14		94		0.7		6		18				97.55						NA
-80.42	43.65	ELORA RC	6142286	43720.2	2019	9	12	0.20833	14.1		13		93		0		7		17				97.63						NA
-80.42	43.65	ELORA RC	6142286	43720.3	2019	9	12	0.25	13.3		12.5		95		0.3		7		13				97.71						NA
-80.42	43.65	ELORA RC	6142286	43720.3	2019	9	12	0.29167	12.7		11.8		95		0.8		6		18				97.78						NA

-80.42	43.65	ELORA RC	6142286	43720.3	2019	9	12	0.33333	12.3		11.5		95		1.9		6		15				97.88						NA
-80.42	43.65	ELORA RC	6142286	43720.4	2019	9	12	0.375	12.6		11.1		90		0.4		7		17				97.89						NA
-80.42	43.65	ELORA RC	6142286	43720.4	2019	9	12	0.41667	13		11.2		89		0		6		24				97.93						NA
-80.42	43.65	ELORA RC	6142286	43720.5	2019	9	12	0.45833	13.2		11.4		88		0		7		18				97.97						NA
-80.42	43.65	ELORA RC	6142286	43720.5	2019	9	12	0.5	14.5		11.7		84		0		9		15				97.97						NA
-80.42	43.65	ELORA RC	6142286	43720.5	2019	9	12	0.54167	15.6		11.6		77		0		8		17				97.99						NA
-80.42	43.65	ELORA RC	6142286	43720.6	2019	9	12	0.58333	16		11.4		74		0		8		15				98						NA
-80.42	43.65	ELORA RC	6142286	43720.6	2019	9	12	0.625	17.1		11		67		0		9		11				98.01						NA
-80.42	43.65	ELORA RC	6142286	43720.7	2019	9	12	0.66667	17.8		11.8		68		0		8		13				98.03						NA
-80.42	43.65	ELORA RC	6142286	43720.7	2019	9	12	0.70833	18.1		11.7		66		0		8		15				98.04						NA
-80.42	43.65	ELORA RC	6142286	43720.8	2019	9	12	0.75	16.3		11.3		73		0		11		11				98.05						NA
-80.42	43.65	ELORA RC	6142286	43720.8	2019	9	12	0.79167	13.8		11.2		85		0		10		11				98.07						NA
-80.42	43.65	ELORA RC	6142286	43720.8	2019	9	12	0.83333	12.6		10.6		88		0		9		10				98.11						NA
-80.42	43.65	ELORA RC	6142286	43720.9	2019	9	12	0.875	11.1		10.2		94		0		7		8				98.17						NA
-80.42	43.65	ELORA RC	6142286	43720.9	2019	9	12	0.91667	11.5		10.7		95		0		9		8				98.18						NA
-80.42	43.65	ELORA RC	6142286	43721	2019	9	12	0.95833	11		10.3		96		0		8		8				98.17						NA
-80.42	43.65	ELORA RC	6142286	43721	2019	9	13	0	11.2		10.4		95		0		9		7				98.17						NA
-80.42	43.65	ELORA RC	6142286	43721	2019	9	13	0.04167	10.8		10		95		0		9		11				98.14						NA
-80.42	43.65	ELORA RC	6142286	43721.1	2019	9	13	0.08333	11		10.2		95		0		10		10				98.08						NA
-80.42	43.65	ELORA RC	6142286	43721.1	2019	9	13	0.125	10.5		9.2		92		0		10		7				98.02						NA
-80.42	43.65	ELORA RC	6142286	43721.2	2019	9	13	0.16667	11.5		9.8		89		0		9		15				97.98						NA
-80.42	43.65	ELORA RC	6142286	43721.2	2019	9	13	0.20833	11.8		10.2		90		0		9		14				97.96						NA
-80.42	43.65	ELORA RC	6142286	43721.3	2019	9	13	0.25	12.1		11.1		94		0		9		13				97.96						NA
-80.42	43.65	ELORA RC	6142286	43721.3	2019	9	13	0.29167	13.7		12		90		0		11		17				97.92						NA
-80.42	43.65	ELORA RC	6142286	43721.3	2019	9	13	0.33333	13.8		12.3		90		0		12		14				97.95						NA
-80.42	43.65	ELORA RC	6142286	43721.4	2019	9	13	0.375	14		12.5		91		0		12		17				97.91						NA
-80.42	43.65	ELORA RC	6142286	43721.4	2019	9	13	0.41667	14.6		13		90		0		11		17				97.85						NA
-80.42	43.65	ELORA RC	6142286	43721.5	2019	9	13	0.45833	15.5		13.5		88		0		12		15				97.78						NA
-80.42	43.65	ELORA RC	6142286	43721.5	2019	9	13	0.5	15.9		14.2		89		0.3		13		14				97.72						NA
-80.42	43.65	ELORA RC	6142286	43721.5	2019	9	13	0.54167	16.6		14.6		88		0		13		17				97.64						NA
-80.42	43.65	ELORA RC	6142286	43721.6	2019	9	13	0.58333	17.5		15.4		87		0		13		18				97.56						NA
-80.42	43.65	ELORA RC	6142286	43721.6	2019	9	13	0.625	18.2		16		87		0		13		20				97.47						NA
-80.42	43.65	ELORA RC	6142286	43721.7	2019	9	13	0.66667	19.8		17.3		86		0		13		18				97.39						NA
-80.42	43.65	ELORA RC	6142286	43721.7	2019	9	13	0.70833	20.5		18.1		86		0		14		18				97.3		27				NA
-80.42	43.65	ELORA RC	6142286	43721.8	2019	9	13	0.75	20.2		18.3		89		0		14		13				97.26		26				NA
-80.42	43.65	ELORA RC	6142286	43721.8	2019	9	13	0.79167	20		18.2		90		0		14		22				97.12		26				NA
-80.42	43.65	ELORA RC	6142286	43721.8	2019	9	13	0.83333	19.2		18.1		93		3.5		15		17				97.08						NA
-80.42	43.65	ELORA RC	6142286	43721.9	2019	9	13	0.875	19		18.3		96		5.6		18		20				97.2						NA
-80.42	43.65	ELORA RC	6142286	43721.9	2019	9	13	0.91667	19.3		18.6		96		0.1		19		18				97.21						NA
-80.42	43.65	ELORA RC	6142286	43722	2019	9	13	0.95833	19.3		18.7		96		0		22		16				97.23						NA
-80.42	43.65	ELORA RC	6142286	43722	2019	9	14	0	19.2		18.4		95		0		21		15				97.27						NA
-80.42	43.65	ELORA RC	6142286	43722	2019	9	14	0.04167	18.6		16.9		90		0		24		14				97.28						NA
-80.42	43.65	ELORA RC	6142286	43722.1	2019	9	14	0.08333	17.1		14.8		86		0		25		19				97.26						NA
-80.42	43.65	ELORA RC	6142286	43722.1	2019	9	14	0.125	15.6		13.1		85		0		26		21				97.3						NA
-80.42	43.65	ELORA RC	6142286	43722.2	2019	9	14	0.16667	13.7		12.1		90		0		24		13				97.34						NA
-80.42	43.65	ELORA RC	6142286	43722.2	2019	9	14	0.20833	13.1		11.8		92		0		24		13				97.37						NA
-80.42	43.65	ELORA RC	6142286	43722.3	2019	9	14	0.25	12.5		11.2		92		0		25		12				97.4						NA
-80.42	43.65	ELORA RC	6142286	43722.3	2019	9	14	0.29167	13.3		11.5		89		0		24		14				97.45						NA
-80.42	43.65	ELORA RC	6142286	43722.3	2019	9	14	0.33333	14		12		87		0		22		14				97.5						NA
-80.42	43.65	ELORA RC	6142286	43722.4	2019	9	14	0.375	15.5		11.7		78		0		24		22				97.55						NA
-80.42	43.65	ELORA RC	6142286	43722.4	2019	9	14	0.41667	16.5		12.5		77		0		25		21				97.56						NA
-80.42	43.65	ELORA RC	6142286	43722.5	2019	9	14	0.45833	17.4		11.8		70		0		26		27				97.57						NA
-80.42	43.65	ELORA RC	6142286	43722.5	2019	9	14	0.5	17.8		10.1		61		0		26		31				97.59						NA
-80.42	43.65	ELORA RC	6142286	43722.5	2019	9	14	0.54167	18.8		9.8		56		0		26		24				97.58						NA
-80.42	43.65	ELORA RC	6142286	43722.6	2019	9	14	0.58333	19.3		9.4		53		0		26		27				97.56						NA
-80.42	43.65	ELORA RC	6142286	43722.6	2019	9	14	0.625	19.3		9.8		54		0		26		23				97.58						NA
-80.42	43.65	ELORA RC	6142286	43722.7	2019	9	14	0.66667	19.2		9.2		52		0		26		25				97.58						NA
-80.42	43.65	ELORA RC	6142286	43722.7	2019	9	14	0.70833	18.7		9.2		54		0		26		21				97.61						NA
-80.42	43.65	ELORA RC	6142286	43722.8	2019	9	14	0.75	17		9.3		60		0		25		13				97.62						NA
-80.42	43.65	ELORA RC	6142286	43722.8	2019	9	14	0.79167	13.5		9.6		77		0		25		6				97.66						NA
-80.42	43.65	ELORA RC	6142286	43722.8	2019	9	14	0.83333	12.1		9		81		0		24		7				97.67						NA
-80.42	43.65	ELORA RC	6142286	43722.9	2019	9	14	0.875	12.1		10		87		0		25		6				97.69						NA
-80.42	43.65	ELORA RC	6142286	43722.9	2019	9	14	0.91667	11.5		9.7		88		0				0				97.71						NA
-80.42	43.65	ELORA RC	6142286	43723	2019	9	14	0.95833	10.5		9.5		94		0		32		3				97.72						NA
-80.42	43.65	ELORA RC	6142286	43723	2019	9	15	0	9.3		8.3		94		0		27		3				97.71						NA
-80.42	43.65	ELORA RC	6142286	43723	2019	9	15	0.04167	9.2		8.6		96		0				0				97.7						NA
-80.42	43.65	ELORA RC	6142286	43723.1	2019	9	15	0.08333	9.2		8.3		94		0		34		3				97.7						NA
-80.42	43.65	ELORA RC	6142286	43723.1	2019	9	15	0.125	8.8		8.1		96		0		8		4				97.66						NA
-80.42	43.65	ELORA RC	6142286	43723.2	2019	9	15	0.16667	8.7		8.2		96		0				0				97.68						NA
-80.42	43.65	ELORA RC	6142286	43723.2	2019	9	15	0.20833	7.8		7.2		96		0		34		5				97.71						NA
-80.42	43.65	ELORA RC	6142286	43723.3	2019	9	15	0.25	9.1		8.7		97		0				0				97.72						NA
-80.42	43.65	ELORA RC	6142286	43723.3	2019	9	15	0.29167	11		10.4		96		0				0				97.71						NA
-80.42	43.65	ELORA RC	6142286	43723.3	2019	9	15	0.33333	12		11.2		94		0				0				97.71						NA
-80.42	43.65	ELORA RC	6142286	43723.4	2019	9	15	0.375	14.1		10.5		79		0		3		11				97.77						NA
-80.42	43.65	ELORA RC	6142286	43723.4	2019	9	15	0.41667	13.1		11.1		88		1.6		4		6				97.77						NA
-80.42	43.65	ELORA RC	6142286	43723.5	2019	9	15	0.45833	14.3		12		86		0		16		7				97.71						NA
-80.42	43.65	ELORA RC	6142286	43723.5	2019	9	15	0.5	14		12.5		91		0.4		13		5				97.68						NA
-80.42	43.65	ELORA RC	6142286	43723.5	2019	9	15	0.54167	15.2		13.1		87		0.6		16		10				97.65						NA
-80.42	43.65	ELORA RC	6142286	43723.6	2019	9	15	0.58333	16.4		13.6		83		0		15		7				97.57						NA
-80.42	43.65	ELORA RC	6142286	43723.6	2019	9	15	0.625	16.2		13.7		85		0		12		6				97.53						NA
-80.42	43.65	ELORA RC	6142286	43723.7	2019	9	15	0.66667	15.8		14.2		91		0		8		11				97.49						NA
-80.42	43.65	ELORA RC	6142286	43723.7	2019	9	15	0.70833	15.2		14.4		95		1.2		10		8				97.48						NA
-80.42	43.65	ELORA RC	6142286	43723.8	2019	9	15	0.75	14.9		14.2		96		0.4		8		9				97.48						NA
-80.42	43.65	ELORA RC	6142286	43723.8	2019	9	15	0.79167	14.4		13.8		96		0.1		12		6				97.5						NA
-80.42	43.65	ELORA RC	6142286	43723.8	2019	9	15	0.83333	13.9		13.3		96		0		11		7				97.51						NA
-80.42	43.65	ELORA RC	6142286	43723.9	2019	9	15	0.875	13.8		13.2		96		0		10		8				97.51						NA
-80.42	43.65	ELORA RC	6142286	43723.9	2019	9	15	0.91667	13.8		13.1		96		0		8		4				97.52						NA
-80.42	43.65	ELORA RC	6142286	43724	2019	9	15	0.95833	13.7		13.1		96		0		10		5				97.53						NA
-80.42	43.65	ELORA RC	6142286	43724	2019	9	16	0	13.7		13.3		97		0		10		5				97.5						NA
-80.42	43.65	ELORA RC	6142286	43724	2019	9	16	0.04167	13.9		13.4		97		0		13		7				97.45						NA
-80.42	43.65	ELORA RC	6142286	43724.1	2019	9	16	0.08333	13.9		13.3		96		0		11		4				97.48						NA
-80.42	43.65	ELORA RC	6142286	43724.1	2019	9	16	0.125	13.7		13.1		96		0		9		9				97.46						NA
-80.42	43.65	ELORA RC	6142286	43724.2	2019	9	16	0.16667	13.7		13.1		96		0		8		8				97.46						NA
-80.42	43.65	ELORA RC	6142286	43724.2	2019	9	16	0.20833	13.8		13.2		96		0		9		10				97.46						NA
-80.42	43.65	ELORA RC	6142286	43724.3	2019	9	16	0.25	13.9		13.3		96		0		11		6				97.51						NA
-80.42	43.65	ELORA RC	6142286	43724.3	2019	9	16	0.29167	14.1		13.4		95		0		9		7				97.55						NA
-80.42	43.65	ELORA RC	6142286	43724.3	2019	9	16	0.33333	14.9		13.8		93		0		11		8				97.56						NA
-80.42	43.65	ELORA RC	6142286	43724.4	2019	9	16	0.375	15.1		13.8		92		0		7		6				97.6						NA
-80.42	43.65	ELORA RC	6142286	43724.4	2019	9	16	0.41667	16.4		14.1		86		0		12		11				97.57						NA
-80.42	43.65	ELORA RC	6142286	43724.5	2019	9	16	0.45833	16.2		14.1		87		0		12		7				97.57						NA
-80.42	43.65	ELORA RC	6142286	43724.5	2019	9	16	0.5	16		14.7		92		0.1		9		9				97.57						NA
-80.42	43.65	ELORA RC	6142286	43724.5	2019	9	16	0.54167	16.3		14.8		91		0.8		11		10				97.55						NA
-80.42	43.65	ELORA RC	6142286	43724.6	2019	9	16	0.58333	16.4		14.6		89		0		8		11				97.54						NA
-80.42	43.65	ELORA RC	6142286	43724.6	2019	9	16	0.625	16.5		14.9		90		0		10		8				97.53						NA
-80.42	43.65	ELORA RC	6142286	43724.7	2019	9	16	0.66667	16		14.7		92		0		8		7				97.55						NA
-80.42	43.65	ELORA RC	6142286	43724.7	2019	9	16	0.70833	16		14.8		92		0		9		8				97.54						NA
-80.42	43.65	ELORA RC	6142286	43724.8	2019	9	16	0.75	15.6		14.5		93		0		9		12				97.57						NA
-80.42	43.65	ELORA RC	6142286	43724.8	2019	9	16	0.79167	15		14.1		94		0		7		9				97.6						NA
-80.42	43.65	ELORA RC	6142286	43724.8	2019	9	16	0.83333	14.6		13.9		95		0		7		7				97.64						NA
-80.42	43.65	ELORA RC	6142286	43724.9	2019	9	16	0.875	13.3		12.7		96		0				0				97.64						NA
-80.42	43.65	ELORA RC	6142286	43724.9	2019	9	16	0.91667	12.6		12.3		98		0		2		5				97.68						NA
-80.42	43.65	ELORA RC	6142286	43725	2019	9	16	0.95833	12.9		12		94		0		1		6				97.7						NA
-80.42	43.65	ELORA RC	6142286	43725	2019	9	17	0	12.4		11.7		95		0		1		4				97.73						NA
-80.42	43.65	ELORA RC	6142286	43725	2019	9	17	0.04167	11.4		10.8		96		0		3		5				97.76						NA
-80.42	43.65	ELORA RC	6142286	43725.1	2019	9	17	0.08333	10.9		10.5		97		0		3		7				97.75						NA
-80.42	43.65	ELORA RC	6142286	43725.1	2019	9	17	0.125	10.7		10.4		98		0		4		5				97.76						NA
-80.42	43.65	ELORA RC	6142286	43725.2	2019	9	17	0.16667	9.6		9.2		98		0		2		4				97.76						NA
-80.42	43.65	ELORA RC	6142286	43725.2	2019	9	17	0.20833	9.4		9		98		0		3		6				97.8						NA
-80.42	43.65	ELORA RC	6142286	43725.3	2019	9	17	0.25	10.4		9.9		97		0		2		6				97.88						NA
-80.42	43.65	ELORA RC	6142286	43725.3	2019	9	17	0.29167	11.2		10.1		93		0		3		7				97.92						NA
-80.42	43.65	ELORA RC	6142286	43725.3	2019	9	17	0.33333	14.1		12.1		88		0		3		6				97.97						NA
-80.42	43.65	ELORA RC	6142286	43725.4	2019	9	17	0.375	17		12.1		73		0		5		6				98						NA
-80.42	43.65	ELORA RC	6142286	43725.4	2019	9	17	0.41667	18.8		10.9		60		0		8		8				97.98						NA
-80.42	43.65	ELORA RC	6142286	43725.5	2019	9	17	0.45833	20.1		11		56		0		11		9				97.95						NA
-80.42	43.65	ELORA RC	6142286	43725.5	2019	9	17	0.5	20.5		10.1		51		0		11		11				97.94						NA
-80.42	43.65	ELORA RC	6142286	43725.5	2019	9	17	0.54167	21.3		10.1		49		0		7		6				97.92						NA
-80.42	43.65	ELORA RC	6142286	43725.6	2019	9	17	0.58333	21.3		9.8		48		0		1		6				97.89						NA
-80.42	43.65	ELORA RC	6142286	43725.6	2019	9	17	0.625	22.3		10.8		48		0		13		3				97.88						NA
-80.42	43.65	ELORA RC	6142286	43725.7	2019	9	17	0.66667	22.1		11.8		52		0		33		5				97.84						NA
-80.42	43.65	ELORA RC	6142286	43725.7	2019	9	17	0.70833	21.8		10.6		49		0		9		4				97.83						NA
-80.42	43.65	ELORA RC	6142286	43725.8	2019	9	17	0.75	20.3		12		59		0		13		5				97.8						NA
-80.42	43.65	ELORA RC	6142286	43725.8	2019	9	17	0.79167	17.2		11.8		70		0		14		8				97.82						NA
-80.42	43.65	ELORA RC	6142286	43725.8	2019	9	17	0.83333	15.1		11.5		79		0				0				97.86						NA
-80.42	43.65	ELORA RC	6142286	43725.9	2019	9	17	0.875	12.9		11.2		89		0		5		3				97.88						NA
-80.42	43.65	ELORA RC	6142286	43725.9	2019	9	17	0.91667	12.5		10.4		87		0		6		4				97.91						NA
-80.42	43.65	ELORA RC	6142286	43726	2019	9	17	0.95833	11.2		9.9		92		0		4		3				97.92						NA
-80.42	43.65	ELORA RC	6142286	43726	2019	9	18	0	10.8		9.4		91		0		5		5				97.92						NA
-80.42	43.65	ELORA RC	6142286	43726	2019	9	18	0.04167	10.3		9		91		0		2		4				97.96						NA

-80.42	43.65	ELORA RC	6142286	43726.1	2019	9	18	0.08333	10.3		8.7		90		0				0				97.94						NA
-80.42	43.65	ELORA RC	6142286	43726.1	2019	9	18	0.125	10.6		8.5		87		0		2		4				97.95						NA
-80.42	43.65	ELORA RC	6142286	43726.2	2019	9	18	0.16667	10.4		8.9		90		0		2		4				97.96						NA
-80.42	43.65	ELORA RC	6142286	43726.2	2019	9	18	0.20833	9.1		8		93		0		5		5				97.94						NA
-80.42	43.65	ELORA RC	6142286	43726.3	2019	9	18	0.25	9.7		8.5		92		0		3		5				98						NA
-80.42	43.65	ELORA RC	6142286	43726.3	2019	9	18	0.29167	11.4		9.8		90		0				0				98.05						NA
-80.42	43.65	ELORA RC	6142286	43726.3	2019	9	18	0.33333	15.3		11.3		77		0		12		7				98.01						NA
-80.42	43.65	ELORA RC	6142286	43726.4	2019	9	18	0.375	17.5		11.3		67		0		14		8				98.08						NA
-80.42	43.65	ELORA RC	6142286	43726.4	2019	9	18	0.41667	19.1		10.3		57		0		14		15				98.06						NA
-80.42	43.65	ELORA RC	6142286	43726.5	2019	9	18	0.45833	20.5		10.3		52		0		16		11				98.07						NA
-80.42	43.65	ELORA RC	6142286	43726.5	2019	9	18	0.5	21.4		11.4		53		0		15		13				98.09						NA
-80.42	43.65	ELORA RC	6142286	43726.5	2019	9	18	0.54167	21.8		11.4		52		0		17		16				98.07						NA
-80.42	43.65	ELORA RC	6142286	43726.6	2019	9	18	0.58333	22.1		11.9		52		0		15		12				98.05						NA
-80.42	43.65	ELORA RC	6142286	43726.6	2019	9	18	0.625	22.4		10.4		47		0		17		13				98.04						NA
-80.42	43.65	ELORA RC	6142286	43726.7	2019	9	18	0.66667	22.2		10.6		48		0		17		11				98.03						NA
-80.42	43.65	ELORA RC	6142286	43726.7	2019	9	18	0.70833	21.4		11.1		52		0		12		12				98.01						NA
-80.42	43.65	ELORA RC	6142286	43726.8	2019	9	18	0.75	19		10.9		59		0		11		10				97.99						NA
-80.42	43.65	ELORA RC	6142286	43726.8	2019	9	18	0.79167	16.9		10.8		67		0		11		7				98.01						NA
-80.42	43.65	ELORA RC	6142286	43726.8	2019	9	18	0.83333	15.2		11		76		0		10		9				98.04						NA
-80.42	43.65	ELORA RC	6142286	43726.9	2019	9	18	0.875	13.8		11.9		88		0		9		11				98.03						NA
-80.42	43.65	ELORA RC	6142286	43726.9	2019	9	18	0.91667	13.2		11.4		89		0		9		8				98.03						NA
-80.42	43.65	ELORA RC	6142286	43727	2019	9	18	0.95833	13.3		11.3		88		0		12		6				97.99						NA
-80.42	43.65	ELORA RC	6142286	43727	2019	9	19	0	13.1		11.5		90		0		11		6				97.99						NA
-80.42	43.65	ELORA RC	6142286	43727	2019	9	19	0.04167	12.3		11.4		94		0		9		8				98.03						NA
-80.42	43.65	ELORA RC	6142286	43727.1	2019	9	19	0.08333	13.6		11.9		89		0		12		7				97.97						NA
-80.42	43.65	ELORA RC	6142286	43727.1	2019	9	19	0.125	13		11.4		90		0		12		8				97.94						NA
-80.42	43.65	ELORA RC	6142286	43727.2	2019	9	19	0.16667	12.6		11.2		91		0		11		6				97.94						NA
-80.42	43.65	ELORA RC	6142286	43727.2	2019	9	19	0.20833	11.3		10.6		95		0				0				97.97						NA
-80.42	43.65	ELORA RC	6142286	43727.3	2019	9	19	0.25	9.9		9.4		97		0		6		5				98.01						NA
-80.42	43.65	ELORA RC	6142286	43727.3	2019	9	19	0.29167	11.8		11.3		96		0		8		5				98.01						NA
-80.42	43.65	ELORA RC	6142286	43727.3	2019	9	19	0.33333	15.4		12.5		83		0		14		8				98.01						NA
-80.42	43.65	ELORA RC	6142286	43727.4	2019	9	19	0.375	17.7		11.1		65		0		17		12				98						NA
-80.42	43.65	ELORA RC	6142286	43727.4	2019	9	19	0.41667	19.5		11.8		61		0		18		16				97.99						NA
-80.42	43.65	ELORA RC	6142286	43727.5	2019	9	19	0.45833	20.2		9.5		50		0		18		16				97.99						NA
-80.42	43.65	ELORA RC	6142286	43727.5	2019	9	19	0.5	21.2		9.8		48		0		19		16				97.95						NA
-80.42	43.65	ELORA RC	6142286	43727.5	2019	9	19	0.54167	21.6		9.5		46		0		16		13				97.94						NA
-80.42	43.65	ELORA RC	6142286	43727.6	2019	9	19	0.58333	22		9.3		44		0		17		17				97.89						NA
-80.42	43.65	ELORA RC	6142286	43727.6	2019	9	19	0.625	21.7		8.7		43		0		16		14				97.84						NA
-80.42	43.65	ELORA RC	6142286	43727.7	2019	9	19	0.66667	21.7		9.6		46		0		16		16				97.83						NA
-80.42	43.65	ELORA RC	6142286	43727.7	2019	9	19	0.70833	20.8		9.2		47		0		15		15				97.81						NA
-80.42	43.65	ELORA RC	6142286	43727.8	2019	9	19	0.75	17.6		9.1		57		0		14		11				97.81						NA
-80.42	43.65	ELORA RC	6142286	43727.8	2019	9	19	0.79167	16.6		8.5		59		0		13		7				97.83						NA
-80.42	43.65	ELORA RC	6142286	43727.8	2019	9	19	0.83333	15		10.5		75		0		12		8				97.85						NA
-80.42	43.65	ELORA RC	6142286	43727.9	2019	9	19	0.875	14.1		11.9		86		0		11		9				97.86						NA
-80.42	43.65	ELORA RC	6142286	43727.9	2019	9	19	0.91667	12.5		11.4		93		0		9		5				97.87						NA
-80.42	43.65	ELORA RC	6142286	43728	2019	9	19	0.95833	11.3		10.4		94		0		8		6				97.82						NA
-80.42	43.65	ELORA RC	6142286	43728	2019	9	20	0	11.5		10.7		95		0		9		7				97.77						NA
-80.42	43.65	ELORA RC	6142286	43728	2019	9	20	0.04167	10.8		9.3		91		0				0				97.8						NA
-80.42	43.65	ELORA RC	6142286	43728.1	2019	9	20	0.08333	11.4		10.4		93		0		6		6				97.77						NA
-80.42	43.65	ELORA RC	6142286	43728.1	2019	9	20	0.125	10.8		10		95		0		4		4				97.78						NA
-80.42	43.65	ELORA RC	6142286	43728.2	2019	9	20	0.16667	10.2		9.7		97		0		7		6				97.79						NA
-80.42	43.65	ELORA RC	6142286	43728.2	2019	9	20	0.20833	9.8		9.4		97		0		7		5				97.8						NA
-80.42	43.65	ELORA RC	6142286	43728.3	2019	9	20	0.25	9.9		9.4		97		0		7		6				97.83						NA
-80.42	43.65	ELORA RC	6142286	43728.3	2019	9	20	0.29167	12.5		11.9		97		0		9		6				97.83						NA
-80.42	43.65	ELORA RC	6142286	43728.3	2019	9	20	0.33333	15		12.8		86		0		11		5				97.85						NA
-80.42	43.65	ELORA RC	6142286	43728.4	2019	9	20	0.375	16.9		14.2		84		0		16		5				97.87						NA
-80.42	43.65	ELORA RC	6142286	43728.4	2019	9	20	0.41667	18.9		14.5		75		0		15		6				97.85						NA
-80.42	43.65	ELORA RC	6142286	43728.5	2019	9	20	0.45833	21.7		15.6		69		0		17		7				97.82		26				NA
-80.42	43.65	ELORA RC	6142286	43728.5	2019	9	20	0.5	23.4		16		63		0		16		6				97.78		28				NA
-80.42	43.65	ELORA RC	6142286	43728.5	2019	9	20	0.54167	24.3		15.4		57		0		18		12				97.74		29				NA
-80.42	43.65	ELORA RC	6142286	43728.6	2019	9	20	0.58333	24.8		16.8		61		0		19		12				97.7		30				NA
-80.42	43.65	ELORA RC	6142286	43728.6	2019	9	20	0.625	25.2		16.3		58		0		19		11				97.65		30				NA
-80.42	43.65	ELORA RC	6142286	43728.7	2019	9	20	0.66667	25.4		16.4		57		0		20		12				97.65		30				NA
-80.42	43.65	ELORA RC	6142286	43728.7	2019	9	20	0.70833	24.5		16.6		61		0		23		14				97.68		30				NA
-80.42	43.65	ELORA RC	6142286	43728.8	2019	9	20	0.75	22.7		16.3		67		0		20		11				97.71		28				NA
-80.42	43.65	ELORA RC	6142286	43728.8	2019	9	20	0.79167	20.4		15.7		74		0		19		6				97.74		25				NA
-80.42	43.65	ELORA RC	6142286	43728.8	2019	9	20	0.83333	19.6		15.6		78		0		14		3				97.79						NA
-80.42	43.65	ELORA RC	6142286	43728.9	2019	9	20	0.875	18.7		15.1		80		0				0				97.78						NA
-80.42	43.65	ELORA RC	6142286	43728.9	2019	9	20	0.91667	17.8		14.3		80		0		13		5				97.78						NA
-80.42	43.65	ELORA RC	6142286	43729	2019	9	20	0.95833	16.5		14		85		0				0				97.77						NA
-80.42	43.65	ELORA RC	6142286	43729	2019	9	21	0	15.4		13.1		86		0		12		5				97.77						NA
-80.42	43.65	ELORA RC	6142286	43729	2019	9	21	0.04167	14.9		13.4		91		0		9		6				97.78						NA
-80.42	43.65	ELORA RC	6142286	43729.1	2019	9	21	0.08333	14.2		13.2		93		0		6		4				97.79						NA
-80.42	43.65	ELORA RC	6142286	43729.1	2019	9	21	0.125	14.5		13.6		94		0		8		5				97.76						NA
-80.42	43.65	ELORA RC	6142286	43729.2	2019	9	21	0.16667	14.1		13.5		96		0		7		6				97.77						NA
-80.42	43.65	ELORA RC	6142286	43729.2	2019	9	21	0.20833	13.4		12.6		95		0		5		4				97.78						NA
-80.42	43.65	ELORA RC	6142286	43729.3	2019	9	21	0.25	13.4		12.2		92		0				0				97.83						NA
-80.42	43.65	ELORA RC	6142286	43729.3	2019	9	21	0.29167	15		13.6		91		0		8		6				97.85						NA
-80.42	43.65	ELORA RC	6142286	43729.3	2019	9	21	0.33333	18		15.3		84		0		13		3				97.86						NA
-80.42	43.65	ELORA RC	6142286	43729.4	2019	9	21	0.375	20.4		16.2		77		0		18		8				97.84		25				NA
-80.42	43.65	ELORA RC	6142286	43729.4	2019	9	21	0.41667	22.6		16.7		69		0		23		9				97.82		28				NA
-80.42	43.65	ELORA RC	6142286	43729.5	2019	9	21	0.45833	24.1		17.3		66		0		18		7				97.78		30				NA
-80.42	43.65	ELORA RC	6142286	43729.5	2019	9	21	0.5	25.7		18.1		63		0		25		12				97.73		32				NA
-80.42	43.65	ELORA RC	6142286	43729.5	2019	9	21	0.54167	25.8		17.8		61		0		25		9				97.66		32				NA
-80.42	43.65	ELORA RC	6142286	43729.6	2019	9	21	0.58333	26.9		17.9		58		0		22		15				97.63		33				NA
-80.42	43.65	ELORA RC	6142286	43729.6	2019	9	21	0.625	27.5		17.8		56		0		24		18				97.6		33				NA
-80.42	43.65	ELORA RC	6142286	43729.7	2019	9	21	0.66667	27.4		17.9		56		0		22		17				97.55		33				NA
-80.42	43.65	ELORA RC	6142286	43729.7	2019	9	21	0.70833	26.7		17.7		58		0		22		14				97.54		32				NA
-80.42	43.65	ELORA RC	6142286	43729.8	2019	9	21	0.75	24.2		18.7		71		0		21		12				97.53		31				NA
-80.42	43.65	ELORA RC	6142286	43729.8	2019	9	21	0.79167	21.8		18.2		80		0		19		10				97.55		28				NA
-80.42	43.65	ELORA RC	6142286	43729.8	2019	9	21	0.83333	20.9		18		83		0		19		10				97.58		27				NA
-80.42	43.65	ELORA RC	6142286	43729.9	2019	9	21	0.875	20.1		17.7		86		0		18		9				97.56		26				NA
-80.42	43.65	ELORA RC	6142286	43729.9	2019	9	21	0.91667	19.4		17.4		88		0		21		5				97.55						NA
-80.42	43.65	ELORA RC	6142286	43730	2019	9	21	0.95833	19.1		17.5		91		0		17		5				97.53						NA
-80.42	43.65	ELORA RC	6142286	43730	2019	9	22	0	18.1		17		94		0		22		6				97.51						NA
-80.42	43.65	ELORA RC	6142286	43730	2019	9	22	0.04167	19.1		18		93		0		22		6				97.48						NA
-80.42	43.65	ELORA RC	6142286	43730.1	2019	9	22	0.08333	18.7		17.7		94		0		19		8				97.44						NA
-80.42	43.65	ELORA RC	6142286	43730.1	2019	9	22	0.125	18		17.3		96		0		21		7				97.39						NA
-80.42	43.65	ELORA RC	6142286	43730.2	2019	9	22	0.16667	19.9		17.7		87		0		24		27				97.41						NA
-80.42	43.65	ELORA RC	6142286	43730.2	2019	9	22	0.20833	19.2		18		93		0.3		29		10				97.41						NA
-80.42	43.65	ELORA RC	6142286	43730.3	2019	9	22	0.25	19.2		18		93		0		24		11				97.45						NA
-80.42	43.65	ELORA RC	6142286	43730.3	2019	9	22	0.29167	19.1		17.8		92		0.4		22		7				97.37						NA
-80.42	43.65	ELORA RC	6142286	43730.3	2019	9	22	0.33333	20.9		18.2		85		0		23		13				97.34		27				NA
-80.42	43.65	ELORA RC	6142286	43730.4	2019	9	22	0.375	21.6		18		80		0		22		14				97.29		28				NA
-80.42	43.65	ELORA RC	6142286	43730.4	2019	9	22	0.41667	24.1		18.2		70		0		22		22				97.23		30				NA
-80.42	43.65	ELORA RC	6142286	43730.5	2019	9	22	0.45833	26.1		17.8		60		0		23		25				97.16		32				NA
-80.42	43.65	ELORA RC	6142286	43730.5	2019	9	22	0.5	26.7		16.4		53		0		23		32				97.09		32				NA
-80.42	43.65	ELORA RC	6142286	43730.5	2019	9	22	0.54167	27.5		16		50		0		24		33				97.01		32				NA
-80.42	43.65	ELORA RC	6142286	43730.6	2019	9	22	0.58333	27.7		15.3		47		0		24		31				96.94		32				NA
-80.42	43.65	ELORA RC	6142286	43730.6	2019	9	22	0.625	27.6		14.5		45		0		23		34				96.88		31				NA
-80.42	43.65	ELORA RC	6142286	43730.7	2019	9	22	0.66667	26.9		16		51		0		22		27				96.85		32				NA
-80.42	43.65	ELORA RC	6142286	43730.7	2019	9	22	0.70833	25.4		15		52		0		21		28				96.82		29				NA
-80.42	43.65	ELORA RC	6142286	43730.8	2019	9	22	0.75	24.4		15		56		0		21		23				96.81		28				NA
-80.42	43.65	ELORA RC	6142286	43730.8	2019	9	22	0.79167	22.9		14.9		61		0		21		16				96.8		27				NA
-80.42	43.65	ELORA RC	6142286	43730.8	2019	9	22	0.83333	23.4		14.7		58		0		22		21				96.78		27				NA
-80.42	43.65	ELORA RC	6142286	43730.9	2019	9	22	0.875	23.5		14.6		57		0		22		19				96.75		27				NA
-80.42	43.65	ELORA RC	6142286	43730.9	2019	9	22	0.91667	23.5		14.5		57		0		23		17				96.69		27				NA
-80.42	43.65	ELORA RC	6142286	43731	2019	9	22	0.95833	22.7		14.7		60		0		23		12				96.6		26				NA
-80.42	43.65	ELORA RC	6142286	43731	2019	9	23	0	22.5		14.9		62		0		21		14				96.57		26				NA
-80.42	43.65	ELORA RC	6142286	43731	2019	9	23	0.04167	19.7		16.5		82		0		28		13				96.56						NA
-80.42	43.65	ELORA RC	6142286	43731.1	2019	9	23	0.08333	20.7		16.3		76		0		19		15				96.42		26				NA
-80.42	43.65	ELORA RC	6142286	43731.1	2019	9	23	0.125	19.7		16.9		84		0.2		23		26				96.36						NA
-80.42	43.65	ELORA RC	6142286	43731.2	2019	9	23	0.16667	19.3		17		87		0.4		23		19				96.34						NA
-80.42	43.65	ELORA RC	6142286	43731.2	2019	9	23	0.20833	19.4		17.2		87		0		23		19				96.31						NA
-80.42	43.65	ELORA RC	6142286	43731.3	2019	9	23	0.25	19.8		17.6		87		0		24		21				96.36						NA
-80.42	43.65	ELORA RC	6142286	43731.3	2019	9	23	0.29167	19.8		17.9		89		0		25		23				96.38						NA
-80.42	43.65	ELORA RC	6142286	43731.3	2019	9	23	0.33333	19.4		17.3		88		0		26		24				96.43						NA
-80.42	43.65	ELORA RC	6142286	43731.4	2019	9	23	0.375	19.8		16.6		82		0		26		25				96.46						NA
-80.42	43.65	ELORA RC	6142286	43731.4	2019	9	23	0.41667	18.5		15.5		83		0		28		23				96.53						NA
-80.42	43.65	ELORA RC	6142286	43731.5	2019	9	23	0.45833																					
-80.42	43.65	ELORA RC	6142286	43731.5	2019	9	23	0.5	19.3		15.3		78		0		30		19				96.53						NA
-80.42	43.65	ELORA RC	6142286	43731.5	2019	9	23	0.54167	20.1		14.9		72		0		29		20				96.47						NA
-80.42	43.65	ELORA RC	6142286	43731.6	2019	9	23	0.58333	20.2		13.6		66		0		29		23				96.45						NA
-80.42	43.65	ELORA RC	6142286	43731.6	2019	9	23	0.625	18.6		15.1		80		0		28		21				96.49						NA
-80.42	43.65	ELORA RC	6142286	43731.7	2019	9	23	0.66667	19.3		11.6		61		0		28		25				96.5						NA
-80.42	43.65	ELORA RC	6142286	43731.7	2019	9	23	0.70833	17.6		11		65		0		29		23				96.53						NA
-80.42	43.65	ELORA RC	6142286	43731.8	2019	9	23	0.75	14.4		12.7		90		0		30		27				96.63						NA
-80.42	43.65	ELORA RC	6142286	43731.8	2019	9	23	0.79167	14.1		12.3		89		0.5		27		17				96.61						NA

-80.42	43.65	ELORA RC	6142286	43731.8	2019	9	23	0.83333	13.6		12		90		0		27		11				96.63						NA
-80.42	43.65	ELORA RC	6142286	43731.9	2019	9	23	0.875	13.6		12.9		96		0.3		26		11				96.64						NA
-80.42	43.65	ELORA RC	6142286	43731.9	2019	9	23	0.91667	13.4		12		91		0		29		16				96.67						NA
-80.42	43.65	ELORA RC	6142286	43732	2019	9	23	0.95833	13.3		12.4		94		0		29		11				96.69						NA
-80.42	43.65	ELORA RC	6142286	43732	2019	9	24	0	13.7		12.8		94		0		28		12				96.67						NA
-80.42	43.65	ELORA RC	6142286	43732	2019	9	24	0.04167	13.8		12.9		94		0.1		29		13				96.69						NA
-80.42	43.65	ELORA RC	6142286	43732.1	2019	9	24	0.08333	13.2		11.4		89		0		33		18				96.69						NA
-80.42	43.65	ELORA RC	6142286	43732.1	2019	9	24	0.125	11.9		10.3		90		0		33		13				96.75						NA
-80.42	43.65	ELORA RC	6142286	43732.2	2019	9	24	0.16667	11.2		10.3		94		0		30		13				96.81						NA
-80.42	43.65	ELORA RC	6142286	43732.2	2019	9	24	0.20833	11		9.9		93		0		33		8				96.84						NA
-80.42	43.65	ELORA RC	6142286	43732.3	2019	9	24	0.25	10.2		9.6		96		0		29		11				96.9						NA
-80.42	43.65	ELORA RC	6142286	43732.3	2019	9	24	0.29167	10.3		9.8		97		0		28		7				96.95						NA
-80.42	43.65	ELORA RC	6142286	43732.3	2019	9	24	0.33333	12.2		11.7		97		0		28		7				96.98						NA
-80.42	43.65	ELORA RC	6142286	43732.4	2019	9	24	0.375	14.2		12		87		0		30		14				97						NA
-80.42	43.65	ELORA RC	6142286	43732.4	2019	9	24	0.41667	16		11		72		0		30		18				96.98						NA
-80.42	43.65	ELORA RC	6142286	43732.5	2019	9	24	0.45833	15.7		8		60		0		30		19				96.98						NA
-80.42	43.65	ELORA RC	6142286	43732.5	2019	9	24	0.5	18.2		8.4		53		0		29		20				96.91						NA
-80.42	43.65	ELORA RC	6142286	43732.5	2019	9	24	0.54167	19		8.1		49		0		29		22				96.84						NA
-80.42	43.65	ELORA RC	6142286	43732.6	2019	9	24	0.58333	18.5		7.1		47		0		31		23				96.81						NA
-80.42	43.65	ELORA RC	6142286	43732.6	2019	9	24	0.625	18.8		7.8		49		0		31		21				96.79						NA
-80.42	43.65	ELORA RC	6142286	43732.7	2019	9	24	0.66667	18.8		7		46		0		30		17				96.81						NA
-80.42	43.65	ELORA RC	6142286	43732.7	2019	9	24	0.70833	18.4		8.1		51		0		28		15				96.83						NA
-80.42	43.65	ELORA RC	6142286	43732.8	2019	9	24	0.75	15.9		8.2		60		0		28		8				96.82						NA
-80.42	43.65	ELORA RC	6142286	43732.8	2019	9	24	0.79167	13.7		8.7		72		0		26		3				96.82						NA
-80.42	43.65	ELORA RC	6142286	43732.8	2019	9	24	0.83333	11.5		9.1		85		0		2		4				96.8						NA
-80.42	43.65	ELORA RC	6142286	43732.9	2019	9	24	0.875	9.6		8.1		90		0		9		3				96.78						NA
-80.42	43.65	ELORA RC	6142286	43732.9	2019	9	24	0.91667	9.2		8.4		95		0		7		3				96.73						NA
-80.42	43.65	ELORA RC	6142286	43733	2019	9	24	0.95833	8.9		8.2		96		0		9		6				96.63						NA
-80.42	43.65	ELORA RC	6142286	43733	2019	9	25	0	9.2		7.7		90		0		7		6				96.63						NA
-80.42	43.65	ELORA RC	6142286	43733	2019	9	25	0.04167	10.3		9.5		95		0				0				96.72						NA
-80.42	43.65	ELORA RC	6142286	43733.1	2019	9	25	0.08333	11.6		10.4		92		0		10		5				96.67						NA
-80.42	43.65	ELORA RC	6142286	43733.1	2019	9	25	0.125	11.8		11		94		0		9		3				96.65						NA
-80.42	43.65	ELORA RC	6142286	43733.2	2019	9	25	0.16667	11.3		10.6		95		0		11		4				96.61						NA
-80.42	43.65	ELORA RC	6142286	43733.2	2019	9	25	0.20833	10.7		10.2		97		0				0				96.6						NA
-80.42	43.65	ELORA RC	6142286	43733.3	2019	9	25	0.25	10.6		10.3		98		0		12		3				96.59						NA
-80.42	43.65	ELORA RC	6142286	43733.3	2019	9	25	0.29167	12.2		11.8		98		0		13		5				96.55						NA
-80.42	43.65	ELORA RC	6142286	43733.3	2019	9	25	0.33333	16.4		14.1		86		0		21		8				96.53						NA
-80.42	43.65	ELORA RC	6142286	43733.4	2019	9	25	0.375	18.8		14.2		75		0		23		20				96.5						NA
-80.42	43.65	ELORA RC	6142286	43733.4	2019	9	25	0.41667	20.8		13.8		64		0		21		26				96.43						NA
-80.42	43.65	ELORA RC	6142286	43733.5	2019	9	25	0.45833	22.5		13.9		58		0		21		27				96.35		26				NA
-80.42	43.65	ELORA RC	6142286	43733.5	2019	9	25	0.5	23.2		13.9		56		0		21		30				96.3		27				NA
-80.42	43.65	ELORA RC	6142286	43733.5	2019	9	25	0.54167	23.6		13.5		53		0		21		31				96.27		27				NA
-80.42	43.65	ELORA RC	6142286	43733.6	2019	9	25	0.58333	22.6		12		51		0		21		27				96.23		25				NA
-80.42	43.65	ELORA RC	6142286	43733.6	2019	9	25	0.625	21.9		11.1		50		0		21		36				96.18						NA
-80.42	43.65	ELORA RC	6142286	43733.7	2019	9	25	0.66667	20.7		11.6		56		0		21		34				96.16						NA
-80.42	43.65	ELORA RC	6142286	43733.7	2019	9	25	0.70833	20.8		11.7		56		0		22		20				96.17						NA
-80.42	43.65	ELORA RC	6142286	43733.8	2019	9	25	0.75	20.9		11.1		54		0		22		24				96.18						NA
-80.42	43.65	ELORA RC	6142286	43733.8	2019	9	25	0.79167	19.5		10.9		58		0		22		18				96.2						NA
-80.42	43.65	ELORA RC	6142286	43733.8	2019	9	25	0.83333	18.8		11.8		64		0		23		21				96.28						NA
-80.42	43.65	ELORA RC	6142286	43733.9	2019	9	25	0.875	18.1		12.2		68		0		23		18				96.27						NA
-80.42	43.65	ELORA RC	6142286	43733.9	2019	9	25	0.91667	17.1		12.5		75		0		24		16				96.29						NA
-80.42	43.65	ELORA RC	6142286	43734	2019	9	25	0.95833	16.8		13		78		0		24		13				96.27						NA
-80.42	43.65	ELORA RC	6142286	43734	2019	9	26	0	17.2		13.4		78		0		24		15				96.23						NA
-80.42	43.65	ELORA RC	6142286	43734	2019	9	26	0.04167	17.1		13.8		81		0		27		16				96.29						NA
-80.42	43.65	ELORA RC	6142286	43734.1	2019	9	26	0.08333	16		13.7		86		0		27		7				96.22						NA
-80.42	43.65	ELORA RC	6142286	43734.1	2019	9	26	0.125	15.8		14.1		89		0		28		5				96.21						NA
-80.42	43.65	ELORA RC	6142286	43734.2	2019	9	26	0.16667	14.6		12.9		90		0.4		28		5				96.22						NA
-80.42	43.65	ELORA RC	6142286	43734.2	2019	9	26	0.20833	13.6		12.3		92		0.1		25		6				96.25						NA
-80.42	43.65	ELORA RC	6142286	43734.3	2019	9	26	0.25	13		11.9		93		0.7		24		4				96.23						NA
-80.42	43.65	ELORA RC	6142286	43734.3	2019	9	26	0.29167	12.6		11.8		95		0		24		4				96.26						NA
-80.42	43.65	ELORA RC	6142286	43734.3	2019	9	26	0.33333	13.7		13		96		0		25		5				96.32						NA
-80.42	43.65	ELORA RC	6142286	43734.4	2019	9	26	0.375	15.1		13.2		88		0		26		12				96.35						NA
-80.42	43.65	ELORA RC	6142286	43734.4	2019	9	26	0.41667	17.1		11.5		70		0		28		23				96.37						NA
-80.42	43.65	ELORA RC	6142286	43734.5	2019	9	26	0.45833	18.1		9		55		0		26		27				96.36						NA
-80.42	43.65	ELORA RC	6142286	43734.5	2019	9	26	0.5	17.2		8.4		56		0		29		30				96.45						NA
-80.42	43.65	ELORA RC	6142286	43734.5	2019	9	26	0.54167	16.3		6.6		52		0		27		31				96.52						NA
-80.42	43.65	ELORA RC	6142286	43734.6	2019	9	26	0.58333	17		6.6		50		0		28		33				96.57						NA
-80.42	43.65	ELORA RC	6142286	43734.6	2019	9	26	0.625	17.1		6.8		51		0		28		28				96.64						NA
-80.42	43.65	ELORA RC	6142286	43734.7	2019	9	26	0.66667	15.7		6.4		54		0		29		25				96.73						NA
-80.42	43.65	ELORA RC	6142286	43734.7	2019	9	26	0.70833	15.5		6.1		53		0		29		20				96.83						NA
-80.42	43.65	ELORA RC	6142286	43734.8	2019	9	26	0.75	13.4		5.7		59		0		27		15				96.91						NA
-80.42	43.65	ELORA RC	6142286	43734.8	2019	9	26	0.79167	11.2		6.3		71		0		25		5				96.93						NA
-80.42	43.65	ELORA RC	6142286	43734.8	2019	9	26	0.83333	9.1		6.3		83		0				0				96.93						NA
-80.42	43.65	ELORA RC	6142286	43734.9	2019	9	26	0.875	7.8		6.2		90		0				0				97						NA
-80.42	43.65	ELORA RC	6142286	43734.9	2019	9	26	0.91667	6.7		5.9		94		0				0				97.05						NA
-80.42	43.65	ELORA RC	6142286	43735	2019	9	26	0.95833	6.6		5.9		95		0				0				97.08						NA
-80.42	43.65	ELORA RC	6142286	43735	2019	9	27	0	5.5		4.6		94		0		8		4				97.09						NA
-80.42	43.65	ELORA RC	6142286	43735	2019	9	27	0.04167	6		5.6		97		0		7		4				97.1						NA
-80.42	43.65	ELORA RC	6142286	43735.1	2019	9	27	0.08333	4.6		3.9		95		0		10		4				97.05						NA
-80.42	43.65	ELORA RC	6142286	43735.1	2019	9	27	0.125	4.2		3.5		95		0		7		4				97.07						NA
-80.42	43.65	ELORA RC	6142286	43735.2	2019	9	27	0.16667	4.6		4		96		0		7		3				97.13						NA
-80.42	43.65	ELORA RC	6142286	43735.2	2019	9	27	0.20833	5.5		5.2		98		0		1		5				97.2						NA
-80.42	43.65	ELORA RC	6142286	43735.3	2019	9	27	0.25	6		5.4		96		0		3		3				97.23						NA
-80.42	43.65	ELORA RC	6142286	43735.3	2019	9	27	0.29167	7.6		6.6		93		0		13		4				97.25						NA
-80.42	43.65	ELORA RC	6142286	43735.3	2019	9	27	0.33333	9.1		7.7		91		0		10		3				97.23						NA
-80.42	43.65	ELORA RC	6142286	43735.4	2019	9	27	0.375	12.1		9		81		0				0				97.23						NA
-80.42	43.65	ELORA RC	6142286	43735.4	2019	9	27	0.41667	16.6		8.3		58		0		20		15				97.21						NA
-80.42	43.65	ELORA RC	6142286	43735.5	2019	9	27	0.45833	19.1		6.9		45		0		20		22				97.15						NA
-80.42	43.65	ELORA RC	6142286	43735.5	2019	9	27	0.5	19.2		10		55		0		19		22				97.06						NA
-80.42	43.65	ELORA RC	6142286	43735.5	2019	9	27	0.54167	20.1		9.8		51		0		19		25				96.95						NA
-80.42	43.65	ELORA RC	6142286	43735.6	2019	9	27	0.58333	20.6		7.9		44		0		18		26				96.88						NA
-80.42	43.65	ELORA RC	6142286	43735.6	2019	9	27	0.625	20.1		7		43		0		19		23				96.82						NA
-80.42	43.65	ELORA RC	6142286	43735.7	2019	9	27	0.66667	20.1		6.9		42		0		18		21				96.76						NA
-80.42	43.65	ELORA RC	6142286	43735.7	2019	9	27	0.70833	19.3		7.5		46		0		17		16				96.78						NA
-80.42	43.65	ELORA RC	6142286	43735.8	2019	9	27	0.75	16.6		10.8		68		0.3		19		10				96.84						NA
-80.42	43.65	ELORA RC	6142286	43735.8	2019	9	27	0.79167	16.1		11.6		75		0.2		16		12				96.76						NA
-80.42	43.65	ELORA RC	6142286	43735.8	2019	9	27	0.83333	15.5		12.3		81		0		27		4				96.79						NA
-80.42	43.65	ELORA RC	6142286	43735.9	2019	9	27	0.875	15.5		12.4		82		0		8		5				96.73						NA
-80.42	43.65	ELORA RC	6142286	43735.9	2019	9	27	0.91667	13.7		12		90		0		8		5				96.67						NA
-80.42	43.65	ELORA RC	6142286	43736	2019	9	27	0.95833	15.4		11.8		79		0		15		9				96.64						NA
-80.42	43.65	ELORA RC	6142286	43736	2019	9	28	0	15.2		12.3		83		0		21		4				96.67						NA
-80.42	43.65	ELORA RC	6142286	43736	2019	9	28	0.04167	15.2		13.2		88		0.1		28		7				96.8						NA
-80.42	43.65	ELORA RC	6142286	43736.1	2019	9	28	0.08333	15.4		14.5		94		0.3		18		4				96.75						NA
-80.42	43.65	ELORA RC	6142286	43736.1	2019	9	28	0.125	16.5		15.4		93		0.1		21		10				96.77						NA
-80.42	43.65	ELORA RC	6142286	43736.2	2019	9	28	0.16667	17.1		16.1		94		1.4		21		16				96.75						NA
-80.42	43.65	ELORA RC	6142286	43736.2	2019	9	28	0.20833	16.8		15.8		93		0.6		23		13				96.77						NA
-80.42	43.65	ELORA RC	6142286	43736.3	2019	9	28	0.25	16.7		15.9		95		0		22		9				96.8						NA
-80.42	43.65	ELORA RC	6142286	43736.3	2019	9	28	0.29167	16.9		16.2		96		0		24		10				96.92						NA
-80.42	43.65	ELORA RC	6142286	43736.3	2019	9	28	0.33333	17.3		16.5		95		0		24		14				96.97						NA
-80.42	43.65	ELORA RC	6142286	43736.4	2019	9	28	0.375	17.6		16.7		95		0		23		16				97.05						NA
-80.42	43.65	ELORA RC	6142286	43736.4	2019	9	28	0.41667	17.7		17		96		0.5		24		14				97.09						NA
-80.42	43.65	ELORA RC	6142286	43736.5	2019	9	28	0.45833	18.1		17.3		96		0.5		25		14				97.16						NA
-80.42	43.65	ELORA RC	6142286	43736.5	2019	9	28	0.5	16.9		16.1		95		0		33		15				97.23						NA
-80.42	43.65	ELORA RC	6142286	43736.5	2019	9	28	0.54167	16.7		14.6		88		0.1		35		13				97.28						NA
-80.42	43.65	ELORA RC	6142286	43736.6	2019	9	28	0.58333	17.1		14.4		84		0		33		9				97.33						NA
-80.42	43.65	ELORA RC	6142286	43736.6	2019	9	28	0.625	17.4		14.4		82		0		32		11				97.47						NA
-80.42	43.65	ELORA RC	6142286	43736.7	2019	9	28	0.66667	17		13.4		79		0		32		14				97.52						NA
-80.42	43.65	ELORA RC	6142286	43736.7	2019	9	28	0.70833	16.6		13.8		84		0		32		9				97.62						NA
-80.42	43.65	ELORA RC	6142286	43736.8	2019	9	28	0.75	15.2		12.8		86		0		31		11				97.68						NA
-80.42	43.65	ELORA RC	6142286	43736.8	2019	9	28	0.79167	14.6		12.5		87		0		34		8				97.75						NA
-80.42	43.65	ELORA RC	6142286	43736.8	2019	9	28	0.83333	12.7		11.7		94		0		34		6				97.85						NA
-80.42	43.65	ELORA RC	6142286	43736.9	2019	9	28	0.875	12.7		11.3		91		0		1		9				97.93						NA
-80.42	43.65	ELORA RC	6142286	43736.9	2019	9	28	0.91667	11.6		10.4		93		0		3		10				97.98						NA
-80.42	43.65	ELORA RC	6142286	43737	2019	9	28	0.95833	10.5		9.5		94		0		3		6				98.02						NA
-80.42	43.65	ELORA RC	6142286	43737	2019	9	29	0	9.8		9.1		96		0		2		3				98.11						NA
-80.42	43.65	ELORA RC	6142286	43737	2019	9	29	0.04167	8.7		8.3		98		0		6		4				98.19						NA
-80.42	43.65	ELORA RC	6142286	43737.1	2019	9	29	0.08333	8.7		8		96		0		3		7				98.21						NA
-80.42	43.65	ELORA RC	6142286	43737.1	2019	9	29	0.125	8.3		7.6		95		0		4		11				98.2						NA
-80.42	43.65	ELORA RC	6142286	43737.2	2019	9	29	0.16667	7.7		6.5		92		0		4		12				98.23						NA
-80.42	43.65	ELORA RC	6142286	43737.2	2019	9	29	0.20833	7.4		6.3		93		0		5		15				98.23						NA
-80.42	43.65	ELORA RC	6142286	43737.3	2019	9	29	0.25	7		6.1		94		0		6		11				98.35						NA
-80.42	43.65	ELORA RC	6142286	43737.3	2019	9	29	0.29167	7.6		6.6		93		0		6		11				98.43						NA
-80.42	43.65	ELORA RC	6142286	43737.3	2019	9	29	0.33333	9.1		6.9		86		0		7		18				98.46						NA
-80.42	43.65	ELORA RC	6142286	43737.4	2019	9	29	0.375	10.2		7.4		83		0		9		19				98.47						NA
-80.42	43.65	ELORA RC	6142286	43737.4	2019	9	29	0.41667	11.3		6.2		71		0		9		23				98.53						NA
-80.42	43.65	ELORA RC	6142286	43737.5	2019	9	29	0.45833	12.5		5.6		63		0		8		21				98.5						NA
-80.42	43.65	ELORA RC	6142286	43737.5	2019	9	29	0.5	12.3		3.7		56		0		9		21				98.47						NA
-80.42	43.65	ELORA RC	6142286	43737.5	2019	9	29	0.54167	12.2		3.5		55		0		9		18				98.43						NA

-80.42	43.65	ELORA RC	6142286	43737.6	2019	9	29	0.58333	12.4		4.6		59		0		9		18				98.43						NA
-80.42	43.65	ELORA RC	6142286	43737.6	2019	9	29	0.625	12.4		4.9		60		0		12		14				98.38						NA
-80.42	43.65	ELORA RC	6142286	43737.7	2019	9	29	0.66667	11.7		5.5		66		0		10		16				98.33						NA
-80.42	43.65	ELORA RC	6142286	43737.7	2019	9	29	0.70833	11.3		5.8		69		0		9		15				98.27						NA
-80.42	43.65	ELORA RC	6142286	43737.8	2019	9	29	0.75	10.8		5		67		0		8		12				98.23						NA
-80.42	43.65	ELORA RC	6142286	43737.8	2019	9	29	0.79167	10.4		5		69		0		8		15				98.16						NA
-80.42	43.65	ELORA RC	6142286	43737.8	2019	9	29	0.83333	10.1		5.4		73		0		8		17				98.17						NA
-80.42	43.65	ELORA RC	6142286	43737.9	2019	9	29	0.875	10.3		5.9		74		0		8		13				98.2						NA
-80.42	43.65	ELORA RC	6142286	43737.9	2019	9	29	0.91667	10.3		6.3		76		0		9		15				98.17						NA
-80.42	43.65	ELORA RC	6142286	43738	2019	9	29	0.95833	10.5		6.6		77		0		8		14				98.19						NA
-80.42	43.65	ELORA RC	6142286	43738	2019	9	30	0	10.7		6.3		74		0		8		18				98.13						NA
-80.42	43.65	ELORA RC	6142286	43738	2019	9	30	0.04167	11.2		6.8		75		0		9		19				98.09						NA
-80.42	43.65	ELORA RC	6142286	43738.1	2019	9	30	0.08333	11.5		7.4		76		0		11		9				98.09						NA
-80.42	43.65	ELORA RC	6142286	43738.1	2019	9	30	0.125	11.1		7.6		79		0		8		17				98						NA
-80.42	43.65	ELORA RC	6142286	43738.2	2019	9	30	0.16667	11		8		82		0		8		17				97.96						NA
-80.42	43.65	ELORA RC	6142286	43738.2	2019	9	30	0.20833	11		8.1		83		0		9		16				97.9						NA
-80.42	43.65	ELORA RC	6142286	43738.3	2019	9	30	0.25	11.1		8.4		83		0		9		13				97.96						NA
-80.42	43.65	ELORA RC	6142286	43738.3	2019	9	30	0.29167	11.2		8.7		85		0		9		15				97.94						NA
-80.42	43.65	ELORA RC	6142286	43738.3	2019	9	30	0.33333	11.1		8.4		83		0		8		17				97.91						NA
-80.42	43.65	ELORA RC	6142286	43738.4	2019	9	30	0.375	11.9		8.5		80		0		9		14				97.88						NA
-80.42	43.65	ELORA RC	6142286	43738.4	2019	9	30	0.41667	13.2		9.2		76		0		11		14				97.88						NA
-80.42	43.65	ELORA RC	6142286	43738.5	2019	9	30	0.45833																					
-80.42	43.65	ELORA RC	6142286	43738.5	2019	9	30	0.5																					
-80.42	43.65	ELORA RC	6142286	43738.5	2019	9	30	0.54167	17.1		11.4		69		0		12		11				97.66						NA
-80.42	43.65	ELORA RC	6142286	43738.6	2019	9	30	0.58333	18.1		12.4		70		0		12		10				97.55						NA
-80.42	43.65	ELORA RC	6142286	43738.6	2019	9	30	0.625	18.6		13.5		72		0		13		13				97.49						NA
-80.42	43.65	ELORA RC	6142286	43738.7	2019	9	30	0.66667	18.1		13		72		0		14		14				97.42						NA
-80.42	43.65	ELORA RC	6142286	43738.7	2019	9	30	0.70833	16.8		12.9		78		0		16		15				97.46						NA
-80.42	43.65	ELORA RC	6142286	43738.8	2019	9	30	0.75	15.4		12.9		85		0		15		10				97.49						NA
-80.42	43.65	ELORA RC	6142286	43738.8	2019	9	30	0.79167	14.1		12.5		90		0		15		13				97.32						NA
-80.42	43.65	ELORA RC	6142286	43738.8	2019	9	30	0.83333	13.4		12.2		92		0		8		7				97.34						NA
-80.42	43.65	ELORA RC	6142286	43738.9	2019	9	30	0.875	12.9		12.1		94		0		13		5				97.3						NA
-80.42	43.65	ELORA RC	6142286	43738.9	2019	9	30	0.91667	13.5		12.5		94		0		16		7				97.26						NA
-80.42	43.65	ELORA RC	6142286	43739	2019	9	30	0.95833	12.7		12		95		0		15		6				97.21						NA

Table S2D. Hourly recorded weather data of the field trial in October 2019

Longitude	Latitude (	Station Na	Climate ID	Date/Tim	Year	Month	Day	Time (LST	Temp (°C)	Temp Flag	Dew Poin	Dew Poin	Rel Hum (	Rel Hum F	Precip. Ar	Precip. Ar	Wind Dir (	Wind Dir	Wind Spd	Wind Spd	Visibility (	Visibility F	Stn Press	Stn Press	Hmdx	Hmdx Flag	Wind Chil	Wind Chil	Weather
-80.42	43.65	ELORA RC	6142286	43739	2019	10	1	0	12.2		11.7		96		0		6		4				97.2						NA
-80.42	43.65	ELORA RC	6142286	43739	2019	10	1	0.04167	13.1		12.6		97		0				0				97.19						NA
-80.42	43.65	ELORA RC	6142286	43739.1	2019	10	1	0.08333	13		12.4		96		0				0				97.13						NA
-80.42	43.65	ELORA RC	6142286	43739.1	2019	10	1	0.125	13.3		12.7		96		0				0				97.1						NA
-80.42	43.65	ELORA RC	6142286	43739.2	2019	10	1	0.16667	13.4		12.8		96		0				0				97.05						NA
-80.42	43.65	ELORA RC	6142286	43739.2	2019	10	1	0.20833	13.6		13.1		97		0				0				97						NA
-80.42	43.65	ELORA RC	6142286	43739.3	2019	10	1	0.25	14.2		13.9		98		0				0				96.99						NA
-80.42	43.65	ELORA RC	6142286	43739.3	2019	10	1	0.29167	14.6		14.3		98		0				0				97.01						NA
-80.42	43.65	ELORA RC	6142286	43739.3	2019	10	1	0.33333	18.7		16.8		89		0		17		3				96.99						NA
-80.42	43.65	ELORA RC	6142286	43739.4	2019	10	1	0.375	22.2		18.5		80		0		22		12				96.96		29				NA
-80.42	43.65	ELORA RC	6142286	43739.4	2019	10	1	0.41667	25.5		19.2		68		0		23		17				96.93		32				NA
-80.42	43.65	ELORA RC	6142286	43739.5	2019	10	1	0.45833	26.2		19.6		67		0		24		24				96.87		33				NA
-80.42	43.65	ELORA RC	6142286	43739.5	2019	10	1	0.5	28.2		20.2		62		0		25		27				96.88		36				NA
-80.42	43.65	ELORA RC	6142286	43739.5	2019	10	1	0.54167	27.2		20.4		66		0		26		23				96.83		35				NA
-80.42	43.65	ELORA RC	6142286	43739.6	2019	10	1	0.58333	24.1		19.9		78		0		27		22				96.84		32				NA
-80.42	43.65	ELORA RC	6142286	43739.6	2019	10	1	0.625	20.2		19.8		97		13.7		25		19				96.95		28				NA
-80.42	43.65	ELORA RC	6142286	43739.7	2019	10	1	0.66667	20.2		19.6		97		13.5		24		17				96.93		27				NA
-80.42	43.65	ELORA RC	6142286	43739.7	2019	10	1	0.70833	19.8		19.3		97		0		26		16				96.95						NA
-80.42	43.65	ELORA RC	6142286	43739.8	2019	10	1	0.75	19.6		18.9		96		0		25		17				96.89						NA
-80.42	43.65	ELORA RC	6142286	43739.8	2019	10	1	0.79167	19.3		18.6		96		0		27		15				96.94						NA
-80.42	43.65	ELORA RC	6142286	43739.8	2019	10	1	0.83333	19		18.2		95		0		29		10				96.9						NA
-80.42	43.65	ELORA RC	6142286	43739.9	2019	10	1	0.875	17.9		17.3		96		0		25		11				96.87						NA
-80.42	43.65	ELORA RC	6142286	43739.9	2019	10	1	0.91667	17.3		16.9		98		0		26		7				96.87						NA
-80.42	43.65	ELORA RC	6142286	43740	2019	10	1	0.95833	17.9		17.6		98		0		25		8				96.81						NA
-80.42	43.65	ELORA RC	6142286	43740	2019	10	2	0	18		17.7		98		0		27		9				96.8						NA
-80.42	43.65	ELORA RC	6142286	43740	2019	10	2	0.04167	18.3		18		98		0		29		11				96.79						NA
-80.42	43.65	ELORA RC	6142286	43740.1	2019	10	2	0.08333	18.2		17.9		98		0.6		32		14				96.76						NA
-80.42	43.65	ELORA RC	6142286	43740.1	2019	10	2	0.125	16.5		16		97		7.4		7		6				96.66						NA
-80.42	43.65	ELORA RC	6142286	43740.2	2019	10	2	0.16667	15.7		15.1		97		0.1		28		6				96.77						NA
-80.42	43.65	ELORA RC	6142286	43740.2	2019	10	2	0.20833	15.6		14.9		95		0.1		1		4				96.75						NA
-80.42	43.65	ELORA RC	6142286	43740.3	2019	10	2	0.25	15.4		14.6		95		0.1		1		8				96.74						NA
-80.42	43.65	ELORA RC	6142286	43740.3	2019	10	2	0.29167	15.3		14.3		94		0.1		2		10				96.79						NA
-80.42	43.65	ELORA RC	6142286	43740.3	2019	10	2	0.33333	15.3		14.1		93		0.1		36		12				96.88						NA
-80.42	43.65	ELORA RC	6142286	43740.4	2019	10	2	0.375	15.2		14.3		94		1		2		10				96.91						NA
-80.42	43.65	ELORA RC	6142286	43740.4	2019	10	2	0.41667	14.4		13.5		94		0.1		2		20				96.95						NA
-80.42	43.65	ELORA RC	6142286	43740.5	2019	10	2	0.45833	14		12.6		91		1.2		3		12				97.01						NA
-80.42	43.65	ELORA RC	6142286	43740.5	2019	10	2	0.5	13.5		11.8		89		0		3		14				97.05						NA
-80.42	43.65	ELORA RC	6142286	43740.5	2019	10	2	0.54167	13.2		11.6		90		0		3		18				97.09						NA
-80.42	43.65	ELORA RC	6142286	43740.6	2019	10	2	0.58333	13		11.7		92		0		5		12				97.13						NA
-80.42	43.65	ELORA RC	6142286	43740.6	2019	10	2	0.625	12.6		10.9		89		0		3		13				97.13						NA
-80.42	43.65	ELORA RC	6142286	43740.7	2019	10	2	0.66667	12.5		10.2		86		0		4		18				97.17						NA
-80.42	43.65	ELORA RC	6142286	43740.7	2019	10	2	0.70833	11.5		9.7		89		0		4		15				97.24						NA
-80.42	43.65	ELORA RC	6142286	43740.8	2019	10	2	0.75	10.8		8.7		86		0		4		18				97.31						NA
-80.42	43.65	ELORA RC	6142286	43740.8	2019	10	2	0.79167	10.6		7.9		83		0		5		18				97.36						NA
-80.42	43.65	ELORA RC	6142286	43740.8	2019	10	2	0.83333	9.8		7.6		86		0		6		13				97.43						NA
-80.42	43.65	ELORA RC	6142286	43740.9	2019	10	2	0.875	9.5		7.1		85		0		7		17				97.46						NA
-80.42	43.65	ELORA RC	6142286	43740.9	2019	10	2	0.91667	8.8		6.2		83		0		7		22				97.42						NA
-80.42	43.65	ELORA RC	6142286	43741	2019	10	2	0.95833	8.4		5.9		84		0		7		22				97.46						NA
-80.42	43.65	ELORA RC	6142286	43741	2019	10	3	0	7.6		4.9		83		0		9		25				97.42						NA
-80.42	43.65	ELORA RC	6142286	43741	2019	10	3	0.04167	7.1		3.8		80		0		9		22				97.39						NA
-80.42	43.65	ELORA RC	6142286	43741.1	2019	10	3	0.08333	7.1		3.7		79		0		8		19				97.43						NA
-80.42	43.65	ELORA RC	6142286	43741.1	2019	10	3	0.125	6.9		3.2		77		0		8		20				97.35						NA
-80.42	43.65	ELORA RC	6142286	43741.2	2019	10	3	0.16667	6.9		3.4		79		0		7		16					M					NA
-80.42	43.65	ELORA RC	6142286	43741.2	2019	10	3	0.20833	6.5		4		84		0		9		17				97.36						NA
-80.42	43.65	ELORA RC	6142286	43741.3	2019	10	3	0.25	6.4		4.6		88		0.1		9		15				97.33						NA
-80.42	43.65	ELORA RC	6142286	43741.3	2019	10	3	0.29167	6.7		5.2		90		0		8		20					M					NA
-80.42	43.65	ELORA RC	6142286	43741.3	2019	10	3	0.33333	6.9		5.9		93		0.1		9		18				97.27						NA
-80.42	43.65	ELORA RC	6142286	43741.4	2019	10	3	0.375	7.5		6.8		95		0.4		8		20				97.18						NA
-80.42	43.65	ELORA RC	6142286	43741.4	2019	10	3	0.41667	7.8		6.9		95		0.2		9		21				97.13						NA
-80.42	43.65	ELORA RC	6142286	43741.5	2019	10	3	0.45833	8.1		7.4		95		0		9		16				97.07						NA
-80.42	43.65	ELORA RC	6142286	43741.5	2019	10	3	0.5	8.6		7.9		95		0		10		18				97.01						NA
-80.42	43.65	ELORA RC	6142286	43741.5	2019	10	3	0.54167	8.7		8		95		0		11		15				96.94						NA
-80.42	43.65	ELORA RC	6142286	43741.6	2019	10	3	0.58333	9.1		8.5		96		0		10		17				96.87						NA
-80.42	43.65	ELORA RC	6142286	43741.6	2019	10	3	0.625	9.3		8.7		96		0.2		11		9				96.88						NA
-80.42	43.65	ELORA RC	6142286	43741.7	2019	10	3	0.66667	9.8		9.2		96		0.1		14		7				96.86						NA
-80.42	43.65	ELORA RC	6142286	43741.7	2019	10	3	0.70833	10.2		9.7		97		0.1		20		3				96.89						NA
-80.42	43.65	ELORA RC	6142286	43741.8	2019	10	3	0.75	10.7		10.4		98		0.3		22		6				96.93						NA
-80.42	43.65	ELORA RC	6142286	43741.8	2019	10	3	0.79167	12		11.4		96		0.2		27		16				96.95						NA
-80.42	43.65	ELORA RC	6142286	43741.8	2019	10	3	0.83333	12.1		10.3		89		0.2		29		26				97.06						NA
-80.42	43.65	ELORA RC	6142286	43741.9	2019	10	3	0.875	11.8		10.5		92		0		29		17				97.1						NA
-80.42	43.65	ELORA RC	6142286	43741.9	2019	10	3	0.91667	11.8		10.7		93		0		28		14				97.13						NA
-80.42	43.65	ELORA RC	6142286	43742	2019	10	3	0.95833	11.8		10.6		92		0		29		15				97.16						NA
-80.42	43.65	ELORA RC	6142286	43742	2019	10	4	0	12		10.3		89		0		32		14				97.22						NA
-80.42	43.65	ELORA RC	6142286	43742	2019	10	4	0.04167	11.3		9.3		87		0		33		15				97.28						NA
-80.42	43.65	ELORA RC	6142286	43742.1	2019	10	4	0.08333	10		7.8		86		0		32		12				97.37						NA
-80.42	43.65	ELORA RC	6142286	43742.1	2019	10	4	0.125	9.3		7.3		87		0		34		12				97.48						NA
-80.42	43.65	ELORA RC	6142286	43742.2	2019	10	4	0.16667	8.7		7		89		0		2		14				97.58						NA
-80.42	43.65	ELORA RC	6142286	43742.2	2019	10	4	0.20833	7.7		5.7		87		0		36		13				97.7						NA
-80.42	43.65	ELORA RC	6142286	43742.3	2019	10	4	0.25	7.5		5.5		87		0		1		16				97.83						NA
-80.42	43.65	ELORA RC	6142286	43742.3	2019	10	4	0.29167	7.2		4.9		85		0		36		14				97.94						NA
-80.42	43.65	ELORA RC	6142286	43742.3	2019	10	4	0.33333	5.8		2.9		81		0		3		16				98.08						NA
-80.42	43.65	ELORA RC	6142286	43742.4	2019	10	4	0.375	7.1		3.2		76		0		5		20				98.17						NA
-80.42	43.65	ELORA RC	6142286	43742.4	2019	10	4	0.41667	6.4		2.6		77		0		4		18				98.24						NA
-80.42	43.65	ELORA RC	6142286	43742.5	2019	10	4	0.45833	6.6		2.1		73		0		3		15				98.32						NA
-80.42	43.65	ELORA RC	6142286	43742.5	2019	10	4	0.5	7.4		2.2		70		0		1		14				98.33						NA
-80.42	43.65	ELORA RC	6142286	43742.5	2019	10	4	0.54167	8.8		2.4		64		0		4		15				98.32						NA
-80.42	43.65	ELORA RC	6142286	43742.6	2019	10	4	0.58333	9.1		3.6		68		0		36		13				98.32						NA
-80.42	43.65	ELORA RC	6142286	43742.6	2019	10	4	0.625	9.8		2.8		62		0		4		10				98.32						NA
-80.42	43.65	ELORA RC	6142286	43742.7	2019	10	4	0.66667	9.6		2.6		61		0		2		10				98.31						NA
-80.42	43.65	ELORA RC	6142286	43742.7	2019	10	4	0.70833	9.4		2.9		64		0		2		8				98.33						NA
-80.42	43.65	ELORA RC	6142286	43742.8	2019	10	4	0.75	7.5		3.1		73		0		2		7				98.36						NA
-80.42	43.65	ELORA RC	6142286	43742.8	2019	10	4	0.79167	6.7		3.9		82		0		36		5				98.43						NA
-80.42	43.65	ELORA RC	6142286	43742.8	2019	10	4	0.83333	5.8		3.7		86		0		2		9				98.46						NA
-80.42	43.65	ELORA RC	6142286	43742.9	2019	10	4	0.875	4.6		2.4		86		0		3		10				98.51						NA
-80.42	43.65	ELORA RC	6142286	43742.9	2019	10	4	0.91667	3.6		1.6		87		0		1		8				98.52						NA
-80.42	43.65	ELORA RC	6142286	43743	2019	10	4	0.95833	3.8		1.7		86		0		2		13				98.5						NA
-80.42	43.65	ELORA RC	6142286	43743	2019	10	5	0	2.5		1.3		92		0		4		6				98.47						NA
-80.42	43.65	ELORA RC	6142286	43743	2019	10	5	0.04167	2.9		1.4		90		0		4		8				98.47						NA
-80.42	43.65	ELORA RC	6142286	43743.1	2019	10	5	0.08333	3.1		1.8		91		0		7		12				98.44						NA
-80.42	43.65	ELORA RC	6142286	43743.1	2019	10	5	0.125	2.4		1.1		91		0		7		6				98.44						NA
-80.42	43.65	ELORA RC	6142286	43743.2	2019	10	5	0.16667	4.5		2.6		87		0		7		11				98.43						NA
-80.42	43.65	ELORA RC	6142286	43743.2	2019	10	5	0.20833	4.1		2.7		90		0		8		11				98.38						NA
-80.42	43.65	ELORA RC	6142286	43743.3	2019	10	5	0.25	4.7		3.2		89		0		8		8				98.39						NA
-80.42	43.65	ELORA RC	6142286	43743.3	2019	10	5	0.29167	4.7		2.9		88		0		7		11				98.39						NA
-80.42	43.65	ELORA RC	6142286	43743.3	2019	10	5	0.33333	5.7		3.5		86		0		7		13				98.34						NA
-80.42	43.65	ELORA RC	6142286	43743.4	2019	10	5	0.375	7.9		4.4		79		0		9		23				98.34						NA
-80.42	43.65	ELORA RC	6142286	43743.4	2019	10	5	0.41667	8.7		4.2		74		0		10		23				98.31						NA
-80.42	43.65	ELORA RC	6142286	43743.5	2019	10	5	0.45833	10.3		4.6		67		0		10		24				98.25						NA
-80.42	43.65	ELORA RC	6142286	43743.5	2019	10	5	0.5	10.7		4.2		64		0		9		27				98.14						NA
-80.42	43.65	ELORA RC	6142286	43743.5	2019	10	5	0.54167	11.3		3.5		59		0		10		20				98						NA
-80.42	43.65	ELORA RC	6142286	43743.6	2019	10	5	0.58333	11.4		2		52		0		11		25				97.89						NA
-80.42	43.65	ELORA RC	6142286	43743.6	2019	10	5	0.625	10.9		3.3		59		0		10		24				97.8						NA
-80.42	43.65	ELORA RC	6142286	43743.7	2019	10	5	0.66667	9.6		3.5		65		0		10		21				97.75						NA
-80.42	43.65	ELORA RC	6142286	43743.7	2019	10	5	0.70833	9.1		3.6		68		0		10		20				97.65						NA
-80.42	43.65	ELORA RC	6142286	43743.8	2019	10	5	0.75	8.3		4.7		78		0		10		19				97.55						NA
-80.42	43.65	ELORA RC	6142286	43743.8	2019	10	5	0.79167	8.2		5.5		83		0		11		16				97.51						NA
-80.42	43.65	ELORA RC	6142286	43743.8	2019	10	5	0.83333	7.8		5.8		87		0		10		11				97.45						NA
-80.42	43.65	ELORA RC	6142286	43743.9	2019	10	5	0.875	7.6		6		89		0		9		9				97.41						NA
-80.42	43.65	ELORA RC	6142286	43743.9	2019	10	5	0.91667	8.3		6.4		88		0		12		7				97.32						NA
-80.42	43.65	ELORA RC	6142286	43744	2019	10	5	0.95833	8.7		6.8		88		0		12		8				97.28						NA
-80.42	43.65	ELORA RC	6142286	43744	2019	10	6	0	9.5		7.5		87		0		15		12				97.17						NA
-80.42	43.65	ELORA RC	6142286	43744	2019	10	6	0.04167	10.6		8.2		85		0		16		16				97.08						NA
-80.42	43.65	ELORA RC	6142286	43744.1	2019	10	6	0.08333	10.9		8.5		85		0		16		16				97.03						NA
-80.42	43.65	ELORA RC	6142286	43744.1	2019	10	6	0.125	10.7		9.3		91		0.2		17		11				97.04						NA
-80.42	43.65	ELORA RC	6142286	43744.2	2019	10	6	0.16667	10.7		9.6		93		0		17		5				97.06						NA
-80.42	43.65	ELORA RC	6142286	43744.2	2019	10	6	0.20833	10.8		9.9		94		0		19		6				97.12						NA
-80.42	43.65	ELORA RC	6142286	43744.3	2019	10	6	0.25	11.1		10.7		97		1.1		17		6				97.13						NA
-80.42	43.65	ELORA RC	6142286	43744.3	2019	10	6	0.29167	12.1		11.7		97		0		15		10				97.08						NA
-80.42	43.65	ELORA RC	6142286	43744.3	2019	10	6	0.33333	13.2		12.8		97		0.1		18		12				97.09						NA
-80.42	43.65	ELORA RC	6142286	43744.4	2019	10	6	0.375	14.2		13.5		96		0.1		19		17				97.11						NA
-80.42	43.65	ELORA RC	6142286	43744.4	2019	10	6	0.41667	16		14.3		90		0.1		20		21				97.07						NA
-80.42	43.65	ELORA RC	6142286	43744.5	2019	10	6	0.45833	17.8		13.9		78		0		22		30				97.05						NA
-80.42	43.65	ELORA RC	6142286	43744.5	2019	10	6	0.5	18.5		14.8		79		0		21		24				97.03						NA
-80.42	43.65	ELORA RC	6142286	43744.5	2019	10	6	0.54167	17.5		14.2		81		0		21		28				97.01						NA

-80.42	43.65	ELORA RC	6142286	43744.6	2019	10	6	0.58333	17.4		13		75		0		25		25				97.07						NA
-80.42	43.65	ELORA RC	6142286	43744.6	2019	10	6	0.625	18.3		9.5		57		0		25		35				97.12						NA
-80.42	43.65	ELORA RC	6142286	43744.7	2019	10	6	0.66667	17.3		9.3		59		0		26		26				97.16						NA
-80.42	43.65	ELORA RC	6142286	43744.7	2019	10	6	0.70833	16.6		8.7		60		0		27		23				97.22						NA
-80.42	43.65	ELORA RC	6142286	43744.8	2019	10	6	0.75	13.7		7.9		68		0		26		15				97.27						NA
-80.42	43.65	ELORA RC	6142286	43744.8	2019	10	6	0.79167	10.7		7		78		0		26		9				97.35						NA
-80.42	43.65	ELORA RC	6142286	43744.8	2019	10	6	0.83333	10.4		7.5		82		0		25		5				97.4						NA
-80.42	43.65	ELORA RC	6142286	43744.9	2019	10	6	0.875	10		7.5		84		0		27		4				97.4						NA
-80.42	43.65	ELORA RC	6142286	43744.9	2019	10	6	0.91667	7.4		6.4		93		0				0				97.37						NA
-80.42	43.65	ELORA RC	6142286	43745	2019	10	6	0.95833	7.3		6.5		95		0		18		4				97.4						NA
-80.42	43.65	ELORA RC	6142286	43745	2019	10	7	0	9.2		7.1		87		0		31		13				97.31						NA
-80.42	43.65	ELORA RC	6142286	43745	2019	10	7	0.04167	9.6		6.3		80		0		23		11				97.37						NA
-80.42	43.65	ELORA RC	6142286	43745.1	2019	10	7	0.08333	9.1		6.8		85		0		19		7				97.29						NA
-80.42	43.65	ELORA RC	6142286	43745.1	2019	10	7	0.125	8.8		7.1		89		0		19		6				97.27						NA
-80.42	43.65	ELORA RC	6142286	43745.2	2019	10	7	0.16667	9.1		7.2		88		0		18		7				97.29						NA
-80.42	43.65	ELORA RC	6142286	43745.2	2019	10	7	0.20833	9.3		7.3		87		0		18		10				97.33						NA
-80.42	43.65	ELORA RC	6142286	43745.3	2019	10	7	0.25	9		7.2		89		0		18		7				97.36						NA
-80.42	43.65	ELORA RC	6142286	43745.3	2019	10	7	0.29167	8.8		7.1		89		0		20		6				97.38						NA
-80.42	43.65	ELORA RC	6142286	43745.3	2019	10	7	0.33333	9.4		7.6		89		0		23		7				97.42						NA
-80.42	43.65	ELORA RC	6142286	43745.4	2019	10	7	0.375	11.3		8.4		83		0		23		13				97.39						NA
-80.42	43.65	ELORA RC	6142286	43745.4	2019	10	7	0.41667	13.6		9		74		0		24		18				97.42						NA
-80.42	43.65	ELORA RC	6142286	43745.5	2019	10	7	0.45833	13.9		8.6		70		0		25		18				97.42						NA
-80.42	43.65	ELORA RC	6142286	43745.5	2019	10	7	0.5	14.8		7.7		63		0		26		26				97.43						NA
-80.42	43.65	ELORA RC	6142286	43745.5	2019	10	7	0.54167	15.3		6.1		54		0		26		24				97.41						NA
-80.42	43.65	ELORA RC	6142286	43745.6	2019	10	7	0.58333	16		2.7		41		0		26		24				97.41						NA
-80.42	43.65	ELORA RC	6142286	43745.6	2019	10	7	0.625	16.5		2.7		39		0		27		22				97.4						NA
-80.42	43.65	ELORA RC	6142286	43745.7	2019	10	7	0.66667	15.7		2.7		42		0		27		23				97.48						NA
-80.42	43.65	ELORA RC	6142286	43745.7	2019	10	7	0.70833	14.7		2.4		43		0		28		21				97.56						NA
-80.42	43.65	ELORA RC	6142286	43745.8	2019	10	7	0.75	11.8		3.7		58		0		29		10				97.58						NA
-80.42	43.65	ELORA RC	6142286	43745.8	2019	10	7	0.79167	9.2		3.3		67		0		27		3				97.63						NA
-80.42	43.65	ELORA RC	6142286	43745.8	2019	10	7	0.83333	7.8		4		77		0				0				97.68						NA
-80.42	43.65	ELORA RC	6142286	43745.9	2019	10	7	0.875	6.2		3.8		85		0				0				97.75						NA
-80.42	43.65	ELORA RC	6142286	43745.9	2019	10	7	0.91667	5.4		3.7		89		0		36		3				97.8						NA
-80.42	43.65	ELORA RC	6142286	43746	2019	10	7	0.95833	5.2		3.9		91		0		36		4				97.84						NA
-80.42	43.65	ELORA RC	6142286	43746	2019	10	8	0	3.9		2.2		88		0		3		3				97.88						NA
-80.42	43.65	ELORA RC	6142286	43746	2019	10	8	0.04167	3.1		2		92		0		12		3				97.92						NA
-80.42	43.65	ELORA RC	6142286	43746.1	2019	10	8	0.08333	3.3		2.6		95		0		4		3				97.95						NA
-80.42	43.65	ELORA RC	6142286	43746.1	2019	10	8	0.125	3.2		2.6		96		0		2		3				97.96						NA
-80.42	43.65	ELORA RC	6142286	43746.2	2019	10	8	0.16667	3.8		3.1		95		0		2		5				98						NA
-80.42	43.65	ELORA RC	6142286	43746.2	2019	10	8	0.20833	4.2		3.5		95		0		4		7				98.04						NA
-80.42	43.65	ELORA RC	6142286	43746.3	2019	10	8	0.25	4.7		3.3		91		0		4		6				98.11						NA
-80.42	43.65	ELORA RC	6142286	43746.3	2019	10	8	0.29167	4.9		4		94		0		5		7				98.13						NA
-80.42	43.65	ELORA RC	6142286	43746.3	2019	10	8	0.33333	8.9		6.3		84		0		12		7				98.18						NA
-80.42	43.65	ELORA RC	6142286	43746.4	2019	10	8	0.375	11.4		6.8		73		0		9		12				98.21						NA
-80.42	43.65	ELORA RC	6142286	43746.4	2019	10	8	0.41667	13.1		6.7		65		0		9		14				98.2						NA
-80.42	43.65	ELORA RC	6142286	43746.5	2019	10	8	0.45833	14.3		6.8		60		0		12		12				98.21						NA
-80.42	43.65	ELORA RC	6142286	43746.5	2019	10	8	0.5	15.4		7.2		58		0		11		10				98.2						NA
-80.42	43.65	ELORA RC	6142286	43746.5	2019	10	8	0.54167	15.8		6		52		0		7		3				98.12						NA
-80.42	43.65	ELORA RC	6142286	43746.6	2019	10	8	0.58333	16.1		5.4		49		0		11		5				98.09						NA
-80.42	43.65	ELORA RC	6142286	43746.6	2019	10	8	0.625	16.5		5		46		0		15		5				98.07						NA
-80.42	43.65	ELORA RC	6142286	43746.7	2019	10	8	0.66667	16.6		6.1		50		0		14		5				98.07						NA
-80.42	43.65	ELORA RC	6142286	43746.7	2019	10	8	0.70833	15.3		6.5		56		0		11		10				98.07						NA
-80.42	43.65	ELORA RC	6142286	43746.8	2019	10	8	0.75	12		6.2		68		0		12		8				98.07						NA
-80.42	43.65	ELORA RC	6142286	43746.8	2019	10	8	0.79167	9.5		6.4		81		0		10		7				98.08						NA
-80.42	43.65	ELORA RC	6142286	43746.8	2019	10	8	0.83333	8.1		5.9		86		0		9		8				98.1						NA
-80.42	43.65	ELORA RC	6142286	43746.9	2019	10	8	0.875	7.1		5.6		90		0		7		6				98.14						NA
-80.42	43.65	ELORA RC	6142286	43746.9	2019	10	8	0.91667	6.6		5		90		0		7		4				98.14						NA
-80.42	43.65	ELORA RC	6142286	43747	2019	10	8	0.95833	6.5		5.9		96		0		9		10				98.13						NA
-80.42	43.65	ELORA RC	6142286	43747	2019	10	9	0	7.6		6.8		95		0		9		12				98.09						NA
-80.42	43.65	ELORA RC	6142286	43747	2019	10	9	0.04167	7.2		6.1		93		0		9		12				98.09						NA
-80.42	43.65	ELORA RC	6142286	43747.1	2019	10	9	0.08333	7		5.8		92		0		9		13				98.08						NA
-80.42	43.65	ELORA RC	6142286	43747.1	2019	10	9	0.125	6.1		4.8		92		0		9		10				98.09						NA
-80.42	43.65	ELORA RC	6142286	43747.2	2019	10	9	0.16667	6.2		4.5		89		0		9		11				98.13						NA
-80.42	43.65	ELORA RC	6142286	43747.2	2019	10	9	0.20833	4.7		3.6		93		0		7		8				98.18						NA
-80.42	43.65	ELORA RC	6142286	43747.3	2019	10	9	0.25	4.5		3.3		92		0		8		8				98.21						NA
-80.42	43.65	ELORA RC	6142286	43747.3	2019	10	9	0.29167	5.5		4.8		95		0		7		9				98.23						NA
-80.42	43.65	ELORA RC	6142286	43747.3	2019	10	9	0.33333	9.1		6.9		86		0		6		6				98.28						NA
-80.42	43.65	ELORA RC	6142286	43747.4	2019	10	9	0.375	11.5		8.6		82		0		10		13				98.27						NA
-80.42	43.65	ELORA RC	6142286	43747.4	2019	10	9	0.41667	13.4		7.7		69		0		10		16				98.27						NA
-80.42	43.65	ELORA RC	6142286	43747.5	2019	10	9	0.45833	14.5		7.7		64		0		9		17				98.23						NA
-80.42	43.65	ELORA RC	6142286	43747.5	2019	10	9	0.5	15.1		7.3		60		0		10		13				98.18						NA
-80.42	43.65	ELORA RC	6142286	43747.5	2019	10	9	0.54167	16.3		6.3		52		0		10		15				98.12						NA
-80.42	43.65	ELORA RC	6142286	43747.6	2019	10	9	0.58333	16.6		5.2		47		0		10		15				98.08						NA
-80.42	43.65	ELORA RC	6142286	43747.6	2019	10	9	0.625	16.3		5.3		48		0		13		15				98.06						NA
-80.42	43.65	ELORA RC	6142286	43747.7	2019	10	9	0.66667	15.4		5.4		51		0		11		16				98.04						NA
-80.42	43.65	ELORA RC	6142286	43747.7	2019	10	9	0.70833	13.6		5.2		57		0		9		17				98.06						NA
-80.42	43.65	ELORA RC	6142286	43747.8	2019	10	9	0.75	10.8		2.9		58		0		9		14				98.05						NA
-80.42	43.65	ELORA RC	6142286	43747.8	2019	10	9	0.79167	8.5		2.5		66		0		9		11				98.08						NA
-80.42	43.65	ELORA RC	6142286	43747.8	2019	10	9	0.83333	6.2		3.2		81		0		7		8				98.09						NA
-80.42	43.65	ELORA RC	6142286	43747.9	2019	10	9	0.875	6.1		3.9		86		0		8		6				98.11						NA
-80.42	43.65	ELORA RC	6142286	43747.9	2019	10	9	0.91667	6.4		3.9		84		0		6		4				98.15						NA
-80.42	43.65	ELORA RC	6142286	43748	2019	10	9	0.95833	6.5		5.3		92		0		8		10				98.12						NA
-80.42	43.65	ELORA RC	6142286	43748	2019	10	10	0	7.3		6.1		92		0		9		11				98.09						NA
-80.42	43.65	ELORA RC	6142286	43748	2019	10	10	0.04167	7		6.2		95		0		9		11				98.07						NA
-80.42	43.65	ELORA RC	6142286	43748.1	2019	10	10	0.08333	7		6.1		94		0		9		12				98.05						NA
-80.42	43.65	ELORA RC	6142286	43748.1	2019	10	10	0.125	6.3		5.4		94		0		7		7				98.06						NA
-80.42	43.65	ELORA RC	6142286	43748.2	2019	10	10	0.16667	5.5		4.9		97		0		6		8				98.06						NA
-80.42	43.65	ELORA RC	6142286	43748.2	2019	10	10	0.20833	7		6.4		96		0		7		12				98.09						NA
-80.42	43.65	ELORA RC	6142286	43748.3	2019	10	10	0.25	6.7		6.1		96		0		8		11				98.14						NA
-80.42	43.65	ELORA RC	6142286	43748.3	2019	10	10	0.29167	7.1		6.5		96		0		8		11				98.15						NA
-80.42	43.65	ELORA RC	6142286	43748.3	2019	10	10	0.33333	8.2		7.6		96		0		8		15				98.19						NA
-80.42	43.65	ELORA RC	6142286	43748.4	2019	10	10	0.375	10.6		9.3		92		0		10		16				98.19						NA
-80.42	43.65	ELORA RC	6142286	43748.4	2019	10	10	0.41667	12.5		8.8		78		0		10		17				98.17						NA
-80.42	43.65	ELORA RC	6142286	43748.5	2019	10	10	0.45833	14.6		7.7		63		0		13		16				98.13						NA
-80.42	43.65	ELORA RC	6142286	43748.5	2019	10	10	0.5	15.7		6		52		0		11		15				98.1						NA
-80.42	43.65	ELORA RC	6142286	43748.5	2019	10	10	0.54167	15.9		6.9		55		0		11		16				98.06						NA
-80.42	43.65	ELORA RC	6142286	43748.6	2019	10	10	0.58333	16.1		5.1		48		0		11		21				98.01						NA
-80.42	43.65	ELORA RC	6142286	43748.6	2019	10	10	0.625	15.9		5		49		0		9		22				97.96						NA
-80.42	43.65	ELORA RC	6142286	43748.7	2019	10	10	0.66667	14.9		5.5		53		0		9		21				97.97						NA
-80.42	43.65	ELORA RC	6142286	43748.7	2019	10	10	0.70833	13.3		4.8		56		0		11		18				97.98						NA
-80.42	43.65	ELORA RC	6142286	43748.8	2019	10	10	0.75	10.8		5.1		68		0		10		13				97.94						NA
-80.42	43.65	ELORA RC	6142286	43748.8	2019	10	10	0.79167	9.2		4.9		75		0		8		13				97.91						NA
-80.42	43.65	ELORA RC	6142286	43748.8	2019	10	10	0.83333	9		5.5		79		0		9		15				97.9						NA
-80.42	43.65	ELORA RC	6142286	43748.9	2019	10	10	0.875	8.2		5.8		85		0		9		12				97.94						NA
-80.42	43.65	ELORA RC	6142286	43748.9	2019	10	10	0.91667	8.2		6.6		89		0		9		14				97.9						NA
-80.42	43.65	ELORA RC	6142286	43749	2019	10	10	0.95833	7.8		6.9		94		0		9		11				97.9						NA
-80.42	43.65	ELORA RC	6142286	43749	2019	10	11	0	8.8		7.4		91		0		9		15				97.84						NA
-80.42	43.65	ELORA RC	6142286	43749	2019	10	11	0.04167	9.2		7.3		88		0		9		17				97.8						NA
-80.42	43.65	ELORA RC	6142286	43749.1	2019	10	11	0.08333	9.3		7.5		88		0		9		16				97.79						NA
-80.42	43.65	ELORA RC	6142286	43749.1	2019	10	11	0.125	9.7		8.5		92		0		9		17				97.78						NA
-80.42	43.65	ELORA RC	6142286	43749.2	2019	10	11	0.16667	9.6		8.6		94		0		10		15				97.77						NA
-80.42	43.65	ELORA RC	6142286	43749.2	2019	10	11	0.20833	9.5		8.6		94		0		9		15				97.75						NA
-80.42	43.65	ELORA RC	6142286	43749.3	2019	10	11	0.25	10		9.6		98		0		9		16				97.76						NA
-80.42	43.65	ELORA RC	6142286	43749.3	2019	10	11	0.29167	10.2		9.9		98		0		9		15				97.76						NA
-80.42	43.65	ELORA RC	6142286	43749.3	2019	10	11	0.33333	11.1		10.4		95		0		10		19				97.79						NA
-80.42	43.65	ELORA RC	6142286	43749.4	2019	10	11	0.375	12.8		10.7		87		0		10		17				97.77						NA
-80.42	43.65	ELORA RC	6142286	43749.4	2019	10	11	0.41667	14.9		10.8		76		0		9		20				97.7						NA
-80.42	43.65	ELORA RC	6142286	43749.5	2019	10	11	0.45833	17.1		9.8		62		0		12		19				97.66						NA
-80.42	43.65	ELORA RC	6142286	43749.5	2019	10	11	0.5	18.4		9.3		55		0		13		17				97.58						NA
-80.42	43.65	ELORA RC	6142286	43749.5	2019	10	11	0.54167	19.1		8.8		51		0		13		17				97.53						NA
-80.42	43.65	ELORA RC	6142286	43749.6	2019	10	11	0.58333	19.6		8.6		49		0		14		19				97.44						NA
-80.42	43.65	ELORA RC	6142286	43749.6	2019	10	11	0.625	19.3		8.5		50		0		12		19				97.36						NA
-80.42	43.65	ELORA RC	6142286	43749.7	2019	10	11	0.66667	17.4		7.1		51		0		11		23				97.3						NA
-80.42	43.65	ELORA RC	6142286	43749.7	2019	10	11	0.70833	15.4		5.2		51		0		11		18				97.27						NA
-80.42	43.65	ELORA RC	6142286	43749.8	2019	10	11	0.75	13.9		4.3		52		0		10		15				97.23						NA
-80.42	43.65	ELORA RC	6142286	43749.8	2019	10	11	0.79167	12.5		5.8		64		0		10		17				97.2						NA
-80.42	43.65	ELORA RC	6142286	43749.8	2019	10	11	0.83333	12.3		7.5		72		0		10		22				97.08						NA
-80.42	43.65	ELORA RC	6142286	43749.9	2019	10	11	0.875	12.8		8.1		73		0		10		18				97.08						NA
-80.42	43.65	ELORA RC	6142286	43749.9	2019	10	11	0.91667	13.6		8.1		69		0		11		20				97.01						NA
-80.42	43.65	ELORA RC	6142286	43750	2019	10	11	0.95833	14.5		7.4		63		0		11		19				96.92						NA
-80.42	43.65	ELORA RC	6142286	43750	2019	10	12	0	13.7		8		68		0		11		16				96.82						NA
-80.42	43.65	ELORA RC	6142286	43750	2019	10	12	0.04167	12.5		9		79		0		16		8				96.86						NA
-80.42	43.65	ELORA RC	6142286	43750.1	2019	10	12	0.08333	11.3		9.6		89		0.7		13		14				96.74						NA
-80.42	43.65	ELORA RC	6142286	43750.1	2019	10	12	0.125	11.1		10.2		94		0.5		14		16				96.62						NA
-80.42	43.65	ELORA RC	6142286	43750.2	2019	10	12	0.16667	11		10.5		97		0.4		25		8				96.65						NA
-80.42	43.65	ELORA RC	6142286	43750.2	2019	10	12	0.20833	9.8		9.1		95		0.5		28		17				96.7						NA
-80.42	43.65	ELORA RC	6142286	43750.3	2019	10	12	0.25	8.9		8.1		95		1.2		28		21				96.74						NA
-80.42	43.65	ELORA RC	6142286	43750.3	2019	10	12	0.29167	7.6		6.7		94		0.2		28		22				96.79						NA

-80.42	43.65	ELORA RC	6142286	43750.3	2019	10	12	0.33333	7		6.1		94		0		29		22				96.9						NA
-80.42	43.65	ELORA RC	6142286	43750.4	2019	10	12	0.375	6.4		5		91		0		27		23				96.96						NA
-80.42	43.65	ELORA RC	6142286	43750.4	2019	10	12	0.41667	7.2		2.6		72		0		27		25				97						NA
-80.42	43.65	ELORA RC	6142286	43750.5	2019	10	12	0.45833	8.1		3.1		71		0		27		26				97.02						NA
-80.42	43.65	ELORA RC	6142286	43750.5	2019	10	12	0.5	8.2		1.8		64		0		26		25				97.04						NA
-80.42	43.65	ELORA RC	6142286	43750.5	2019	10	12	0.54167	8.4		1.3		61		0		26		27				97.02						NA
-80.42	43.65	ELORA RC	6142286	43750.6	2019	10	12	0.58333	8.4		0.6		58		0		26		24				97.02						NA
-80.42	43.65	ELORA RC	6142286	43750.6	2019	10	12	0.625	8.1		0.3		58		0		24		23				97.05						NA
-80.42	43.65	ELORA RC	6142286	43750.7	2019	10	12	0.66667	7.7		0.7		61		0		25		20				97.05						NA
-80.42	43.65	ELORA RC	6142286	43750.7	2019	10	12	0.70833	7.2		0.3		61		0		23		15				97.08						NA
-80.42	43.65	ELORA RC	6142286	43750.8	2019	10	12	0.75	4.9		0.4		72		0		23		14				97.07						NA
-80.42	43.65	ELORA RC	6142286	43750.8	2019	10	12	0.79167	3.1		0		80		0		21		9				97.09						NA
-80.42	43.65	ELORA RC	6142286	43750.8	2019	10	12	0.83333	4.3		0.3		75		0		22		15				97.13						NA
-80.42	43.65	ELORA RC	6142286	43750.9	2019	10	12	0.875	3.1		-0.1		79		0		20		8				97.13						NA
-80.42	43.65	ELORA RC	6142286	43750.9	2019	10	12	0.91667	2.5		-0.4		81		0		19		6				97.12						NA
-80.42	43.65	ELORA RC	6142286	43751	2019	10	12	0.95833	1.8		-0.5		85		0		18		4				97.1						NA
-80.42	43.65	ELORA RC	6142286	43751	2019	10	13	0	0.7		-0.5		91		0		14		3				97.08						NA
-80.42	43.65	ELORA RC	6142286	43751	2019	10	13	0.04167	-0.1		-1.5		90		0		13		4				97.06				-1		NA
-80.42	43.65	ELORA RC	6142286	43751.1	2019	10	13	0.08333	0.1		-1		92		0		10		7				97.04						NA
-80.42	43.65	ELORA RC	6142286	43751.1	2019	10	13	0.125	1.2		-0.7		87		0		13		9				96.99						NA
-80.42	43.65	ELORA RC	6142286	43751.2	2019	10	13	0.16667	1.8		-0.5		85		0		14		12				96.99						NA
-80.42	43.65	ELORA RC	6142286	43751.2	2019	10	13	0.20833	2		-0.2		85		0		14		9				96.96						NA
-80.42	43.65	ELORA RC	6142286	43751.3	2019	10	13	0.25	1.9		-0.1		86		0		13		8				96.94						NA
-80.42	43.65	ELORA RC	6142286	43751.3	2019	10	13	0.29167	2.2		0.3		87		0		13		8				96.94						NA
-80.42	43.65	ELORA RC	6142286	43751.3	2019	10	13	0.33333	4.9		2.1		82		0		14		11				96.9						NA
-80.42	43.65	ELORA RC	6142286	43751.4	2019	10	13	0.375	8.9		5.2		77		0		17		18				96.84						NA
-80.42	43.65	ELORA RC	6142286	43751.4	2019	10	13	0.41667	11.7		6.3		69		0		19		23				96.8						NA
-80.42	43.65	ELORA RC	6142286	43751.5	2019	10	13	0.45833	13.1		6.7		65		0		18		26				96.73						NA
-80.42	43.65	ELORA RC	6142286	43751.5	2019	10	13	0.5	13.6		5.1		56		0		19		29				96.63						NA
-80.42	43.65	ELORA RC	6142286	43751.5	2019	10	13	0.54167	14.8		3.6		47		0		21		34				96.54						NA
-80.42	43.65	ELORA RC	6142286	43751.6	2019	10	13	0.58333	14.9		1.6		41		0		19		39				96.43						NA
-80.42	43.65	ELORA RC	6142286	43751.6	2019	10	13	0.625	14.7		3.1		46		0		20		35				96.42						NA
-80.42	43.65	ELORA RC	6142286	43751.7	2019	10	13	0.66667	14.3		2.4		44		0		20		36				96.38						NA
-80.42	43.65	ELORA RC	6142286	43751.7	2019	10	13	0.70833	13.5		2.3		46		0		21		32				96.41						NA
-80.42	43.65	ELORA RC	6142286	43751.8	2019	10	13	0.75	12.5		2.9		52		0		21		19				96.41						NA
-80.42	43.65	ELORA RC	6142286	43751.8	2019	10	13	0.79167	10.1		3.1		62		0		21		13				96.45						NA
-80.42	43.65	ELORA RC	6142286	43751.8	2019	10	13	0.83333	8.8		4.1		73		0		30		31				96.58						NA
-80.42	43.65	ELORA RC	6142286	43751.9	2019	10	13	0.875	7.1		2.7		74		0		30		21				96.71						NA
-80.42	43.65	ELORA RC	6142286	43751.9	2019	10	13	0.91667	6.8		2.2		72		0		28		11				96.58						NA
-80.42	43.65	ELORA RC	6142286	43752	2019	10	13	0.95833	6.5		2.4		75		0		28		14				96.61						NA
-80.42	43.65	ELORA RC	6142286	43752	2019	10	14	0	6.1		2.6		78		0		24		14				96.59						NA
-80.42	43.65	ELORA RC	6142286	43752	2019	10	14	0.04167	5.8		2.9		81		0		23		9				96.62						NA
-80.42	43.65	ELORA RC	6142286	43752.1	2019	10	14	0.08333	5.8		2.3		79		0		24		13				96.6						NA
-80.42	43.65	ELORA RC	6142286	43752.1	2019	10	14	0.125	5.5		1.5		75		0		26		18				96.62						NA
-80.42	43.65	ELORA RC	6142286	43752.2	2019	10	14	0.16667	5.3		1.7		78		0		25		15				96.68						NA
-80.42	43.65	ELORA RC	6142286	43752.2	2019	10	14	0.20833	5		1.5		78		0		25		13				96.73						NA
-80.42	43.65	ELORA RC	6142286	43752.3	2019	10	14	0.25	3.8		1.1		82		0		26		13				96.79						NA
-80.42	43.65	ELORA RC	6142286	43752.3	2019	10	14	0.29167	2.8		0.5		85		0		25		11				96.85						NA
-80.42	43.65	ELORA RC	6142286	43752.3	2019	10	14	0.33333	5		0.8		74		0		24		14				96.91						NA
-80.42	43.65	ELORA RC	6142286	43752.4	2019	10	14	0.375	6.7		0.8		66		0		26		26				96.94						NA
-80.42	43.65	ELORA RC	6142286	43752.4	2019	10	14	0.41667	7.8		-1.4		52		0		27		30				96.98						NA
-80.42	43.65	ELORA RC	6142286	43752.5	2019	10	14	0.45833	7.4		-1.1		55		0		27		23				97						NA
-80.42	43.65	ELORA RC	6142286	43752.5	2019	10	14	0.5	8		-2		49		0		26		31				97.02						NA
-80.42	43.65	ELORA RC	6142286	43752.5	2019	10	14	0.54167	7.8		-1.8		51		0		26		28				97.05						NA
-80.42	43.65	ELORA RC	6142286	43752.6	2019	10	14	0.58333	8.8		-2.2		46		0		26		31				97.04						NA
-80.42	43.65	ELORA RC	6142286	43752.6	2019	10	14	0.625	8.9		-1.1		49		0		26		28				97.1						NA
-80.42	43.65	ELORA RC	6142286	43752.7	2019	10	14	0.66667	8.7		-1.8		47		0		26		24				97.14						NA
-80.42	43.65	ELORA RC	6142286	43752.7	2019	10	14	0.70833	7.6		-1.6		52		0		28		18				97.21						NA
-80.42	43.65	ELORA RC	6142286	43752.8	2019	10	14	0.75	4.8		-0.7		67		0		25		11				97.24						NA
-80.42	43.65	ELORA RC	6142286	43752.8	2019	10	14	0.79167	4.4		-0.3		71		0		23		5				97.28						NA
-80.42	43.65	ELORA RC	6142286	43752.8	2019	10	14	0.83333	4.9		0.7		74		0		24		5				97.32						NA
-80.42	43.65	ELORA RC	6142286	43752.9	2019	10	14	0.875	5		1.4		77		0		23		3				97.35						NA
-80.42	43.65	ELORA RC	6142286	43752.9	2019	10	14	0.91667	5.3		1.8		78		0		23		7				97.36						NA
-80.42	43.65	ELORA RC	6142286	43753	2019	10	14	0.95833	5.4		1.9		78		0		22		8				97.38						NA
-80.42	43.65	ELORA RC	6142286	43753	2019	10	15	0	3.3		0.5		82		0		23		8				97.41						NA
-80.42	43.65	ELORA RC	6142286	43753	2019	10	15	0.04167	2.8		0.9		87		0		23		4				97.42						NA
-80.42	43.65	ELORA RC	6142286	43753.1	2019	10	15	0.08333	2.1		0.7		90		0				0				97.44						NA
-80.42	43.65	ELORA RC	6142286	43753.1	2019	10	15	0.125	0.9		-0.3		92		0				0				97.44						NA
-80.42	43.65	ELORA RC	6142286	43753.2	2019	10	15	0.16667	-0.1		-0.8		95		0				0				97.46						NA
-80.42	43.65	ELORA RC	6142286	43753.2	2019	10	15	0.20833	-0.8		-1.4		96		0				0				97.47						NA
-80.42	43.65	ELORA RC	6142286	43753.3	2019	10	15	0.25	-0.9		-1.4		97		0		7		3				97.48				-2		NA
-80.42	43.65	ELORA RC	6142286	43753.3	2019	10	15	0.29167	-0.7		-1.2		96		0				0				97.49						NA
-80.42	43.65	ELORA RC	6142286	43753.3	2019	10	15	0.33333	3.2		1.6		89		0				0				97.51						NA
-80.42	43.65	ELORA RC	6142286	43753.4	2019	10	15	0.375	6.4		3		79		0		21		7				97.5						NA
-80.42	43.65	ELORA RC	6142286	43753.4	2019	10	15	0.41667	8.8		1.5		60		0		22		13				97.41						NA
-80.42	43.65	ELORA RC	6142286	43753.5	2019	10	15	0.45833	10.4		1.3		53		0		19		20				97.33						NA
-80.42	43.65	ELORA RC	6142286	43753.5	2019	10	15	0.5	11.9		3.7		58		0		19		21				97.22						NA
-80.42	43.65	ELORA RC	6142286	43753.5	2019	10	15	0.54167	12.2		4.4		59		0		18		19				97.15						NA
-80.42	43.65	ELORA RC	6142286	43753.6	2019	10	15	0.58333	12.8		4.5		57		0		17		21				97.08						NA
-80.42	43.65	ELORA RC	6142286	43753.6	2019	10	15	0.625	13.7		4.4		54		0		18		14				96.98						NA
-80.42	43.65	ELORA RC	6142286	43753.7	2019	10	15	0.66667	13.3		4.8		56		0		17		13				96.91						NA
-80.42	43.65	ELORA RC	6142286	43753.7	2019	10	15	0.70833	11.8		5.5		66		0		14		10				96.9						NA
-80.42	43.65	ELORA RC	6142286	43753.8	2019	10	15	0.75	10.8		4.8		67		0		14		11				96.9						NA
-80.42	43.65	ELORA RC	6142286	43753.8	2019	10	15	0.79167	10.4		5		69		0		12		13				96.83						NA
-80.42	43.65	ELORA RC	6142286	43753.8	2019	10	15	0.83333	9.9		5.8		76		0		11		14				96.77						NA
-80.42	43.65	ELORA RC	6142286	43753.9	2019	10	15	0.875	9.3		6.3		81		0		11		17				96.66						NA
-80.42	43.65	ELORA RC	6142286	43753.9	2019	10	15	0.91667	9.5		6.4		81		0		10		20				96.53						NA
-80.42	43.65	ELORA RC	6142286	43754	2019	10	15	0.95833	9.7		6.9		82		0		9		20				96.37						NA
-80.42	43.65	ELORA RC	6142286	43754	2019	10	16	0	9.7		7.1		84		0		10		14				96.25						NA
-80.42	43.65	ELORA RC	6142286	43754	2019	10	16	0.04167	9.1		6.8		85		0		10		14				96.13						NA
-80.42	43.65	ELORA RC	6142286	43754.1	2019	10	16	0.08333	9.6		6.8		83		0		11		15				96.01						NA
-80.42	43.65	ELORA RC	6142286	43754.1	2019	10	16	0.125	10.7		7.4		80		0		13		12				95.86						NA
-80.42	43.65	ELORA RC	6142286	43754.2	2019	10	16	0.16667	10.3		8.6		89		0.1		14		14				95.73						NA
-80.42	43.65	ELORA RC	6142286	43754.2	2019	10	16	0.20833	10.5		9.8		95		2		20		11				95.75						NA
-80.42	43.65	ELORA RC	6142286	43754.3	2019	10	16	0.25	10.8		9.9		94		4.1		29		13				95.7						NA
-80.42	43.65	ELORA RC	6142286	43754.3	2019	10	16	0.29167	10.2		9.6		96		0.5		30		5				95.62						NA
-80.42	43.65	ELORA RC	6142286	43754.3	2019	10	16	0.33333	10		9.1		94		1.8		24		16				95.69						NA
-80.42	43.65	ELORA RC	6142286	43754.4	2019	10	16	0.375	9.1		8.1		93		2.1		15		7				95.53						NA
-80.42	43.65	ELORA RC	6142286	43754.4	2019	10	16	0.41667	8.5		7.5		93		2.2		18		21				95.64						NA
-80.42	43.65	ELORA RC	6142286	43754.5	2019	10	16	0.45833	7.9		6.9		93		0.2		22		17				95.59						NA
-80.42	43.65	ELORA RC	6142286	43754.5	2019	10	16	0.5	7.6		6.1		90		0.2		24		14				95.59						NA
-80.42	43.65	ELORA RC	6142286	43754.5	2019	10	16	0.54167	7.1		5.5		89		0		22		22				95.57						NA
-80.42	43.65	ELORA RC	6142286	43754.6	2019	10	16	0.58333	7.9		4.7		80		0		21		18				95.52						NA
-80.42	43.65	ELORA RC	6142286	43754.6	2019	10	16	0.625	8.1		5.1		82		0		25		19				95.49						NA
-80.42	43.65	ELORA RC	6142286	43754.7	2019	10	16	0.66667	7.4		5.5		88		0		24		14				95.49						NA
-80.42	43.65	ELORA RC	6142286	43754.7	2019	10	16	0.70833	7.2		5.5		89		0		24		17				95.46						NA
-80.42	43.65	ELORA RC	6142286	43754.8	2019	10	16	0.75	6		4.3		89		0		27		11				95.47						NA
-80.42	43.65	ELORA RC	6142286	43754.8	2019	10	16	0.79167	6.2		5.1		92		0		25		9				95.48						NA
-80.42	43.65	ELORA RC	6142286	43754.8	2019	10	16	0.83333	6.9		5.6		91		0		27		19				95.46						NA
-80.42	43.65	ELORA RC	6142286	43754.9	2019	10	16	0.875	6.2		5.5		95		0.3		27		20				95.47						NA
-80.42	43.65	ELORA RC	6142286	43754.9	2019	10	16	0.91667	6.3		5.3		94		0.2		30		23				95.45						NA
-80.42	43.65	ELORA RC	6142286	43755	2019	10	16	0.95833	6.1		5		93		0		30		26				95.46						NA
-80.42	43.65	ELORA RC	6142286	43755	2019	10	17	0	5.4		3.7		89		0		31		33				95.47						NA
-80.42	43.65	ELORA RC	6142286	43755	2019	10	17	0.04167	4.5		2.9		89		0		31		32				95.47						NA
-80.42	43.65	ELORA RC	6142286	43755.1	2019	10	17	0.08333	4.5		3.4		92		0		31		34				95.55						NA
-80.42	43.65	ELORA RC	6142286	43755.1	2019	10	17	0.125	4.8		3.9		94		0		32		26				95.61						NA
-80.42	43.65	ELORA RC	6142286	43755.2	2019	10	17	0.16667	5		3.9		92		0		32		25				95.64						NA
-80.42	43.65	ELORA RC	6142286	43755.2	2019	10	17	0.20833	5.1		3.5		90		0		32		26				95.69						NA
-80.42	43.65	ELORA RC	6142286	43755.3	2019	10	17	0.25	4.8		3.6		92		0		31		28				95.74						NA
-80.42	43.65	ELORA RC	6142286	43755.3	2019	10	17	0.29167	4.9		3.5		91		0		32		28				95.84						NA
-80.42	43.65	ELORA RC	6142286	43755.3	2019	10	17	0.33333	5		3.8		92		0		32		27				95.91						NA
-80.42	43.65	ELORA RC	6142286	43755.4	2019	10	17	0.375	5.4		3.8		90		0		32		29				95.95						NA
-80.42	43.65	ELORA RC	6142286	43755.4	2019	10	17	0.41667	5.9		3.6		85		0		32		33				95.98						NA
-80.42	43.65	ELORA RC	6142286	43755.5	2019	10	17	0.45833	5.4		3.1		85		0		32		30				96.08						NA
-80.42	43.65	ELORA RC	6142286	43755.5	2019	10	17	0.5	5.1		3.1		87		0		32		31				96.14						NA
-80.42	43.65	ELORA RC	6142286	43755.5	2019	10	17	0.54167	5.2		3.1		87		0		32		26				96.2						NA
-80.42	43.65	ELORA RC	6142286	43755.6	2019	10	17	0.58333	5.2		3.2		87		0		31		25				96.25						NA
-80.42	43.65	ELORA RC	6142286	43755.6	2019	10	17	0.625	5.3		3.3		87		0		32		25				96.3						NA
-80.42	43.65	ELORA RC	6142286	43755.7	2019	10	17	0.66667	5.6		2.9		83		0		32		27				96.34						NA
-80.42	43.65	ELORA RC	6142286	43755.7	2019	10	17	0.70833	5.8		2.6		80		0		32		32				96.39						NA
-80.42	43.65	ELORA RC	6142286	43755.8	2019	10	17	0.75	6		2.3		77		0		33		28				96.47						NA
-80.42	43.65	ELORA RC	6142286	43755.8	2019	10	17	0.79167	5.6		2.1		78		0		32		22				96.54						NA
-80.42	43.65	ELORA RC	6142286	43755.8	2019	10	17	0.83333	4.7		2.4		85		0		31		16				96.58						NA
-80.42	43.65	ELORA RC	6142286	43755.9	2019	10	17	0.875	4.1		2.5		89		0		31		13				96.62						NA
-80.42	43.65	ELORA RC	6142286	43755.9	2019	10	17	0.91667	4.2		2.2		87		0		31		15				96.65						NA
-80.42	43.65	ELORA RC	6142286	43756	2019	10	17	0.95833	2.3		0.6		88		0		30		9				96.7						NA
-80.42	43.65	ELORA RC	6142286	43756	2019	10	18	0	3.4		1.5		87		0		32		10				96.73						NA
-80.42	43.65	ELORA RC	6142286	43756	2019	10	18	0.04167	2.1		0.3		88		0		31		12				96.77						NA

-80.42	43.65	ELORA RC	6142286	43756.1	2019	10	18	0.08333	0.7		-0.2		94		0		28		8				96.82						NA
-80.42	43.65	ELORA RC	6142286	43756.1	2019	10	18	0.125	0.4		-0.3		95		0		28		9				96.85						NA
-80.42	43.65	ELORA RC	6142286	43756.2	2019	10	18	0.16667	-0.1		-0.6		96		0		29		10				96.92				-3		NA
-80.42	43.65	ELORA RC	6142286	43756.2	2019	10	18	0.20833	-0.6		-1.3		95		0		27		10				97				-4		NA
-80.42	43.65	ELORA RC	6142286	43756.3	2019	10	18	0.25	-1.2		-1.7		96		0		26		8				97.06				-4		NA
-80.42	43.65	ELORA RC	6142286	43756.3	2019	10	18	0.29167	0.2		-0.4		96		0		31		5				97.1						NA
-80.42	43.65	ELORA RC	6142286	43756.3	2019	10	18	0.33333	2.6		0.6		87		0		31		15				97.19						NA
-80.42	43.65	ELORA RC	6142286	43756.4	2019	10	18	0.375	5		0.5		73		0		33		19				97.21						NA
-80.42	43.65	ELORA RC	6142286	43756.4	2019	10	18	0.41667	5.5		-0.3		67		0		33		19				97.24						NA
-80.42	43.65	ELORA RC	6142286	43756.5	2019	10	18	0.45833	7		1		66		0		33		18				97.21						NA
-80.42	43.65	ELORA RC	6142286	43756.5	2019	10	18	0.5	7.8		0.7		61		0		31		16				97.23						NA
-80.42	43.65	ELORA RC	6142286	43756.5	2019	10	18	0.54167	8.5		0.3		57		0		30		15				97.2						NA
-80.42	43.65	ELORA RC	6142286	43756.6	2019	10	18	0.58333	9.4		1.3		57		0		28		15				97.18						NA
-80.42	43.65	ELORA RC	6142286	43756.6	2019	10	18	0.625	9.6		0.6		54		0		30		13				97.17						NA
-80.42	43.65	ELORA RC	6142286	43756.7	2019	10	18	0.66667	9.3		1.1		56		0		32		10				97.17						NA
-80.42	43.65	ELORA RC	6142286	43756.7	2019	10	18	0.70833	8.8		1.6		60		0		28		8				97.19						NA
-80.42	43.65	ELORA RC	6142286	43756.8	2019	10	18	0.75	6.2		1.2		70		0		30		6				97.21						NA
-80.42	43.65	ELORA RC	6142286	43756.8	2019	10	18	0.79167	4		1.1		81		0		32		4				97.23						NA
-80.42	43.65	ELORA RC	6142286	43756.8	2019	10	18	0.83333	2.9		1.1		88		0		29		4				97.26						NA
-80.42	43.65	ELORA RC	6142286	43756.9	2019	10	18	0.875	2.6		1.4		91		0		31		5				97.3						NA
-80.42	43.65	ELORA RC	6142286	43756.9	2019	10	18	0.91667	1.5		0.7		95		0		9		3				97.3						NA
-80.42	43.65	ELORA RC	6142286	43757	2019	10	18	0.95833	-0.2		-0.9		95		0				0				97.29						NA
-80.42	43.65	ELORA RC	6142286	43757	2019	10	19	0	-0.6		-1		97		0				0				97.28						NA
-80.42	43.65	ELORA RC	6142286	43757	2019	10	19	0.04167	-0.8		-1.3		96		0		2		3				97.28				-2		NA
-80.42	43.65	ELORA RC	6142286	43757.1	2019	10	19	0.08333	-0.7		-1.2		97		0		5		6				97.25				-3		NA
-80.42	43.65	ELORA RC	6142286	43757.1	2019	10	19	0.125	-1.4		-1.9		96		0		1		3				97.27				-2		NA
-80.42	43.65	ELORA RC	6142286	43757.2	2019	10	19	0.16667	-1.5		-2		97		0		5		5				97.27				-3		NA
-80.42	43.65	ELORA RC	6142286	43757.2	2019	10	19	0.20833	-2.3		-2.7		97		0				0				97.31						NA
-80.42	43.65	ELORA RC	6142286	43757.3	2019	10	19	0.25	-2.1		-2.6		97		0		4		4				97.29				-4		NA
-80.42	43.65	ELORA RC	6142286	43757.3	2019	10	19	0.29167	-1.6		-2.1		96		0		4		3				97.31				-3		NA
-80.42	43.65	ELORA RC	6142286	43757.3	2019	10	19	0.33333	1.2		0.2		93		0		6		7				97.29						NA
-80.42	43.65	ELORA RC	6142286	43757.4	2019	10	19	0.375	5.7		4		89		0		16		6				97.31						NA
-80.42	43.65	ELORA RC	6142286	43757.4	2019	10	19	0.41667	8.1		3.8		74		0		17		7				97.32						NA
-80.42	43.65	ELORA RC	6142286	43757.5	2019	10	19	0.45833	9.8		4.5		70		0		15		10				97.24						NA
-80.42	43.65	ELORA RC	6142286	43757.5	2019	10	19	0.5	11.2		4.3		63		0		16		14				97.19						NA
-80.42	43.65	ELORA RC	6142286	43757.5	2019	10	19	0.54167	12.1		3.9		57		0		16		12				97.12						NA
-80.42	43.65	ELORA RC	6142286	43757.6	2019	10	19	0.58333	12.8		4.1		56		0		13		10				97.02						NA
-80.42	43.65	ELORA RC	6142286	43757.6	2019	10	19	0.625	12.8		3.8		54		0		14		13				96.99						NA
-80.42	43.65	ELORA RC	6142286	43757.7	2019	10	19	0.66667	11.9		4.3		60		0		15		12				96.95						NA
-80.42	43.65	ELORA RC	6142286	43757.7	2019	10	19	0.70833	10.5		5		69		0		12		12				96.92						NA
-80.42	43.65	ELORA RC	6142286	43757.8	2019	10	19	0.75	7.6		4.7		82		0		9		13				96.9						NA
-80.42	43.65	ELORA RC	6142286	43757.8	2019	10	19	0.79167	6.1		4.1		87		0		11		10				96.91						NA
-80.42	43.65	ELORA RC	6142286	43757.8	2019	10	19	0.83333	5.2		4.1		92		0		10		11				96.83						NA
-80.42	43.65	ELORA RC	6142286	43757.9	2019	10	19	0.875	4.7		4		96		0		9		7				96.86						NA
-80.42	43.65	ELORA RC	6142286	43757.9	2019	10	19	0.91667	6.5		5.9		96		0		10		13				96.85						NA
-80.42	43.65	ELORA RC	6142286	43758	2019	10	19	0.95833	7.1		6.4		95		0		8		8				96.82						NA
-80.42	43.65	ELORA RC	6142286	43758	2019	10	20	0	6.5		5.9		96		0		11		12				96.74						NA
-80.42	43.65	ELORA RC	6142286	43758	2019	10	20	0.04167	7.2		6.8		98		0		10		11				96.73						NA
-80.42	43.65	ELORA RC	6142286	43758.1	2019	10	20	0.08333	7		6.6		97		0		8		8				96.72						NA
-80.42	43.65	ELORA RC	6142286	43758.1	2019	10	20	0.125	6.8		6.5		98		0		7		9				96.68						NA
-80.42	43.65	ELORA RC	6142286	43758.2	2019	10	20	0.16667	6.5		6.2		98		0		7		9				96.63						NA
-80.42	43.65	ELORA RC	6142286	43758.2	2019	10	20	0.20833	6.2		6		98		0		7		9				96.68						NA
-80.42	43.65	ELORA RC	6142286	43758.3	2019	10	20	0.25	5.9		5.7		98		0		7		8				96.73						NA
-80.42	43.65	ELORA RC	6142286	43758.3	2019	10	20	0.29167	5.8		5.6		98		0		8		8				96.73						NA
-80.42	43.65	ELORA RC	6142286	43758.3	2019	10	20	0.33333	6		5.8		98		0		6		5				96.77						NA
-80.42	43.65	ELORA RC	6142286	43758.4	2019	10	20	0.375	6.8		6.6		98		0				0				96.79						NA
-80.42	43.65	ELORA RC	6142286	43758.4	2019	10	20	0.41667	9.1		8.3		94		0		17		3				96.83						NA
-80.42	43.65	ELORA RC	6142286	43758.5	2019	10	20	0.45833	12.4		8		75		0		15		4				96.81						NA
-80.42	43.65	ELORA RC	6142286	43758.5	2019	10	20	0.5	15.4		7.9		61		0		19		9				96.8						NA
-80.42	43.65	ELORA RC	6142286	43758.5	2019	10	20	0.54167	16.9		7.1		52		0		22		10				96.79						NA
-80.42	43.65	ELORA RC	6142286	43758.6	2019	10	20	0.58333	17.5		7.3		51		0		20		10				96.8						NA
-80.42	43.65	ELORA RC	6142286	43758.6	2019	10	20	0.625	17.8		7.2		50		0		20		7				96.82						NA
-80.42	43.65	ELORA RC	6142286	43758.7	2019	10	20	0.66667	17.9		6.5		47		0		22		8				96.85						NA
-80.42	43.65	ELORA RC	6142286	43758.7	2019	10	20	0.70833	16.7		7.5		55		0		25		4				96.88						NA
-80.42	43.65	ELORA RC	6142286	43758.8	2019	10	20	0.75	13.9		7.6		66		0				0				96.94						NA
-80.42	43.65	ELORA RC	6142286	43758.8	2019	10	20	0.79167	11.7		7.8		77		0		10		7				96.99						NA
-80.42	43.65	ELORA RC	6142286	43758.8	2019	10	20	0.83333	9.6		7.5		87		0				0				97.05						NA
-80.42	43.65	ELORA RC	6142286	43758.9	2019	10	20	0.875	9.4		7.3		87		0		35		5				97.12						NA
-80.42	43.65	ELORA RC	6142286	43758.9	2019	10	20	0.91667	8.1		6.2		88		0		1		6				97.15						NA
-80.42	43.65	ELORA RC	6142286	43759	2019	10	20	0.95833	6.6		5.8		95		0		10		3				97.17						NA
-80.42	43.65	ELORA RC	6142286	43759	2019	10	21	0	6		4.9		93		0		4		5				97.19						NA
-80.42	43.65	ELORA RC	6142286	43759	2019	10	21	0.04167	6.5		5.6		94		0		5		10				97.19						NA
-80.42	43.65	ELORA RC	6142286	43759.1	2019	10	21	0.08333	6.5		5.5		94		0		5		7				97.22						NA
-80.42	43.65	ELORA RC	6142286	43759.1	2019	10	21	0.125	5.6		4.6		93		0		5		8				97.21						NA
-80.42	43.65	ELORA RC	6142286	43759.2	2019	10	21	0.16667	5.4		4.9		97		0		17		3				97.24						NA
-80.42	43.65	ELORA RC	6142286	43759.2	2019	10	21	0.20833	5		4.5		96		0		9		6				97.24						NA
-80.42	43.65	ELORA RC	6142286	43759.3	2019	10	21	0.25	5.3		4.5		95		0		10		7				97.31						NA
-80.42	43.65	ELORA RC	6142286	43759.3	2019	10	21	0.29167	5.9		5.2		95		0		7		9				97.31						NA
-80.42	43.65	ELORA RC	6142286	43759.3	2019	10	21	0.33333	8.2		6.7		90		0		9		17				97.33						NA
-80.42	43.65	ELORA RC	6142286	43759.4	2019	10	21	0.375	9		8.7		98		0		10		18				97.27						NA
-80.42	43.65	ELORA RC	6142286	43759.4	2019	10	21	0.41667	12.1		9.5		84		0		10		19				97.21						NA
-80.42	43.65	ELORA RC	6142286	43759.5	2019	10	21	0.45833	14		8.4		69		0		12		21				97.19						NA
-80.42	43.65	ELORA RC	6142286	43759.5	2019	10	21	0.5	14.8		8.5		66		0		11		23				97.12						NA
-80.42	43.65	ELORA RC	6142286	43759.5	2019	10	21	0.54167	14.7		7.4		62		0		11		21				97.04						NA
-80.42	43.65	ELORA RC	6142286	43759.6	2019	10	21	0.58333	14.3		8.5		68		0		10		27				96.89						NA
-80.42	43.65	ELORA RC	6142286	43759.6	2019	10	21	0.625	13.4		8.4		72		0		10		22				96.88						NA
-80.42	43.65	ELORA RC	6142286	43759.7	2019	10	21	0.66667	12.8		8.6		76		0		10		23				96.81						NA
-80.42	43.65	ELORA RC	6142286	43759.7	2019	10	21	0.70833	12		8.8		81		0		9		26				96.73						NA
-80.42	43.65	ELORA RC	6142286	43759.8	2019	10	21	0.75	11.4		8.6		83		0		10		29				96.65						NA
-80.42	43.65	ELORA RC	6142286	43759.8	2019	10	21	0.79167	11.6		8.4		81		0		10		27				96.6						NA
-80.42	43.65	ELORA RC	6142286	43759.8	2019	10	21	0.83333	12		9.2		83		0		10		24				96.55						NA
-80.42	43.65	ELORA RC	6142286	43759.9	2019	10	21	0.875	12.3		9.1		81		0		10		18				96.46						NA
-80.42	43.65	ELORA RC	6142286	43759.9	2019	10	21	0.91667	11.8		9.4		85		0.1		10		19				96.43						NA
-80.42	43.65	ELORA RC	6142286	43760	2019	10	21	0.95833	11.5		9.6		88		0.4		10		17				96.33						NA
-80.42	43.65	ELORA RC	6142286	43760	2019	10	22	0	11.5		9.7		89		0.4		14		11				96.41						NA
-80.42	43.65	ELORA RC	6142286	43760	2019	10	22	0.04167	11.2		10.2		93		2.7		13		16				96.35						NA
-80.42	43.65	ELORA RC	6142286	43760.1	2019	10	22	0.08333	11.4		10.7		96		3.6		13		11				96.3						NA
-80.42	43.65	ELORA RC	6142286	43760.1	2019	10	22	0.125	11.9		11.5		97		0.6		15		15				96.23						NA
-80.42	43.65	ELORA RC	6142286	43760.2	2019	10	22	0.16667	13		12.5		97		0.1		17		19				96.21						NA
-80.42	43.65	ELORA RC	6142286	43760.2	2019	10	22	0.20833	13.3		12.8		97		0.4		17		19				96.18						NA
-80.42	43.65	ELORA RC	6142286	43760.3	2019	10	22	0.25	13.6		13		96		0		16		17				96.07						NA
-80.42	43.65	ELORA RC	6142286	43760.3	2019	10	22	0.29167	13.6		13		96		0		17		18				96.14						NA
-80.42	43.65	ELORA RC	6142286	43760.3	2019	10	22	0.33333	13.7		12.9		95		0		18		19				96.14						NA
-80.42	43.65	ELORA RC	6142286	43760.4	2019	10	22	0.375	13.7		12.6		93		0.2		15		28				96.02						NA
-80.42	43.65	ELORA RC	6142286	43760.4	2019	10	22	0.41667	12.6		9.5		81		0.2		21		19				96.06						NA
-80.42	43.65	ELORA RC	6142286	43760.5	2019	10	22	0.45833	12		7.6		74		0		17		14				96.07						NA
-80.42	43.65	ELORA RC	6142286	43760.5	2019	10	22	0.5	14.5		8.8		68		0		19		24				95.98						NA
-80.42	43.65	ELORA RC	6142286	43760.5	2019	10	22	0.54167	15.1		8.9		66		0		19		28				95.92						NA
-80.42	43.65	ELORA RC	6142286	43760.6	2019	10	22	0.58333	15.4		3.7		45		0		20		41				95.94						NA
-80.42	43.65	ELORA RC	6142286	43760.6	2019	10	22	0.625	14.3		4.2		51		0		22		41				95.97						NA
-80.42	43.65	ELORA RC	6142286	43760.7	2019	10	22	0.66667	13.7		4.1		52		0		22		40				96.01						NA
-80.42	43.65	ELORA RC	6142286	43760.7	2019	10	22	0.70833	12		4.2		59		0		23		35				96.05						NA
-80.42	43.65	ELORA RC	6142286	43760.8	2019	10	22	0.75	10.6		3		59		0		23		26				96.08						NA
-80.42	43.65	ELORA RC	6142286	43760.8	2019	10	22	0.79167	9.8		3.3		64		0		23		24				96.16						NA
-80.42	43.65	ELORA RC	6142286	43760.8	2019	10	22	0.83333	9.4		4.4		71		0		23		27				96.17						NA
-80.42	43.65	ELORA RC	6142286	43760.9	2019	10	22	0.875	9.1		4.3		72		0		23		30				96.26						NA
-80.42	43.65	ELORA RC	6142286	43760.9	2019	10	22	0.91667	8.5		4.4		75		0		22		23				96.29						NA
-80.42	43.65	ELORA RC	6142286	43761	2019	10	22	0.95833	8		3.5		73		0		23		26				96.3						NA
-80.42	43.65	ELORA RC	6142286	43761	2019	10	23	0	7.7		3.7		76		0		22		22				96.28						NA
-80.42	43.65	ELORA RC	6142286	43761	2019	10	23	0.04167	7.9		3.4		73		0		23		20				96.27						NA
-80.42	43.65	ELORA RC	6142286	43761.1	2019	10	23	0.08333	7.6		3.6		76		0		22		25				96.29						NA
-80.42	43.65	ELORA RC	6142286	43761.1	2019	10	23	0.125	6.6		3.5		81		0		22		20				96.3						NA
-80.42	43.65	ELORA RC	6142286	43761.2	2019	10	23	0.16667	5.6		2.9		83		0		22		17				96.32						NA
-80.42	43.65	ELORA RC	6142286	43761.2	2019	10	23	0.20833	5.8		2.4		78		0		24		21				96.37						NA
-80.42	43.65	ELORA RC	6142286	43761.3	2019	10	23	0.25	4.9		2.2		82		0		21		15				96.43						NA
-80.42	43.65	ELORA RC	6142286	43761.3	2019	10	23	0.29167	4.8		2.5		85		0		21		17				96.46						NA
-80.42	43.65	ELORA RC	6142286	43761.3	2019	10	23	0.33333	6.2		3		80		0		22		24				96.53						NA
-80.42	43.65	ELORA RC	6142286	43761.4	2019	10	23	0.375	7.8		2.6		70		0		23		30				96.58						NA
-80.42	43.65	ELORA RC	6142286	43761.4	2019	10	23	0.41667	7.9		2.6		69		0		24		30				96.67						NA
-80.42	43.65	ELORA RC	6142286	43761.5	2019	10	23	0.45833	8.4		1.9		64		0		25		31				96.71						NA
-80.42	43.65	ELORA RC	6142286	43761.5	2019	10	23	0.5	9.6		0.8		54		0		24		35				96.72						NA
-80.42	43.65	ELORA RC	6142286	43761.5	2019	10	23	0.54167	10.1		0.4		51		0		24		36				96.71						NA
-80.42	43.65	ELORA RC	6142286	43761.6	2019	10	23	0.58333	10.9		0.7		49		0		25		36				96.79						NA
-80.42	43.65	ELORA RC	6142286	43761.6	2019	10	23	0.625	10.8		0.4		48		0		24		34				96.82						NA
-80.42	43.65	ELORA RC	6142286	43761.7	2019	10	23	0.66667	10.8		0.7		50		0		24		31				96.9						NA
-80.42	43.65	ELORA RC	6142286	43761.7	2019	10	23	0.70833	9.8		0.2		51		0		23		21				96.94						NA
-80.42	43.65	ELORA RC	6142286	43761.8	2019	10	23	0.75	7.8		-1.2		53		0		23		15				97.02						NA
-80.42	43.65	ELORA RC	6142286	43761.8	2019	10	23	0.79167	5.5		-1.3		61		0		21		10				97.1						NA

-80.42	43.65	ELORA RC	6142286	43761.8	2019	10	23	0.83333	5.3		0		68		0		22		12				97.16					NA
-80.42	43.65	ELORA RC	6142286	43761.9	2019	10	23	0.875	5.3		0.5		71		0		23		9				97.19					NA
-80.42	43.65	ELORA RC	6142286	43761.9	2019	10	23	0.91667	5.4		1.2		74		0		23		9				97.21					NA
-80.42	43.65	ELORA RC	6142286	43762	2019	10	23	0.95833	4.7		1.1		77		0		22		8				97.21					NA
-80.42	43.65	ELORA RC	6142286	43762	2019	10	24	0	6.6		2.7		76		0		22		8				97.21					NA
-80.42	43.65	ELORA RC	6142286	43762	2019	10	24	0.04167	7.5		2.7		72		0		21		9				97.19					NA
-80.42	43.65	ELORA RC	6142286	43762.1	2019	10	24	0.08333	8.7		4.5		75		0		22		11				97.16					NA
-80.42	43.65	ELORA RC	6142286	43762.1	2019	10	24	0.125	8.4		4.4		76		0		18		8				97.12					NA
-80.42	43.65	ELORA RC	6142286	43762.2	2019	10	24	0.16667	10.1		4.1		66		0		21		19				97.05					NA
-80.42	43.65	ELORA RC	6142286	43762.2	2019	10	24	0.20833	11.1		4.6		64		0		22		23				97.1					NA
-80.42	43.65	ELORA RC	6142286	43762.3	2019	10	24	0.25	9.8		5.4		74		0		23		22				97.16					NA
-80.42	43.65	ELORA RC	6142286	43762.3	2019	10	24	0.29167	8.7		4.4		74		0.2		23		18				97.21					NA
-80.42	43.65	ELORA RC	6142286	43762.3	2019	10	24	0.33333	9.3		4.5		72		0		25		14				97.33					NA
-80.42	43.65	ELORA RC	6142286	43762.4	2019	10	24	0.375	9		4.6		74		0		29		17				97.44					NA
-80.42	43.65	ELORA RC	6142286	43762.4	2019	10	24	0.41667	9.2		4.6		72		0		29		21				97.56					NA
-80.42	43.65	ELORA RC	6142286	43762.5	2019	10	24	0.45833	8.8		3.8		71		0		27		23				97.65					NA
-80.42	43.65	ELORA RC	6142286	43762.5	2019	10	24	0.5	10		3.8		65		0		27		22				97.68					NA
-80.42	43.65	ELORA RC	6142286	43762.5	2019	10	24	0.54167	11.2		3.8		61		0		27		28				97.67					NA
-80.42	43.65	ELORA RC	6142286	43762.6	2019	10	24	0.58333	10.4		2.4		58		0		27		24				97.7					NA
-80.42	43.65	ELORA RC	6142286	43762.6	2019	10	24	0.625	9.9		2.1		58		0		29		24				97.8					NA
-80.42	43.65	ELORA RC	6142286	43762.7	2019	10	24	0.66667	9.2		2.2		62		0		30		22				97.89					NA
-80.42	43.65	ELORA RC	6142286	43762.7	2019	10	24	0.70833	7.5		1.8		67		0		31		17				97.94					NA
-80.42	43.65	ELORA RC	6142286	43762.8	2019	10	24	0.75	5.1		1.3		76		0		29		9				98					NA
-80.42	43.65	ELORA RC	6142286	43762.8	2019	10	24	0.79167	4.6		1.1		78		0		32		7				98.01					NA
-80.42	43.65	ELORA RC	6142286	43762.8	2019	10	24	0.83333	2.9		0.7		85		0		21		4				98.06					NA
-80.42	43.65	ELORA RC	6142286	43762.9	2019	10	24	0.875	2.5		1		89		0				0				98.1					NA
-80.42	43.65	ELORA RC	6142286	43762.9	2019	10	24	0.91667	1.9		0.8		92		0		14		3				98.16					NA
-80.42	43.65	ELORA RC	6142286	43763	2019	10	24	0.95833	2.3		1.4		93		0				0				98.18					NA
-80.42	43.65	ELORA RC	6142286	43763	2019	10	25	0	2.7		1.2		90		0		36		6				98.16					NA
-80.42	43.65	ELORA RC	6142286	43763	2019	10	25	0.04167	3.2		1.5		89		0		2		4				98.14					NA
-80.42	43.65	ELORA RC	6142286	43763.1	2019	10	25	0.08333	3.4		1.9		90		0		9		4				98.15					NA
-80.42	43.65	ELORA RC	6142286	43763.1	2019	10	25	0.125	3.2		2.1		92		0		10		3				98.1					NA
-80.42	43.65	ELORA RC	6142286	43763.2	2019	10	25	0.16667	3.3		2.3		94		0		15		4				98.14					NA
-80.42	43.65	ELORA RC	6142286	43763.2	2019	10	25	0.20833	3.2		2.3		94		0		6		3				98.14					NA
-80.42	43.65	ELORA RC	6142286	43763.3	2019	10	25	0.25	3.2		2.3		94		0				0				98.11					NA
-80.42	43.65	ELORA RC	6142286	43763.3	2019	10	25	0.29167	3.3		2.3		93		0		36		4				98.07					NA
-80.42	43.65	ELORA RC	6142286	43763.3	2019	10	25	0.33333	4		3.1		93		0		35		4				98.13					NA
-80.42	43.65	ELORA RC	6142286	43763.4	2019	10	25	0.375	6		2.8		80		0				0				98.08					NA
-80.42	43.65	ELORA RC	6142286	43763.4	2019	10	25	0.41667	7.6		2.6		71		0		23		4				98.09					NA
-80.42	43.65	ELORA RC	6142286	43763.5	2019	10	25	0.45833	8.4		2.6		67		0		20		10				98.02					NA
-80.42	43.65	ELORA RC	6142286	43763.5	2019	10	25	0.5	9.5		2.5		62		0		22		10				97.94					NA
-80.42	43.65	ELORA RC	6142286	43763.5	2019	10	25	0.54167	9.8		2.2		59		0		25		13				97.88					NA
-80.42	43.65	ELORA RC	6142286	43763.6	2019	10	25	0.58333	9.5		2.3		61		0		26		10				97.85					NA
-80.42	43.65	ELORA RC	6142286	43763.6	2019	10	25	0.625	9.3		3.1		65		0		21		9				97.8					NA
-80.42	43.65	ELORA RC	6142286	43763.7	2019	10	25	0.66667	9		3		66		0		26		10				97.77					NA
-80.42	43.65	ELORA RC	6142286	43763.7	2019	10	25	0.70833	8.5		3.6		72		0		28		8				97.76					NA
-80.42	43.65	ELORA RC	6142286	43763.8	2019	10	25	0.75	7.9		3.8		75		0		28		13				97.81					NA
-80.42	43.65	ELORA RC	6142286	43763.8	2019	10	25	0.79167	6.9		3.9		81		0		27		9				97.78					NA
-80.42	43.65	ELORA RC	6142286	43763.8	2019	10	25	0.83333	6.2		3.5		83		0		30		15				97.81					NA
-80.42	43.65	ELORA RC	6142286	43763.9	2019	10	25	0.875	3.1		1.2		87		0		30		10				97.83					NA
-80.42	43.65	ELORA RC	6142286	43763.9	2019	10	25	0.91667	1.9		0.3		89		0		31		8				97.88					NA
-80.42	43.65	ELORA RC	6142286	43764	2019	10	25	0.95833	1.5		0.2		91		0		33		3				97.86					NA
-80.42	43.65	ELORA RC	6142286	43764	2019	10	26	0	0.8		-0.5		91		0				0				97.82					NA
-80.42	43.65	ELORA RC	6142286	43764	2019	10	26	0.04167	-0.8		-1.4		95		0				0				97.81					NA
-80.42	43.65	ELORA RC	6142286	43764.1	2019	10	26	0.08333	-1.3		-1.8		97		0		35		3				97.81				-2	NA
-80.42	43.65	ELORA RC	6142286	43764.1	2019	10	26	0.125	-1.9		-2.5		96		0				0				97.87					NA
-80.42	43.65	ELORA RC	6142286	43764.2	2019	10	26	0.16667	-2.4		-2.9		96		0				0				97.91					NA
-80.42	43.65	ELORA RC	6142286	43764.2	2019	10	26	0.20833	-2.4		-3		96		0				0				97.93					NA
-80.42	43.65	ELORA RC	6142286	43764.3	2019	10	26	0.25	-1.9		-2.4		96		0		1		5				97.92				-4	NA
-80.42	43.65	ELORA RC	6142286	43764.3	2019	10	26	0.29167	-2		-2.7		95		0		6		5				97.89				-4	NA
-80.42	43.65	ELORA RC	6142286	43764.3	2019	10	26	0.33333	0.5		-0.6		92		0		7		3				97.93					NA
-80.42	43.65	ELORA RC	6142286	43764.4	2019	10	26	0.375	4.3		0.6		77		0		9		10				97.9					NA
-80.42	43.65	ELORA RC	6142286	43764.4	2019	10	26	0.41667	6.6		0.3		64		0		10		20				97.83					NA
-80.42	43.65	ELORA RC	6142286	43764.5	2019	10	26	0.45833	7.6		-0.1		58		0		11		16				97.76					NA
-80.42	43.65	ELORA RC	6142286	43764.5	2019	10	26	0.5	8.9		0.8		57		0		12		18				97.72					NA
-80.42	43.65	ELORA RC	6142286	43764.5	2019	10	26	0.54167	7.9		1.2		63		0		12		18				97.64					NA
-80.42	43.65	ELORA RC	6142286	43764.6	2019	10	26	0.58333	7.9		2		66		0		10		20				97.57					NA
-80.42	43.65	ELORA RC	6142286	43764.6	2019	10	26	0.625	7.6		2.1		68		0		10		22				97.44					NA
-80.42	43.65	ELORA RC	6142286	43764.7	2019	10	26	0.66667	7.5		3		73		0		11		19				97.39					NA
-80.42	43.65	ELORA RC	6142286	43764.7	2019	10	26	0.70833	7		4		81		0		10		20				97.34					NA
-80.42	43.65	ELORA RC	6142286	43764.8	2019	10	26	0.75	6.6		4.7		87		0		11		19				97.29					NA
-80.42	43.65	ELORA RC	6142286	43764.8	2019	10	26	0.79167	6.5		5.6		94		0.7		9		22				97.18					NA
-80.42	43.65	ELORA RC	6142286	43764.8	2019	10	26	0.83333	7.1		6.2		94		0.3		10		32				96.99					NA
-80.42	43.65	ELORA RC	6142286	43764.9	2019	10	26	0.875	7.7		7		95		0.5		11		26				96.96					NA
-80.42	43.65	ELORA RC	6142286	43764.9	2019	10	26	0.91667	8.4		7.7		95		3.3		10		28				96.77					NA
-80.42	43.65	ELORA RC	6142286	43765	2019	10	26	0.95833	8.7		8.1		96		3.1		11		21				96.62					NA
-80.42	43.65	ELORA RC	6142286	43765	2019	10	27	0	8.8		8.1		96		2.8		11		20				96.43					NA
-80.42	43.65	ELORA RC	6142286	43765	2019	10	27	0.04167	9		8.5		96		2.8		10		19				96.32					NA
-80.42	43.65	ELORA RC	6142286	43765.1	2019	10	27	0.08333	9.4		8.8		96		7.6		10		21				96.19					NA
-80.42	43.65	ELORA RC	6142286	43765.1	2019	10	27	0.125	9.4		8.9		97		3.4		10		18				96.04					NA
-80.42	43.65	ELORA RC	6142286	43765.2	2019	10	27	0.16667	9.7		9.3		97		2.3		10		18				95.92					NA
-80.42	43.65	ELORA RC	6142286	43765.2	2019	10	27	0.20833	10.3		9.8		97		2.4		10		25				95.71					NA
-80.42	43.65	ELORA RC	6142286	43765.3	2019	10	27	0.25	11.4		10.8		96		0		13		22				95.6					NA
-80.42	43.65	ELORA RC	6142286	43765.3	2019	10	27	0.29167	12.5		11.7		95		0.3		14		28				95.45					NA
-80.42	43.65	ELORA RC	6142286	43765.3	2019	10	27	0.33333	11.7		10.3		91		2.1		21		42				95.56					NA
-80.42	43.65	ELORA RC	6142286	43765.4	2019	10	27	0.375	10		8.4		90		0.7		22		40				95.61					NA
-80.42	43.65	ELORA RC	6142286	43765.4	2019	10	27	0.41667	9.4		8.3		93		0		23		29				95.7					NA
-80.42	43.65	ELORA RC	6142286	43765.5	2019	10	27	0.45833	9.9		8.3		90		0		23		30				95.77					NA
-80.42	43.65	ELORA RC	6142286	43765.5	2019	10	27	0.5	10.3		9		92		0		24		32				95.85					NA
-80.42	43.65	ELORA RC	6142286	43765.5	2019	10	27	0.54167	10.5		9.4		93		0		25		29				95.94					NA
-80.42	43.65	ELORA RC	6142286	43765.6	2019	10	27	0.58333	10.2		9.5		95		0		26		31				96.08					NA
-80.42	43.65	ELORA RC	6142286	43765.6	2019	10	27	0.625	10.1		9.3		95		0		27		26				96.25					NA
-80.42	43.65	ELORA RC	6142286	43765.7	2019	10	27	0.66667	9.5		8.4		93		0		29		28				96.45					NA
-80.42	43.65	ELORA RC	6142286	43765.7	2019	10	27	0.70833	9.3		7.6		89		0		29		25				96.56					NA
-80.42	43.65	ELORA RC	6142286	43765.8	2019	10	27	0.75	9.5		7.4		87		0		28		26				96.7					NA
-80.42	43.65	ELORA RC	6142286	43765.8	2019	10	27	0.79167	9.4		7.6		89		0		28		15				96.78					NA
-80.42	43.65	ELORA RC	6142286	43765.8	2019	10	27	0.83333	9.1		7.9		92		0		26		17				96.94					NA
-80.42	43.65	ELORA RC	6142286	43765.9	2019	10	27	0.875	8.9		7.7		92		0		28		14				97					NA
-80.42	43.65	ELORA RC	6142286	43765.9	2019	10	27	0.91667	7.7		7		95		0		24		7				97.05					NA
-80.42	43.65	ELORA RC	6142286	43766	2019	10	27	0.95833	7.3		7		98		0		26		4				97.11					NA
-80.42	43.65	ELORA RC	6142286	43766	2019	10	28	0	7.4		7		98		0		22		5				97.11					NA
-80.42	43.65	ELORA RC	6142286	43766	2019	10	28	0.04167	6.2		5.9		98		0		27		8				97.14					NA
-80.42	43.65	ELORA RC	6142286	43766.1	2019	10	28	0.08333	5.5		5.2		98		0		24		4				97.2					NA
-80.42	43.65	ELORA RC	6142286	43766.1	2019	10	28	0.125	3.4		3.1		98		0		19		3				97.23					NA
-80.42	43.65	ELORA RC	6142286	43766.2	2019	10	28	0.16667	2.1		1.9		98		0		10		3				97.23					NA
-80.42	43.65	ELORA RC	6142286	43766.2	2019	10	28	0.20833	1.9		1.7		98		0				0				97.31					NA
-80.42	43.65	ELORA RC	6142286	43766.3	2019	10	28	0.25	1		0.8		98		0		32		3				97.39					NA
-80.42	43.65	ELORA RC	6142286	43766.3	2019	10	28	0.29167	0.7		0.4		98		0				0				97.4					NA
-80.42	43.65	ELORA RC	6142286	43766.3	2019	10	28	0.33333	2.4		2.1		98		0				0				97.41					NA
-80.42	43.65	ELORA RC	6142286	43766.4	2019	10	28	0.375	5.6		4.8		95		0		25		5				97.45					NA
-80.42	43.65	ELORA RC	6142286	43766.4	2019	10	28	0.41667	9.6		5.3		74		0		21		11				97.45					NA
-80.42	43.65	ELORA RC	6142286	43766.5	2019	10	28	0.45833	12.6		4.3		57		0		19		16				97.45					NA
-80.42	43.65	ELORA RC	6142286	43766.5	2019	10	28	0.5	13.9		5.5		57		0		21		16				97.43					NA
-80.42	43.65	ELORA RC	6142286	43766.5	2019	10	28	0.54167	15		5.8		54		0		20		15				97.37					NA
-80.42	43.65	ELORA RC	6142286	43766.6	2019	10	28	0.58333	15.7		5.9		52		0		19		16				97.35					NA
-80.42	43.65	ELORA RC	6142286	43766.6	2019	10	28	0.625	15.7		7.6		58		0		18		16				97.34					NA
-80.42	43.65	ELORA RC	6142286	43766.7	2019	10	28	0.66667	15.4		7.1		58		0		18		16				97.35					NA
-80.42	43.65	ELORA RC	6142286	43766.7	2019	10	28	0.70833	13.1		6.7		65		0		19		10				97.39					NA
-80.42	43.65	ELORA RC	6142286	43766.8	2019	10	28	0.75	11.4		5.5		67		0		18		8				97.43					NA
-80.42	43.65	ELORA RC	6142286	43766.8	2019	10	28	0.79167	10		5.2		72		0		18		7				97.45					NA
-80.42	43.65	ELORA RC	6142286	43766.8	2019	10	28	0.83333	6.9		4.5		85		0		12		4				97.42					NA
-80.42	43.65	ELORA RC	6142286	43766.9	2019	10	28	0.875	7		4.9		86		0		9		6				97.41					NA
-80.42	43.65	ELORA RC	6142286	43766.9	2019	10	28	0.91667	5.5		4.3		92		0		8		5				97.39					NA
-80.42	43.65	ELORA RC	6142286	43767	2019	10	28	0.95833	5		3.7		91		0		10		7				97.36					NA
-80.42	43.65	ELORA RC	6142286	43767	2019	10	29	0	4.3		3.2		93		0		7		6				97.35					NA
-80.42	43.65	ELORA RC	6142286	43767	2019	10	29	0.04167	5		4.3		95		0		10		10				97.29					NA
-80.42	43.65	ELORA RC	6142286	43767.1	2019	10	29	0.08333	3.9		3.3		96		0		3		3				97.26					NA
-80.42	43.65	ELORA RC	6142286	43767.1	2019	10	29	0.125	3.1		2.6		97		0		4		4				97.23					NA
-80.42	43.65	ELORA RC	6142286	43767.2	2019	10	29	0.16667	3.3		2.8		97		0		3		5				97.21					NA
-80.42	43.65	ELORA RC	6142286	43767.2	2019	10	29	0.20833	2.6		1.8		95		0		3		5				97.23					NA
-80.42	43.65	ELORA RC	6142286	43767.3	2019	10	29	0.25	3.2		2.7		97		0		7		4				97.24					NA
-80.42	43.65	ELORA RC	6142286	43767.3	2019	10	29	0.29167	3.2		2.9		98		0		9		7				97.25					NA
-80.42	43.65	ELORA RC	6142286	43767.3	2019	10	29	0.33333	5.4		5.1		98		0		9		3				97.3					NA
-80.42	43.65	ELORA RC	6142286	43767.4	2019	10	29	0.375	8.2		7.5		95		0		17		11				97.28					NA
-80.42	43.65	ELORA RC	6142286	43767.4	2019	10	29	0.41667	10.1		9.1		93		0		19		13				97.3					NA
-80.42	43.65	ELORA RC	6142286	43767.5	2019	10	29	0.45833	12.8		10.7		87		0		22		18				97.35					NA
-80.42	43.65	ELORA RC	6142286	43767.5	2019	10	29	0.5	13.7		10.3		80		0		24		22				97.34					NA
-80.42	43.65	ELORA RC	6142286	43767.5	2019	10	29	0.54167	13.6		9.3		75		0		26		19				97.39					NA

-80.42	43.65	ELORA RC	6142286	43767.6	2019	10	29	0.58333	13.7		9.1		73		0		25		18				97.41						NA
-80.42	43.65	ELORA RC	6142286	43767.6	2019	10	29	0.625	13.2		8.9		75		0		25		13				97.41						NA
-80.42	43.65	ELORA RC	6142286	43767.7	2019	10	29	0.66667	12.7		9.4		80		0		27		11				97.49						NA
-80.42	43.65	ELORA RC	6142286	43767.7	2019	10	29	0.70833	11.8		8.8		82		0		26		5				97.53						NA
-80.42	43.65	ELORA RC	6142286	43767.8	2019	10	29	0.75	11.7		8.6		81		0				0				97.57						NA
-80.42	43.65	ELORA RC	6142286	43767.8	2019	10	29	0.79167	10.5		9.2		92		0		29		5				97.63						NA
-80.42	43.65	ELORA RC	6142286	43767.8	2019	10	29	0.83333	9.6		8.8		94		0		34		5				97.67						NA
-80.42	43.65	ELORA RC	6142286	43767.9	2019	10	29	0.875	8.2		7.1		92		0		31		9				97.78						NA
-80.42	43.65	ELORA RC	6142286	43767.9	2019	10	29	0.91667	7.4		6.2		92		0		34		6				97.8						NA
-80.42	43.65	ELORA RC	6142286	43768	2019	10	29	0.95833	7.5		6.4		93		0		33		10				97.79						NA
-80.42	43.65	ELORA RC	6142286	43768	2019	10	30	0	6.9		6		94		0		29		7				97.84						NA
-80.42	43.65	ELORA RC	6142286	43768	2019	10	30	0.04167	6.1		4.9		92		0		35		11				97.88						NA
-80.42	43.65	ELORA RC	6142286	43768.1	2019	10	30	0.08333	6.1		4.7		91		0		33		12				97.9						NA
-80.42	43.65	ELORA RC	6142286	43768.1	2019	10	30	0.125	5.9		4.2		89		0		2		10				97.87						NA
-80.42	43.65	ELORA RC	6142286	43768.2	2019	10	30	0.16667	6.1		4.2		87		0		4		8				97.94						NA
-80.42	43.65	ELORA RC	6142286	43768.2	2019	10	30	0.20833	5.8		3.9		88		0		2		6				97.95						NA
-80.42	43.65	ELORA RC	6142286	43768.3	2019	10	30	0.25	5.7		4		89		0		36		4				98.06						NA
-80.42	43.65	ELORA RC	6142286	43768.3	2019	10	30	0.29167	5.6		3.3		85		0		5		13				97.96						NA
-80.42	43.65	ELORA RC	6142286	43768.3	2019	10	30	0.33333	5.8		3.2		84		0		7		7				98.08						NA
-80.42	43.65	ELORA RC	6142286	43768.4	2019	10	30	0.375	5.7		3		83		0		6		10				97.99						NA
-80.42	43.65	ELORA RC	6142286	43768.4	2019	10	30	0.41667	5.2		3.5		88		1.1		7		10				97.98						NA
-80.42	43.65	ELORA RC	6142286	43768.5	2019	10	30	0.45833	5.4		4		90		1.2		9		11				97.96						NA
-80.42	43.65	ELORA RC	6142286	43768.5	2019	10	30	0.5	5.6		4		90		1.1		6		12				97.83						NA
-80.42	43.65	ELORA RC	6142286	43768.5	2019	10	30	0.54167	5.4		4		90		1.1		6		14				97.72						NA
-80.42	43.65	ELORA RC	6142286	43768.6	2019	10	30	0.58333	5.3		4.2		93		1.1		6		14				97.63						NA
-80.42	43.65	ELORA RC	6142286	43768.6	2019	10	30	0.625	5.4		4.4		94		2		5		12				97.6						NA
-80.42	43.65	ELORA RC	6142286	43768.7	2019	10	30	0.66667	5.1		4.1		93		1.2		6		15				97.51						NA
-80.42	43.65	ELORA RC	6142286	43768.7	2019	10	30	0.70833	4.8		3.9		94		0.5		5		17				97.37						NA
-80.42	43.65	ELORA RC	6142286	43768.8	2019	10	30	0.75	4.9		4		94		0.5		5		14				97.37						NA
-80.42	43.65	ELORA RC	6142286	43768.8	2019	10	30	0.79167	4.9		4.2		95		2.2		6		18				97.26						NA
-80.42	43.65	ELORA RC	6142286	43768.8	2019	10	30	0.83333	5.1		4.4		95		1		6		18				97.11						NA
-80.42	43.65	ELORA RC	6142286	43768.9	2019	10	30	0.875	5.5		4.8		95		0.5		7		18				97.01						NA
-80.42	43.65	ELORA RC	6142286	43768.9	2019	10	30	0.91667	5.8		5.1		95		1		8		18				96.91						NA
-80.42	43.65	ELORA RC	6142286	43769	2019	10	30	0.95833	5.8		5.2		95		1.1		7		18				96.8						NA
-80.42	43.65	ELORA RC	6142286	43769	2019	10	31	0	5.9		5.2		95		1.1		4		13				96.74						NA
-80.42	43.65	ELORA RC	6142286	43769	2019	10	31	0.04167	6.1		5.6		97		4.4		7		12				96.61						NA
-80.42	43.65	ELORA RC	6142286	43769.1	2019	10	31	0.08333	6.4		6		97		2.3		5		7				96.55						NA
-80.42	43.65	ELORA RC	6142286	43769.1	2019	10	31	0.125	6.2		5.7		97		0.4		5		12				96.44						NA
-80.42	43.65	ELORA RC	6142286	43769.2	2019	10	31	0.16667	5.8		5.2		96		0		5		11				96.34						NA
-80.42	43.65	ELORA RC	6142286	43769.2	2019	10	31	0.20833	5.7		5.1		96		0.2		3		11				96.3						NA
-80.42	43.65	ELORA RC	6142286	43769.3	2019	10	31	0.25	5.5		5		97		0		5		11				96.23						NA
-80.42	43.65	ELORA RC	6142286	43769.3	2019	10	31	0.29167	5.4		5		98		0		4		7				96.19						NA
-80.42	43.65	ELORA RC	6142286	43769.3	2019	10	31	0.33333	5.3		5		98		0		4		10				96.13						NA
-80.42	43.65	ELORA RC	6142286	43769.4	2019	10	31	0.375	5.5		5.2		98		1.3		5		11				96.04						NA
-80.42	43.65	ELORA RC	6142286	43769.4	2019	10	31	0.41667	5.9		5.5		97		2		4		11				95.87						NA
-80.42	43.65	ELORA RC	6142286	43769.5	2019	10	31	0.45833	5.7		5		95		2.9		1		13				95.89						NA
-80.42	43.65	ELORA RC	6142286	43769.5	2019	10	31	0.5	6.2		5.5		95		2.4		5		19				95.61						NA
-80.42	43.65	ELORA RC	6142286	43769.5	2019	10	31	0.54167	6.8		6.2		96		1.7		5		16				95.42						NA
-80.42	43.65	ELORA RC	6142286	43769.6	2019	10	31	0.58333	7.4		6.5		94		0.7		3		16				95.33						NA
-80.42	43.65	ELORA RC	6142286	43769.6	2019	10	31	0.625	6.8		6.1		95		1.3		7		19				95.1						NA
-80.42	43.65	ELORA RC	6142286	43769.7	2019	10	31	0.66667	6.9		6.3		96		0.7		8		18				94.93						NA
-80.42	43.65	ELORA RC	6142286	43769.7	2019	10	31	0.70833	7.2		6.8		97		3.4		12		7				94.9						NA
-80.42	43.65	ELORA RC	6142286	43769.8	2019	10	31	0.75	7.4		7.1		98		1.7		22		5				94.98						NA
-80.42	43.65	ELORA RC	6142286	43769.8	2019	10	31	0.79167	8.6		8.1		97		0.5		21		27				95.02						NA
-80.42	43.65	ELORA RC	6142286	43769.8	2019	10	31	0.83333	8		6.1		88		0		22		34				95.05						NA
-80.42	43.65	ELORA RC	6142286	43769.9	2019	10	31	0.875	5.9		4.5		91		0		21		38				95.07						NA
-80.42	43.65	ELORA RC	6142286	43769.9	2019	10	31	0.91667	4.2		2.9		91		0		20		30				95.04						NA
-80.42	43.65	ELORA RC	6142286	43770	2019	10	31	0.95833	4.1		2.7		91		0		27		38				95.11						NA
