# Supplementary material for: Conservation and diversity of the pollen microbiome of Pan-American maize using PacBio and MiSeq
Source: Front Microbiol. 2023 Dec 21;14:1276241. doi: 10.3389/fmicb.2023.1276241 (PMC10764481; doi:10.3389/fmicb.2023.1276241)
Supplement: Supplementary file 11 [file Table_4.PDF]

Table S4. Summary of the taxonomic composition of the Pan-American maize pollen microbiome identified by FL-PacBio and V4-MiSeq sequencing platforms, aggregated at six taxonomic levels.

Taxonomic level		FL_PacBio		V4_MiSeq	
		Taxa count	Read count	Taxa count	Read count
**Total (Bacteria + Archaea)**		765	66,327	1448	1,599,974
**Total (Bacteria)**		765	66,327	1432	1,598,857
**Phylum**	**RA ≥ 1%**	4	66,298 (99.96%)	4	1,593,504 (99.66 %)
	**RA < 1%**	1	16 (0.02 %)	16	5166 (0.32 %)
	**Total**	5	66,314 (99.98%)	20	1,598,670 (99.99 %)
**Class**	**RA ≥ 1%**	5	66,291 (99.95%)	5	1,589,449 (99.41%)
	**RA < 1%**	3	23 (0.03 %)	40	9,130 (0.57%)
	**Total**	8	66,314 (99.98%)	45	1,598,579 (99.98%)
**Order**	**RA ≥ 1%**	6	65,068 (98.12 %)	6	1,511,786 (94.55 %)
	**RA < 1%**	14	1,246 (1.88 %)	89	83,730 (5.25 %)
	**Total**	20	66,314 (99.98%)	95	1,595,516 (99.79%)
**Family**	**RA ≥ 1%**	9	63,951 (96.42 %)	10	1,214,107 (75.94 %)
	**RA < 1%**	19	1,792 (2.72 %)	127	87,419 (6.72 %)
	**Total**	28	65,743 (99.12%)	137	1,301,526 (81.40%)
**Genus**	**RA ≥ 1%**	12	61,499 (92.72 %)	10	1,104,806 (69.10 %)
	**RA < 1%**	27	1,762 (2.78 %)	224	104,222 (6.51 %)
	**Total**	39	63,261 (95.38%)	234	1,209,028 (75.62%)
**Species**	**RA ≥ 1%**	9	35,278 (53.19 %)	5	54,595 (3.41 %)
	**RA < 1%**	37	3,453 (5.21 %)	46	2,670 (0.17 %)
	**Total**	46	38,731 (58.39%)	51	57,265 (3.58%)

All taxonomic levels were calculated for taxa that belong to Bacteria kingdom. Taxa with RA ≥ 1% represent dominant taxa, whereas those with RA < 1% represents rare taxa.
