# Supplementary material for: Conservation and diversity of the pollen microbiome of Pan-American maize using PacBio and MiSeq
Source: Front Microbiol. 2023 Dec 21;14:1276241. doi: 10.3389/fmicb.2023.1276241 (PMC10764481; doi:10.3389/fmicb.2023.1276241)
Supplement: Supplementary file 12 [file Table_5.PDF]

Table S5. The contribution of core dominant taxa to the pollen microbiome at the genus and species levels amongst maize accessions with ≥3 replicates.

	#Rep	Total taxa #	Taxon taxa #	% Taxon taxa #	Total read #	Taxon read #	% Taxon read #
***Pantoea* (215 taxa)**							
**Highland site of diversification**							
Chalqueno	**5**	353	181	**51.27**	8806	5471	**62.13**
Palomero Toluqueno	**4**	371	174	**46.9**	10670	4916	**46.07**
Bolita	**5**	389	179	**46.02**	5790	2830	**48.88**
Conico	**5**	286	133	**46.5**	5352	1704	**31.84**
B73	**5**	341	150	**43.99**	4540	1797	**39.58**
**Distant to maize origin**							
Dente Branco	**5**	315	101	**32.06**	4221	384	**9.1**
Kulli	**3**	271	90	**33.21**	2404	440	**18.3**
Cateto Nortista	**4**	366	73	**19.95**	5748	232	**4.04**
Chapalote	**4**	258	44	**17.05**	2983	103	**3.45**
Oloton	**3**	247	24	**9.72**	4925	97	**1.97**
***Pseudomonas* (80 taxa)**							
**Highland site of diversification**							
Chalqueno	**5**	353	16	**4.53**	8806	74	**0.84**
Palomero Toluqueno	**4**	371	32	**8.63**	10670	317	**2.97**
Bolita	**5**	389	38	**9.77**	5790	338	**5.84**
Conico	**5**	286	22	**7.69**	5352	93	**1.74**
B73	**5**	341	36	**10.56**	4540	418	**9.21**
**Distant to maize origin**							
Dente Branco	**5**	315	32	**10.16**	4221	2472	**58.56**
Kulli	**3**	271	37	**13.65**	2404	165	**6.86**
Cateto Nortista	**4**	366	50	**13.66**	5748	1077	**18.74**
Chapalote	**4**	258	27	**10.47**	2983	147	**4.93**
Oloton	**3**	247	10	**4.05**	4925	25	**0.51**
***Lactococcus* (115 taxa)**							
**Highland site of diversification**							
Chalqueno	**5**	353	2	**0.57**	8806	3	**0.03**
Palomero Toluqueno	**4**	371	13	**3.5**	10670	39	**0.37**
Bolita	**5**	389	15	**3.86**	5790	99	**1.71**
Conico	**5**	286	1	**0.35**	5352	1	**0.02**

B73	**5**	341	38	**11.14**	4540	681	**15**
**Distant to maize origin**							
Dente Branco	**5**	315	23	**7.3**	4221	120	**2.84**
Kulli	**3**	271	12	**4.43**	2404	71	**2.95**
Cateto Nortista	**4**	366	63	**17.21**	5748	1688	**29.37**
Chapalote	**4**	258	73	**28.29**	2983	1271	**42.61**
Oloton	**3**	247	64	**25.91**	4925	885	**17.97**
***Erwinia* (75 taxa)**							
**Highland site of diversification**							
Chalqueno	**5**	353	34	**9.63**	8806	99	**1.12**
Palomero Toluqueno	**4**	371	66	**17.79**	10670	4380	**41.05**
Bolita	**5**	389	59	**15.17**	5790	1459	**25.2**
Conico	**5**	286	39	**13.64**	5352	250	**4.67**
B73	**5**	341	22	**6.45**	4540	50	**1.1**
**Distant to maize origin**							
Dente Branco	**5**	315	50	**15.87**	4221	213	**5.05**
Kulli	**3**	271	67	**24.72**	2404	1337	**55.62**
Cateto Nortista	**4**	366	54	**14.75**	5748	292	**5.08**
Chapalote	**4**	258	6	**2.33**	2983	10	**0.34**
Oloton	**3**	247	19	**7.69**	4925	145	**2.94**
***P. ananatis* (180 taxa)**							
**Highland site of diversification**							
Chalqueno	**5**	353	156	**44.19**	5209	8806	**59.15**
Palomero Toluqueno	**4**	371	150	**40.43**	4305	10670	**40.35**
Bolita	**5**	389	146	**37.53**	2308	5790	**39.86**
Conico	**5**	286	108	**37.76**	1356	5352	**25.34**
B73	**5**	341	127	**37.24**	1660	4540	**36.56**
**Distant to maize origin**							
Dente Branco	**5**	315	78	**24.76**	320	4221	**7.58**
Kulli	**3**	271	71	**26.2**	331	2404	**13.77**
Cateto Nortista	**4**	366	55	**15.03**	177	5748	**3.08**
Chapalote	**4**	258	30	**11.63**	57	2983	**1.91**
Oloton	**3**	247	19	**7.69**	53	4925	**1.08**
***Ps. rhizosphaerae* (9 taxa)**							
**Highland site of diversification**							

Chalqueno	**5**	353	2	0.57	8806	3	0.03
Palomero Toluqueno	**4**	371	4	1.08	10670	20	0.19
Bolita	**5**	389	6	1.54	5790	42	0.73
Conico	**5**	286	2	0.7	5352	2	0.04
B73	**5**	341	4	1.17	4540	7	0.15
**Distant to maize origin**							
Dente Branco	**5**	315	5	1.59	4221	12	0.28
Kulli	**3**	271	5	1.85	2404	39	1.62
Cateto Nortista	**4**	366	9	2.46	5748	138	2.4
Chapalote	**4**	258	3	1.16	2983	5	0.17
Oloton	**3**	247	2	0.81	4925	6	0.12
***Lactococcus lactis* (74 taxa)**							
**Highland site of diversification**							
Chalqueno	**5**	353	1	0.28	8806	2	0.02
Palomero Toluqueno	**4**	371	3	0.81	10670	19	0.18
Bolita	**5**	389	5	1.29	5790	79	1.36
Conico	**5**	286	0	0	5352	0	0
B73	**5**	341	13	3.81	4540	610	13.44
**Distant to maize origin**							
Dente Branco	**5**	315	9	2.86	4221	94	2.23
Kulli	**3**	271	3	1.11	2404	58	2.41
Cateto Nortista	**4**	366	41	11.2	5748	1614	28.08
Chapalote	**4**	258	46	17.83	2983	1147	38.45
Oloton	**3**	247	47	19.03	4925	831	16.87
